# Caged Monomethyl Auristatin F (MMAF) for Cell-Specific Activation Using a 488-nm-Optimized Photolabile Group

**DOI:** 10.1021/jacsau.6c00203

**Published:** 2026-06-03

**Authors:** Yuhang Lai, Francesco Russo, Alexandre Fürstenberg, Nicolas Winssinger

**Affiliations:** School of Chemistry and Biochemistry, 27212 University of Geneva, CH-1211 Geneva, Switzerland

**Keywords:** PPG, coumarin, MMAF, cytotoxic, photocaged cell permeability

## Abstract

Photolabile protecting groups (PPGs) are central tools for achieving precise spatial and temporal control of bioactive molecules in living systems. Their practical utility is critically dependent on efficient uncaging under biologically compatible conditions. Here, we report a coumarin-based PPG specifically optimized for 488 nm excitation, a ubiquitous laser line in fluorescence microscopy, enabling uncaging under irradiation conditions comparable to those used for GFP imaging. We applied the optimized PPG to cage the C-terminal carboxylate of monomethyl auristatin F (cMMAF), converting a membrane-impermeant antimitotic agent into a cell-permeant prodrug that regenerates an impermeant MMAF upon photoactivation. We showcase its utility through the light-triggered release of MMAF using irradiance typically used for imaging. We demonstrate the ability to confine the pharmacological activity of MMAF to a single cell, sparing adjacent cells (neighboring effect).

## Graphical abstract

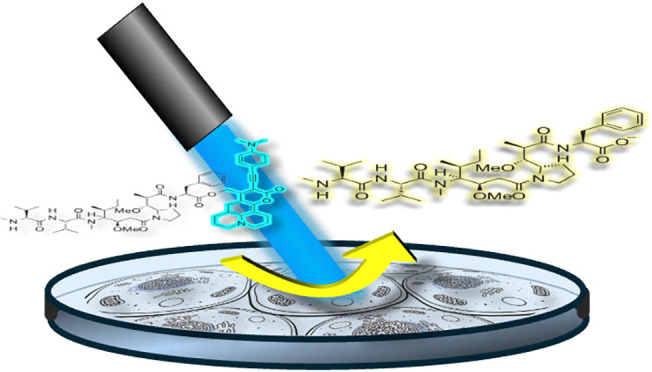

## Introduction

Photolabile protecting groups (PPGs) empower biomedical research with spatial and temporal regulation of pharmacological activity, potentially down to single-cell or subcellular resolution.^1–4^ The resolution that can be achieved largely depends on the method used and the diffusion of the uncaged pharmacological agent. The practical utility of PPGs depends on achieving an optimal balance between excitation wavelength, cage stability, and uncaging kinetics. While highly stable, classical nitrobenzyl-based PPGs require UV light, which is detrimental to cellular biochemistry; however, coumarin-based PPGs enabled uncaging with visible light.^5^ In these systems, photouncaging proceeds via heterolytic cleavage of a benzyl substituent at the 4-position, with excited-state stabilization of the resulting benzylic cation, lowering the transition-state energy for this otherwise unfavorable fragmentation (Figure 1a, excited-state polarization denoted by **δ**±). Ellis-Davies and co-workers advanced the field by reporting a 7-diethylamino coumarin derivative (DEAC450) with an extended conjugation at the 3-position that red-shifted the absorption to ∼450 nm.^6^ Subsequently, Zhu et al. showed that electron-rich styryl groups at this position, while slightly blue-shifting the absorption, increased photolysis efficiency by trapping the benzylic cation through intramolecular cyclization (Figure 1b).^7^ Conversely, electron-deficient vinylpyridinium red-shifted absorption but at the detriment of photolysis efficiency.^8,9^ Capitalizing on electron-rich styrene substituents, we previously demonstrated that energy transfer from a luciferase can drive photouncaging via bioluminescence resonance energy transfer.^10,11^ In parallel, Feringa and co-workers showed that increased substitution at the benzylic position accelerates cleavage, with hyperconjugation and allylic delocalization lowering the transition-state energy.^12^ Finally, cyclic surrogates of the diethylamino group have also been shown to improve photolysis.^13^ However, the combined effect of extended 3-position conjugation and higher benzylic substitution on coumarin’s uncaging performance has not been investigated.

**Figure 1:**
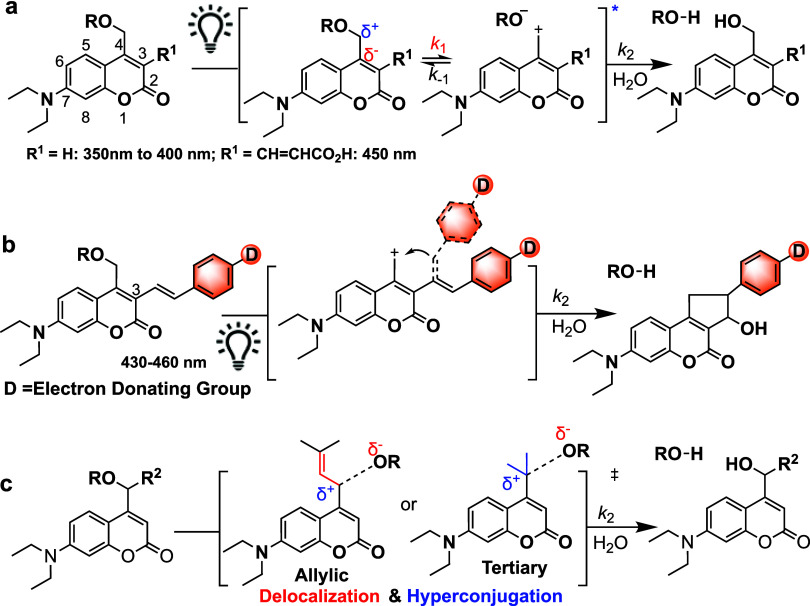
Coumarin-based photolabile protecting groups (PPG). (a) General structure of 7-diethyaminocoumarin and mechanism of photolysis. (b) Photolysis of coumarins with 3-styryl moiety can undergo cyclization upon photolysis. (c) Illustration of the transition-state stabilization achieved with benzylic substituents.

Photopharmacology using PPGs closely parallels that of photoswitchable drugs.^14–16^ Although photoswitches allow reversible interconversion between active and inactive states, PPGs enable a single, irreversible transition. However, drug diffusion can dilute uncaged compounds to subeffective concentrations, making photocaged drugs effectively short-acting and requiring repeated light exposure for sustained activity. We recently addressed this limitation by caging an acid group appended to a PLK inhibitor, which diffuses across membranes in its caged form but becomes impermeant upon uncaging, thereby confining activity to illuminated cells.^17^ For cellular or organoid studies, using microscope lasers for uncaging PPGs is most practical, as it allows simultaneous imaging and pharmacological activation. In that context, the caging group used previously was suboptimal for 488 nm excitation,^17^ a common laser line in microscopy. We therefore asked whether combining independent advances in coumarin photochemistry^5^ could yield a PPG with improved performance optimized for 488 nm excitation. Notably, Śtacko and co-workers also demonstrated delivery and release of the carboxylic acid cargo using an NIR dye to cage the acid. However, the release proceeds through a two-step mechanism (singlet oxygen-mediated dye fragmentation, elimination), thereby decoupling the photoirradiation from the cargo release.^18^

Herein, we report our findings with a photolabile protecting group optimized for 488 nm that displays significantly faster uncaging than prior coumarin-based PPGs and demonstrates its applicability to deliver an inhibitor of tubulin dynamics, MMAF, that is cell impermeant once uncaged.

## Results and Discussion

### Structure–Activity Relationship of Photolysis

We initiated our study with the synthesis of modified coumarins that combined (i) substituents capable of stabilizing the benzylic cation generated upon heterolytic C–O cleavage, (ii) extended conjugation at position 3 to red-shift absorption, and (iii) include a cyclic version of the 7-diethylamino group (Figure 2a; see the Supporting Information for synthetic details). To assess the impact of modifications, the relative cleavage rates were compared using competition experiments, monitoring conversions by NMR in an equimolar mixture of two compounds (Figure 2b). The absorption maximum across the 14 coumarins ranged from 377 to 480 nm, and we opted for a 455 nm LED lamp as a starting point for these comparisons. The relative rates were normalized for comparison across the range of compounds tested, as shown in Figure 2c (see Figure S1 for synthetic schemes, Figure S3 for individual comparisons, and Supporting Information for individual rates).

**Figure 2:**
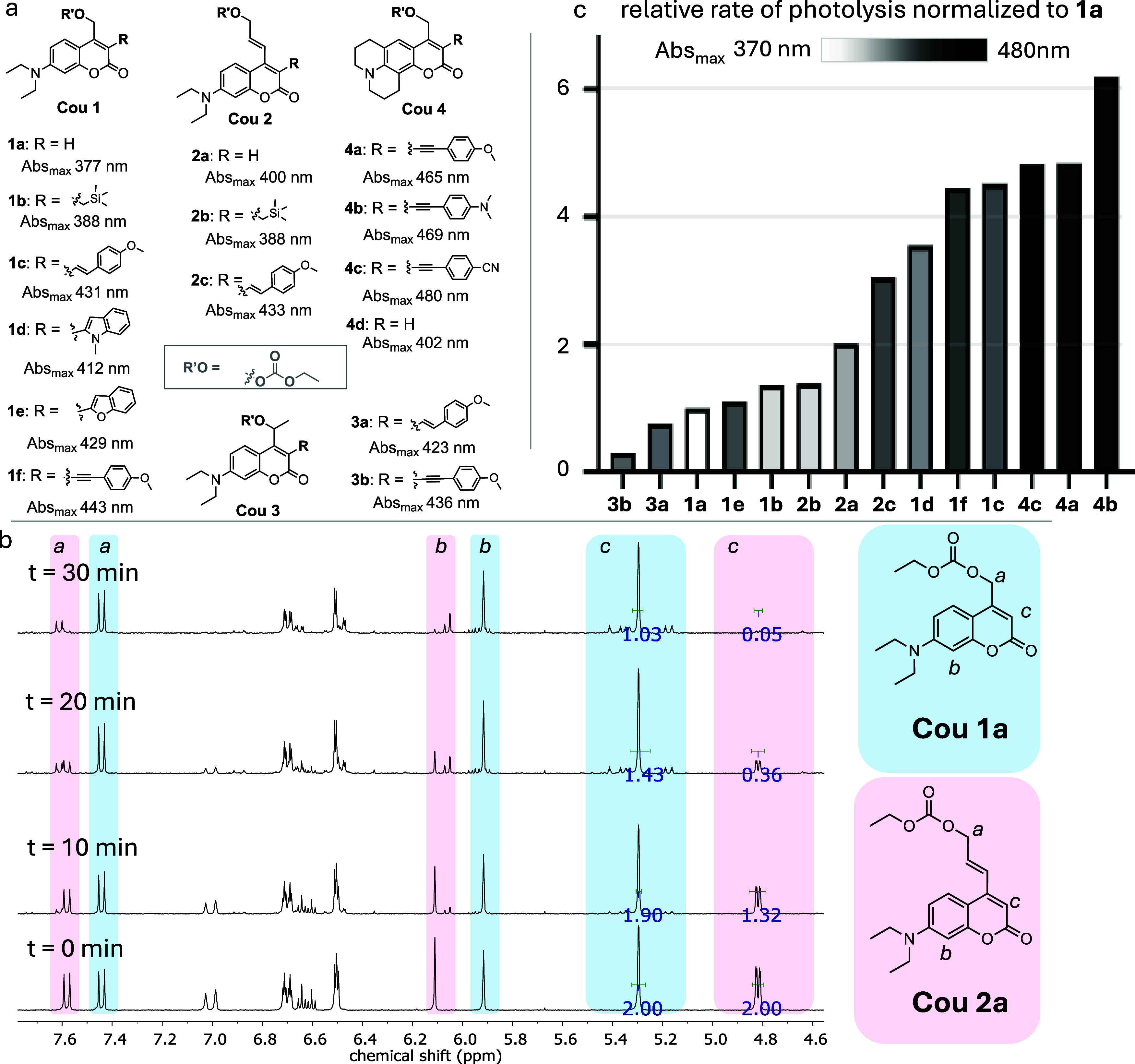
Structure–activity relationship of coumarin photolysis. (a) Structure of modified coumarins tested and absorption maxima. (b) Competition photolysis between **1a** and **2a** quantified by NMR to derive relative rates; signal from proton at position *a* were selected for further experiments since these signals are present on all tested substrates. Experimental conditions: irradiation with a 455 nm LED light source using a 2 mM substrate solution in DMSO-d6 and D_2_O (vDMSO:vH_2_O = 9:1). (c) Relative rates derived from competition experiments, normalized to the rate of **1a**. Shading of gray reflects the absorption max as shown in the scale bar.

Using the classical diethylamino coumarin **1a** as a reference, coumarin **2a**, which features an allylic connection to the caged residue, cleaved approximately twice as fast, while a methylene-silyl substituent at position 3 (**1b**), introduced to enhance hyperconjugative stabilization, provided a more modest acceleration (∼30% faster, **1b** vs **1a**). Because the irradiation wavelength is far from λ_max_ of the tested compounds and **2a** is red-shifted relative to **1a**, its faster cleavage rate likely reflects a combination of increased transition-state stabilization and higher ε_455_. However, combining these two modifications (**2b**) did not yield additive gains in photolysis rate.

Consistent with prior reports,^6,7^ extension of the π-system at position 3 was highly beneficial: **1c** exhibited ∼5-fold faster photolysis under these conditions (**1c** vs **1a**). Other substituents selected for their capacity to stabilize the transition state to the cyclization product with more nucleophilic alkenes (**1d**, **1e**) were found to be slower than **1c**. Replacing the trans-alkene of **1c** with an alkyne (**1f**) gave comparable rates but afforded an additional 12 nm red shift.

Notably, combining modifications that individually improve performance, benzylic stabilization (**2a** vs **1a**, 2-fold) and extended conjugation (**1c** vs **1a**, ∼5-fold), proved detrimental (**2c** vs **1c**). Even more strikingly, compounds **3a** and **3b** showed dramatically slower rates relative to **1c** and **1f** (6-fold and 14-fold, respectively), despite secondary benzylic substitution being beneficial in the absence of extended conjugation at position 3. Because the compared analogues have similar absorption maxima, we infer that the conjugation is not substantially perturbed; instead, steric clash in these doubly modified systems likely disrupts the necessary perpendicular alignment of the C–O bond with the coumarin π-system, compromising transition-state stabilization (Figure 3).

**Figure 3:**
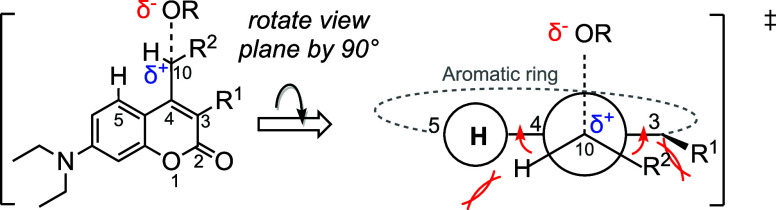
Schematic illustration of steric strain to achieve optimal perpendicular geometry required for heterolytic bond cleavage.

Given the slight red shift in the absorption of **1f** relative to **1c** and their comparable photolysis rates, combined with the lack of prior report of such aryl-alkyne-modified coumarin, we next examined how electron-donating versus electron-withdrawing substituents influence the aryl-alkyne-functionalized coumarins **4a**–**c**. Among these, the diethylamino derivative **4b** exhibited the fastest photolysis among the studied compounds. Considering the goal of a photocaging group optimized for microscopy uncaging using 488 nm, we next compared the photolysis kinetics of **4b** and **1c** under 488 nm laser irradiation using a substrate concentration of 20 μM and measuring conversion at intervals of 2 s with irradiance doses of 10 mJ/cm^2^, comparable to irradiance doses used in GFP imaging (10–100 mJ/cm^2^). Under these conditions, the half-life of photolysis was less than 6 s, corresponding to 22.5 mJ/cm^2^, while **1c** had significantly slower photolysis and **1a** or **4d** had insignificant photolysis under these conditions (Figure 4a). We also established that photolysis of **4b** did not yield a cyclization reaction, as is the case for **1c** (Figure 4b, signal of the benzylic position is denoted with the letter d and shows the characteristic chemical shift from a benzylic carbonate in the substrate to a benzylic alcohol in the product). To gain further insights into the performance of the caging group in biologically relevant conditions, we next measured the initial rate of photolysis in Dulbecco’s modified Eagle medium (DMEM: fluorobrite, phenol-red free) for **4b** and **1c** and calculated an absolute quantum of photodecomposition according to a previously reported method^19^ (Figure 4c). A possible difference when using media is the presence of flavin that can contribute to photocatalysis and energy transfer. A 5.6-fold difference in rate was observed between the photodecomposition of **4b** and **1c** under these conditions. We show that this difference stems from a slightly superior quantum yield of **4b** (6.1% vs 4.3%) coupled to a higher absorption coefficient at 488 nm (34,810 vs 8,510 M^–1^cm^–1^). Finally, we used a biotin conjugate to quantify the release of **4b** (R′ = biotin → R = H) from streptavidin beads in the presence of full media, affording comparable kinetics to the one measured in DMEM; however, the quantum yield in this case is likely biased by the fact that photolysis is performed from the streptavidin beads (see Supporting Information for detailed analysis).

**Figure 4:**
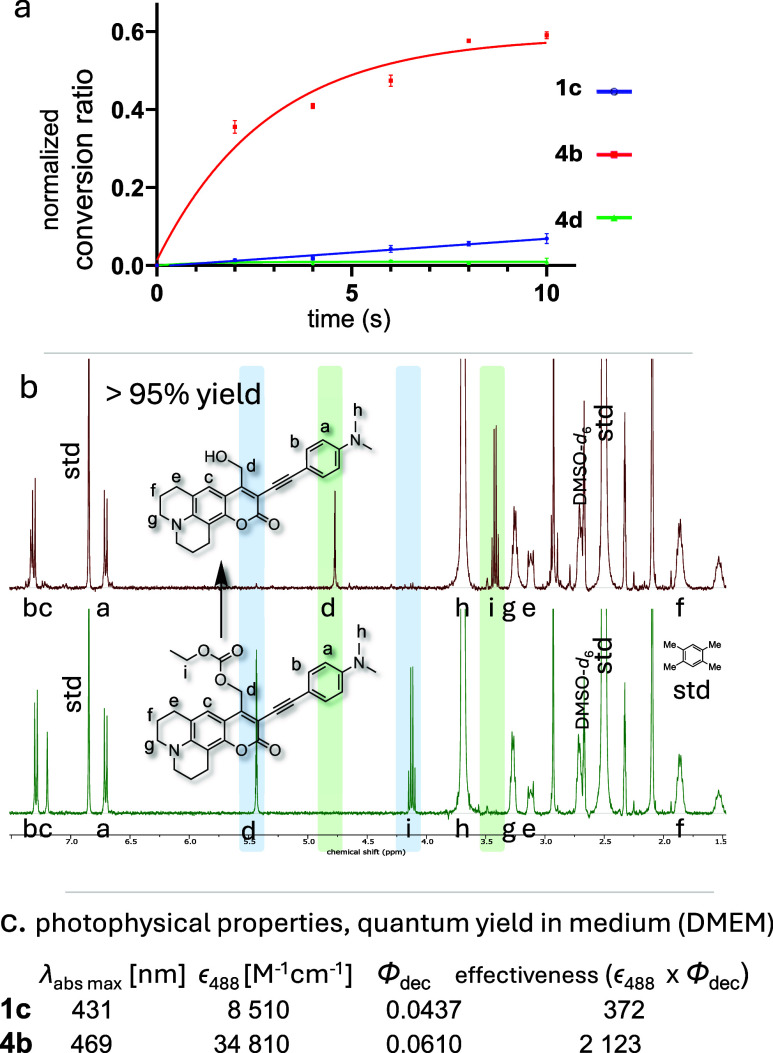
Characterization of photolysis product and competition reactions using a 488 nm laser. (a) Photolysis reactions of **Cou 4b** (red), **Cou 1c** (blue), and **Cou4d** (green) were independently carried out under 488 nm laser irradiation (5 mW/cm^2^) at a concentration of 20 μM in DMSO/H_2_O (v/v = 9:1) and expressed as a conversion ratio. (b) ^1^H NMR-monitored photolysis experiment of **Cou 4b**. Experimental conditions: irradiation with a 455 nm LED light source in DMSO-d6 and D_2_O (vDMSO: vH_2_O = 9:1). (c) Wavelength of absorption maxima, absorption coefficient at 488 nm, and quantum yield of photodecomposition. The quantum yield was measured from the decomposition of **1c** and **4b** in Dulbecco’s modified Eagle medium (fluorobrite).

### Caged Monomethyl Auristatin (cMMAF)

Monomethyl auristatin E (MMAE) is a potent cytotoxic used in multiple FDA-approved antibody-drug conjugates (ADC). In cell culture, MMAE’s cytotoxicity is in the low nM range. A closely related analogue, monomethyl auristatin F (MMAF), is known to be dramatically less cytotoxic in cell culture, yet MMAF has been shown to exhibit higher affinity for tubulin than MMAE.^20^ This discrepancy is rationalized by the presence of a carboxylate on MMAF that greatly diminishes cellular permeability compared to MMAE that lacks this carboxylate. Importantly, this carboxylate participates in interactions deep within the tubulin binding groove and contributes significantly to microtubule binding, accounting for the higher affinity of MMAF relative to MMAE. Based on these observations, we sought to leverage PPG **4b** for the synthesis of a caged MMAF (**cMMAF**). We reasoned that caging this functional group would not only abrogate tubulin binding but might also restore cellular permeability (Figure 5). In addition, given the intrinsic cellular impermeability of MMAF, we anticipated that its activity would remain confined to the cell in which uncaging occurred.

**Figure 5:**
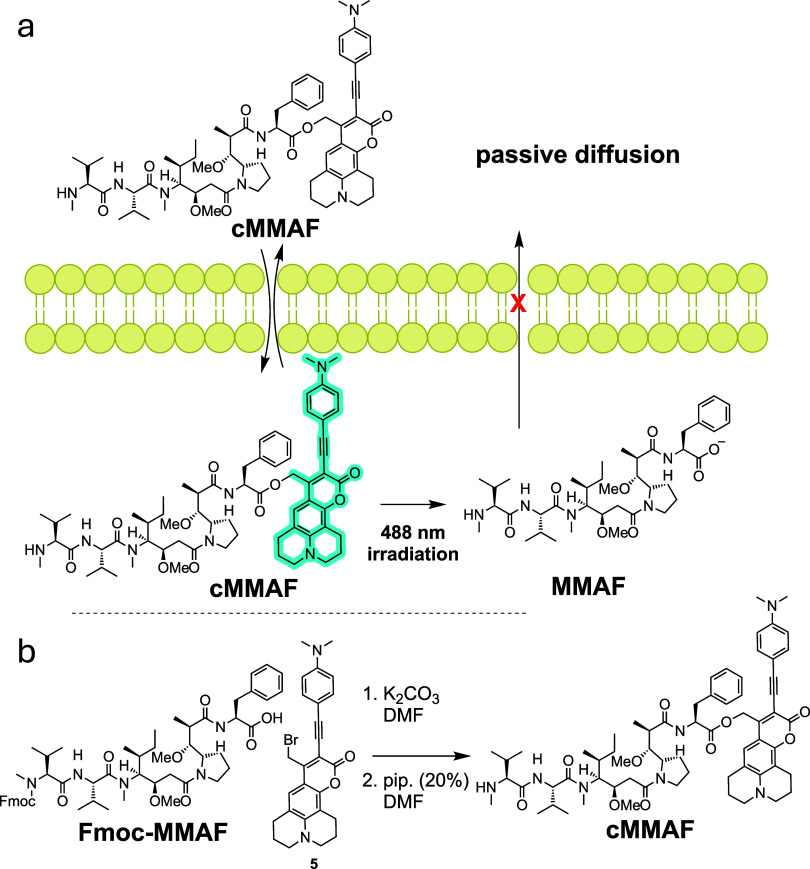
Confining MMAF to a cell following photolysis. (a) Schematic illustration; (b) synthesis of **cMMAF** from coumarin **5** and **Fmoc-MMAF**.

Concerned about possible epimerization during activation of the MMAF phenylalanine residue, we introduced the coumarin cage via nucleophilic substitution of coumarin bromide **5** with the Fmoc-protected MMAF carboxylate under mild basic conditions. Gratifyingly, under these conditions, Fmoc deprotection was slow relative to carboxylate alkylation, leading to a smooth conversion (more than 70% yield estimated by LC-MS). The Fmoc group was subsequently removed under standard conditions (20% piperidine in DMF), affording the desired **cMMAF** (35% overall isolated yield, following HPLC purification).

To monitor tubulin dynamics in real time while maintaining spectral separation from the uncaging chemistry, we employed a cell line constitutively expressing EB3–tdTomato,^21^ a protein that associates to the plus-end of growing tubulin and tracks tubulin network growth, visible as comets when imaging EB3 and frequently employed as a measure of microtubule dynamics.^22–28^ We confirmed that MMAF itself had no detectable effect on tubulin dynamics at concentrations up to 500 nM due to cellular impermeability. In contrast, cells exposed to cMMAF and imaged following media exchange exhibited residual intracellular fluorescence, indicating that cMMAF was cell permeable. However, prolonged incubation (5 h) led to perturbation of tubulin dynamics. We reasoned that lysosomal proteolytic activity could be responsible for background hydrolysis of cMMAF, yielding the active drug. This hypothesis is consistent with the substrate specificity of cathepsin L (P1: aromatic, P2: hydrophobic), as well as prior reports describing intracellular drug release from MMAF–mAb conjugates in lysosomes.^29,30^ To limit background retention due to hydrolysis, drug incubation was restricted to a maximum of 2 h prior to photoirradiation. Under these conditions, we observed a ∼20-fold lower cell viability IC_50_ for HeLa cell culture irradiated to achieve photouncaging of cMMAF vs the same conditions but kept in the dark (Figure 6a). The sub-nM IC_50_ of uncaged MMAF highlights the potency of this cytotoxic compound once the permeability limitation is removed. While the IC50 of **cMMAF** (10 nM) in dark conditions is far lower than that of MMAF (>100 nM), a residual pharmacological activity of **cMMAF** can be expected. In addition, the activity observed in the dark could arise from partial enzymatic hydrolysis of **cMMAF**, but this must be less than 5%, considering the difference in activity between caged and uncaged **cMMAF**. The same experiment performed with a control with butyric acid in lieu of MMAF (**cCTL**) showed no growth inhibition at concentrations up to 1 μM under irradiation in the dark (Figure 4a). We also confirmed that cMMAF is sufficiently stable in media, affording less than 14% decomposition after 12h, which is longer than any treatment used in the present work (see Supporting Information, section 9 for protocol).

**Figure 6:**
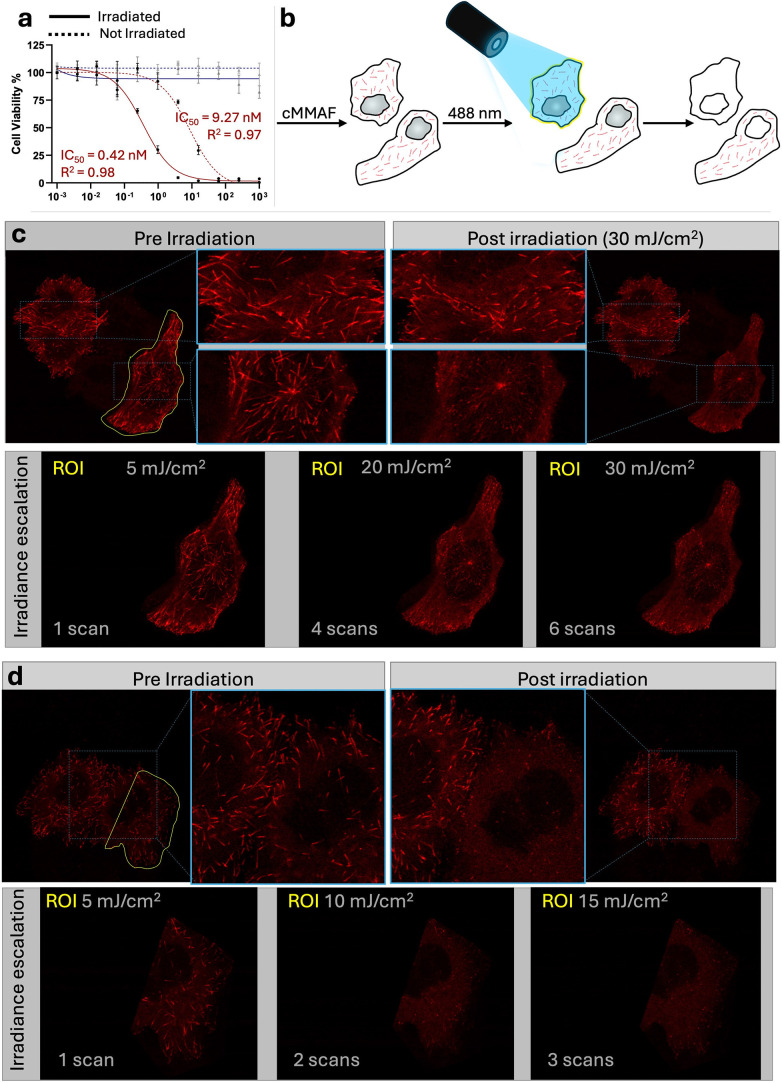
Resolution of MMAF uncaging and confinement. (a) Cell viability (HeLa) as a function of **cMMAF** concentration (nM, x-axis) for 2 h in the dark, followed by irradiation using 450 nm LED (6.5 mW/cm^2^) for 10 min (solid red curve) or maintained in the dark (dashed red curve). As control, cells were treated with the caged butyric acid using the same photolabile protecting group (**cCTL**; solid blue curve and dotted curve for irradiated and dark conditions, respectively). Vertical bars represent the standard deviation of independent biological replicates (*n* = 3). (b) Schematic representation of the workflow presented in c and d. In brief, after treatment with **cMMAF** (50 nM), the dish was placed under the microscope, and a single cell was imaged using a 488 nm laser line (area marked by the yellow circle line and designated as ROI) for uncaging **cMMAF**, followed by imaging of EB3–tdTomato (λ_ex_ 570) at the indicated time. (c) Top: a side-by-side comparison before and after uncaging at 488 nm. Blue squares represent zoom-ins of the cell body to facilitate EB3 comets visualization. Yellow lines represent the region of interest (ROI) irradiated to perform the uncaging reaction. On the bottom, sequential acquisitions of the irradiated cell show time-resolved phenotypical changes upon uncaging using different number of scans for the uncaging at 488 nm (each scan uses 2 μs dwell time per pixel with a power of 3.5 μW and a diffraction-limited spot size of 140,000 nm^2^, which corresponds to an irradiance dose of 5 mJ/cm^2^ per scan). (d) Top: side-by-side comparison of cells before and after uncaging via single-cell irradiation. Blue squares represent zoom-ins of the cell body to ease EB3 comets visualization. On the bottom, sequential acquisitions of the irradiated cell show time-resolved phenotypical changes upon uncaging at different numbers of scans (same conditions as in (c)).

We next asked whether the activity of MMAF could be confined to a single cell. Using EB3–tdTomato-expressing HeLa cells to visualize microtubule dynamics in real time, we performed uncaging using 488 nm irradiation in a confocal microscope within a defined region of interest (ROI, Figure 6b, highlighted in yellow: 2 μs/pixel, 3.5 μW with a diffraction-limited spot size of 140,000 nm^2^, which corresponds to an irradiance dose of 5 mJ/cm^2^). For cells in close proximity but not at confluence, irradiation of the ROI dramatically reduced the EB3 comets associated with microtubule growth in the targeted cell, while leaving comet density in the neighboring cell unchanged (Figure 6c). Qualitative analysis of the comets as a function of irradiance dose suggests that a single scan (5 mJ/cm^2^) is enough to produce a discernible reduction of comet density; 4 scans (20 mJ/cm^2^) yield a disappearance of the vast majority of comets. Collectively, these data point to a fast pharmacological effect that is achieved at a total irradiance dose well below the levels used in FRAP. The same treatment with a control using **cCTL** did not yield a qualitative change in comet number or intensity, indicating that this irradiance is insufficient for tdTomato photobleaching or laser induced tubulin damage (see Supporting Information, Figure S4).

We then considered a more stringent case where two cells are in direct contact and asked whether pharmacological activity could still be restricted to a single cell. To ensure that only the target cell was exposed to uncaged MMAF, irradiation was limited to a subset of the target cell, leaving a large margin from the cell–cell interface (Figure 6d). Gratifyingly, despite this restricted irradiation area (ROI < cell area), the uncaged MMAF rapidly diffused throughout the targeted cell and impacted the tubulin dynamics within the whole cell beyond the irradiated area, but did not spread to the adjacent cell. The marked difference in comet density between the two cells strongly supports the assumption that MMAF does not diffuse into neighboring cells due to its membrane-impermeant nature. Collectively, these experiments demonstrate that 1–4 scans in a confocal microscope are sufficient to uncage a dose of cMMAF adequate to dramatically alter tubulin dynamics.

There is significant interest in red-shifted PPGs^31^ with recent reports achieving uncaging at NIR wavelengths for deeper tissue penetration^32–35^ and orthogonal deprotection.^36^ However, the lower energy of a 700 nm photon relative to a 480 nm photon (1.77 and 2.54 eV, respectively) inherently requires a more fragile connection between the PPG and the caged molecule (0.77 eV; ca. 18 kcal/mol) for comparable heterolytic bond cleavage kinetics, assuming that the activation energy is primarily derived from the difference between the carbocation and the excited-state energy. Alternatively, two-photon activation with >700 nm light allows deep tissue penetration with very accurate spatial resolution. **cMMAF** was found to be efficiently uncaged using two-photon irradiation (976 nm, Figure 7), suggesting that coumarin **4b** and **cMMAF** could be considered for applications requiring deeper tissue penetration of light.

**Figure 7:**
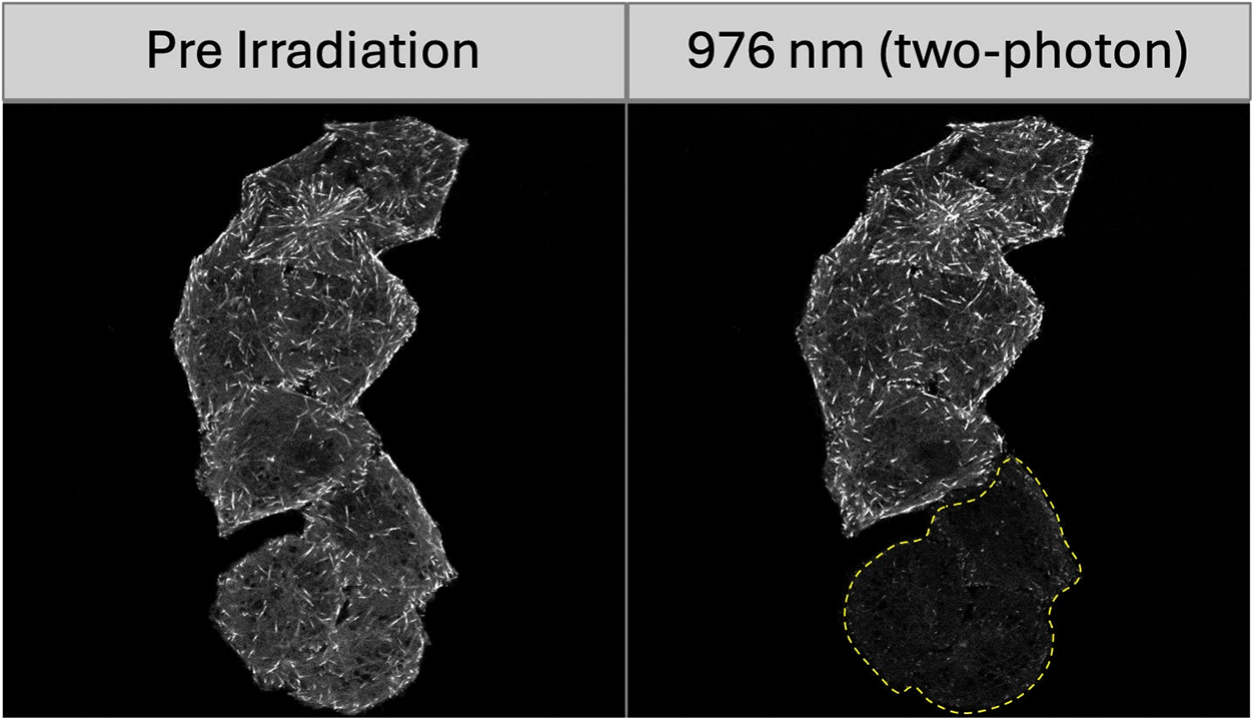
Uncaging of cMMAF using two-photon irradiation (976 nm). Cells were treated to the same conditions as for Figure 6 (50 nm **cMMAF**), and comets were imaged (EB3–tdTomato: λ_ex_ 570 nm); one cell was selected for two-photon irradiation at 976 nm (ROI circled in yellow) using two scans (total of 4 μs dwell) and comets were imaged again across the whole area as before two-photon irradiation.

## Conclusion

We developed a coumarin-based photolabile protecting group engineered for efficient uncaging under 488 nm excitation, a standard laser line in fluorescence microscopy. Systematic structure–activity studies identify substituent patterns that maximize effective photolysis under these conditions. Notably, modifications that individually enhance photolysis can become antagonistic when combined, consistent with steric and conformational constraints that disrupt the orbital alignment required for efficient fragmentation. The resulting optimized coumarin-based PPG exhibits a dramatically faster photolysis compared to best-in-class coumarins at 488 nm. The efficient photolysis facilitates uncaging under irradiation conditions compatible with routine live-cell imaging. We leveraged this 488-nm-optimized PPG to create a photocaged derivative of MMAF by masking its C-terminal carboxylate. In its caged form, the compound shows cellular uptake, while photoactivation regenerates MMAF, which we show does not diffuse to neighboring cells. This permeability switch provides an effective strategy to confine pharmacological activity to illuminated cells. Using EB3–tdTomato as a real-time reporter of microtubule growth, we demonstrate that brief 488 nm irradiation within a defined ROI rapidly suppresses microtubule dynamics in the targeted cell while sparing neighboring cells, including under conditions where cells are in direct contact. Notably, the spectral resolution between the PPG and tdTomato or other red-synthetic dyes enables fluorescence imaging without uncaging. Together, these results establish a broadly applicable platform for single-cell-resolved photopharmacology using standard microscopy instrumentation, and they provide practical design principles for next-generation visible-light PPGs tailored to biologically relevant excitation wavelengths.

## Supplementary Material



## Data Availability

All raw data have been deposited on Zenodo.

## Notes

The Supporting Information is available free of charge at https://pubs.acs.org/doi/10.1021/jacsau.6c00203. Raw data is availabe from https://doi.org/10.5281/zenodo.20414935

Full characterization of synthetic products and procedures (PDF)
