# Supplementary material for: Caged Monomethyl Auristatin F (MMAF) for Cell-Specific Activation Using a 488-nm-Optimized Photolabile Group
Source: JACS Au. 2026 Jun 3;6(6):3230–8. doi: 10.1021/jacsau.6c00203 (PMC13291978; doi:10.1021/jacsau.6c00203)
Supplement: Supplementary file 1 [file au6c00203_si_001.pdf]

Caged monomethyl auristatin F (MMAF) for cell specific activation using a 488 nm-optimized photolabile group

Yuhang Lai, Francesco Russo, Alexandre Fürstenberg, Nicolas Winssinger*

School of Chemistry and Biochemistry, University of Geneva, Geneva, Switzerland.

Supporting Information

1. Abbreviations 2

2. General methods 2

3. Compound synthesis and characterization 4

Figure S1 6

Figure S2 27

4.Competition experiments 30

Figure S3 31

5. Fluoresence emission spectra of Cou4a, 4b, 4c 46

7.Reaction rate analysis and quantum yield calculation 47

8. Biological activty 54

Figure S4 56

9. Stability of cMMAF in media 57

10.Reference 58

11. Annex: NMR spectra 58

1. Abbreviations

ACN	Acetonitrile
^{t}Bu	*tert*-butyl
$ClCO_2Et$	Ethyl chloroformate
DCM	Dichloromethane
DMF	Dimethylformamide
DMAP	4-(Dimethylamine)pyridine
DMSO	Dimethyl sulfoxide
DIC	N, N′-isopropyl carbodiimide
EtOH	Ethanol
ESI	Electrospray ionization
EtOAc	Ethyl acetate
Fmoc	Fluorenyl methoxycarbonyl
HPLC	High-Performance Liquid Chromatography
HRMS	High-Resolution Mass Spectrometry
LG	Leaving Group
Me	Methyl group
MeOH	Methanol
MS	Mass spectrometry
NBS	N-Bromo succinimide
NEt_3	Triethylamine
TBS	Tert-Butyl dimethyl silyl group
TFA	Trifluoroacetic acid
THF	Tetrahydrofuran
ROI	Region of interest

2. General methods

All reagents and solvents for the organic synthesis were purchased from commercial sources and were used without further purification. Unless otherwise specified, all synthetic reactions were performed under a nitrogen atmosphere. HPLC purification was performed with an Agilent Technologies 1260 infinity HPLC using a ZORBAX 300SB−C18 column (9.4×250 mm). LC–MS spectra were recorded on a DIONEX Ultimate 3000 UHPLC (condition for elution gradient: 0 min, A : B=100:0; 4 min, A : B=10 : 90; solution A: 0.01 % aqueous TFA solution; solution B, 0.01 % TFA in HPLC grade acetonitrile; flow rate: 0.750 mL/min) with a Thermo LCQ Fleet Mass Spectrometer System using PINNACLE DB C18 column (1.9 μm, 50×2.1 mm) operated in positive mode. All the LC–MS spectra were measured by electrospray ionization (ESI), linear gradient 0 to 100 %. LC–HRMS analyses were performed on a Xevo G2 TOF instrument in positive ion mode using a PINNACLE DB C18 column (1.9 μm, 50 × 2.1 mm). Elution was carried out with a linear gradient from 5% to 95% solvent B over 10 min, where solvent A was water with 0.1% formic acid and solvent B was acetonitrile with 0.1% formic acid. Photolysis was quantified using control compound X (MW = X) as an internal standard for normalization.
MALDI-TOF Mass spectra were measured using a Bruker Daltonics Autoflex spectrometer operated in positive mode. The samples were analyzed using 2,5-dihydroxybenzoic acid (DHB)

matrix. High-resolution mass spectra (HR-MS) were obtained on a Xevo G2 TOF spectrometer (Ionization mode: ESI positive polarity; Mobile phase: MeOH 100 µl/min). Thin layer chromatography (TLC) was performed on plates of silica precoated with 0.25 mm Kieselgel 60 F254 from Merck. Flash chromatography was performed using silica gel SiliaFlash® P60 (230–400 mesh) from Silicycle. Reverse phase column chromatography was performed with Isolera Biotage using SNAP Cartridge KP-C18HS of 12 g with a linear gradient of 5% ACN to 100% ACN in water with 0.1% TFA in 1 h. 1H and 13C NMR spectra were recorded on Bruker AVANCE 3 HD NMR spectrometer 400 MHz and Bruker 500 UltraShield NMR spectrometer 500 MHz. UV–vis spectra were recorded on a Jasco V-650 spectrophotometer using a deuterium lamp (190–350 nm) and a halogen lamp (330–900 nm) controlled by Spectra Manager II software with temperature regulation provided by a programmable system. Fluorescence measurements were performed on a Horiba Scientific Fluoromax-3 (or Fluoromax-Plus) spectrofluorometer equipped with a 150 W xenon lamp using FluorEssence™ software and a Peltier cuvette holder. Uncaging of cMMAF on cells was carried out by a LED light 7.5 cm above the plate (455 nm, 1W: Thorlabs, part number M455L2-C1 – www.thorlabs.com). The power of the LED lamp and microscopy laser was measured by a power meter (PM100USB - Thorlabs). Fluorescence imaging was carried out using a Leica Stellaris 8 FALCON confocal microscope equipped with a Leica white light laser (l = 440 to 750 nm) using a 63x oil immersion objective, and a Leica SP8 dive FALCON multiphoton confocal microscope (objective 63x oil – white light laser 488 nm – multiphoton laser 976 nm). Resulting images were acquired by HyD S detectors. For nuclei count, a Molecular DevicesTM ImageXpress Micro (IXM) XL automated microscope (objective 10x dry – laser 358 nm) was used. All microscopy images were analyzed with Image J or MetaXpress® software. Graphs were plotted using GraphPad.

3. Compound synthesis and characterization

A.

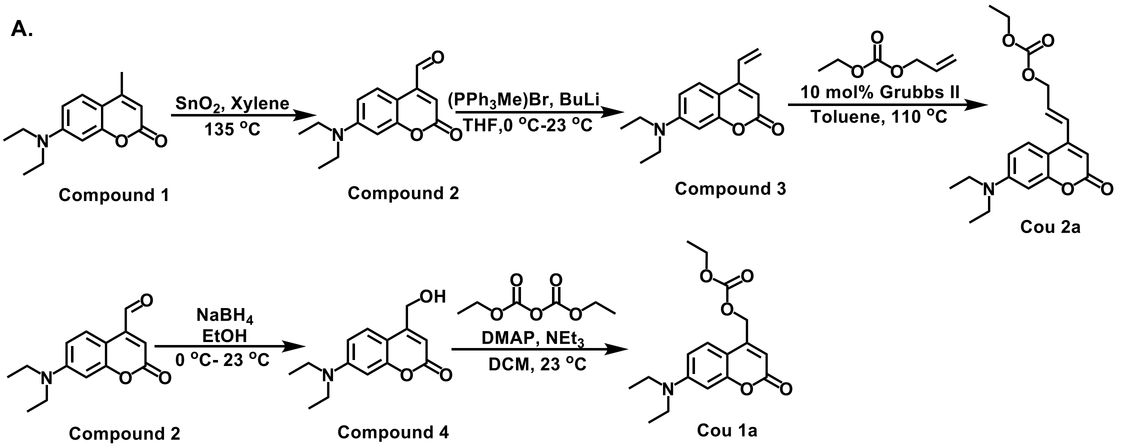

B.

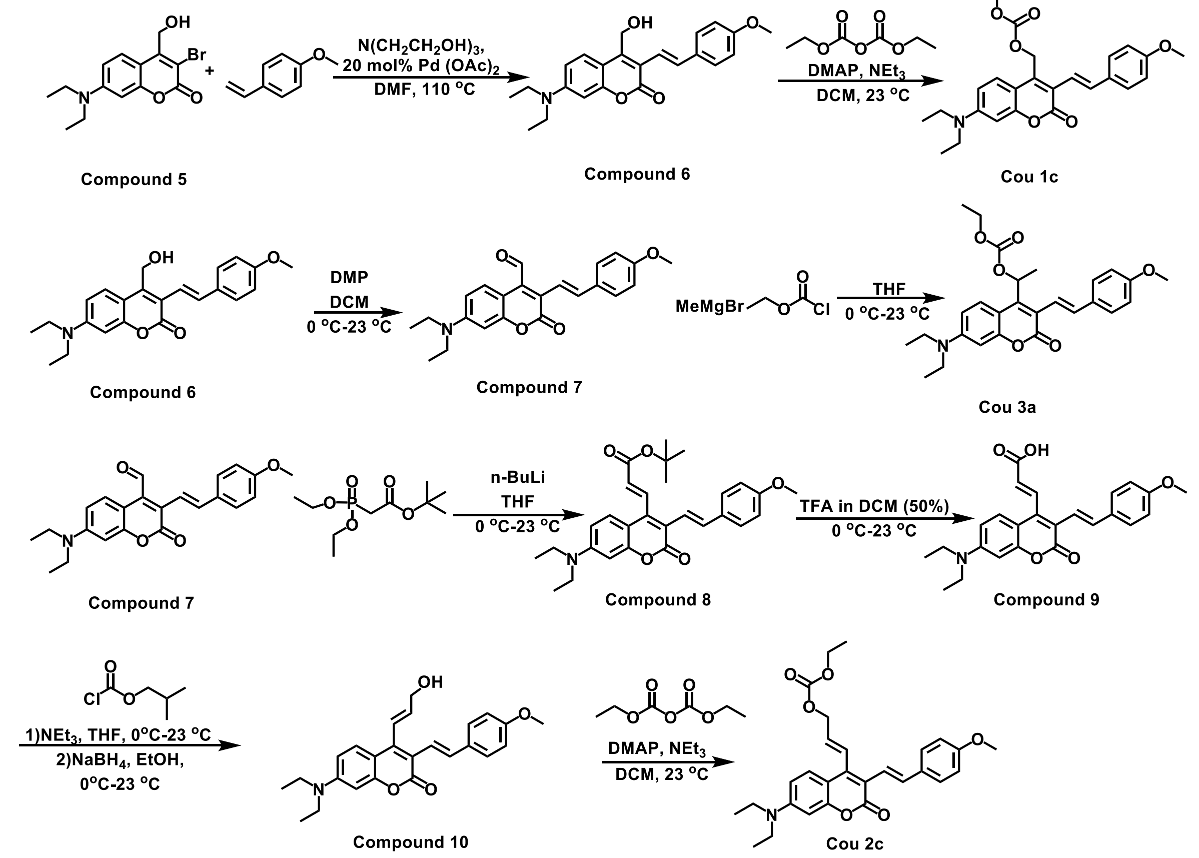

C.

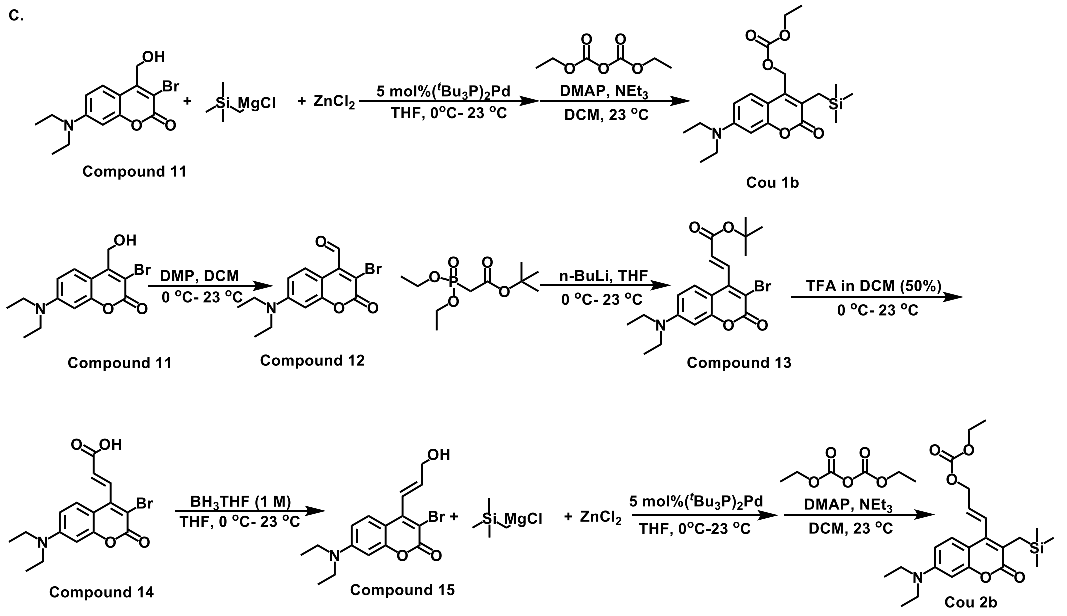
Compound 11
5 mol%(tBu3P)2Pd
THF, 0°C- 23 °C
DMAP, NEt3
DCM, 23 °C
Cou 1b
DMP, DCM
0 °C- 23 °C
Compound 11
Compound 12
n-BuLi, THF
0 °C- 23 °C
Compound 13
TFA in DCM (50%)
0 °C- 23 °C
Compound 14
BH3THF (1 M)
THF, 0 °C- 23 °C
Compound 15
5 mol%(tBu3P)2Pd
THF, 0°C-23 °C
DMAP, NEt3
DCM, 23 °C
Cou 2b

D.

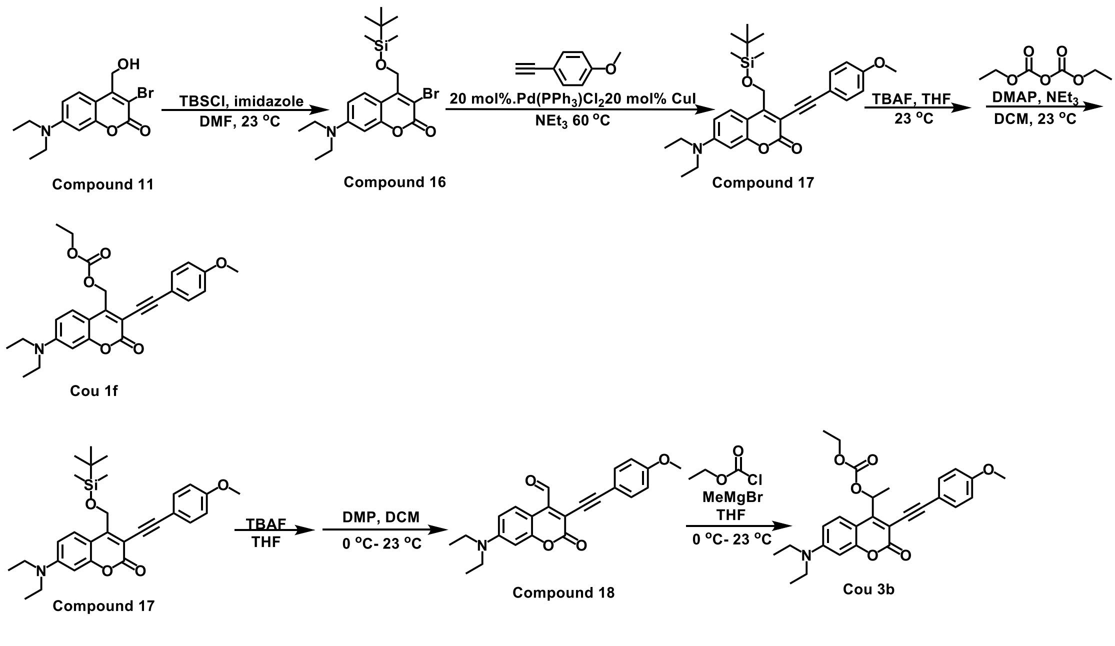
Compound 11
TBSCl, imidazole
DMF, 23 °C
Compound 16
20 mol%.Pd(PPh3)Cl2 20 mol% CuI
NEt3 60 °C
Compound 17
TBAF, THF
23 °C
DMAP, NEt3
DCM, 23 °C
Cou 1f
Compound 17
TBAF
THF
DMP, DCM
0 °C- 23 °C
Compound 18
MeMgBr
THF
0 °C- 23 °C
Cou 3b

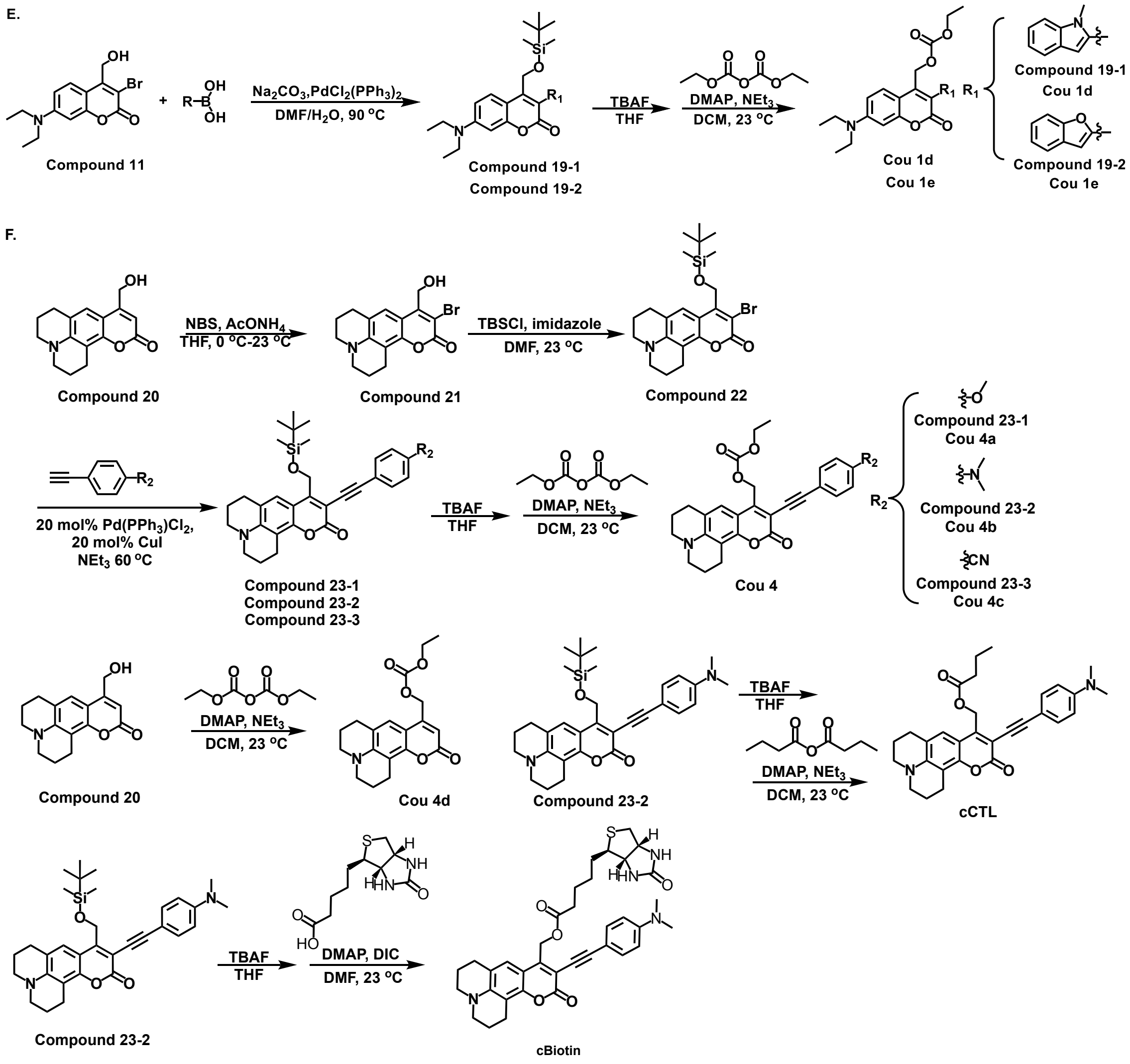

Figure S1. The synthetic routes of Coumarin moieties.

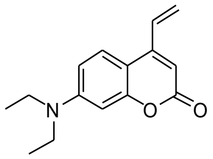

Compound 3: A 25 mL Schlenk tube equipped with a magnetic stirring bar was purged with nitrogen and cooled to 0 °C by an ice bath. Under a nitrogen atmosphere, anhydrous THF (2 mL) and methyltriphenylphosphonium bromide (160 mg, 0.448 mmol, 1.1 equiv.) were added. While stirring, a solution of n-butyllithium in hexane (267.5 μL 1.6 M, 0.428 mmol, 1.05 equiv.) was added dropwise via syringe. The mixture was stirred at 0°C for 20 minutes to allow for ylide formation. Subsequently, a solution of **Compound 2** (92.4mg, 0.4 mmol, 1 equiv.), synthesized from commercially available **Compound 1**, according to a previously reported procedure, [1] in anhydrous THF was added. The reaction mixture was then gradually warmed to 23 °C and stirred for an additional 3 hours. Upon completion monitored by TLC, the reaction was quenched with water, and the organic layer was extracted using brine and ethyl acetate, followed by drying over anhydrous Na_2SO_4. The solvent was removed under reduced pressure, and the crude product was purified via flash chromatography, yielding 62.2 mg of the desired compound (64% yield).

^{1}H NMR (400 MHz, Chloroform-*d*) δ 7.46 (d, J = 9.0 Hz, 1H), 6.94 (dd, J = 17.3, 11.0 Hz, 1H), 6.58 (dd, J = 9.0, 2.6 Hz, 1H), 6.52 (d, J = 2.6 Hz, 1H), 6.13 (s, 1H), 5.94 (dd, J = 17.3, 1.2 Hz, 1H), 5.64 (dd, J = 11.0, 1.2 Hz, 1H), 3.42 (q, J = 7.1 Hz, 4H), 1.21 (t, J = 7.1 Hz, 6H). ^{13}C NMR (126 MHz, Chloroform-*d*) δ 162.71, 156.61, 151.24, 150.76, 130.92, 125.59, 122.43, 108.63, 107.58, 104.80, 98.09, 44.90, 12.61. HRMS (ESI, Pos, m/z): calcd for $C_{15}H_{18}NO_2$ $[M+H]^+$: 244.1338, found: 244.1339.

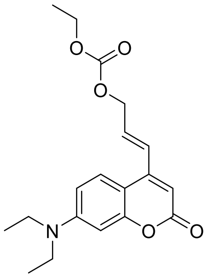

Cou 2a: A 5 mL Schlenk tube equipped with a magnetic stirring bar was charged with a solution of **Compound 3** (13.1mg, 0.054 mmol, 1 equiv.) and allyl ethyl carbonate (0.065 mmol, 1.2 equiv.) in toluene (0.54 mL) under a nitrogen atmosphere. Grubbs Generation II catalyst (4.2mg, 10 mol%, 0.005 mmol) was then added, and the solution was degassed by nitrogen bubbling. The reaction mixture was heated to 110°C and stirred for 1 hour. Upon completion monitored by TLC, the solvent was removed under reduced pressure, and the crude product was purified via flash chromatography, yielding the desired compound as a purified product (8.9 mg, 48%).

^{1}H NMR (400 MHz, Chloroform-*d*) δ 7.46 (d, J = 9.0 Hz, 1H), 6.90 (dq, J = 15.8, 0.9 Hz, 1H), 6.61 (dd, J = 9.0, 2.6 Hz, 1H), 6.54 (d, J = 2.6 Hz, 1H), 6.45 (dt, J = 15.7, 5.5 Hz, 1H), 6.12 (d, J = 0.7 Hz, 1H), 4.85 (dd, J = 5.5, 1.7 Hz, 2H), 4.26 (q, J = 7.1 Hz, 2H), 3.42 (q, J = 7.1 Hz, 4H), 1.35 (t, J = 7.1 Hz, 3H), 1.21 (t, J = 7.1 Hz, 6H). ^{13}C NMR (126 MHz, Chloroform-*d*) δ 150.69, 131.47, 126.67, 125.68, 108.94, 105.18, 98.32, 67.14, 64.58, 45.08, 14.43, 12.55. HRMS (ESI, Pos, m/z): calcd for $C_{19}H_{24}NO_5$ $[M+H]^+$: 346.1654, found: 346.1641.

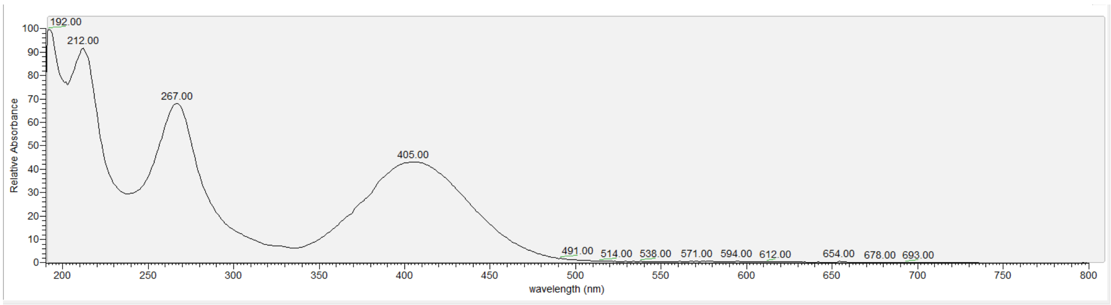

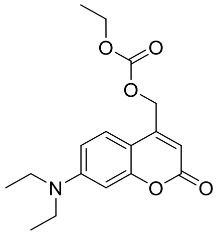

Cou 1a: A 5 mL flask equipped with a magnetic stirring bar was charged with a solution of **Compound 4** (9.88mg, 0.040 mmol,1 equiv.), which was synthesized according to a previously reported procedure [2], in anhydrous DCM (0.4 mL). To this solution, diethyl carbonate (19.8mg, 0.122 mmol, 3.0 equiv.), DMAP (0.5 mg, 0.0040 mmol, 0.1 equiv.), and NEt_3 (0.142 mmol, 3.5 equiv.) were added. The reaction mixture was stirred at 23 °C for 1 hour. Upon completion monitored by TLC, the solvent was removed under reduced pressure, and the crude product was purified by flash chromatography, affording the desired compound in 11.5mg (90%) yield. ^{1}H NMR (400 MHz, Chloroform-*d*) δ 7.29 (d, J = 9.0 Hz, 1H), 6.57 (dd, J = 9.0, 2.6 Hz, 1H), 6.50 (d, J = 2.6 Hz, 1H), 6.18 – 6.10 (m, 1H), 5.25 (d, J = 1.3 Hz, 2H), 4.26 (q, J = 7.1 Hz, 2H), 3.41 (q, J = 7.1 Hz, 4H), 1.34 (t, J = 7.1 Hz, 3H), 1.20 (t, J = 7.1 Hz, 6H). ^{13}C NMR (101 MHz, Chloroform-*d*) δ 161.91, 156.42, 154.80, 150.82, 149.00, 124.50, 108.81, 106.75, 105.98, 97.99, 64.91, 64.57, 44.89, 14.36, 12.55. HRMS (ESI, Pos, m/z): calcd for $C_{17}H_{22}NO_5$ $[M+H]^+$: 320.1498, found: 320.1484.

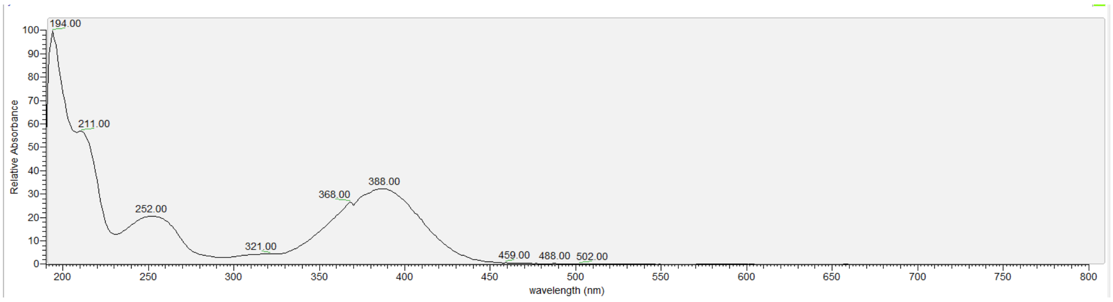

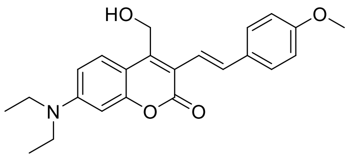

Compound 6: A 25 mL Schlenk tube equipped with a magnetic stirring bar was charged with a solution of **Compound 5** (300.0 mg, 0.92 mmol,1.0 equiv), which was synthesized according to a previously reported procedure[2], in anhydrous DMF (4.6 mL). Under a nitrogen atmosphere, 1-methoxy-4-vinylbenzene (370.0 mg, 2.76 mmol, 3.0 equiv.) and triethanolamine (411.2 mg, 2.76 mmol, 3.0 equiv.) were added. The reaction mixture was subjected to three freeze-pump-thaw cycles to remove dissolved gases. Subsequently, Pd $(OAc)_2$ (42.0 mg, 0.184 mmol, 0.2 equiv.) was added under nitrogen, and the reaction was heated to 110 °C overnight. Upon completion monitored by TLC, the reaction mixture was poured into brine and extracted with ethyl acetate. The organic phase was collected, dried over Na_2SO_4, and concentrated under reduced pressure. The crude product was purified via flash chromatography, affording the desired compound in 51.6% yield (179.92 mg).

^{1}H NMR (500 MHz, Chloroform-*d*) δ 7.66 (d, J = 9.0 Hz, 1H), 7.51 (d, J = 16.2 Hz, 1H), 7.49 – 7.46 (d, J = 8.7 Hz, 2H), 7.08 (d, J = 16.1 Hz, 1H), 6.89 (d, J = 8.7 Hz, 2H), 6.68 (dd, J = 9.1, 2.6 Hz, 1H), 6.56 (d, J = 2.6 Hz, 1H), 4.98 (s, 2H), 3.83 (s, 3H), 3.43 (q, J = 7.1 Hz, 4H), 1.22 (t, J = 7.1 Hz, 6H). ^{13}C NMR (126 MHz, Chloroform- *d*) δ 161.55, 159.65, 154.91, 149.57, 145.31, 134.91, 130.44, 128.12, 126.19, 118.28, 118.15, 114.11, 109.63, 108.94, 98.17, 57.90, 55.35, 45.20, 12.42. HRMS (ESI, Pos, m/z): calcd for $C_{23}H_{26}NO_4$ $[M+H]^+$: 380.1862, found: 380.1855.

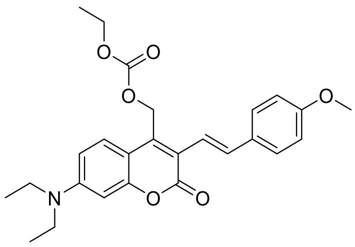

Cou 1c: This compound was synthesized from **Compound 6** (23.9 mg, 0.053 mmol, 1.0 euiv.) following the procedure for **Cou 1a**. The desired product was obtained in 87% yield (20.79 mg). ^{1}H NMR (400 MHz, Chloroform-*d*) δ 7.55 (d, J = 9.1 Hz, 1H), 7.55 (d, J = 16.1 Hz, 1H), 7.49 (d, J = 8.5 Hz, 1H), 7.13 (d, J = 16.1 Hz, 1H), 6.91 (d, J = 8.7 Hz, 1H), 6.64 (dd, J = 9.1, 2.7 Hz, 1H), 6.52 (d, J = 2.6 Hz, 1H), 5.48 (s, 2H), 4.26 (q, J = 7.1 Hz, 2H), 3.84 (s, 2H), 3.43 (q, J = 7.1 Hz, 4H), 1.33 (t, J = 7.1 Hz, 3H), 1.22 (t, J = 7.1 Hz, 6H). ^{13}C NMR (126 MHz, Chloroform-*d*) δ 150.21, 140.55, 135.34, 128.34, 126.15, 118.46, 114.24, 109.35, 97.61, 64.83, 61.94, 55.48, 29.85, 14.40, 12.66. HRMS (ESI, Pos, m/z): calcd for $C_{26}H_{29}NO_6Na[M+Na]^+$: 474.1893, found: 474.1884.

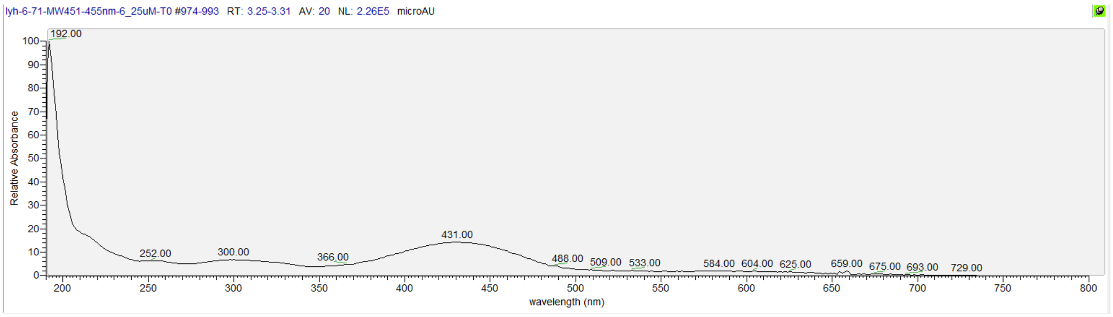

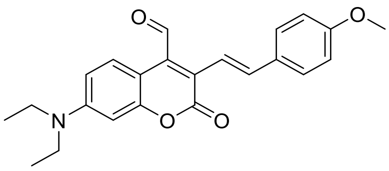

Compound 7: A round-bottom flask equipped with a magnetic stirring bar was charged with **Compound 6** (100.8 mg, 0.266 mmol, 1.0 equiv.) and dissolved in anhydrous DCM. The solution was cooled to 0°C using an ice bath, followed by the addition of Dess–Martin periodinane (123.0 mg, 0.29 mmol, 1.1 equiv.). The reaction mixture was stirred at 0°C for 10 minutes, then gradually warmed to 23 °C and stirred for an additional 2 hours. Upon completion monitored by TLC, the reaction was quenched with saturated $Na_2S_2O_3$ solution and $NaHCO_3$ solution, then extracted with brine and ethyl acetate. The organic phase was collected, dried

over Na_2SO_4, and concentrated under reduced pressure. The crude product was purified by flash chromatography, affording the desired compound in 79% yield (79.56 mg).

^1H NMR (400 MHz, Chloroform-*d*) δ 10.15 (s, 1H), 8.05 (d, J = 9.2 Hz, 1H), 7.49 (d, J = 8.6 Hz, 2H), 7.23 (d, J = 15.9 Hz, 1H), 6.91 (d, J = 8.8 Hz, 2H), 6.78 (d, J = 15.9 Hz, 1H), 6.63 (dd, J = 9.3, 2.7 Hz, 1H), 6.53 (d, J = 2.6 Hz, 1H), 3.85 (s, 3H), 3.43 (q, J = 7.1 Hz, 4H), 1.22 (t, J = 7.1 Hz, 6H). ^{13}C NMR (126 MHz, Chloroform-*d*) δ 192.15, 162.58, 160.62, 155.35, 150.39, 139.88, 129.00, 127.14, 117.62, 114.42, 109.78, 97.79, 55.54, 44.97, 12.65. HRMS (ESI, Pos, m/z): calcd for $C_{23}H_{24}NO_4$ $[M+H]^+$: 378.1706, found: 378.1712.

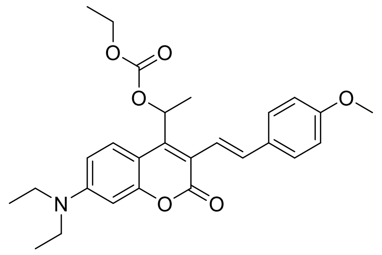

Cou 3a: A 5 mL Schlenk tube equipped with a magnetic stirring bar was charged with a solution of **Compound 7** (9.8mg, 0.026 mmol,1 equiv.) in THF (0.027 mL) under a nitrogen atmosphere. The solution was cooled to 0°C, and MeMgBr (1.4 M, 70.7 μL, 0.099 mmol, 3 equiv.) was added dropwise via syringe while maintaining the temperature at 0°C. The reaction mixture was stirred at 0°C for 10 minutes, then gradually warmed to 23 °C and stirred for an additional 20 minutes. Next, under a nitrogen atmosphere, $ClCO_2Et$ (14.3mg, 0.132 mmol, 5 equiv.) was added to the reaction mixture, which was then stirred for 30 minutes. The reaction was quenched with saturated $NaHCO_3$ solution, followed by extraction with brine and ethyl acetate. The organic phase was collected, dried over Na_2SO_4, and concentrated under reduced pressure. The crude product was purified by flash chromatography, affording the desired compound in 62.1% yield (7.65 mg).

^1H NMR (500 MHz, Chloroform-*d*) δ 7.88 (d, J = 9.2 Hz, 1H), 7.48 (d, J = 8.7 Hz, 2H), 7.44 (d, J = 16.1 Hz, 1H), 7.12 (d, J = 16.1 Hz, 1H), 6.89 (d, J = 8.7 Hz, 2H), 6.64 (dd, J = 9.2, 2.7 Hz, 1H), 6.54 (d, J = 2.7 Hz, 1H), 6.33 (q, J = 6.9 Hz, 1H), 4.23 – 4.06 (m, 2H), 3.83 (s, 3H), 3.42 (q, J = 7.1 Hz, 4H), 1.25 (t, J = 7.1 Hz, 3H), 1.21 (t, J = 7.1 Hz, 6H). ^{13}C NMR (126 MHz, Chloroform-*d*) δ 149.37, 146.73, 135.15, 130.56, 128.11, 127.09, 118.23, 114.06, 109.17, 98.10, 72.08, 64.37, 55.34, 44.98, 20.26, 14.22, 12.48. HRMS (ESI, Pos, m/z): calcd for $C_{27}H_{32}NO_6$ $[M+H]^+$: 466.2230, found: 466.2226.

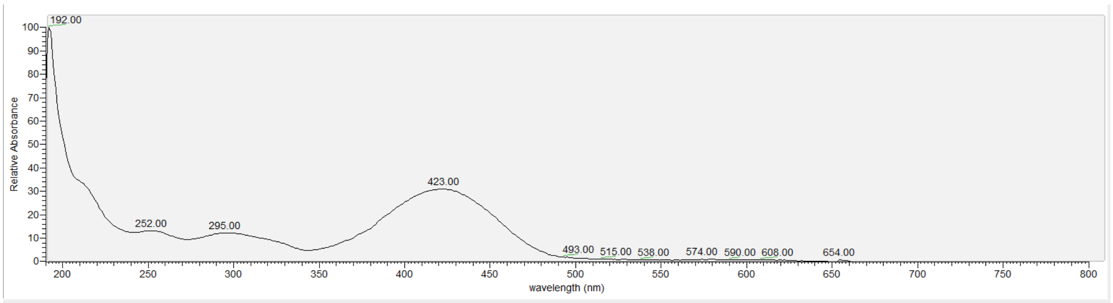

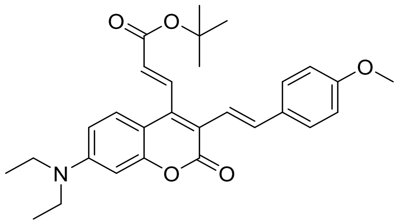

Compound 8: A 10 mL Schlenk tube equipped with a magnetic stirring bar was placed under a nitrogen atmosphere and cooled to 0°C using an ice bath. Anhydrous THF (1.3 mL) and tert-butyl 2-(diethoxyphosphoryl) acetate (34.5mg, 0.137 mmol, 1.08 equiv.) were added under nitrogen. While stirring, n-butyllithium solution in hexane (83.1 μL, 1.6 M, 0.133 mmol, 1.05 equiv.) was added dropwise via syringe. The reaction mixture was stirred at 0°C for 10 minutes under a nitrogen atmosphere, then gradually warmed to 23 °C and stirred for an additional 20 minutes. Next, a solution of **Compound 7** (48 mg, 0.127 mmol, 1.0 equiv.) in anhydrous THF was added under nitrogen, and the reaction was allowed to stir overnight at 23 °C. The reaction was then quenched with water, extracted with brine and ethyl acetate, and the organic phase was dried over Na_2SO_4. The solvent was removed under reduced pressure, and the crude product was purified by flash chromatography, affording the desired compound in 71.8% yield (43.4 mg).

^{1}H NMR (400 MHz, Chloroform-d) δ 7.75 (d, J = 16.4 Hz, 1H), 7.69 (d, J = 16.1 Hz, 1H), 7.44 (m, 3H), 6.96 (d, J = 16.1 Hz, 1H), 6.89 (d, J = 8.8 Hz, 2H), 6.60 (dd, J = 9.1, 2.6 Hz, 1H), 6.54 (d, J = 2.6 Hz, 1H), 6.22 (d, J = 16.3 Hz, 1H), 3.84 (s, 3H), 3.43 (q, J = 7.1 Hz, 5H), 1.58 (s, 9H), 1.23 (t, J = 7.1 Hz, 7H). 13C NMR (126 MHz, Chloroform-d) δ 164.82, 160.75, 159.54, 154.73, 150.13, 143.49, 137.83, 134.14, 130.84, 129.74, 128.03, 127.04, 119.53, 115.66, 114.12, 108.97, 97.56, 55.34, 44.86, 28.21, 12.51. HRMS (ESI, Pos, m/z): calcd for $C_{29}H_{34}NO_5$ $[M+H]^+$: 476.2437, found: 476.2435.

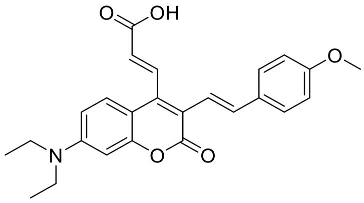

Compound 9: A round-bottom flask equipped with a magnetic stirring bar was charged with **Compound 8** (30.0 mg, 0.063 mmol, 1.0 equiv.) and dissolved in a 1:1 mixture of DCM and TFA. The reaction mixture was stirred at 23 °C for 2 hours. Upon completion monitored by TLC, the solvent was removed under reduced pressure. The crude product was obtained without further purification in 93% yield (24.63 mg).

^{1}H NMR (400 MHz, Chloroform-d) δ 7.96 (d, J = 16.4 Hz, 1H), 7.69 (d, J = 16.1 Hz, 1H), 7.45 (d, J = 8.7 Hz, 2H), 7.44 (d, J = 9.1 Hz, 1H), 6.94 (d, J = 16.1 Hz, 1H), 6.89 (d, J = 8.7 Hz, 2H), 6.70 (dd, J = 9.1, 2.5 Hz, 1H), 6.63 (d, J = 2.5 Hz, 1H), 6.36 (d, J = 16.4 Hz, 1H), 3.82 (s, 3H), 3.45 (q, J = 7.0 Hz, 4H), 1.22 (t, J = 7.2 Hz, 6H). ^{13}C NMR (101 MHz, Chloroform-d) δ 169.59, 160.71, 159.93, 154.66, 149.34, 142.39, 141.42, 135.38, 130.65, 128.34, 127.17, 127.00,

119.18, 116.84, 114.34, 110.30, 108.73, 99.02, 55.48, 45.87, 12.42. HRMS $C_{25}H_{26}NO_5$ $[M+H]^+$: 420.1811, found: 420.1823.

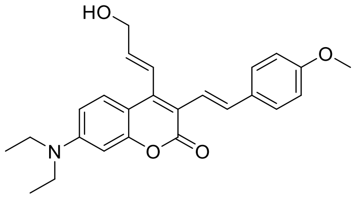

Compound 10: A round-bottom flask equipped with a magnetic stirring bar was charged with **Compound 9**, (14.5 mg, 0.0345mmol, 1.0 equiv.) which was dissolved in anhydrous THF (0.4 mL). NEt_3 (13.9 mg, 0.138 mmol, 4 equiv.) and isobutyl chloroformate (16.1 mg, 0.118 mmol, 1.5 equiv.) were then added at 0°C. The reaction mixture was warm up to 23 °C stirred for 60 minutes, after which the solvent was added EtOH (0.35 mL), and $NaBH_4$ (2.7 mg, 0.071 mmol, 3 equiv.) was added at 0°C and stirred for another 4 hours 23 °C. Upon completion monitored by TLC, the reaction was quenched with water, extracted with brine and ethyl acetate, and the organic phase was dried over Na_2SO_4. The solvent was removed under reduced pressure, and the crude product was purified by flash chromatography, affording the desired compound in 66% yield (9.41 mg).

^{1}H NMR (500 MHz, Chloroform-*d*) δ 7.79 (d, J = 16.1 Hz, 1H), 7.48 (d, J = 9.0 Hz, 1H), 7.45 – 7.40 (m, 2H), 7.02 (d, J = 16.1 Hz, 1H), 6.88 (d, J = 8.8 Hz, 2H), 6.76 (dt, J = 16.3, 1.9 Hz, 1H), 6.57 (dd, J = 9.1, 2.6 Hz, 1H), 6.52 (d, J = 2.6 Hz, 1H), 6.17 (dt, J = 16.3, 4.8 Hz, 1H), 4.50 (dd, J = 4.8, 1.9 Hz, 2H), 3.82 (s, 3H), 3.42 (q, J = 7.1 Hz, 4H), 1.21 (t, J = 7.1 Hz, 6H). ^{13}C NMR (126 MHz, Chloroform-d) δ 161.17, 159.40, 154.90, 150.15, 146.39, 138.68, 132.77, 131.44, 127.95, 127.41, 125.97, 123.58, 120.36, 114.95, 114.22, 108.84, 97.58, 63.25, 55.46, 44.95, 12.66. HRMS (ESI, Pos, m/z): calcd for $C_{25}H_{28}NO_4$ $[M+H]^+$: 406.2018, found: 406.2022.

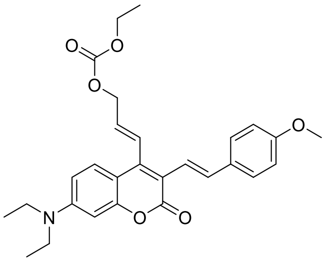

Cou 2c: The compound was synthesized from **Compound 10** (3.0 mg, 0.0074 mmol) following the synthetic procedure for **Cou 1a**, yielding the desired product in 81% yield (2.43 mg).

^{1}H NMR (400 MHz, Chloroform-*d*) δ 7.77 (d, J = 16.1 Hz, 1H), 7.45 (d, J =8.8 Hz, 2H), 7.43 (d, J =9.0 Hz, 1H), 7.00 (d, J = 16.1 Hz, 1H), 6.88 (d, J = 8.8 Hz, 2H), 6.77 (dt, J = 16.3, 1.6 Hz, 1H), 6.58 (dd, J = 9.0, 2.6 Hz, 1H), 6.53 (d, J = 2.5 Hz, 1H),6.09 (dt, J = 16.3, 5.6 Hz, 1H), 4.92 (dd, J = 5.6, 1.6 Hz, 2H), 4.26 (q, J = 7.1 Hz, 2H), 3.82 (s, 3H), 3.42 (q, J = 7.1 Hz, 4H), 1.34 (t, J = 7.1 Hz, 3H), 1.21 (t, J = 7.1 Hz, 6H). ^{13}C NMR (126 MHz, Chloroform-*d*) δ 161.07, 159.47, 155.14, 154.92, 150.22, 145.45, 133.11, 132.59, 131.33, 128.08, 127.49, 127.36, 120.08, 115.19, 114.19, 108.92, 108.59, 97.56, 64.53, 55.46, 44.94, 14.46, 12.66. HRMS (ESI, Pos, m/z): calcd for $C_{28}H_{32}NO_6$ $[M+H]^+$: 478.2230, found: 478.2235.

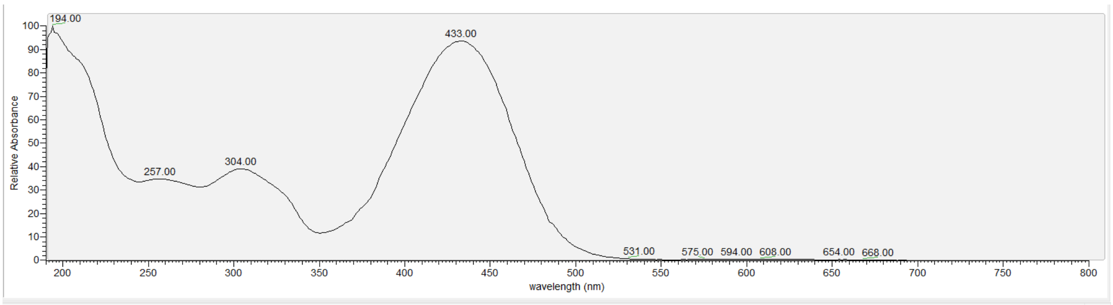

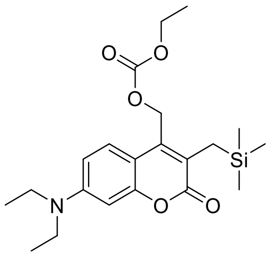

Cou 1b: A 1 M solution of $ZnCl_2$ in THF (2 mL) was then cooled to 0°C. To this solution, $TMSCH_2MgBr$ (1.0 M in Et_2O, 4.0 mL, 4.0 mmol, 2 equiv.) was added dropwise. After 10 minutes, the ice bath was removed, and the mixture was stirred vigorously at 23 °C for 4 hours, until a white precipitate formed. Subsequently, 3 mL of the supernatant from the freshly prepared solution was transferred to a new reaction vessel. $[(^tBu_3P)\ _2Pd]$ (0.15 mmol, 6 mol%) and **Compound 11** (821.5 mg, 2.5 mmol, 1.0 equiv.) were dissolved in 3 mL of THF. The resulting solution was transferred via cannula to the alkyl zinc reagent. The reaction mixture was stirred at 23 °C for 18 hours. After completion, the reaction was quenched with 20 mL of saturated aqueous NH_4Cl solution. The mixture was then extracted with diethyl ether (30 mL × 3), and the combined organic phases were washed with brine. The organic phase was dried over Na_2SO_4, filtered, flushed by a silicon pad filter and concentrated under reduced pressure. The residue was taken 15mg following the synthetic procedure for **Cou 1a**, yielding the desired product in 88.1% yield (16.1mg).

^{1}H NMR (500 MHz, Chloroform-d) δ 7.43 (d, J = 9.1 Hz, 1H), 6.61 – 6.57 (m, 1H), 6.50 (d, J = 2.6 Hz, 1H), 5.23 (s, 2H), 4.22 (q, J = 7.1 Hz, 2H), 3.39 (q, J = 7.1 Hz, 4H), 2.27 (s, 2H), 1.30 (t, J = 7.1 Hz, 5H), 1.19 (t, J = 7.1 Hz, 6H), 0.05 (s, 9H) ^{13}C NMR (126 MHz, Chloroform-d) δ 162.84, 155.14, 154.19, 149.29, 138.20, 125.25, 123.24, 108.91, 108.29, 97.85, 64.71, 62.49, 44.83, 18.05, 14.38, 12.64, -0.98HRMS (ESI, Pos, m/z): calcd for $C_{21}H_{32}NO_5Si$ $[M+H]^+$: 406.2050, found: 406.2090.

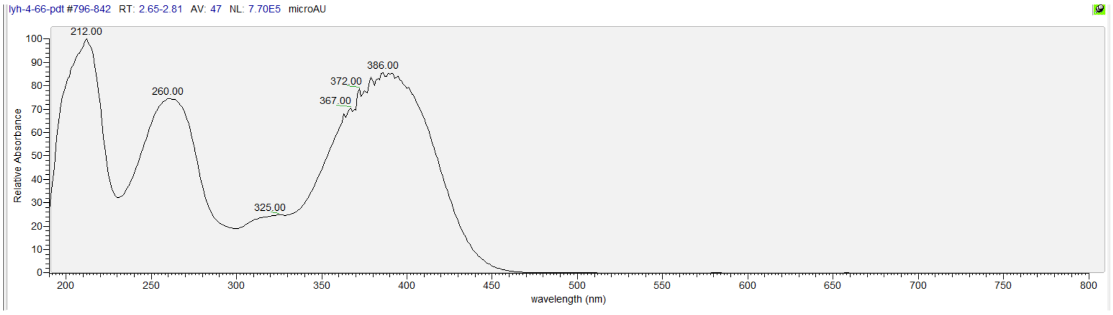

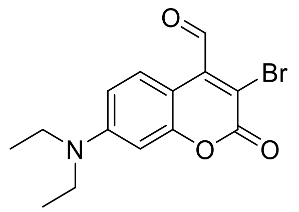

Compound 12: It was synthesized from **Compound 11** (199.2 mg, 0.613 mmol, 1 equiv.) following the synthetic procedure for **Compound 7**, yielding the desired product in 90.6% yield (180.21mg).

^{1}H NMR (500 MHz, Chloroform-d) δ 10.39 (s, 1H), 7.97 (d, J = 9.3 Hz, 1H), 6.62 (dd, J = 9.3, 2.7 Hz, 1H), 6.51 (d, J = 2.6 Hz, 1H), 3.42 (q, J = 7.1 Hz, 4H), 1.22 (t, J = 7.1 Hz, 6H). ^{13}C NMR (126 MHz, Chloroform-d) δ 193.00, 158.40, 155.87, 151.15, 142.10, 110.07, 109.88, 104.79, 97.57, 45.03, 12.56. HRMS (ESI, Pos, m/z): calcd for $C_{14}H_{15}BrNO_3$ [M+H] $^+$: 324.0235, found: 324.0232.

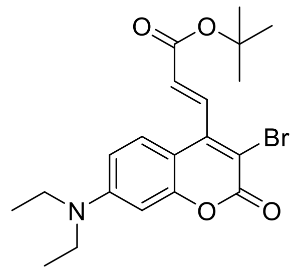

Compound 13: It was synthesized from **Compound 12** (49.3 mg, 0.152 mmol, 1 equiv.) following the synthetic procedure for **Compound 8**, yielding the desired product in 73.0% yield (46.9 mg).

^{1}H NMR (500 MHz, Chloroform-d) δ 7.53 (d, J = 16.3 Hz, 1H), 7.41 (d, J = 9.1 Hz, 1H), 6.59 (dd, J = 9.2, 2.6 Hz, 1H), 6.52 (d, J = 2.5 Hz, 1H), 6.25 (d, J = 16.4 Hz, 1H), 3.42 (q, J = 7.1 Hz, 4H), 1.57 (s, 9H), 1.21 (t, J = 7.1 Hz, 6H). ^{13}C NMR (126 MHz, Chloroform-d) δ 164.58, 158.07, 155.43, 151.03, 148.74, 137.68, 130.41, 127.30, 109.34, 107.35, 103.98, 97.79, 45.03, 28.28, 12.55. HRMS (ESI, Pos, m/z): calcd for $C_{20}H_{24}BrNO_4Na$ [M+Na] $^+$: 444.0786, found: 444.0781.

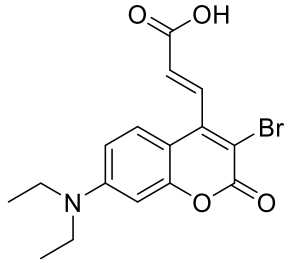

Compound 14: It was synthesized from **Compound 13** (74.0mg, 0.175 mmol, 1equiv.). following the general procedure for **Compound 9**, yielding the desired product in 73.0% yield (46.9 mg)

^1H NMR (400 MHz, Chloroform-d) δ 7.75 (d, J = 16.4 Hz, 1H), 7.45 (d, J = 9.0 Hz, 1H), 6.77 (dd, J = 9.0 Hz, 2.5Hz, 1H), 6.68 (d, J = 2.5 Hz, 1H), 6.39 (d, J = 16.4 Hz, 1H), 3.47 (q, J = 7.1 Hz, 5H), 1.22 (t, J = 7.1 Hz, 6H).^{13}C NMR (101 MHz, Chloroform-d) δ 169.66, 157.95, 155.14, 149.73, 147.98, 141.03, 127.73, 127.23, 111.24, 108.88, 105.34, 99.82, 46.40, 12.2HRMS (ESI, Pos, m/z): calcd for $C_{16}H_{17}BrNO_4$ [M+H]$^+$: 366.0341, found: 366.0318.

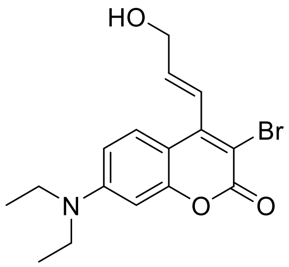

Compound 15: To a Schlenk tube equipped with a magnetic stirring bar and maintained under a nitrogen atmosphere, **Compound 14** (104.0 mg, 0.284 mmol 1.0 equiv.) was dissolved in THF. A 1.0 M BH_3·THF solution (0.57 mL, 0.57 mmol, 2.0 equiv) was added dropwise via syringe at 0°C. The reaction mixture was stirred at 0 °C for 1 h and then allowed to warm to 23 °C, monitoring the progress by TLC. Upon completion, the reaction was quenched with saturated $NaHCO_3$ solution, and the aqueous phase was extracted with EtOAc (3 × 10 mL). The combined organic layers were dried over Na_2SO_4, filtered, and concentrated under reduced pressure to afford the crude product (94 mg, 93.9 % yield) without further purification. ^1H NMR (500 MHz, Chloroform-d) δ 7.49 (d, J = 9.1 Hz, 1H), 6.63 (dt, J = 16.3, 1.9 Hz, 1H), 6.56 (dd, J = 9.2, 2.6 Hz, 1H), 6.50 (d, J = 2.7 Hz, 1H), 6.29 (dt, J = 16.3, 4.6 Hz, 1H), 4.47 (dd, J = 4.6, 1.9 Hz, 2H), 3.41 (q, J = 7.1 Hz, 4H), 1.21 (t, J = 7.1 Hz, 6H). ^{13}C NMR (126 MHz, Chloroform-d) δ 158.53, 155.40, 150.85, 150.64, 139.60, 127.72, 124.42, 109.08, 108.02, 103.36, 97.66, 62.99, 44.96, 12.57. HRMS (ESI, Pos, m/z): calcd for $C_{16}H_{19}BrNO_3$[M+H] $^+$: 352.0548, found: 352.0566.

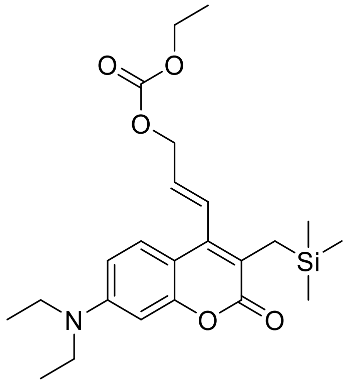

Cou 2b: This compound was synthesized from **Compound 15** (9.8 mg, 0.0278 mmol, 1.0 equiv.) following the synthetic procedure for **Cou 1b**, yielding the desired product in 76.8% yield (9.21mg).

^1H NMR (400 MHz, Chloroform-d) δ 7.40 (d, J = 9.1 Hz, 1H), 6.65 – 6.54 (m, 3H), 6.04 (dt, J = 16.4, 5.7 Hz, 1H), 4.87 (dd, J = 5.7, 1.6 Hz, 2H), 4.27 (q, J = 7.2 Hz, 2H), 3.41 (q, J = 7.1 Hz,

4H), 2.08 (s, 2H), 1.36 (t, J = 7.1 Hz, 3H), 1.20 (t, J = 7.1 Hz, 6H), 0.05 (s, 9H). ^{13}C NMR (101 MHz, $CDCl_3$) δ 162.88, 155.10, 154.48, 149.27, 142.63, 131.46, 128.05, 126.51, 119.41, 108.50, 108.47, 97.92, 77.48, 77.16, 76.84, 67.49, 64.47, 44.82, 18.47, 14.44, 12.63, -0.44. HRMS (ESI, Pos, m/z): calcd for $C_{23}H_{34}NO_5Si$ $[M+H]^+$: 432.2206, found: 432.2241.

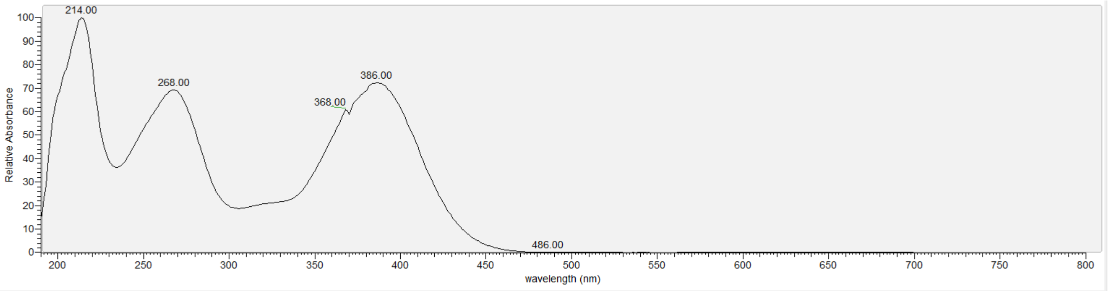

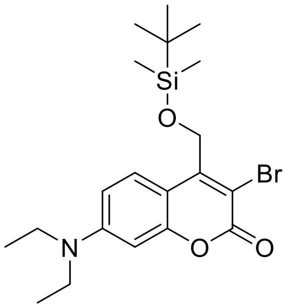

Compound 16: In a round-bottom flask equipped with a stirring bar, **Compound 11** (497.3 mg, 1.53 mmol, 1.0 equiv.) was dissolved in anhydrous DMF (5.1 mL). tert-Butyldimethylsilyl chloride (345.0 mg, 2.30 mmol, 1.5 equiv.) and imidazole (156.4 mg, 2.30 mmol, 1.5 equiv.) were then added, and the reaction mixture was stirred for 3 hours. After completion, the reaction was extracted with brine and ethyl acetate, and the organic layer was dried over anhydrous Na_2SO_4. The solvent was removed under reduced pressure, and the crude product was purified by flash chromatography, affording the desired product in an 83.9% yield (566.5 mg).The proton NMR is the same as reported data[3].

^{1}H NMR (400 MHz, Chloroform-d) δ 7.77 (d, J = 9.2 Hz, 1H), 6.62 (dd, J = 9.2, 2.7 Hz, 1H), 6.49 (d, J = 2.6 Hz, 1H), 5.01 (s, 2H), 3.42 (q, J = 7.1 Hz, 4H), 1.22 (t, J = 7.1 Hz, 6H), 0.91 (s, 9H), 0.14 (s, 6H). HRMS (ESI, Pos, m/z): calcd for $C_{20}H_{30}BrNO_3Si$ $[M+H]^+$: 440.1257, found: 440.1290.

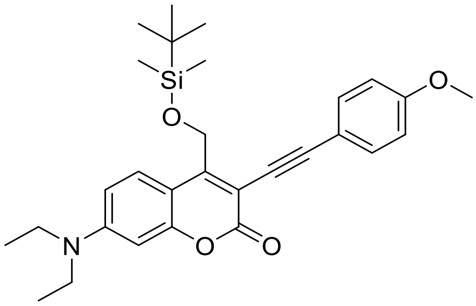

Compound 17: A 10 mL Schlenk tube was equipped with a stirring bar and charged with a solution of **Compound 16** (199.8 mg, 0.454 mmol, 1.0 equiv.) in anhydrous NEt_3 (2.1 mL, 15.89 mmol, 35 equiv.). 1-Ethynyl-4-methoxybenzene (179.5 mg, 1.36 mmol, 3.0 equiv.), Pd (PPh_3)

$_2Cl_2$ (63.8 mg, 0.091 mmol, 20 mol%), and CuI (17.3 mg, 0.091 mmol, 0.2 equiv.) were then added under a nitrogen atmosphere. The reaction mixture was subjected to three freeze-thaw cycles under vacuum to remove dissolved gases. The solution was then heated to 60 °C and stirred overnight. After completion, the reaction mixture was poured into brine and extracted with ethyl acetate. The organic phase was collected, dried over anhydrous Na_2SO_4, and concentrated under reduced pressure. The crude product was purified by flash chromatography, affording the desired product in a 74.1% yield (166.1 mg).

^1H NMR (400 MHz, Chloroform-d) δ 7.81 (d, J = 9.1 Hz, 1H), 7.50 (d, J = 8.9 Hz, 2H), 6.88 (d, J = 8.9 Hz, 2H), 6.61 (dd, J = 9.2, 2.6 Hz, 1H), 6.49 (d, J = 2.6 Hz, 1H), 5.08 (s, 2H), 3.83 (s, 3H), 3.43 (q, J = 7.1 Hz, 4H), 1.24 – 1.20 (t, 6H), 0.90 (s, 9H), 0.13 (s, 6H). ^{13}C NMR (126 MHz, Chloroform-d) δ 161.32, 159.91, 155.55, 153.72, 150.76, 133.24, 128.05, 115.43, 114.13, 109.04, 108.18, 104.01, 97.77, 97.39, 81.97, 61.82, 55.46, 44.95, 29.87, 26.00, 12.68, -4.93. HRMS (ESI, Pos, m/z): calcd for $C_{29}H_{38}NO_4Si$ $[M+H]^+$: 492.2570, found: 492.2562.

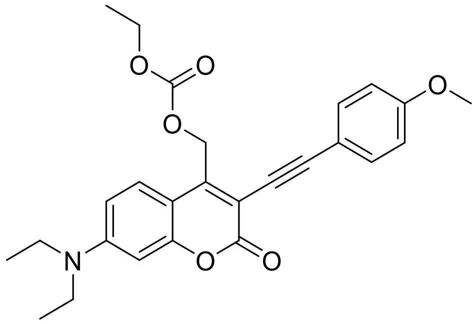

Cou 1f: In a round-bottom flask equipped with a stirring bar, **Compound 17**(159.9 mg, 0.325 mmol, 1.0 equiv) was dissolved in anhydrous THF (3.25 mL). Under an inert atmosphere, a solution of TBAF 1.0 M in THF (358 uL, 0.358 mmol, 1.1 equiv.) in THF was added dropwise via syringe. The reaction mixture was stirred at 23 °C for 30 minutes. Upon completion (monitored by TLC), the reaction was quenched with water, and the product was extracted with brine and ethyl acetate. The organic layer was dried over anhydrous Na_2SO_4, and the solvent was removed under reduced pressure to afford residue without further purification. The subsequent step followed the general procedure for **Cou 1a**, yielding the desired product in 89.1% yield (109.4 mg) for two steps.

^1H NMR (500 MHz, Chloroform-d) δ 7.53 (d, J = 8.8 Hz, 2H), 7.49 (s, 1H), 6.87 (d, J = 8.8 Hz, 2H), 6.63 (dd, J = 9.1, 2.6 Hz, 1H), 6.51 (d, J = 2.6 Hz, 1H), 5.54 (s, 2H), 4.24 (q, J = 7.1 Hz, 2H), 3.83 (s, 3H), 3.43 (q, J = 7.1 Hz, 4H), 1.30 (t, J = 7.1 Hz, 3H), 1.22 (t, J = 7.1 Hz, 6H). ^{13}C NMR (126 MHz, Chloroform-d) δ 160.62, 160.09, 155.45, 154.91, 150.93, 147.45, 133.49, 126.45, 115.13, 114.11, 109.54, 107.59, 106.80, 99.07, 97.78, 81.51, 64.81, 64.12, 55.47, 45.03, 14.39, 12.64. HRMS (ESI, Pos, m/z): calcd for $C_{26}H_{28}NO_6$ $[M+H]^+$: 450.1917, found: 450.1932.

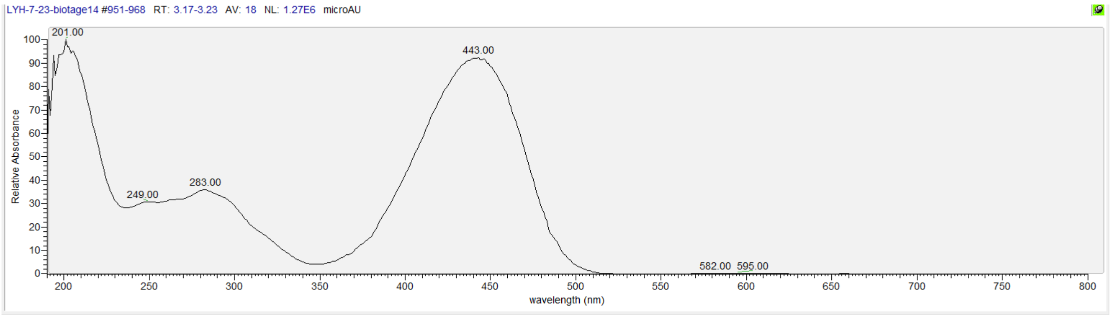

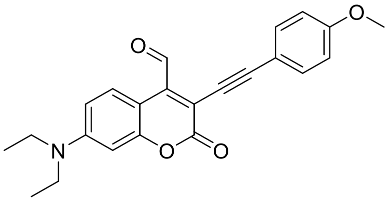

Compound 18: In a round-bottom flask equipped with a stirring bar, **Compound 17** (38.9 mg, 0.079 mmol, 1.0 equiv.) was dissolved in anhydrous THF (3.25 mL). Under an inert atmosphere, a solution of TBAF 1.0 M in THF (87 uL, 0.087 mmol, 1.1 equiv.) in THF was added dropwise via syringe. The reaction mixture was stirred at 23 °C for 30 minutes. Upon completion monitored by TLC, the reaction was quenched with water, and the product was extracted with brine and ethyl acetate. The organic layer was dried over anhydrous Na_2SO_4, and the solvent was removed under reduced pressure to afford residue without further purification. The subsequent transformation followed the general procedure for **Compound 7**, yielding the desired product in 75.1% yield (22.4 mg) for two steps.

^{1}H NMR (500 MHz, Chloroform-d) δ 10.62 (s, 1H), 8.20 (d, J = 9.3 Hz, 1H), 7.43 – 7.37 (m, 2H), 6.80 – 6.71 (m, 2H), 6.51 (dd, J = 9.3, 2.7 Hz, 1H), 6.38 (d, J = 2.6 Hz, 1H), 3.71 (s, 3H), 3.31 (q, J = 7.1 Hz, 4H), 1.10 (t, J = 7.1 Hz, 6H). ^{13}C NMR (126 MHz, Chloroform-d) δ 193.78, 161.04, 160.67, 155.67, 150.96, 141.53, 133.68, 127.52, 114.36, 114.33, 110.19, 104.34, 103.59, 97.71, 80.60, 55.52, 45.02, 12.65. HRMS (ESI, Pos, m/z): calcd for C23H22NO4 [M+H] $^+$: 376.1549, found: 376.1567.

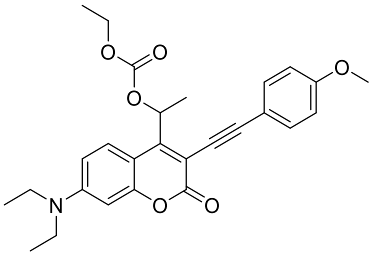

Cou 3b: This compound was synthesized from **Compound 18** (27.1 mg, 0.0719 mmol, 1.0 equiv.) following the synthetic procedure for **Cou 3a**, yielding the desired product in 68.2% yield (22.74 mg).

^{1}H NMR (500 MHz, Chloroform-d) δ 7.91 (d, J = 9.2 Hz, 1H), 7.53 (d, J = 8.8 Hz, 1H), 6.87 (d, J = 8.8 Hz, 1H), 6.61 (dd, J = 9.3, 2.6 Hz, 1H), 6.51 (d, J = 2.6 Hz, 1H), 6.42 (d, J = 6.9 Hz, 1H), 4.18 (dd, J = 14.0, 7.1 Hz, 1H), 3.83 (s, 2H), 3.42 (q, J = 7.1 Hz, 4H), 1.76 (d, J = 7.0 Hz, 3H), 1.27 (t, J = 7.2 Hz, 4H), 1.22 (t, J = 7.1 Hz, 6H). ^{13}C NMR (126 MHz, Chloroform-d) δ 160.70,

160.01, 155.81, 154.22, 153.90, 150.56, 133.43 (d, J = 2.4 Hz), 127.24, 115.37, 114.09 (d, J = 2.4 Hz), 109.21, 105.76, 103.58, 99.70, 98.04 (d, J = 2.4 Hz), 81.39, 74.58, 55.46, 44.94, 19.89, 14.39, 12.66. HRMS (ESI, Pos, m/z): calcd for $C_{27}H_{29}NO_6Na$ [M+Na] +: 486.1893, found: 486.1871.

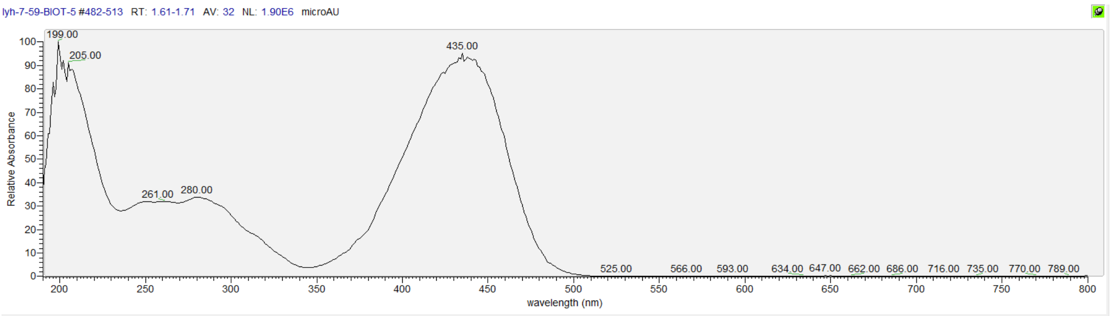

General Procedure A

This procedure was used for Compound19-1 and Compound19-2.

A 10 mL Schlenk tube was equipped with a stirring bar and charged with a solution of **Compound 11** (1.0 equiv.) in a DMF/H_2O mixture (8:2). Na_2CO_3, (3.5 equiv.) boronic acid derivatives (3.0 equiv.), and Pd $(PPh_3)_2Cl_2$ (10 mol%) were then added under a nitrogen atmosphere. The reaction mixture was subjected to three freeze-thaw cycles under vacuum to remove dissolved gases. The solution was then heated to 90°C and stirred overnight. After completion monitored by TLC, the reaction mixture was poured into brine and extracted with ethyl acetate. The organic phase was collected, dried over anhydrous Na_2SO_4, and concentrated under reduced pressure. The crude product was purified by flash chromatography, affording the desired product.

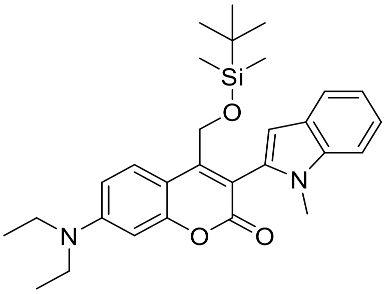

Compound 19-1: This compound was synthesized from **Compound 11** (23.4 mg, 0.0719 mmol 1.0 equiv.) following the general procedure A yielding the desired product in 62.1% yield (41.5 mg).

^{1}H NMR (500 MHz, Chloroform-d) δ 7.80 (d, J = 9.1 Hz, 1H), 7.63 (dt, J = 7.9, 1.0 Hz, 1H), 7.41 – 7.35 (m, 1H), 7.27 – 7.21 (m, 1H), 7.17 – 7.10 (m, 1H), 6.68 (dd, J = 9.1, 2.6 Hz, 1H), 6.58 (d, J = 2.6 Hz, 1H), 6.52 (d, J = 0.8 Hz, 1H), 4.84 – 4.57 (m, 2H), 3.65 (s, 3H), 3.48 (q, J = 7.1 Hz, 4H), 1.27 (t, J = 7.1 Hz, 6H), 0.86 (s, 9H), -0.03 (d, J = 25.5 Hz, 6H). ^{13}C NMR (126 MHz, Chloroform-d) δ 161.78, 156.53, 152.77, 150.79, 137.93, 132.71, 128.15, 127.79, 121.81, 120.78, 119.54, 112.13, 109.56, 109.01, 108.16, 104.47, 97.50, 60.28, 44.99, 30.80, 29.86, 25.89, 12.64, -5.33 (d, J = 15.5 Hz). HRMS (ESI, Pos, m/z): calcd for: $C_{29}H_{39}N_2O_3Si$ $[M+H]^+$: 491.2730, found: 491.2912.

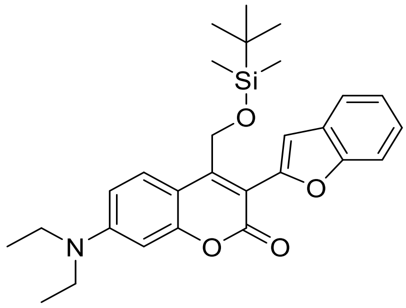

Compound 19-2: This compound was synthesized from **Compound 11** (44.336 mg, 0.136 mmol, 1.0 equiv.) following the general procedure A yielding the desired product in 85.1% yield (55.4 mg).
^{1}H NMR (400 MHz, Chloroform-d) δ 7.82 (d, J = 9.2 Hz, 1H), 7.61 (ddd, J = 7.5, 1.5, 0.7 Hz, 1H), 7.50 (dt, J = 8.2, 0.9 Hz, 1H), 7.31 – 7.27 (m, 1H), 7.23 (dd, J = 7.3, 1.1 Hz, 1H), 7.18 (d, J = 1.0 Hz, 1H), 6.64 (dd, J = 9.2, 2.6 Hz, 1H), 6.52 (d, J = 2.6 Hz, 1H), 4.96 (s, 2H), 3.45 (q, J = 7.1 Hz, 4H), 1.24 (t, J = 7.1 Hz, 6H), 0.87 (s, 9H), 0.05 (s, 6H). ^{13}C NMR (101 MHz, Chloroform-d) δ 161.04, 156.09, 154.80, 151.28, 150.93, 149.95, 128.79, 128.38, 124.54, 122.95, 121.38, 111.21, 110.13, 109.59, 109.05, 108.41, 97.22, 60.26, 44.93, 25.94, 18.40, 12.66, -5.15. HRMS (ESI, Pos, m/z): calcd for: $C_{28}H_{36}NO_4Si$ [M+H]+: 478.2414, found: 478.2459

General Procedure B

This procedure was used for Cou 1d, Cou 1e, Cou 4a, Cou 4b, Cou 4c

To a round bottom flask with a stirring bar, Coumarin derivatives (**Compound 23-1** or **Compound 23-2**, **Compound 23-3**, **Compound 19-1** and **Compound 19-2**) (1.0 equiv.) were dissolved in anhydrous THF, the solution of 1.0 M TBAF in THF (1.1 equiv.) was added via syringe. To stir it for 30 mins at 23 °C and extract it by brine and ethyl acetate and dry it over by Na_2SO_4. Removing the solvent and no further purification. Then dissolve the solid mixture is DCM, NEt_3(3.3 equiv.), Diethyl dicarbonates (3.0 equiv.) and DMAP (0.1eq.) were added then stirred until completion (monitored by TLC). To extract it by brine and ethyl acetate and dry it over by Na_2SO_4. Removing the solvent and purification by fast chromatography.

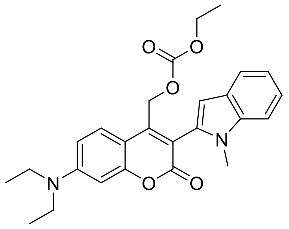

Cou 1d: This compound was synthesized from **Compound 19-1** (71.2 mg, 0.145 mmol, 1.0 equiv.) following the general procedure B yielding the desired product in 85.1% yield (55.4 mg).
^{1}H NMR (400 MHz, Chloroform-d) δ 7.64 – 7.58 (m, 2H), 7.36 (d, 1H), 7.27-7.22 (m, 1H), 7.12 (ddd, J = 7.9, 7.0, 1.0 Hz, 1H), 6.73 (dd, J = 9.1, 2.6 Hz, 1H), 6.63 (d, J = 2.5 Hz, 1H), 6.50 (d, J = 0.8 Hz, 1H), 5.37 (d, J = 12.0 Hz, 1H), 5.09 (d, J = 11.9 Hz, 1H), 4.14 (m, 2H), 3.66 (s, 3H), 3.47 (q, J = 7.1 Hz, 4H), 1.25 (m, 9H). ^{13}C NMR (126 MHz, Chloroform-d) δ 150.45, 147.42, 127.68, 127.14, 122.22, 120.94, 119.73, 110.22, 109.75, 104.92, 98.54, 64.77, 63.49, 45.60,

30.88, 14.32, 12.48. HRMS (ESI, Pos, m/z): calcd for $C_{26}H_{28}N_2O_5Na$ $[M+Na]^+$: 471.1896, found: 471.1856.

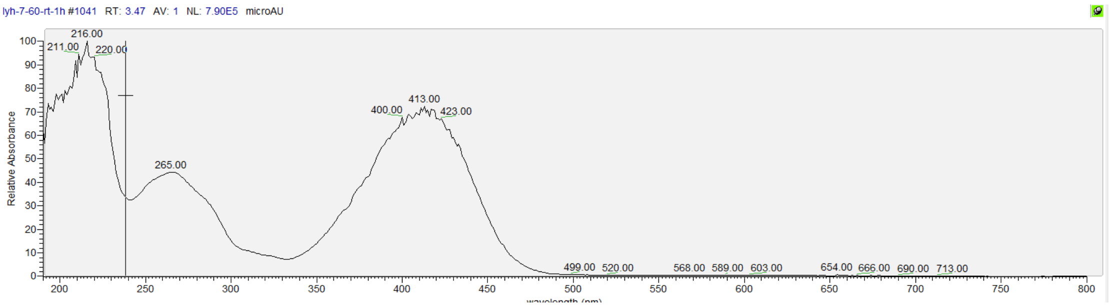

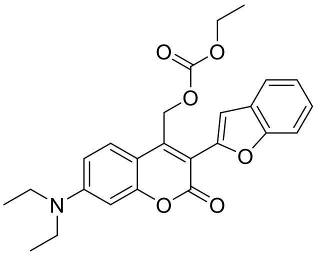

Cou 1e: This compound was synthesized from **Compound 19-2** (29.0 mg, 0.061 mmol, 1.0 equiv.) following the general procedure B yielding the desired product in 91.2% yield (24.1mg). ^1H NMR (500 MHz, Chloroform-d) δ 7.62 (ddd, J = 7.6, 1.4, 0.7 Hz, 1H), 7.59 (d, J = 9.1 Hz, 1H), 7.48 (dd, J = 8.2, 1.0 Hz, 1H), 7.37 (d, J = 1.0 Hz, 1H), 7.32 – 7.20 (m, 2H), 6.66 (dd, J = 9.2, 2.6 Hz, 1H), 6.54 (d, J = 2.6 Hz, 1H), 5.57 (s, 2H), 4.21 (q, J = 7.2 Hz, 2H), 3.45 (q, J = 7.1 Hz, 4H), 1.25 (m, 9H). ^{13}C NMR (126 MHz, Chloroform-d) δ 150.98, 144.18, 126.77, 124.73, 122.98, 121.50, 111.12, 110.15, 109.49, 107.84, 97.40, 64.59, 63.03, 44.89, 14.23, 12.51. HRMS (ESI, Pos, m/z): calcd for $C_{25}H_{24}NO_6Na$ $[M+Na]^+$: 458.1580, found: 458.1563.

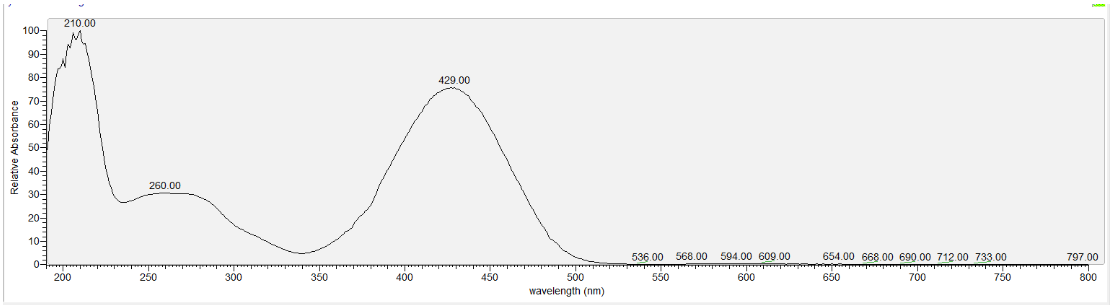

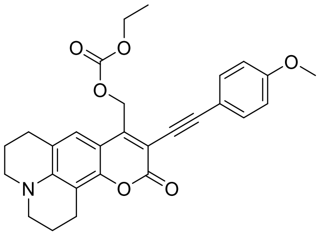

Cou 4a: This compound was synthesized from **Compound 23-1** (38.0 mg, 0.0737 mmol, 1.0 equiv.) following General Procedure B, affording the target product in a 94.0% yield (33.27 mg). ^1H NMR (500 MHz, Chloroform-d) δ 7.53 (d, J = 9.0 Hz, 2H), 7.07 (s, 1H), 6.87 (d, J = 8.9 Hz, 2H), 5.51 (s, 2H), 4.23 (q, J = 7.1 Hz, 2H), 3.83 (s, 3H), 3.32 – 3.24 (m, 4H), 2.89 (t, J = 6.5 Hz, 2H), 2.78 (t, J = 6.3 Hz, 2H), 2.00 – 1.94 (m, 4H), 1.30 (t, J = 7.1 Hz, 3H). ^{13}C NMR (126 MHz,

Chloroform-d) δ 159.97, 147.50, 146.45, 133.45, 122.37, 119.01, 114.07, 107.50, 98.65, 64.73, 64.09, 55.46, 49.76, 21.56, 20.64, 20.52, 14.40. HRMS (ESI, Pos, m/z): calcd for C28H28NO6 [M+Na]+: 474.1917, found: 474.1919

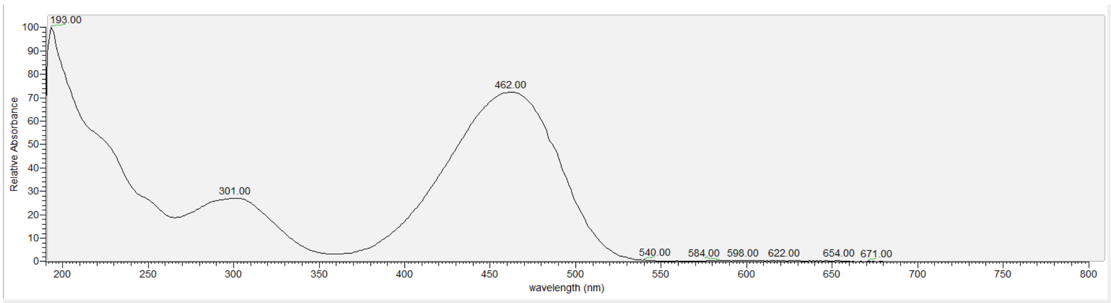

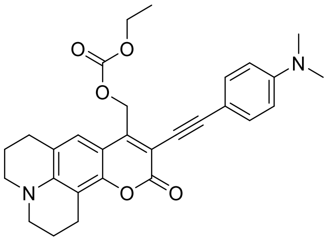

Cou 4b: This compound was synthesized from **Compound 23-2** (15.8 mg, 0.0299 mmol, 1.0 equiv.) following General Procedure B, affording the target product in an 89.3% yield (13.0 mg). ^{1}H NMR (500 MHz, Chloroform-d) δ 7.46 (d, J = 9.0 Hz, 2H), 7.06 (s, 1H), 6.65 (d, J = 8.9 Hz, 2H), 5.52 (s, 2H), 4.24 (q, J = 7.2 Hz, 2H), 3.27 (m,4), 2.99 (s, 6H), 2.89 (t, J = 6.4 Hz, 2H), 2.78 (t, J = 6.1 Hz, 2H), 2.01 – 1.87 (m, 4H), 1.30 (t, J = 7.1 Hz, 3H).^{13}C NMR (126 MHz, Chloroform-d) δ 146.42, 146.14, 133.14, 122.21, 118.91, 111.84, 106.89, 100.25, 81.36, 64.67, 64.22, 50.17, 49.74, 40.35, 27.94, 21.61, 20.70, 20.55, 14.42.

HRMS (ESI, Pos, m/z): calcd for $C_{29}H_{31}N_2O_5$ [M+H] $^+$: 487.2233, found: 487.2228

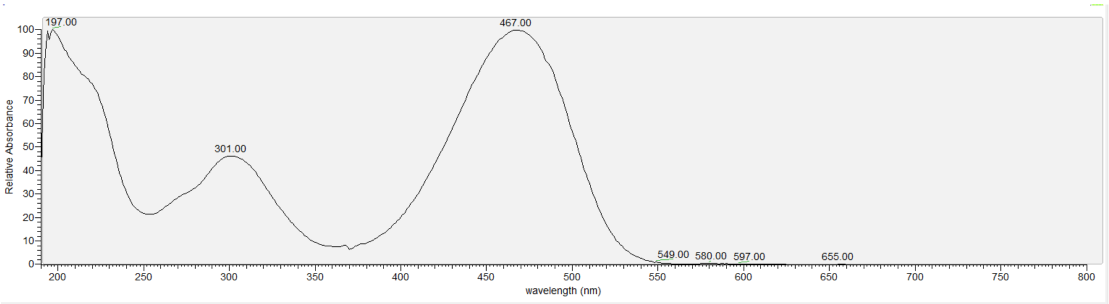

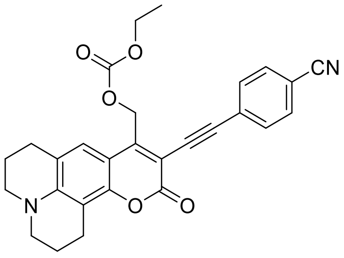

Cou 4c: This compound was synthesized from **Compound 23-3** (15 mg, 0.0294 mmol, 1.0 equiv.) following General Procedure B, affording the target product in an 88.7% yield (12.2 mg). ^{1}H NMR (500 MHz, Chloroform-d) δ 7.37 (s, 1H), 4.97 (s, 2H), 3.33 – 3.21 (m, 4H), 2.87 (t, J = 6.5 Hz, 2H), 2.81 – 2.74 (m, 2H), 1.97 (tdd, J = 8.7, 5.9, 3.2 Hz, 4H), 0.89 (s, 9H), 0.12 (s, 6H).

^{13}C NMR (126 MHz, Chloroform-d) δ 160.59, 154.91, 149.35, 147.19, 132.23, 132.11, 128.21, 122.71, 119.33, 118.79, 111.55, 107.33, 103.88, 96.48, 87.94, 64.87, 63.75, 50.25, 49.81, 29.85, 27.92, 21.43, 20.50, 20.43, 14.39. HRMS (ESI, Pos, m/z): calcd for $C_{28}H_{25}N_2O_5$ [M+H] $^+$: 469.1764, found: 469.1753.

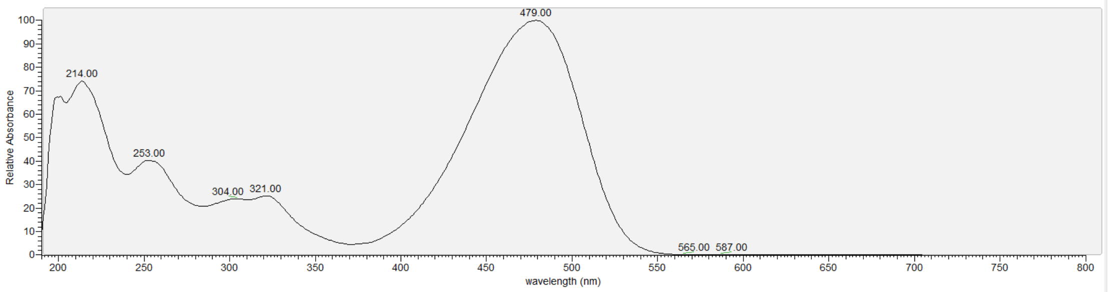

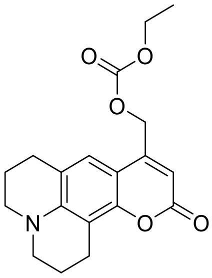

Cou 4d

This compound was synthesized from **Compound 20** (20 mg, 0.074 mmol, 1.0 equiv.) following General Procedure B without TBAF deprotection affording the target product in a 79.0% yield (20.6 mg). 1H NMR (500 MHz, Chloroform-d) δ 6.88 (s, 1H), 6.13 (s, 1H), 5.24 (d, J = 1.3 Hz, 2H), 4.26 (q, J = 7.1 Hz, 2H), 3.26 (dt, J = 9.0, 5.7 Hz, 4H), 2.89 (t, J = 6.5 Hz, 2H), 2.79 – 2.71 (m, 2H), 2.01 – 1.93 (m, 4H), 1.35 (t, J = 7.1 Hz, 3H). ^{13}C NMR (101 MHz, CDCl3) δ 162.26, 154.85, 151.39, 149.09, 146.12, 120.53, 118.40, 107.19, 105.97, 105.83, 77.48, 77.36, 77.16, 76.84, 64.87, 64.64, 50.06, 49.66, 27.86, 21.61, 20.72, 20.57, 14.38. HRMS (ESI, Pos, m/z): calcd for $C_{19}H_{22}NO_5$ [M+H] $^+$: 344.1498, found: 344.1483.

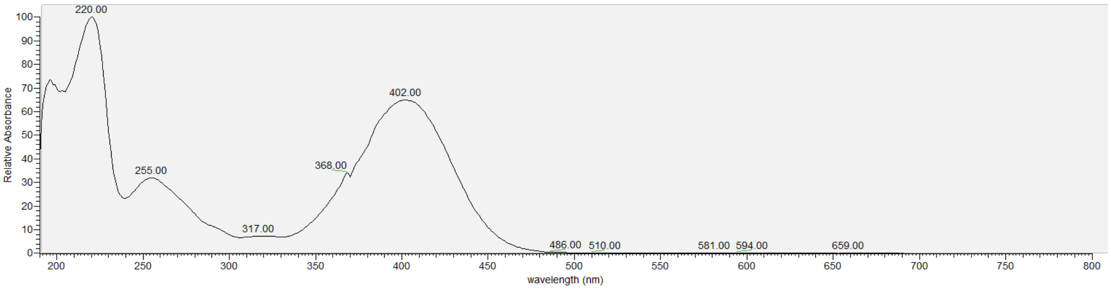

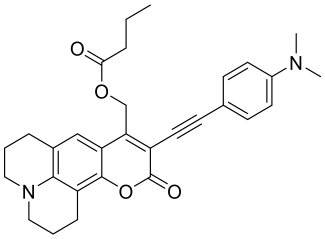

cCTL: This compound was synthesized from **Compound 23-2** (21.5 mg, 0.04 mmol, 1.0 equiv.) following General Procedure B with the Butyric anhydride, affording the target product in a 75.2% yield (13.2 mg). 1H NMR (500 MHz, Chloroform-d) δ 7.44 (d, J = 8.8 Hz, 2H), 6.98

(s, 1H), 6.64 (d, J = 8.4 Hz, 2H), 5.44 (s, 2H), 3.28 (dt, J = 8.8, 5.6 Hz, 4H), 2.99 (s, 6H), 2.90 (t, J = 6.5 Hz, 4H), 2.76 (t, J = 6.2 Hz, 4H), 2.34 (t, J = 7.4 Hz, 2H), 2.03 – 1.83 (m, 4H), 1.66 (q, J = 7.4 Hz, 2H), 0.93 (t, J = 7.4 Hz, 3H). 13C NMR (126 MHz, CDCl3) δ 173.49, 160.98, 150.34, 150.29, 147.51, 146.11, 133.09, 122.10, 118.81, 111.87, 109.95, 107.76, 106.93, 106.66, 100.08, 81.43, 77.41, 77.16, 76.91, 60.85, 50.15, 40.36, 36.26, 27.91, 21.61, 20.68, 20.57, 18.65, 13.82. HRMS (ESI, Pos, m/z): calcd for $C_{30}H_{33}N_2O_4$ [M+H] $^+$: 485.2440, found: 485.2437.

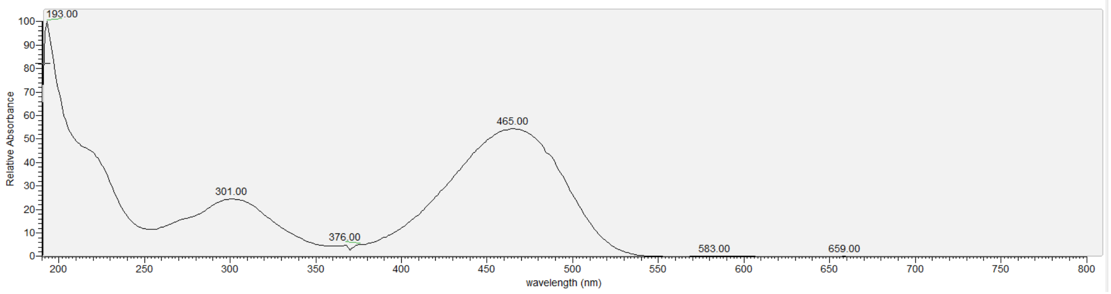

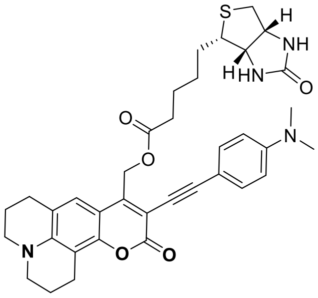

cBiotin: This compound was synthesized from **Compound 23-2** (21.5 mg, 0.04 mmol, 1.0 equiv.) following General Procedure B TBAF deprotection and mix with biotin(9.37 mg, 0.04 mmol, 1.1 equiv.) DIC (6.85 mg, 0.54 mmol, 1.5 equiv.) and DMAP (6.63 mg, 0.54 mmol, 1.5 equiv.) in DMF (0.05M) at room temperature, purified by fast chromatography, affording the target product in a 47.8% yield (11.1 mg). ^{1}H NMR (500 MHz, Chloroform-d) δ 7.43 (d, J = 8.9 Hz, 2H), 7.00 (s, 1H), 6.65 (d, J = 9.0 Hz, 2H), 5.51 – 5.28 (m, 2H), 4.74 (s, 1H), 4.55 (s, 1H), 4.43 (dd, J = 7.8, 5.0 Hz, 1H), 4.18 (ddd, J = 7.9, 4.6, 1.6 Hz, 1H), 3.34 – 3.24 (m, 4H), 3.01 (d, J = 14.9 Hz, 7H), 2.92 – 2.88 (m, 2H), 2.85 (dd, J = 12.8, 5.0 Hz, 1H), 2.78 (t, J = 6.3 Hz, 2H), 2.66 (d, J = 12.8 Hz, 1H), 2.38 (t, J = 7.3 Hz, 2H), 1.97 (ddt, J = 8.8, 6.4, 3.1 Hz, 4H), 1.66 (p, J = 7.5 Hz, 2H), 1.47 – 1.34 (m, 2H). ^{13}C NMR (126 MHz, Chloroform-d) δ 173.26, 162.75, 160.98, 150.38, 147.42, 146.23, 133.09, 121.99, 118.93, 111.87, 107.74, 106.96, 100.15, 81.62, 61.87, 60.76, 60.14, 55.13, 50.16, 49.74, 40.65, 40.35, 33.97, 28.26, 27.96, 24.98, 21.59, 20.65, 20.57.HRMS (ESI, Pos, m/z): calcd for $C_{36}H_{41}N_4O_5S$ [M+H] $^+$: 641.2798, found: 641.2772.

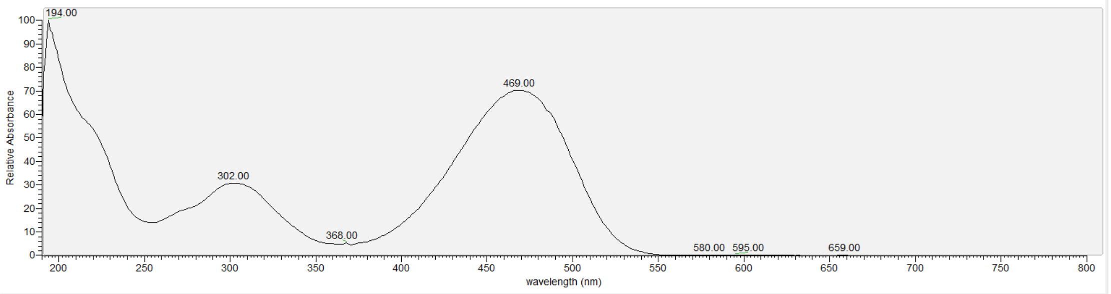

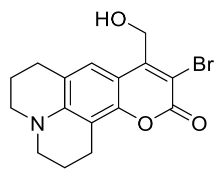

Compound 21: To a round-bottom flask equipped with a stirring bar, **Compound 20** (531.2 mg, 1.96 mmol, 1.0 equiv.), synthesized according to a previously reported procedure, [3] was dissolved in anhydrous THF. The reaction mixture was cooled to 0°C using an ice bath, followed by the addition of NBS (384.5 mg, 2.16 mmol, 1.1 equiv.) and $AcONH_4$ (51.2 mg, 0.196 mmol, 0.1 equiv.). After stirring at 0°C for 10 minutes, the mixture was warmed to 23 °C and stirred for an additional 2 hours. The reaction was quenched with brine and extracted with ethyl acetate. The organic layer was collected, dried over Na_2SO_4, and concentrated. Purification by flash chromatography afforded the desired product in 42.8% yield (287.8 mg).
^{1}H NMR (400 MHz, Chloroform-d) δ 7.23 (s, 1H), 4.93 (s, 2H), 3.38 – 3.17 (m, 4H), 2.87 (t, J = 6.5 Hz, 2H), 2.83 – 2.76 ((t, J = 6.5 Hz, 2H), 2.04 – 1.88 (m, 4H) ^{13}C NMR (101 MHz, Chloroform-d) δ 158.68, 150.92, 150.49, 146.35, 122.45, 119.13, 107.50, 106.77, 104.91, 61.64, 50.11, 49.65, 27.92, 21.55, 20.60, 20.54. HRMS (ESI, Pos, m/z): calcd for $C_{16}H_{17}BrNO_3$ [M+H] $^+$: 350.0392, found: 350.0403.

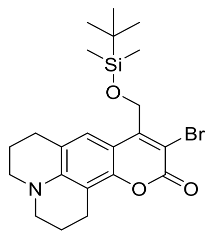

Compound 22: This compound was synthesized from **Compound 21** (1.130 mmol) following the synthetic procedure as for **Compound 16**, affording the target product in an 87.7% yield (458.4 mg).

General Procedure C

This procedure is suitable for Cou 4a-1, Cou 4b-1 and Cou 4c-1.

A 10 mL Schlenk tube was equipped with a stirring bar and charged with a solution of Coumarin derivatives (1 equiv.) in anhydrous NEt_3 (35 equiv.). Under a nitrogen atmosphere, phenylethyne derivatives (3.0 equiv.), Pd $(PPh_3)_2Cl_2$ (20mol%), and CuI (20mol%.) were added. The reaction mixture underwent three freeze-thaw cycles under vacuum to remove dissolved gases, then was heated to 60°C and stirred overnight. Upon completion monitored by TLC, the reaction mixture was poured into brine and extracted with ethyl acetate. The organic phase was collected, dried over anhydrous Na_2SO_4, and concentrated under reduced pressure. The crude product was purified by flash chromatography, affording the desired product.

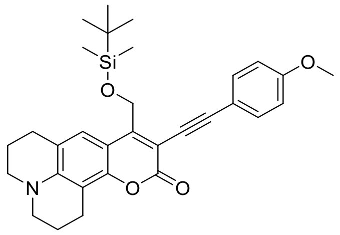

Compound 23-1: This compound was synthesized from **Compound 22** (3.44 mg, 0.086 mmol, 1.0 equiv.) following General Procedure C, affording the target product in an 86.6% yield (38.5 mg).

^{1}H NMR (400 MHz, Chloroform-d) δ 7.49 (d, J = 8.9 Hz, 1H), 7.44 (s, 1H), 6.87 (d, J = 8.8 Hz, 1H), 5.06 (s, 2H), 3.83 (s, 2H), 3.32 – 3.19 (m, 3H), 2.89 (t, J = 6.5 Hz, 2H), 2.77 (t, J = 6.3 Hz, 2H), 1.98 (t, J = 6.0 Hz, 3H), 0.89 (s, 6H), 0.12 (s, 4H). ^{13}C NMR (126 MHz, Chloroform-d) δ 153.84, 146.16, 133.22, 124.22, 118.42, 114.08, 97.42, 82.36, 61.84, 55.45, 50.18, 49.74, 27.88, 25.97, 21.73, 20.76, 20.56, -4.92. HRMS (ESI, Pos, m/z): calcd for $C_{31}H_{38}NO_4Si$ [M+H]$^+$: 516.2570, found: 516.2614.

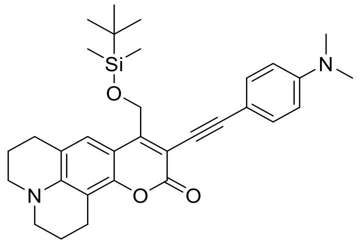

Compound 23-2: This compound was synthesized from **Compound 22** (36.1 mg, 0.082 mmol, 1.0 equiv.) following General Procedure C, affording the target product in an 82.6% yield (37.5 mg). 1H NMR (500 MHz, Chloroform-d) δ 7.55 – 7.38 (m, 3H), 6.65 (d, J = 9.0 Hz, 2H), 5.07 (s, 2H), 3.52 – 3.21 (m, 4H), 2.99 (s, 6H), 2.89 (t, J = 6.5 Hz, 2H), 2.77 (t, J = 6.1 Hz, 2H), 1.98 (m, 4H), 0.89 (s, 9H), 0.12 (s, 6H).13C NMR (126 MHz, Chloroform-d) δ 152.91, 132.92, 124.12, 118.33, 111.87, 98.89, 81.64, 61.89, 50.17, 49.74, 40.35, 29.86, 27.88, 25.99, 21.78, 20.82, 20.59, -4.91. HRMS (ESI, Pos, m/z): calcd for $C_{32}H_{41}N_2O_3Si$ [M+H]$^+$: 529.2886, found: 529.2875.

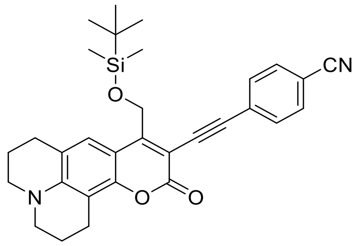

Compound 23-3: This compound was synthesized from **Compound22** (37.8 mg, 0.086 mmol, 1.0 equiv.) following General Procedure C, affording the target product in a 49.7% yield (21.9 mg).

^{1}H NMR (400 MHz, Chloroform-d) δ 7.62 (s, 4H), 7.44 (s, 1H), 5.04 (s, 2H), 3.30 (dt, J = 8.1, 5.8 Hz, 4H), 2.89 (t, J = 6.5 Hz, 2H), 2.78 (t, J = 6.3 Hz, 2H), 2.04 – 1.94 (m, 4H), 0.89 (s, 9H), 0.12 (s, 6H). ^{13}C NMR (101 MHz, Chloroform-d) δ 161.21, 155.50, 151.03, 146.87, 132.16,

132.01, 128.43, 124.46, 118.76, 118.70, 111.42, 107.77, 106.29, 101.43, 95.51, 88.61, 61.72, 50.23, 49.78, 27.87, 25.92, 21.60, 20.47, 18.34, -4.92. HRMS (ESI, Pos, m/z): calcd for $C_{31}H_{35}N_2O_3Si$ $[M+H]^+$: 511.2417, found: 511.2483.

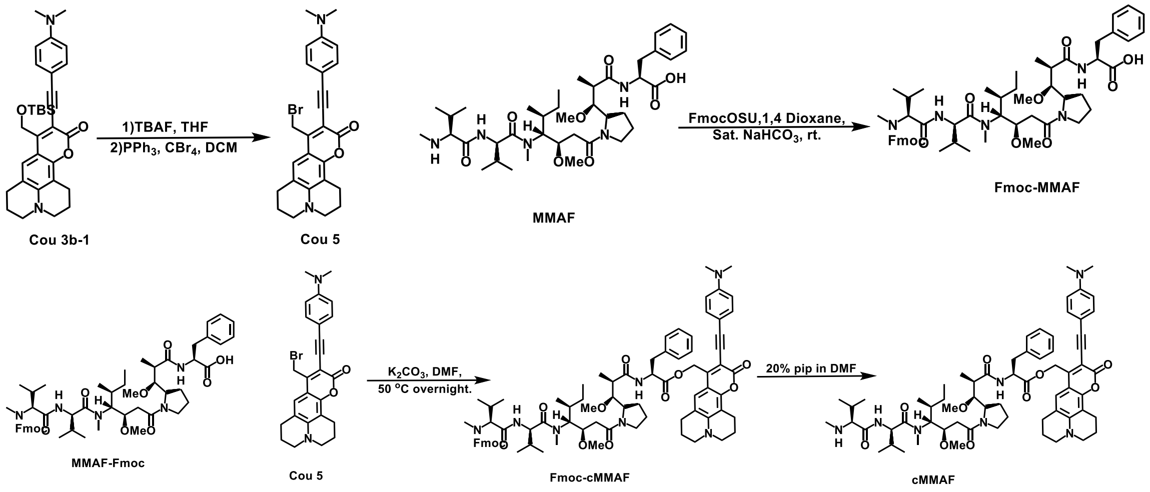

Figure S2. The synthesis of cMMAF.

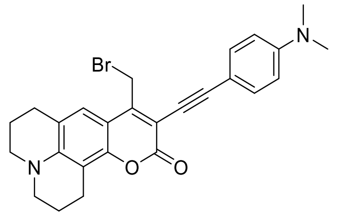

Cou 5: The crude material (14 mg, 0.23 mmol, 1.0 equiv.) was dissolved in THF in a round-bottom flask equipped with a magnetic stirring bar. A 1 M solution of TBAF (253 µL, 0.25 mmol, 1.1 eq.) was added, and the mixture was stirred at 23 °C for 0.5 h. The solvent was removed under reduced pressure, and the residue was passed through a short plug of silica gel and concentrated to give the crude alcohol.

To a Schlenk tube under a nitrogen atmosphere, the crude alcohol and triphenylphosphine (120.7 mg, 0.46 mmol, 2.0 eq.) were added and dissolved in dry DCM at 0°C. The carbon tetrabromide (152.5 mg, 0.46 mmol, 2.0 eq.) was added to the solution. The mixture was stirring for 20 min at 0°C, warmed up to 23 °C and stirred until completion (monitored by TLC). The solvent was evaporated, and the residue was purified by column chromatography to afford the desired product (8.3 mg, 64.9%).

^{1}H NMR (400 MHz, Chloroform-*d*) δ 7.48 (d, J = 8.9 Hz, 2H), 7.06 (s, 1H), 6.66 (d, J = 8.9 Hz, 2H), 4.74 (s, 2H), 3.28 (dt, J = 10.8, 5.6 Hz, 4H), 3.00 (s, 6H), 2.89 (t, J = 6.5 Hz, 2H), 2.81 (t, J = 6.3 Hz, 2H), 2.25 – 1.77 (m, 4H). ^{13}C NMR (101 MHz, $CDCl_3$) δ 160.87, 150.46, 150.38, 149.31, 146.27, 133.13, 121.64, 118.88, 111.85, 109.90, 107.07, 106.50, 105.13, 101.57, 81.52, 77.48, 50.16, 49.74, 40.33, 29.85, 27.96, 21.59, 20.65, 20.57. HRMS (ESI, Pos, m/z): calcd for $C_{26}H_{26}BrN_2O_2$ $[M+H]^+$: 477.1178, found: 477.1146.

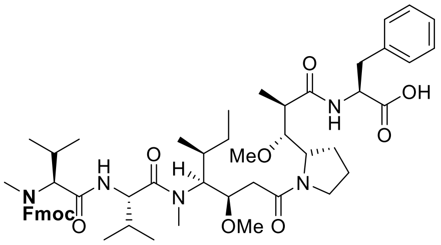

Fmoc-MMAF: MMAF (22.8 mg, 31.2 μmol, 1.0 equiv.) was dissolved in saturated aqueous $NaHCO_3$ solution (0.5 mL) in a 1.5 mL Eppendorf tube. Fmoc-OSu (15.0 mg, 44.0 μmol, 1.4 equiv.) dissolved in 1,4-dioxane (0.5 mL) was added dropwise, and the reaction mixture was stirred at 23 °C overnight. After completion (monitored by LC–MS), the organic solvent was removed under reduced pressure, and the aqueous layer was extracted with EtOAc (3 × 1 mL) and brine (1 mL). The combined organic extracts were dried over anhydrous Na_2SO_4, filtered, and concentrated under reduced pressure. The crude residue was purified by silica gel column chromatography (10% MeOH in DCM) to afford Fmoc–MMAF as a white solid.

Chemical Formula: $C_{54}H_{75}N_5O_{10}$ Exact Mass: 953.55 Molecular Weight: 954.22 LC-MS (ESI): RT=3.38min. Absorbance at =266 nm. m/z expected for $[M+H]^+$: 954.22, m/z observed: 954.41. HRMS (ESI, Pos, m/z): calcd for $C_{54}H_{76}N_5O_{10}$ $[M+H]^+$: 954.5592, found: 954.5571.

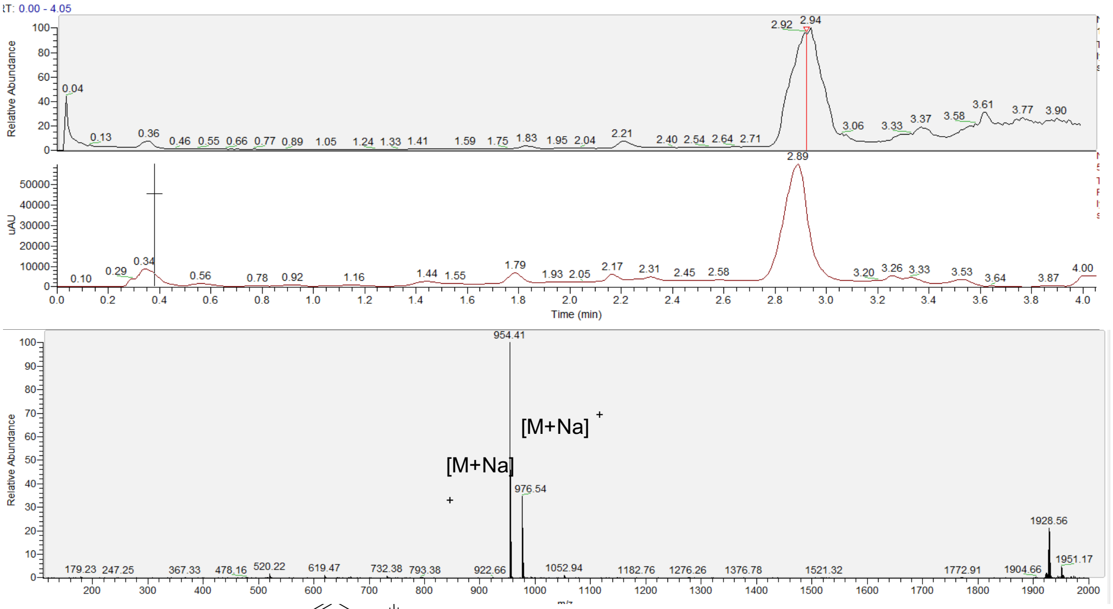

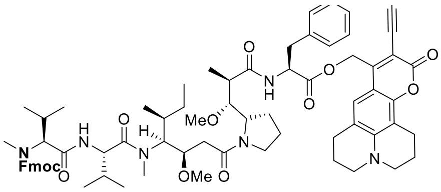

Fmoc-cMMAF

Fmoc-cMMAF: Cou 5 (10.0 mg, 21.0 μmol, 1.0 equiv.) was dissolved in DMF (0.5 mL) in a 1.5 mL Eppendorf tube. K_2CO_3 (4.3 mg, 31.5 μmol, 1.5 equiv) was added, followed by Fmoc–MMAF (1.0 equiv, 21.0 μmol). The reaction mixture was shaken at 23 °C overnight. Upon completion (monitored by LC–MS), the solvent was removed under reduced pressure, and the crude product was purified by preparative HPLC. The purified

fraction was lyophilized to afford Fmoc-cMMAF as a solid (Yield:35.7%).

Chemical Formula: $C_{80}H_{99}N_7O_{12}$ Exact Mass: 1349.74 Molecular Weight: 1350.71 LC-MS (ESI): RT=3.38min. Absorbance at =466 nm. m/z expected for $[M+H]^+$: 1350.71, m/z observed: 1350.79. HRMS (ESI, Pos, m/z): calcd for $C_{80}H_{100}N_7O_{12}$ $[M+H]^+$: 1350.7430, found: 1350.7434.

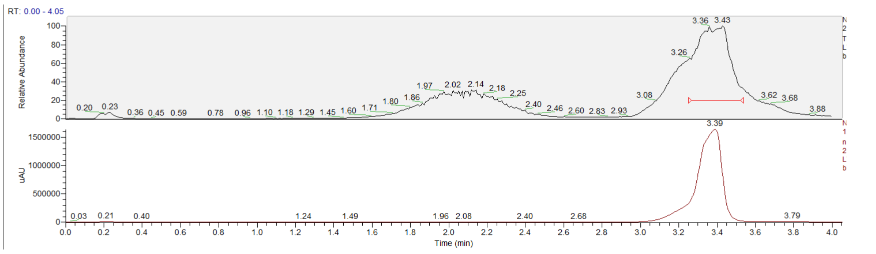

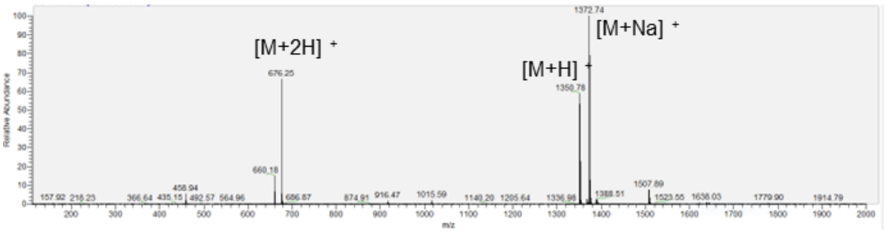

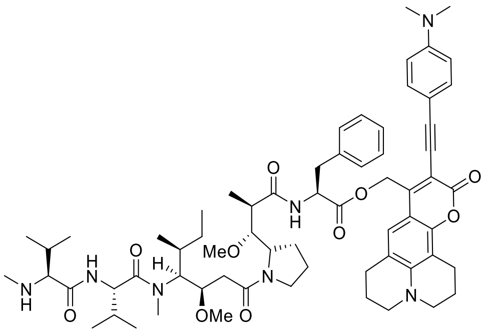

cMMAF

cMMAF: To the Fmoc-cMMAF, 20% piperidine in DMF (0.5 mL) was added, and the mixture was shaken at 23 °C until completion (monitored by LC–MS). The reaction mixture was concentrated under reduced pressure, and the crude product was purified by preparative HPLC to afford the desired deprotected conjugate (Yield 54.5%). ^{1}H NMR (500 MHz, DMSO-d_6) δ 8.79 (m, 4H), 8.52 (d, J = 8.4 Hz, 1H), 8.29 (d, J = 7.8 Hz, 1H), 7.33 (d, J = 8.5 Hz, 4H), 7.24 – 7.06 (m, 12H), 6.77 – 6.67 (m, 4H), 5.47 (s, 2H), 5.43 (s, 2H), 4.80 – 4.48 (m, 6H), 3.97 (m, 2H), 3.75 – 3.61 (m, 3H), 3.55 – 3.44 (m, 2H), 3.40 (m, 2H), 3.29 (m, 9H), 3.23 (s, 3H), 3.21 (m, 1H), 3.18 (s, 6H), 3.15 (s, 3H), 3.14 (m, 1H), 3.07 (m, 1H), 3.03 (m, 3H), 3.00 (m, 2H), 2.97 (15H), 2.89 – 2.80 (m, 2H), 2.74 (m, 8H), 2.47 (t, J = 5.1 Hz, 6H), 2.42 – 2.32 (m, 2H), 2.28 – 2.14 (m, 4H), 2.12 – 1.98 (m, 4H), 1.89 (m, 8H), 1.76 (m, 4H), 1.63 (m, 2H), 1.47 – 1.39 (m, 1H), 1.38 – 1.33 (m, 1H), 1.33 – 1.21 (m, 3H), 1.17 (m, 1H), 0.99 – 0.85 (m, 38H), 0.75 (m, 6H). ^{13}C NMR (126 MHz, DMSO) δ 174.07, 173.93, 172.56, 172.44, 171.82, 171.62, 169.40, 169.17, 166.45, 160.09, 150.67, 150.06, 148.27, 148.23, 146.52, 137.53, 137.28, 132.79, 129.32, 129.01, 128.56, 126.94, 126.82, 123.13, 123.02, 119.18, 119.13, 112.37, 112.32, 108.93, 108.89, 107.07, 105.97, 105.91, 104.56, 104.52, 99.55, 99.46, 85.66, 82.09, 81.74, 78.12, 66.05, 66.00, 61.63, 61.59, 61.42, 60.65, 58.99, 58.71, 57.62, 57.53, 56.33, 55.65, 55.10, 55.04, 53.71, 52.45, 49.24, 47.54, 46.60, 44.19, 43.55, 43.30, 40.16, 37.44, 36.76, 36.70, 35.55, 32.48, 32.44, 32.27, 32.04, 31.89, 31.73, 30.54, 30.40, 29.97, 29.92, 27.52, 25.84, 25.80, 25.65, 24.71, 24.67, 23.51,

22.68, 22.09, 21.29, 20.41, 20.31, 19.17, 18.99, 18.88, 18.77, 18.73, 18.09, 16.00, 15.81, 15.44, 15.14, 10.82, 10.59.

Chemical Formula: $C_{65}H_{89}N_7O_{10}$ Exact Mass: 1127.67 Molecular Weight: 1128.47 LC-MS (ESI): RT=2.97min. Absorbance at =472 nm. m/z expected for $[M+H]^+$: 1128.47, m/z observed: 1128.54. HRMS (ESI, Pos, m/z): calcd for $C_{65}H_{90}N_7O_{10}$ $[M+H]^+$: 1128.6749, found: 1128.6747.

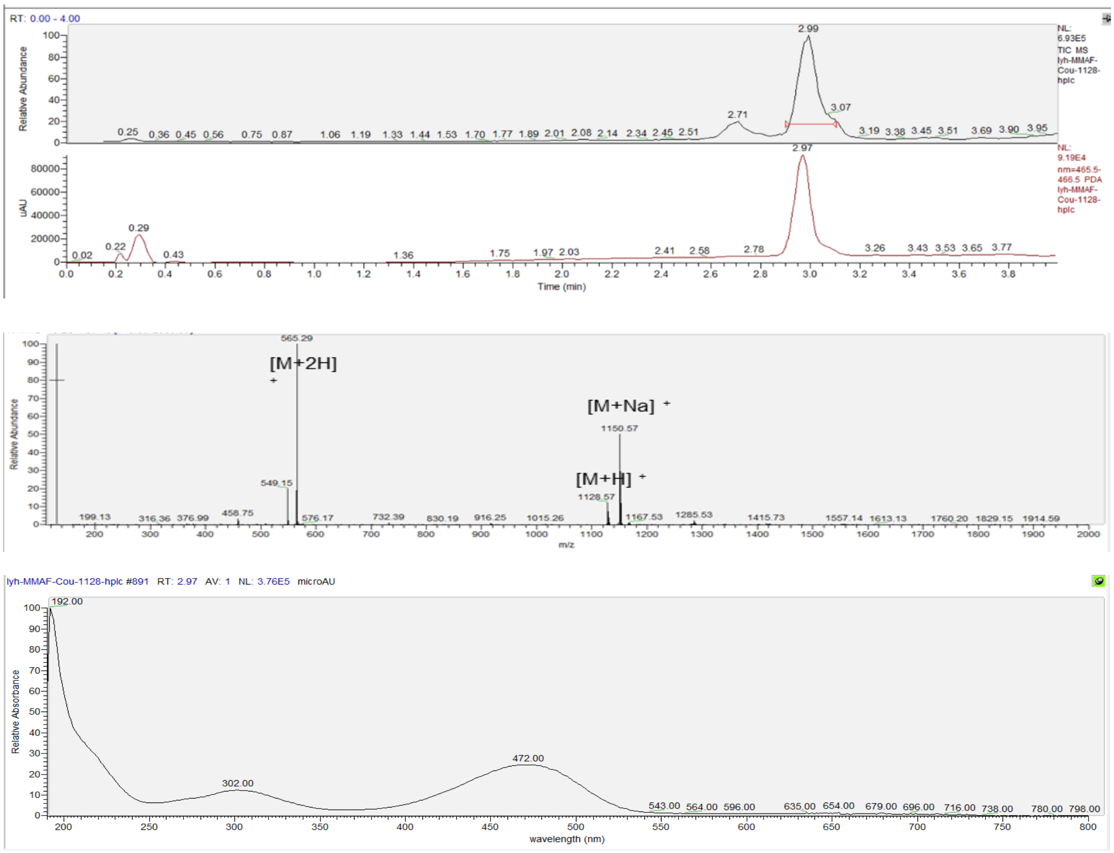

The conversion of the reaction to synthesize Fmoc-cMMAF (50 °C overnight)

4.Competition experiments

In the subsequent photolysis competition experiments, the following assumptions were applied for kinetic analysis. Under experimental conditions, the photon flux provided by the LED light source is considered as sufficiently high relative to the number of reacting molecules and therefore not rate-limiting. As a result, the number of incident photons can be treated as effectively constant and in excess over the time scale of the experiment, such that photon depletion does not influence the observed reaction rate.

Under this assumption, the photolysis process can be approximated as pseudo-first-order kinetics, and the experimental data were fitted using the first-order rate equation $\ln\left(\frac{[S]_t}{[S]_0}\right) = -k_{obs}t$, where the $[S]_t$ and $[S]_0$ denote the substrate

concentration at time t and $t = 0$, respectively, and k_{obs} is the apparent first order rate constant.

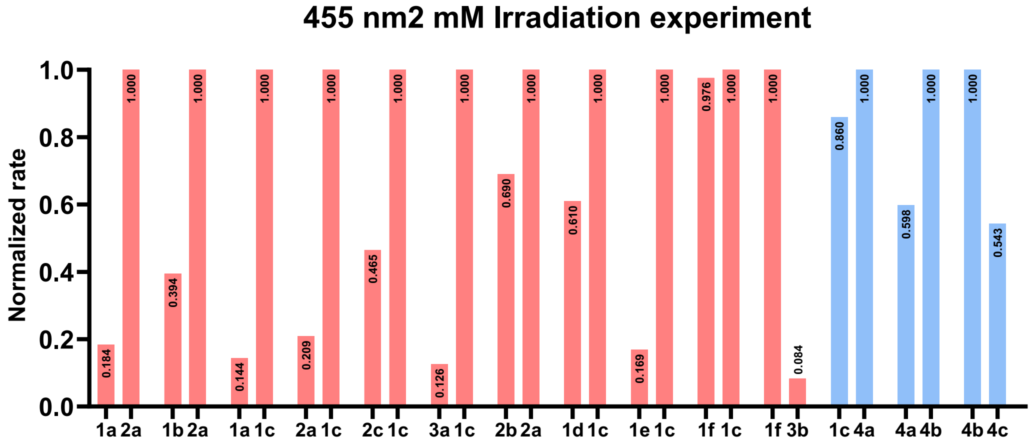

Figure S3 presents the NMR monitoring results of the competition experiment. The consumption rates of the starting materials corresponding to the relatively faster-reacting compound were normalized to 1.000. The rate for the other compounds was expressed relative rate. Experimental conditions: irradiation with a 455 nm LED light source; initial concentration of substrates were about 2 mM in a total reaction volume of 0.5 mL; mixed solvent system of DMSO-d6 and D_2O (vDMSO: vH_2O = 9:1). The comsumption of the faster compounds was between 25%-80% in all the competition experiments. Durene is the reference for NMR competition experiments (Tetramethylsilane was as the reference for competition between Cou 1a and Cou 2a). Comparation between Cou 1f and Cou 3b was carried out by LCMS at 200uM in DMSO/H_2O (vDMSO: vH_2O = 9:1), Compound 4 was as the reference.

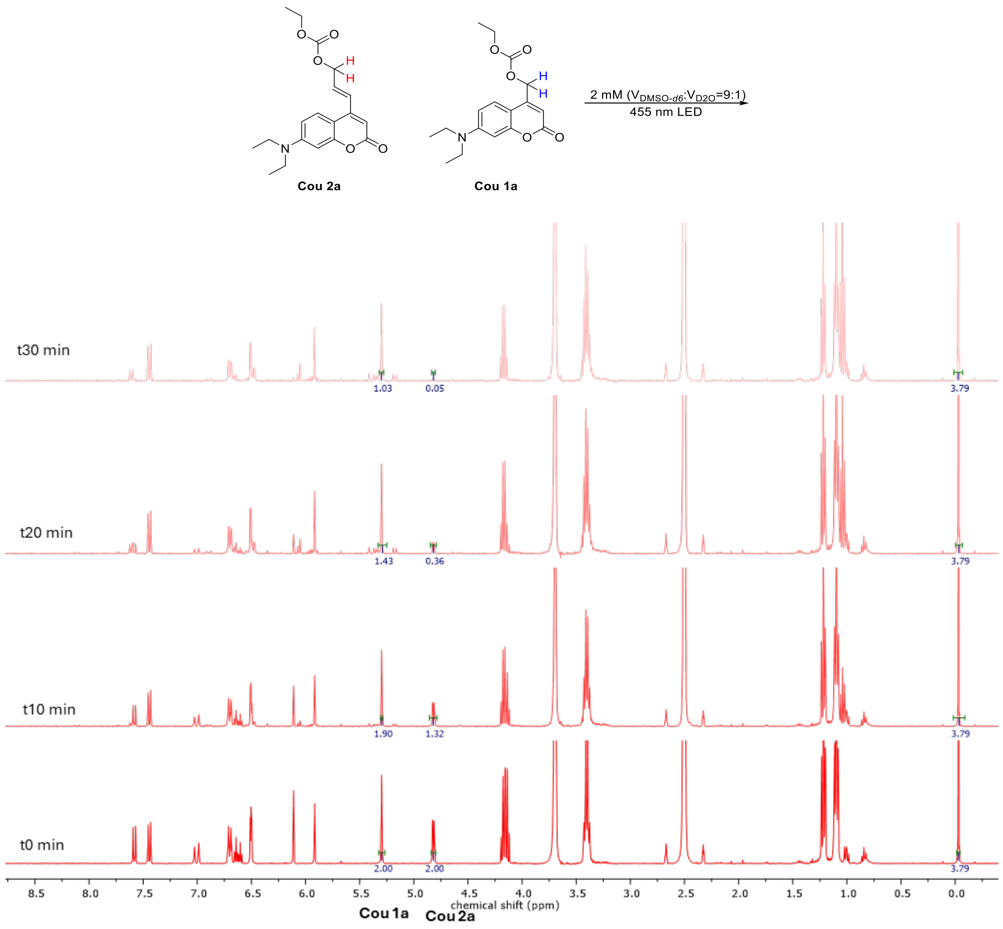

TMS was the reference.

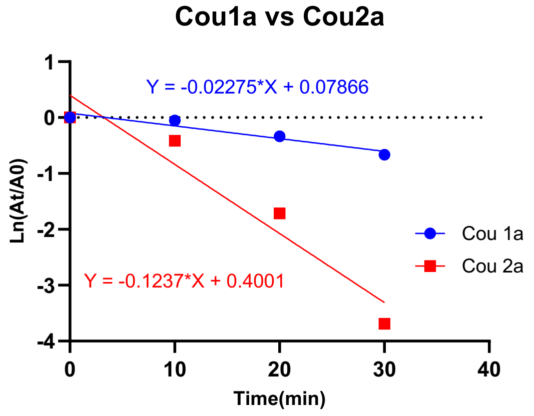

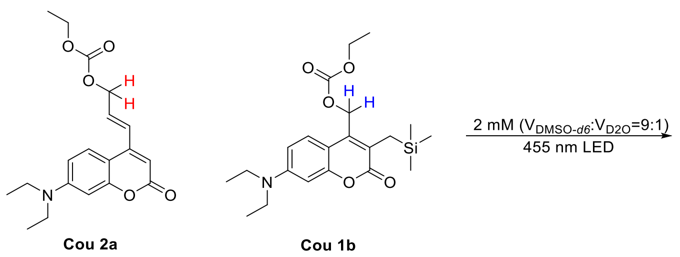
Cou 2a
Cou 1b
2 mM (V_{DMSO-d6}:V_{D2O}=9:1)
455 nm LED

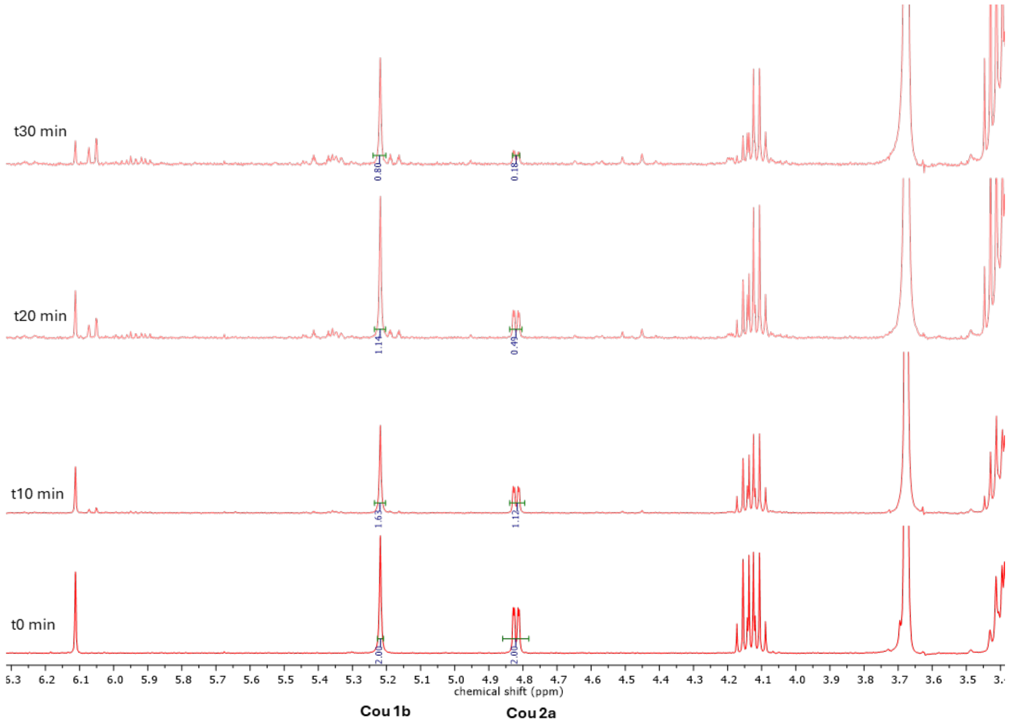
t30 min
t20 min
t10 min
t0 min
chemical shift (ppm)
Cou 1b
Cou 2a

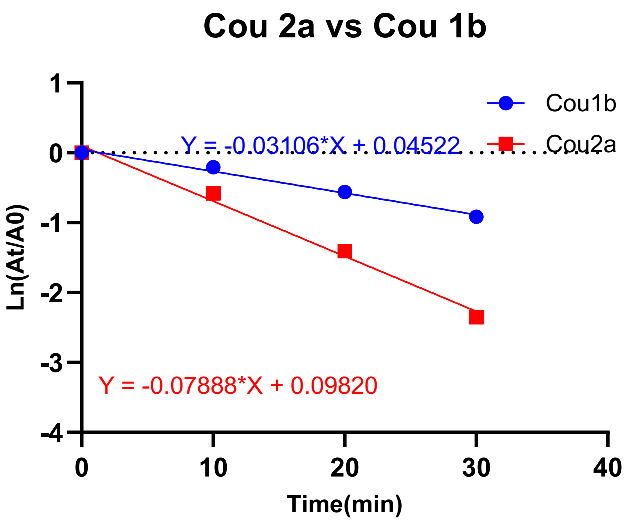
Cou 2a vs Cou 1b
Cou1b
Cou2a
Y = -0.03106*X + 0.04522
Y = -0.07888*X + 0.09820
Ln(At/A0)
Time(min)

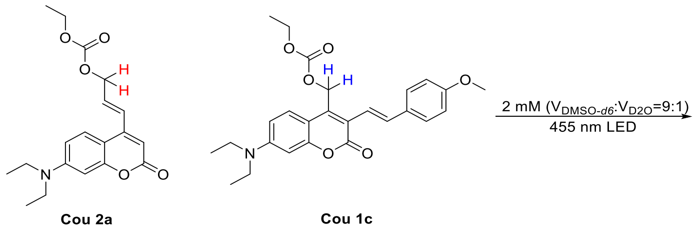
Cou 2a
Cou 1c
2 mM (V_DMSO-d6:V_D2O=9:1)
455 nm LED

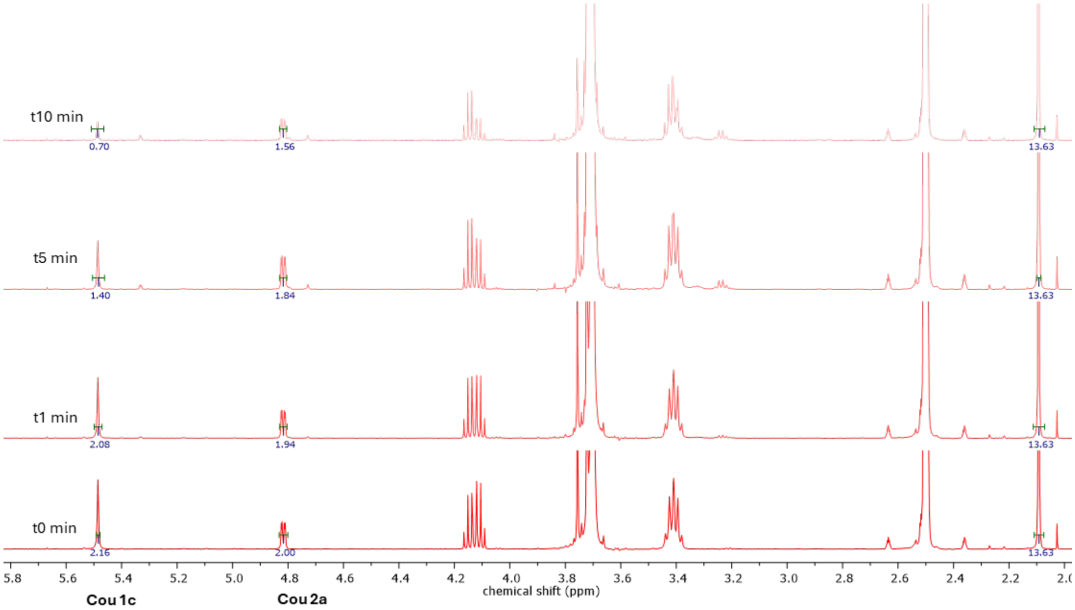
t10 min
t5 min
t1 min
t0 min
chemical shift (ppm)
Cou 1c
Cou 2a

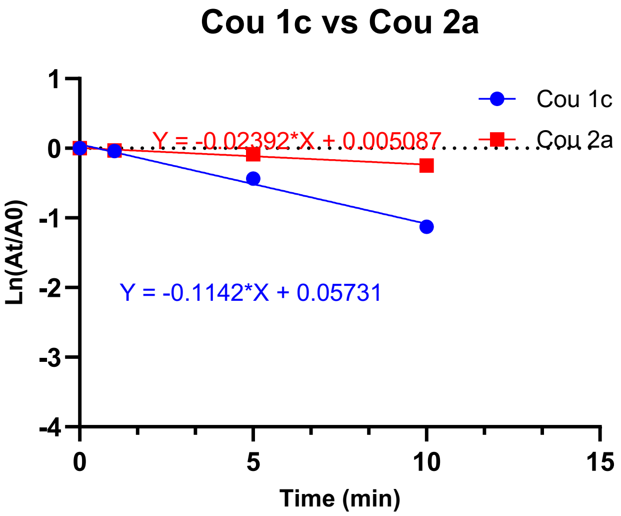
Cou 1c vs Cou 2a
Cou 1c
Cou 2a
Y = -0.02392*X + 0.005087
Y = -0.1142*X + 0.05731
Ln(At/A0)
Time (min)

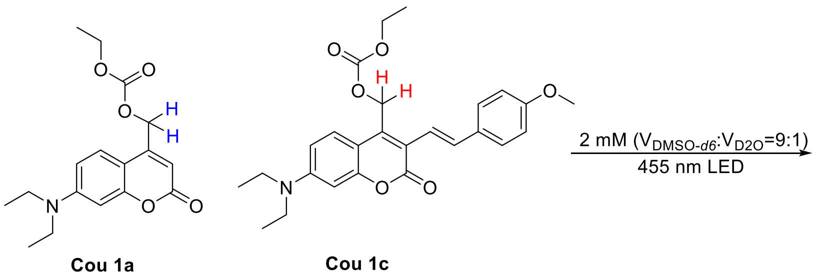
2 mM (V_DMSO-d6:V_D2O=9:1)
455 nm LED
Cou 1a
Cou 1c

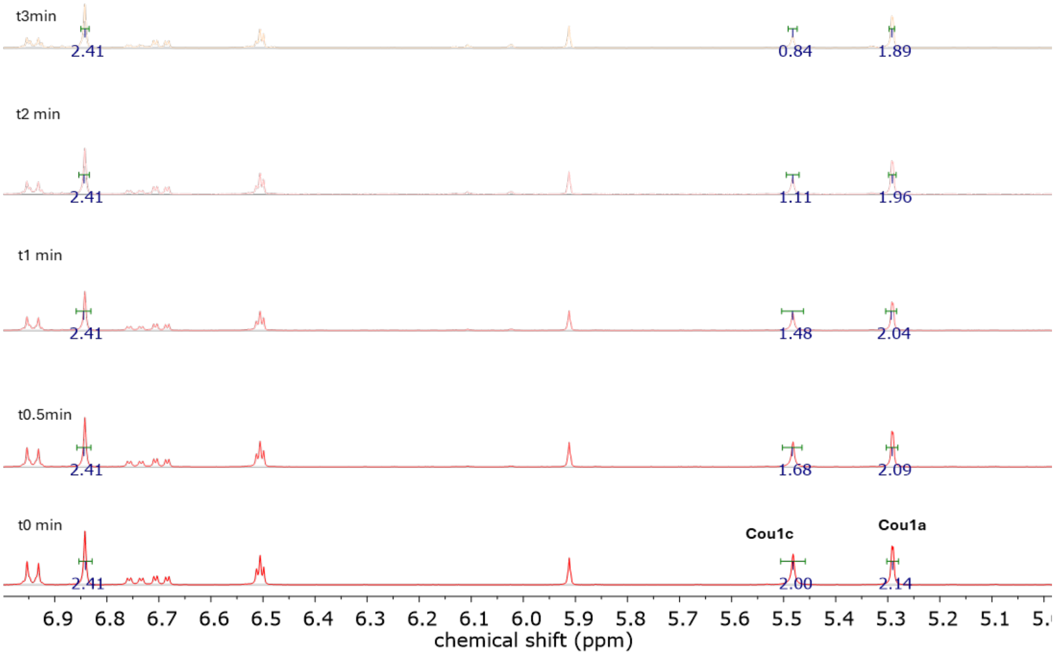
t3min
2.41
0.84
1.89
t2 min
2.41
1.11
1.96
t1 min
2.41
1.48
2.04
t0.5min
2.41
1.68
2.09
t0 min
Cou1c
Cou1a
2.41
2.00
2.14
chemical shift (ppm)

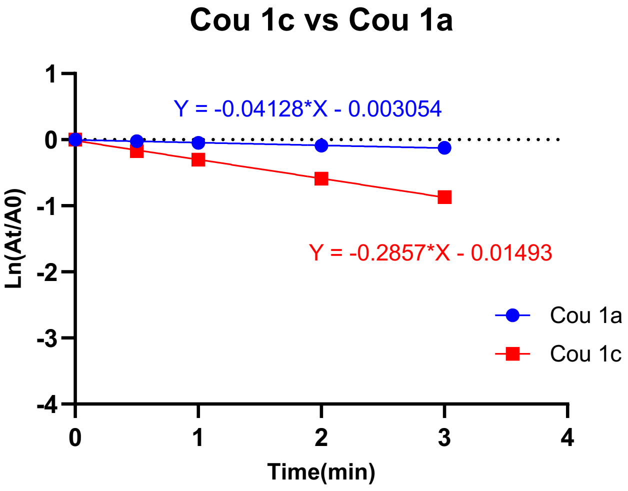
Cou 1c vs Cou 1a
Y = -0.04128*X - 0.003054
Y = -0.2857*X - 0.01493
Ln(At/A0)
Time(min)
Cou 1a
Cou 1c

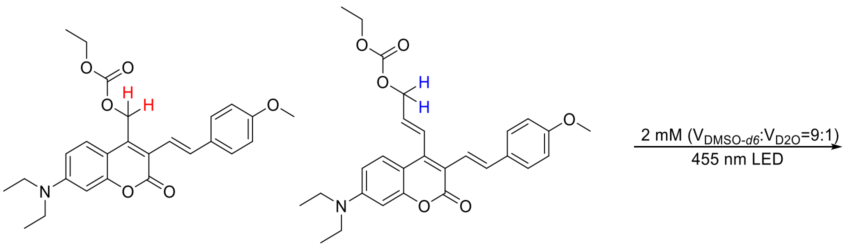

2 mM (V_{DMSO-d6}:V_{D2O}=9:1)
455 nm LED

Cou 1c Cou 2c

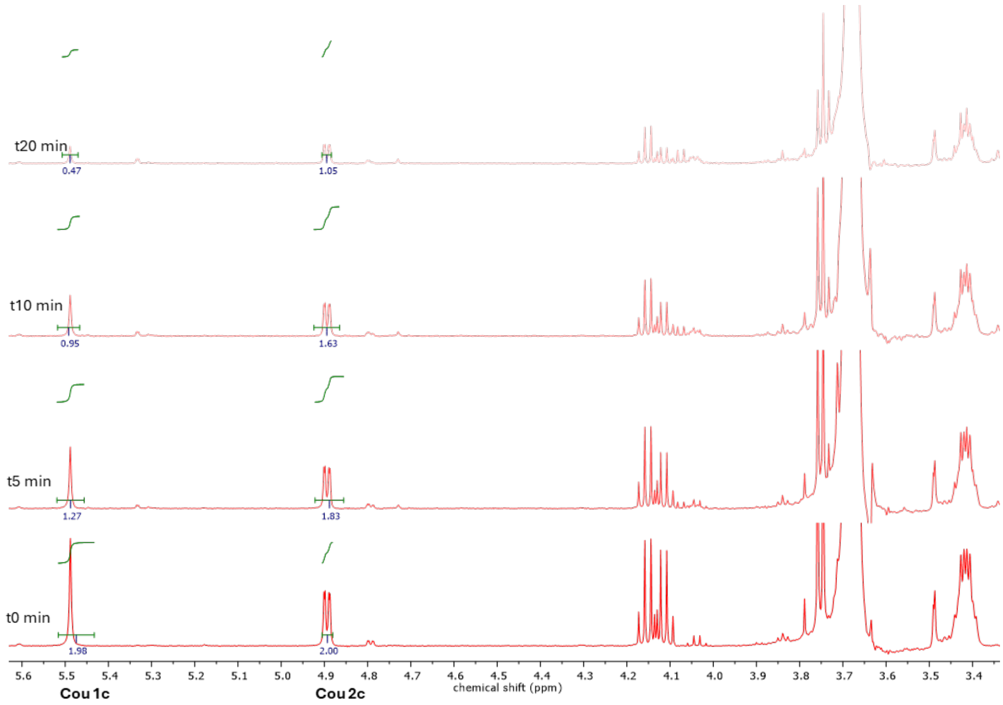

t20 min
t10 min
t5 min
t0 min
0.47
1.05
0.95
1.63
1.27
1.83
1.98
2.00
chemical shift (ppm)
Cou 1c
Cou 2c

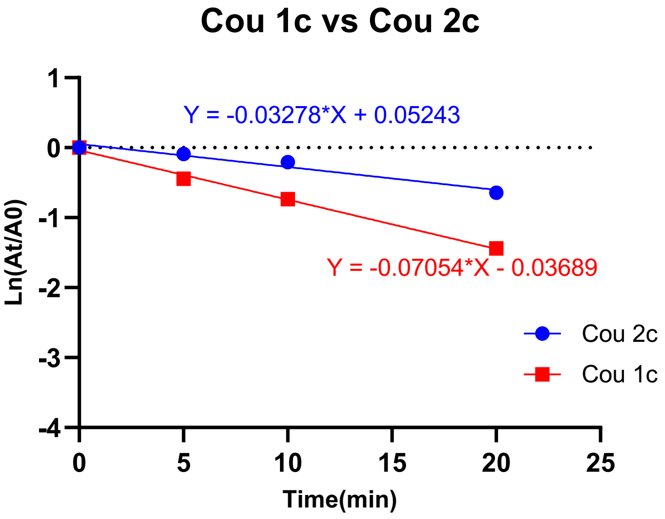

Cou 1c vs Cou 2c
Y = -0.03278*X + 0.05243
Y = -0.07054*X - 0.03689
Ln(At/A0)
Time(min)
Cou 2c
Cou 1c

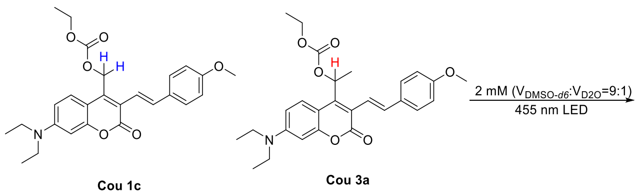

Cou 1c
Cou 3a
2 mM (VDMSO-d6:VD2O=9:1)
455 nm LED

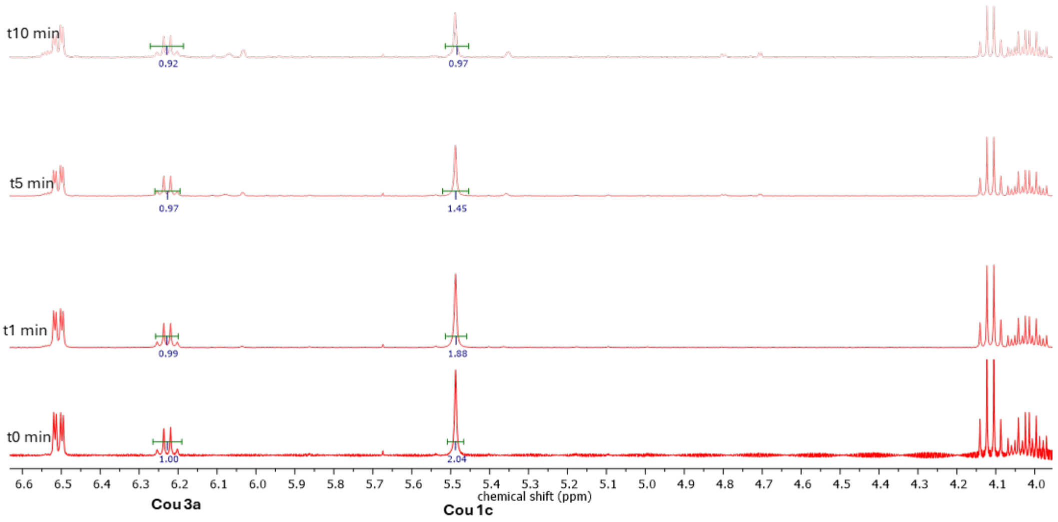

t10 min
t5 min
t1 min
t0 min
0.92
0.97
0.97
1.45
0.99
1.88
1.00
2.04
chemical shift (ppm)
Cou 3a
Cou 1c

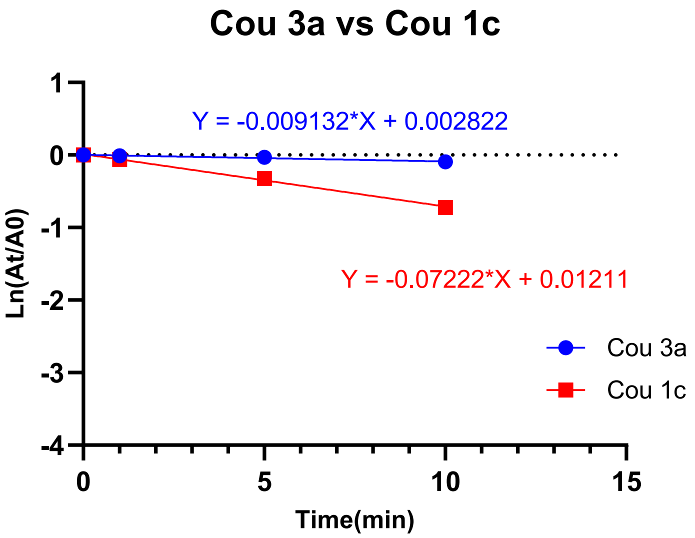

Cou 3a vs Cou 1c
Y = -0.009132*X + 0.002822
Y = -0.07222*X + 0.01211
Ln(At/A0)
Time(min)
Cou 3a
Cou 1c

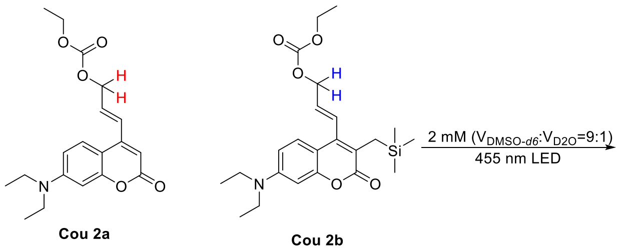
H
H
O
O
O
N
O
O
Cou 2a
H
H
O
O
O
Si
N
O
O
Cou 2b
2 mM (V$_{DMSO-d6}$:V$_{D2O}$=9:1)
455 nm LED

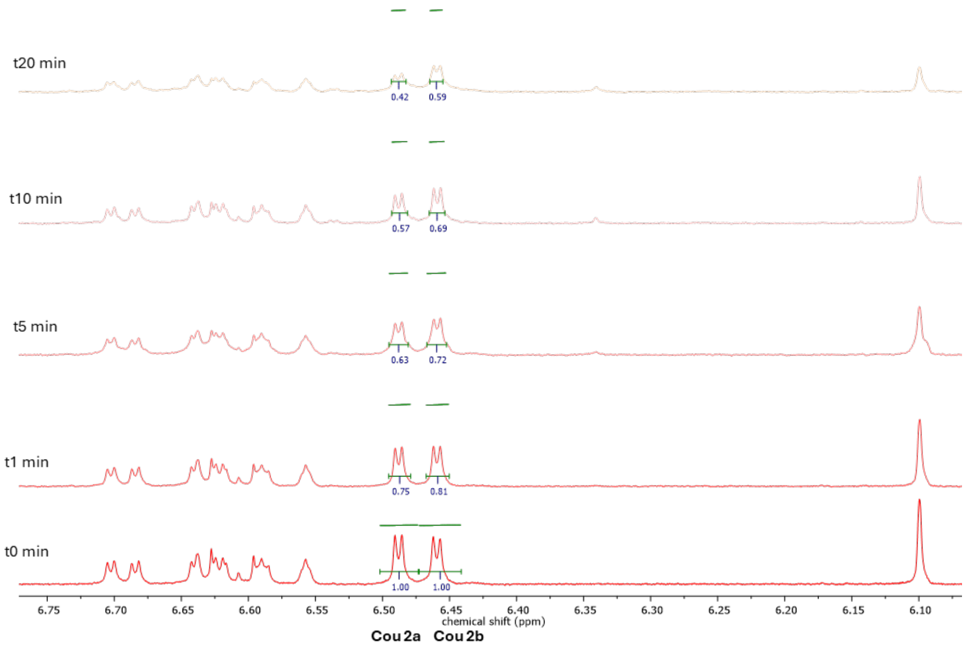
t20 min
0.42
0.59
t10 min
0.57
0.69
t5 min
0.63
0.72
t1 min
0.75
0.81
t0 min
1.00
1.00
6.75
6.70
6.65
6.60
6.55
6.50
6.45
6.40
6.35
6.30
6.25
6.20
6.15
6.10
chemical shift (ppm)
Cou 2a
Cou 2b

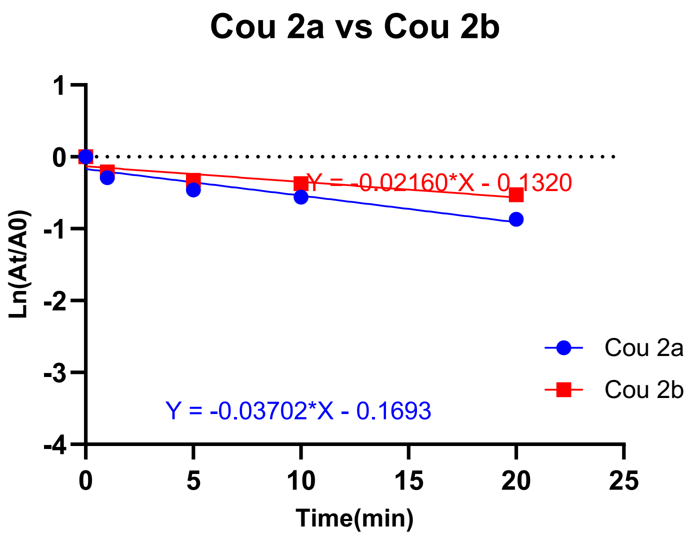
Cou 2a vs Cou 2b
Ln(At/A0)
Time(min)
Y = -0.02160*X - 0.1320
Y = -0.03702*X - 0.1693
Cou 2a
Cou 2b
1
0
-1
-2
-3
-4
0
5
10
15
20
25

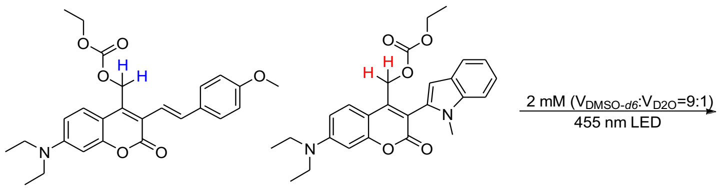
Cou 1c
Cou 1d
2 mM (VDMSO-d6:VD2O=9:1)
455 nm LED

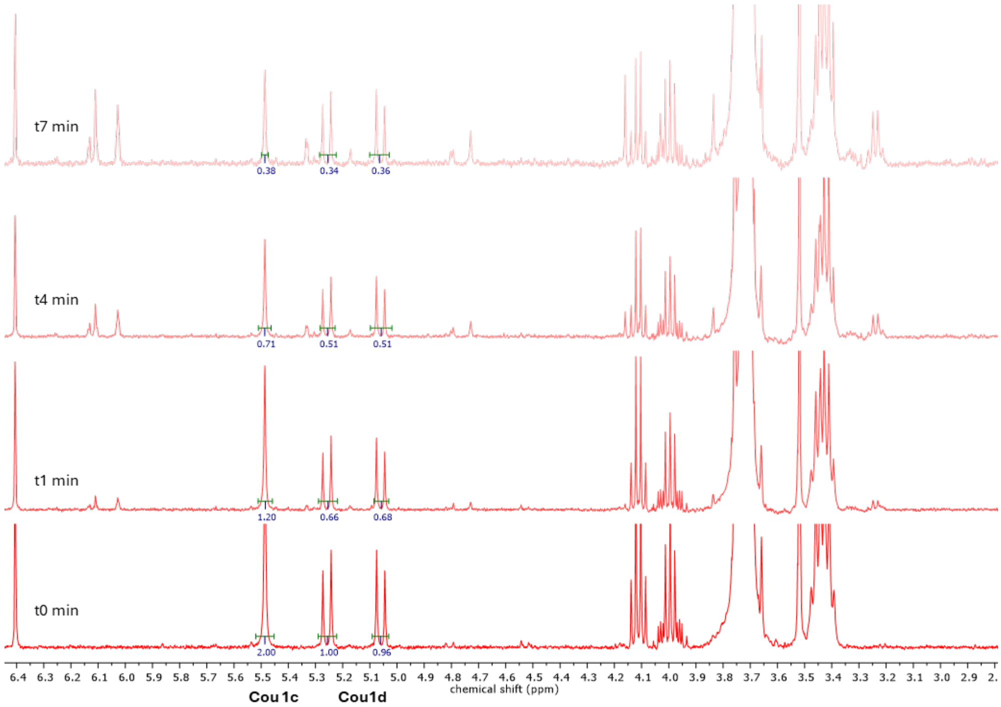
t7 min
t4 min
t1 min
t0 min
chemical shift (ppm)
Cou 1c
Cou1d

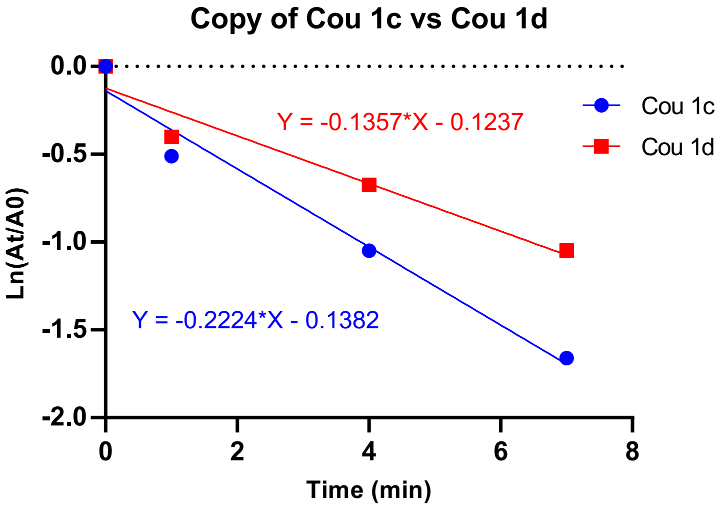
Copy of Cou 1c vs Cou 1d
Ln(At/A0)
Time (min)
Y = -0.1357*X - 0.1237
Y = -0.2224*X - 0.1382
Cou 1c
Cou 1d

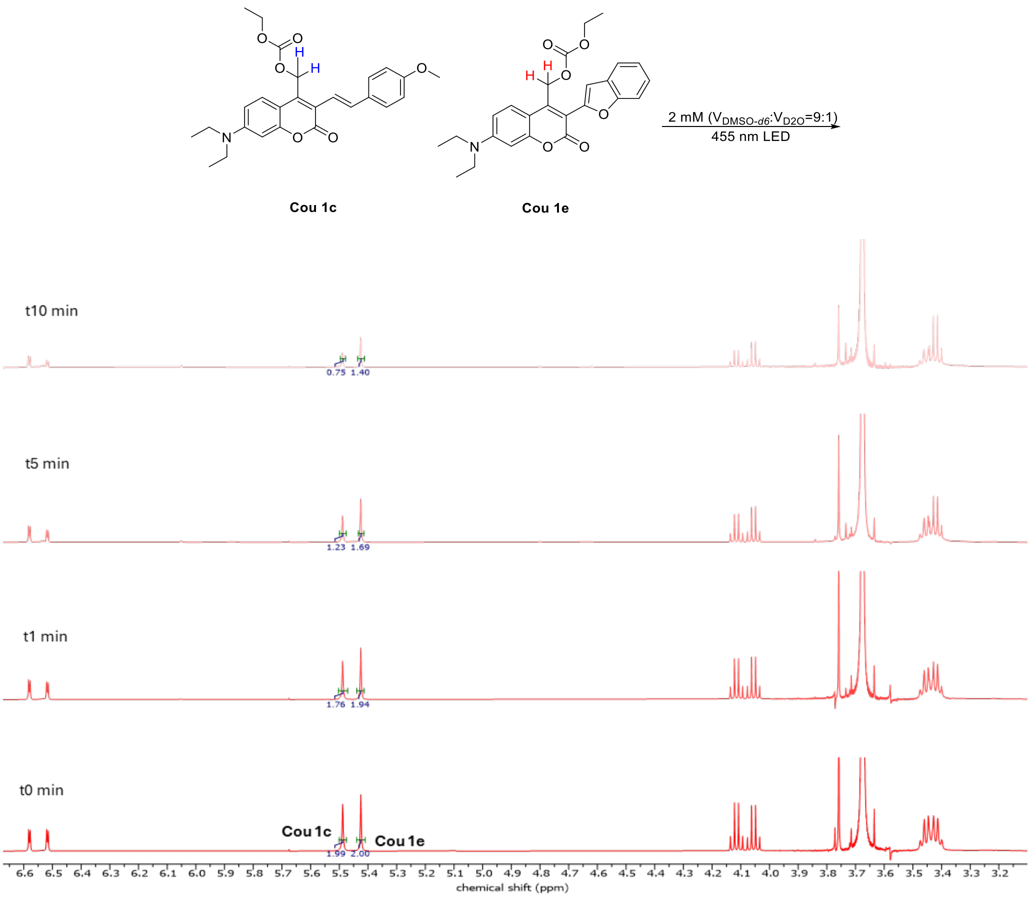

Cou 1c
Cou 1e
2 mM (VDMSO-d6:VD2O=9:1)
455 nm LED
t10 min
0.75 1.40
t5 min
1.23 1.69
t1 min
1.76 1.94
t0 min
Cou 1c
Cou 1e
1.99 2.00
6.6 6.5 6.4 6.3 6.2 6.1 6.0 5.9 5.8 5.7 5.6 5.5 5.4 5.3 5.2 5.1 5.0 4.9 4.8 4.7 4.6 4.5 4.4 4.3 4.2 4.1 4.0 3.9 3.8 3.7 3.6 3.5 3.4 3.3 3.2
chemical shift (ppm)

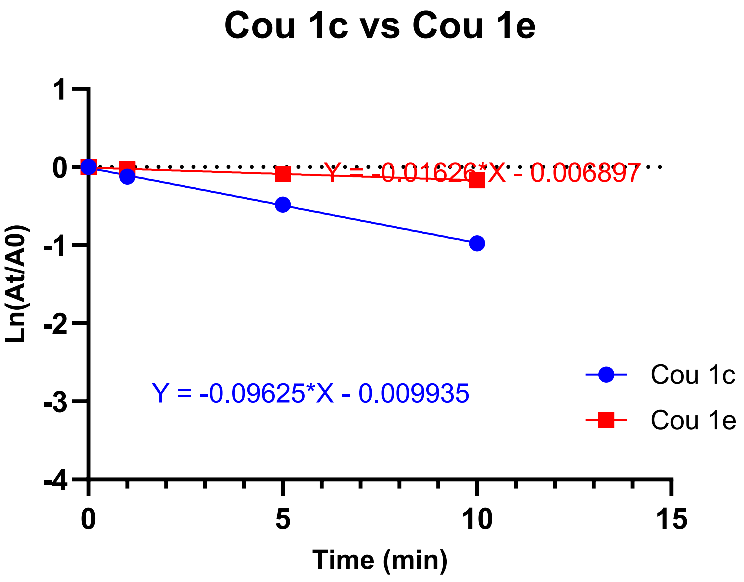

Cou 1c vs Cou 1e
Ln(At/A0)
Time (min)
Y = -0.01626*X - 0.006897
Y = -0.09625*X - 0.009935
Cou 1c
Cou 1e

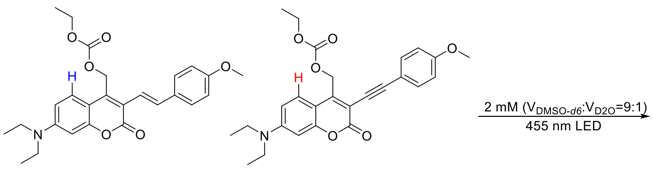
H
N
O
H
2 mM (V$_{DMSO-d6}$:V$_{D2O}$=9:1)
455 nm LED

Cou 1c Cou 1f

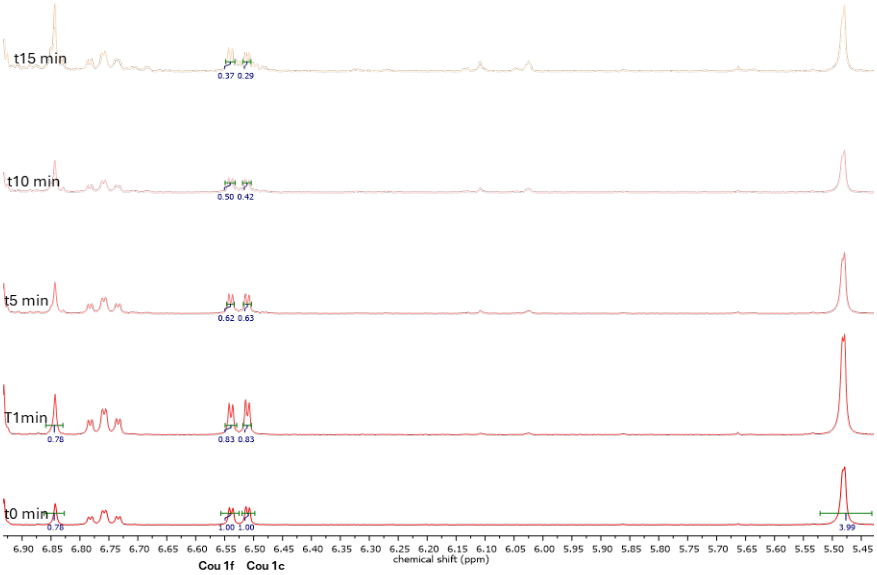
t15 min
0.37 0.29
t10 min
0.50 0.42
t5 min
0.62 0.63
T1min
0.78
0.83 0.83
t0 min
0.78
1.00 1.00
3.99
6.90 6.85 6.80 6.75 6.70 6.65 6.60 6.55 6.50 6.45 6.40 6.35 6.30 6.25 6.20 6.15 6.10 6.05 6.00 5.95 5.90 5.85 5.80 5.75 5.70 5.65 5.60 5.55 5.50 5.45
chemical shift (ppm)
Cou 1f Cou 1c

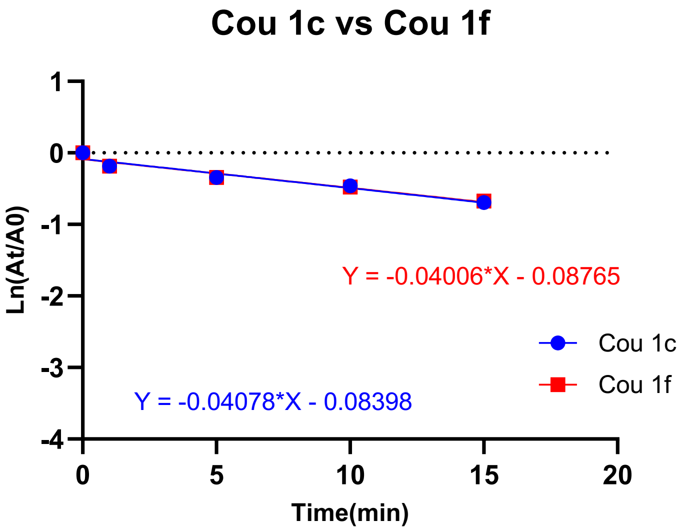
Cou 1c vs Cou 1f
Ln(At/A0)
1
0
-1
-2
-3
-4
Y = -0.04006*X - 0.08765
Y = -0.04078*X - 0.08398
Cou 1c
Cou 1f
0
5
10
15
20
Time(min)

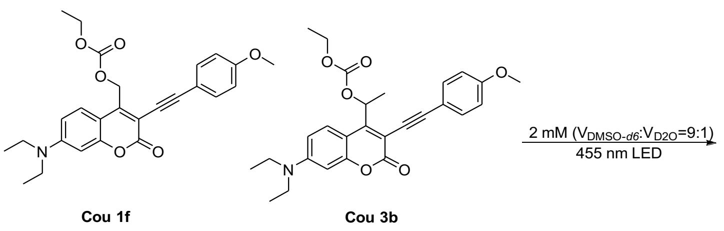
Cou 1f
Cou 3b
2 mM (VDMSO-d6:VD2O=9:1)
455 nm LED

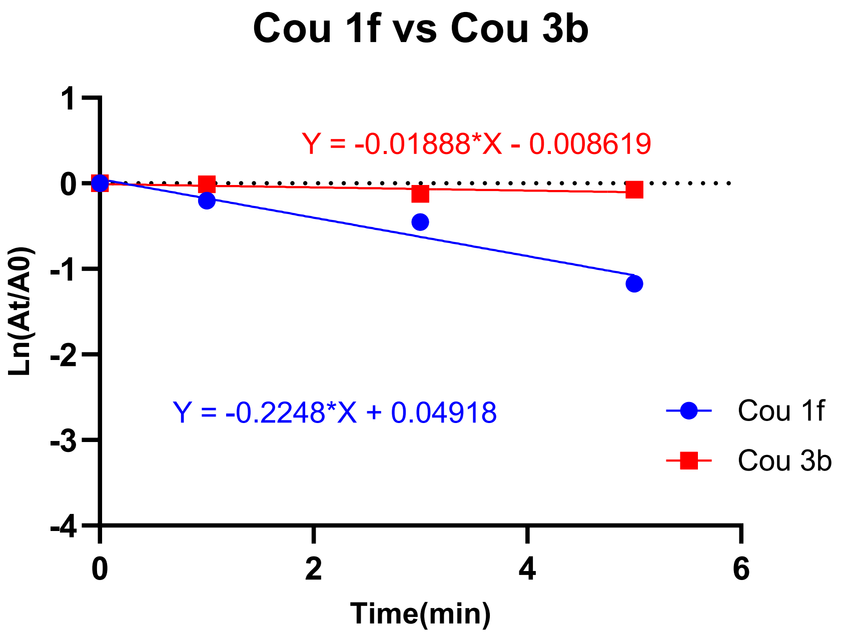
Cou 1f vs Cou 3b
Y = -0.01888*X - 0.008619
Y = -0.2248*X + 0.04918
Cou 1f
Cou 3b
Ln(At/A0)
Time(min)

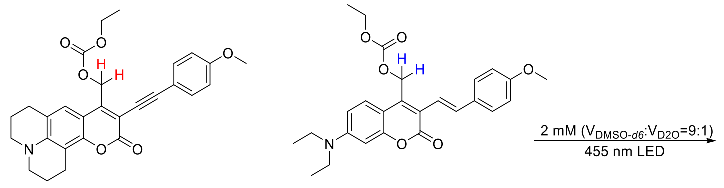
2 mM ($V_{DMSO\text{-}d6}$:V_{D2O}=9:1)
455 nm LED

Cou 4a **Cou 1c**

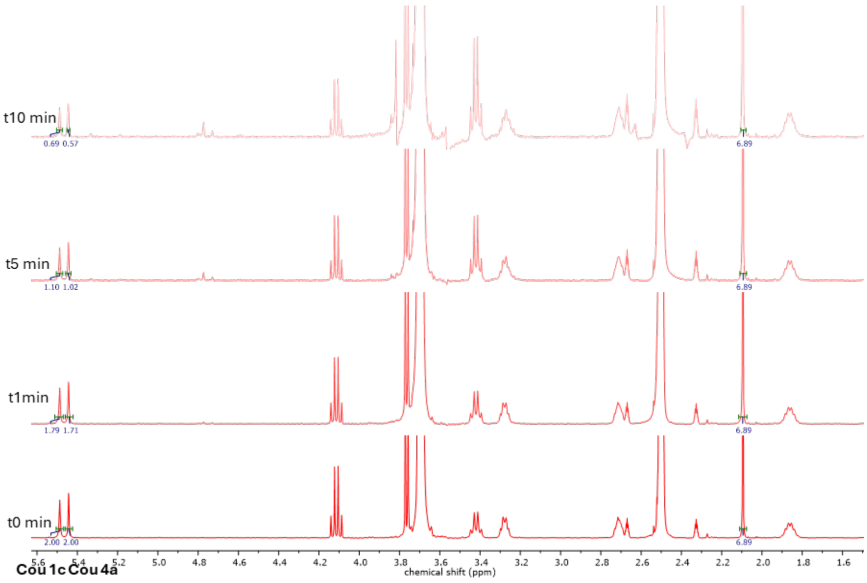
t10 min
0.69 0.57
6.89
t5 min
1.10 1.02
6.89
t1min
1.79 1.71
6.89
t0 min
2.00 2.00
6.89
Cou 1c Cou 4a
chemical shift (ppm)

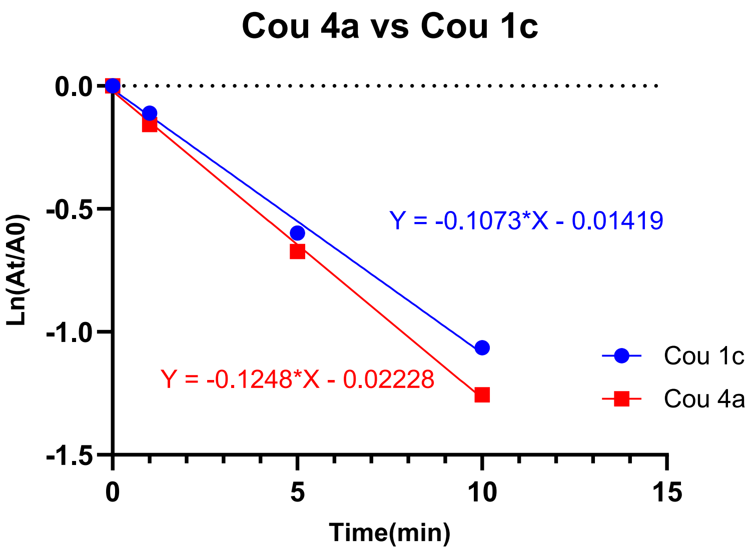
Cou 4a vs Cou 1c
Ln(At/A0)
Time(min)
Y = -0.1073*X - 0.01419
Y = -0.1248*X - 0.02228
Cou 1c
Cou 4a

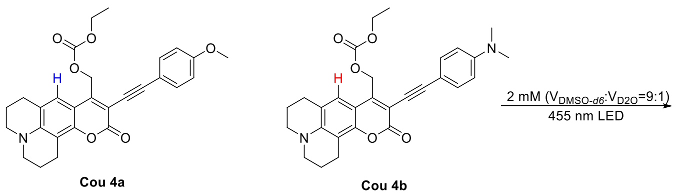
Cou 4a
Cou 4b
2 mM (V$_{DMSO-d6}$:V$_{D2O}$=9:1)
455 nm LED

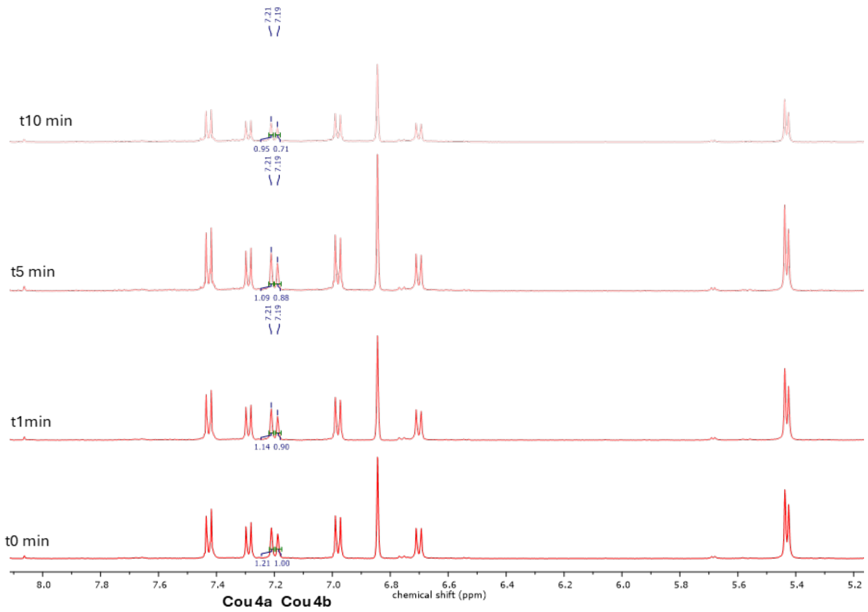
t10 min
t5 min
t1min
t0 min
chemical shift (ppm)
Cou 4a Cou 4b

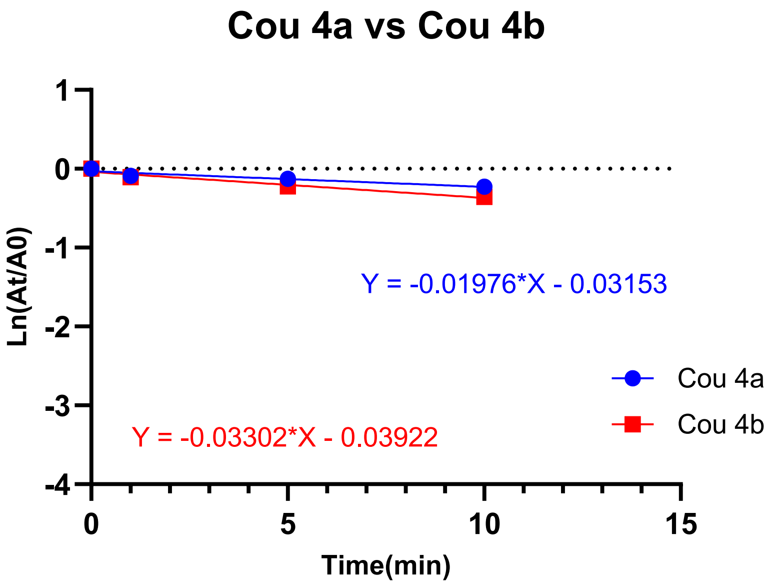
Cou 4a vs Cou 4b
Ln(At/A0)
Time(min)
Y = -0.01976*X - 0.03153
Y = -0.03302*X - 0.03922
Cou 4a
Cou 4b

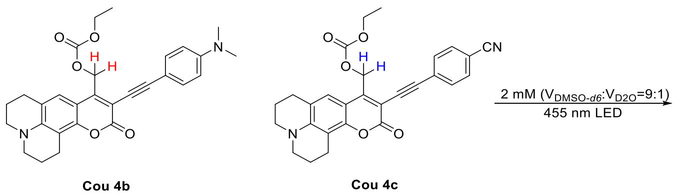
Cou 4b
Cou 4c
2 mM (V_DMSO-d6:V_D2O=9:1)
455 nm LED

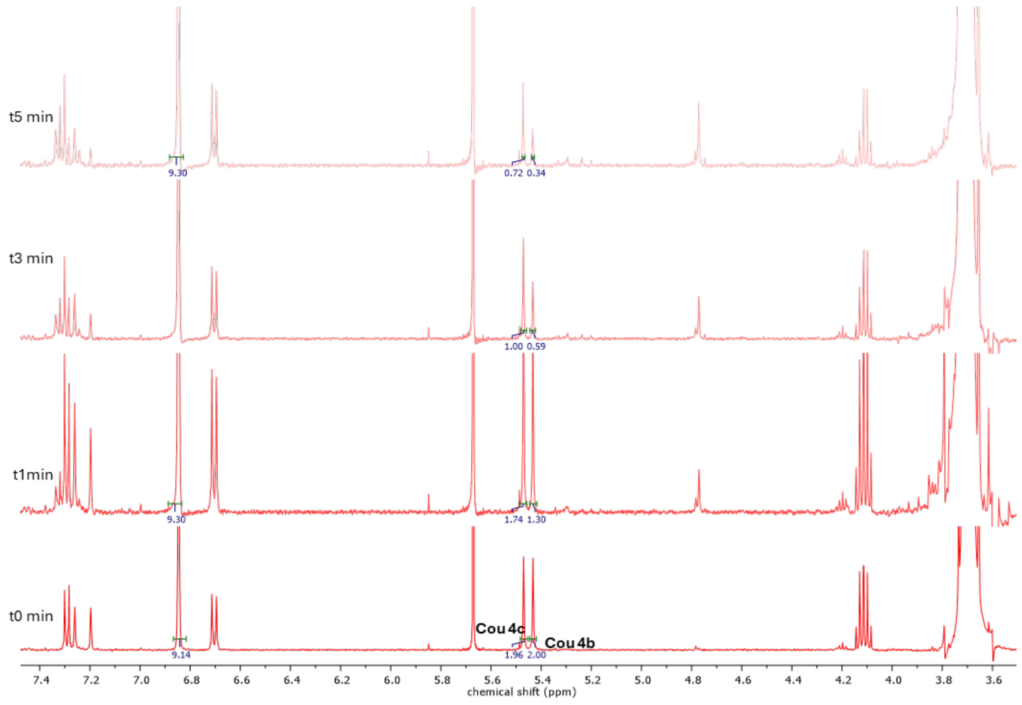
t5 min
t3 min
t1min
t0 min
Cou 4c
Cou 4b
chemical shift (ppm)

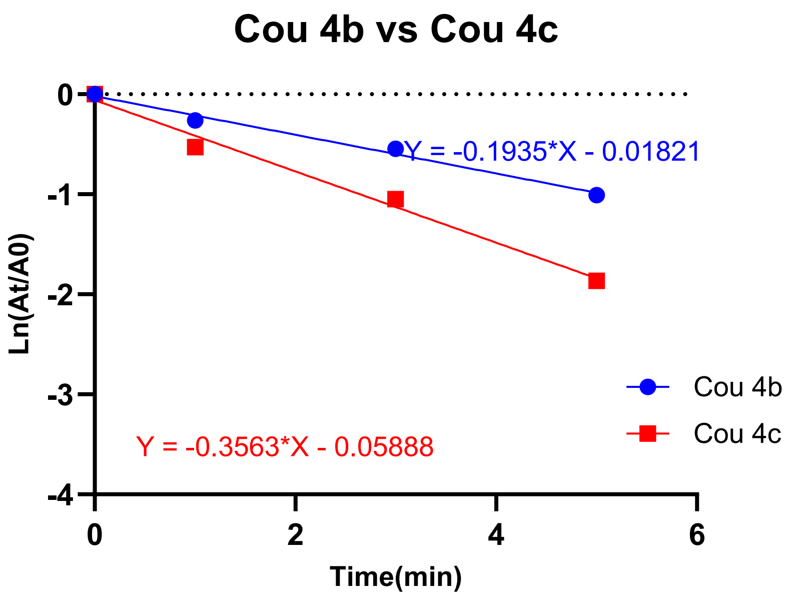
Cou 4b vs Cou 4c
Ln(At/A0)
Time(min)
Y = -0.1935*X - 0.01821
Y = -0.3563*X - 0.05888
Cou 4b
Cou 4c

5. Fluoresence emission spectra of Cou4a, 4b, 4c

Emission and excitation spectra of the compounds were recorded using a fluorescence spectrophotometer with a 1 cm path length quartz cuvette. Samples were prepared at a final concentration of 1 nmol in a DMSO/H_2O (9:1, v/v) solvent system. Emission spectra were collected by exciting samples at their respective excitation maxima, while excitation spectra were obtained by monitoring the emission at the corresponding emission maxima. All spectra were normalized to their respective maximum intensities (either emission or excitation peaks) using GraphPad Prism software for comparative analysis.

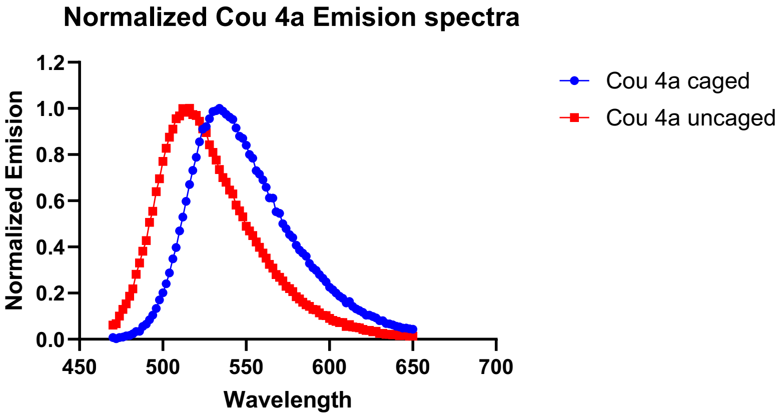

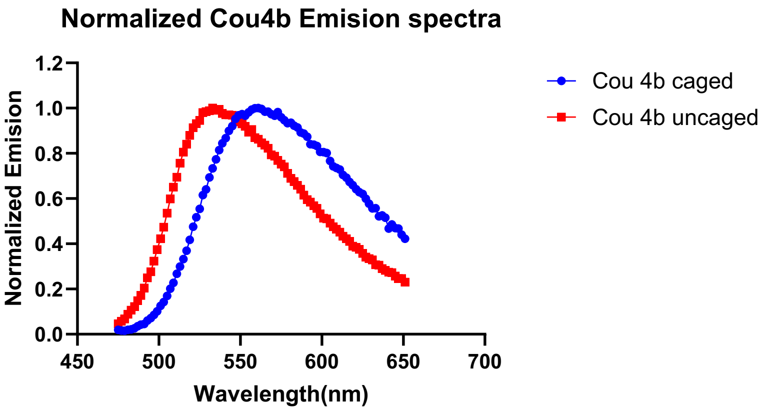

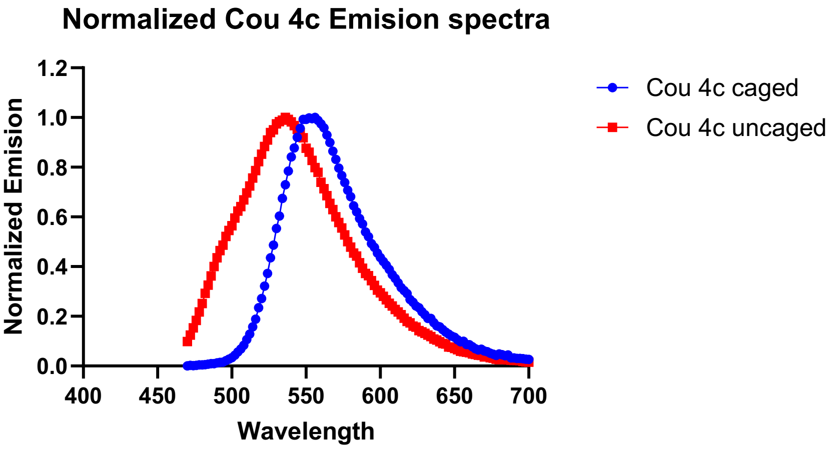

7.Reaction rate analysis and quantum yield calculation

The rates of reactions were derived from the quantification of the photolysis substrate (Cou 1c, 4b, 4d) using LC-MS integration with an internal standard (7-Diethylamino-4-methylcoumarin) at different time points following 488 nm irradiation (Oxxius LaserBoxx 488-50). The laser beam was spatially filtered by a pinhole and adjusted in size by a telescope system to form a circular spot with a diameter of 0.98 cm (the area of the beam was 0.75 cm^2). The photon flux was measured using a PM100A Powermeter (Thorlabs) with a calibrated S130C sensor (Thorlabs). A solution of test compound at a final concentration of 20μM in DMSO/H_2O with an equimolar concentration of standard was added to a 0.1 cm path-length cuvette for irradiation. Following irradiation, the solution was transfered to a LC-MS injection vial and protected from light with aluminum foil. The photodecomposition of the substrate was quantified by integrating its absorption peak (254nm) and normalizing it to the reference compound.

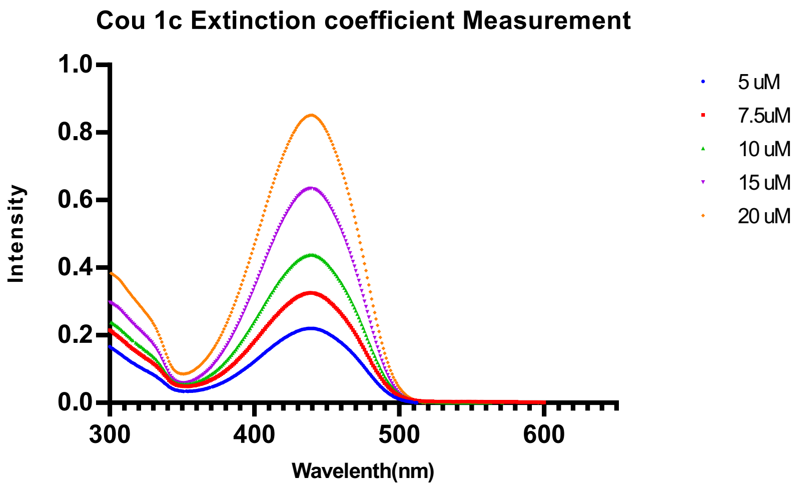

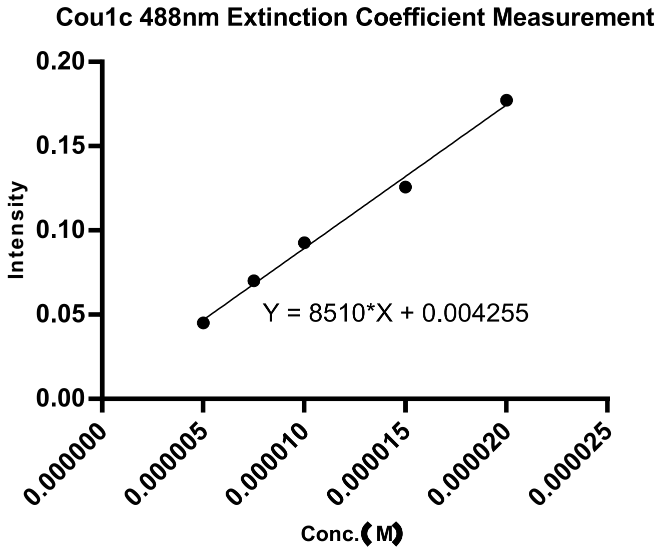

$$\varepsilon_{1c_{488nm}} = 0.851 \times 10^4 M^{-1} cm^{-1}$$

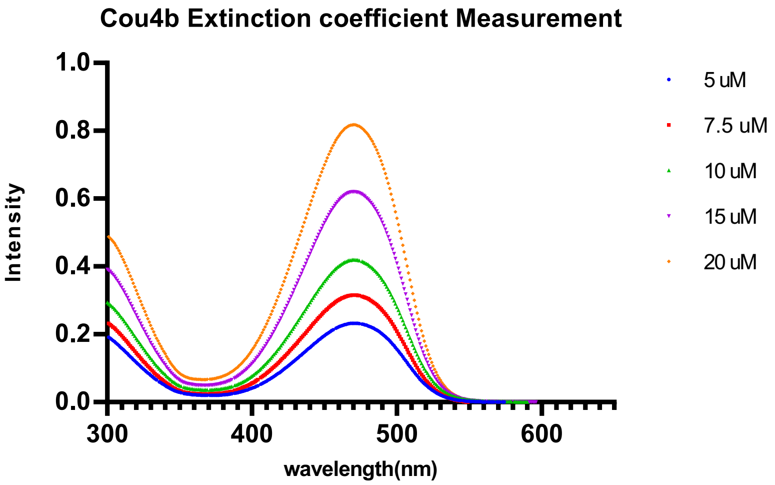

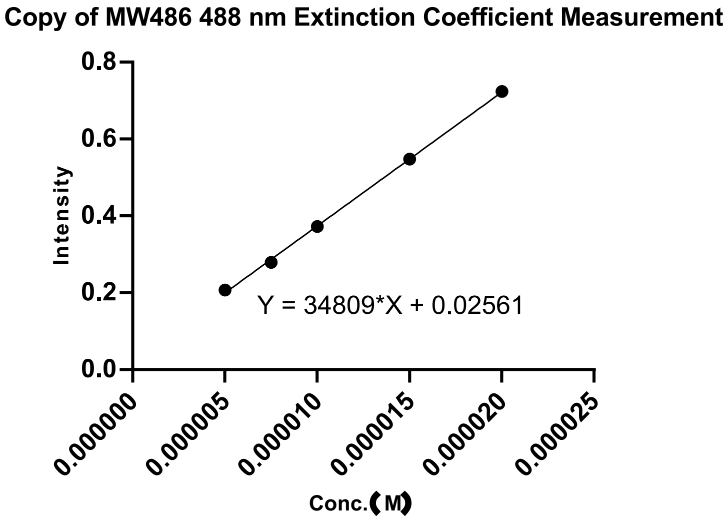

$$\varepsilon_{4b_{488nm}} = 3.4809 \times 10^4 M^{-1} cm^{-1}$$

Rate of uncaging for Cou 1c, 4b and 4d in DMEM and computation of quantum yield

As above, the rates of reactions were derived from the quantification of the photolysis substrate (**Cou 1a**, **1c**, **4b** and **4d**) using LC-MS integration with an internal standard (7-Diethylamino-4-methylcoumarin) at different time points following 488 nm irradiation (Oxxius LaserBoxx 488-50). The laser beam was spatially filtered by a pinhole and adjusted in size by a telescope system to form a circular spot with a diameter of 0.98 cm (the area of the beam was 0.75 cm^2). The photon flux was directly determined using a laser power meter. A solution of test compound at a final concentration of 1μM in 1:1 (v/v) DMEM/DMSO with an equimolar

concentration of standard was added to a 1 cm path-length cuvette for irradiation. Following irradiation, the solution was transfered to a LC-MS injection vial and protected from light with aluminum foil. The photodecomposition of the substrate was quantified by integrating its TIC trace and normalizing it to the reference compound. The quantum yield was calculated by according to previous reported procedure.[4] Briefly, the photodecomposition was fitted to a first order rate equation and the rate. The absolute quantum yield of photodecomposition was derived from the following formula: $\Phi_{dec} = k/I\sigma$ and $k = \ln(Ao/At)t^{-1}$.

I = photon flux

σ = decadic extinction coefficient in cm^2mol^{-1}

Φ_{dec} = absolute quantum yield of decomposition

simplified to $\Phi_{dec} = k/I\,\sigma$ where $\sigma = 1000\varepsilon$

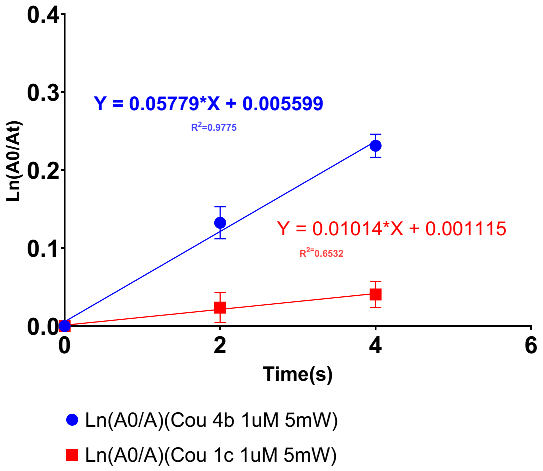

$k_{4b} = 0.0578\ s^{-1}$ $k_{1c} = 0.0101\ s^{-1}$

$$P = 5\ mW$$

$$E_{photon} = \frac{hc}{\lambda} = 4.07 \times 10^{-19}\ J$$

$$Photons\ per\ seconds:\ N_{photon} = \frac{P}{E_{photon}} = \frac{5\ mJs^{-1}}{4.07 \times 10^{-19} J/photon} = 1.23 \times 10^{16} s^{-1}$$

$$= 2.04 \times 10^{-8} ein\ s^{-1}$$

$$I = \frac{ein\ s^{-1}}{area} = 2.72\ \times 10^{-8}\ ein\ s^{-1}cm^{-2}$$

$$\varepsilon_{4b_{488nm}} = 3.4809 \times 10^4 M^{-1}cm^{-1}$$

$$\varepsilon_{1c_{488nm}} = 0.8510 \times 10^4 M^{-1}cm^{-1}$$

$$\Phi_{4b\ decompostion} = \frac{k}{I\varepsilon * 1000} = \frac{0.0578}{0.947} = 0.0610\ \text{mol/ein}$$

$$\Phi_{1C\ decomposition} = \frac{k}{I\varepsilon * 1000} = \frac{0.0101}{0.231} = 0.0437 mol/ein$$

LC–MS TIC peak area integration of Cou 1c in triplicate

Time	Ref	SM	Normalized Ref	Normalized SM	Normalized Conv	ln(A0/A)
0	5584	12089	1	12089	0	0
2	4879	10285	1.144497	11771.15	0.026293	0.026644
4	4782	9984	1.167712	11658.44	0.035616	0.036266

Time	Ref	SM	Normalized Ref	Normalized SM	Normalized Conv	ln(A0/A)
0	4868	8941	1	8941	0	0
2	4758	8387	1.023119	8580.899	0.040275	0.041109
4	4707	8151	1.034204	8429.8	0.057175	0.058874

Time	Ref	SM	Normalized Ref	Normalized SM	Normalized Conv	ln(A0/A)
0	4432	7998	1	7998	0	0
2	4000	7196	1.108	7973.168	0.003105	0.00311
4	4262	7490	1.039887	7788.756	0.026162	0.02651

LC–MS TIC peak area integration of Cou 4b in triplicate

Time(s)	Ref	SM	Normalized Ref	Normalized SM	Normalized Conv	ln(A0/A)
0	5428	13865	1	13865	0	0
2	5338	11988	1.01686	12190.12	0.120799	0.128742
4	5299	10266	1.024344	10515.92	0.241549	0.276478

Time(s)	Ref	SM	Normalized Ref	Normalized SM	Normalized Conv	ln(A0/A)
0	5235	14611	1	14611	0	0
2	5164	12163	1.013749	12330.23	0.1561	0.169721
4	5171	11002	1.012377	11138.17	0.237686	0.271397

Time(s)	Ref	SM	Normalized Ref	Normalized SM	Normalized Conv	ln(A0/A)
0	3232	7887	1	7887	0	0
2	4546	9837	0.710955	6993.661	0.113267	0.120212
4	1521	2996	2.124918	6366.254	0.192817	0.214205

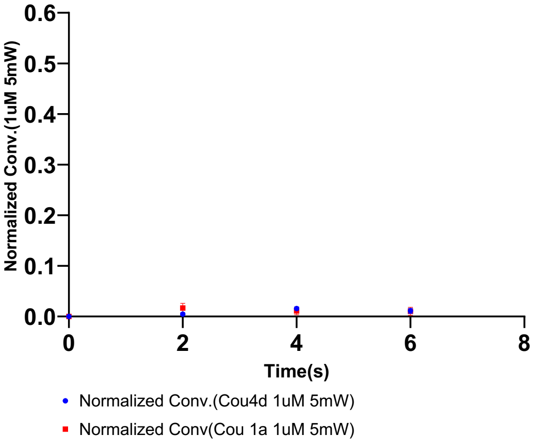

For **Cou1a** and **Cou 4d**, the rate of photolysis observed under the conditions used of **Cou1c** and **4b** (488 nm irradiation, 5 mW) did not yield enough photolysis to measure an accurate rate, the reaction yield is negligible.

LC–MS TIC peak area integration of Cou 1a in triplicate

Time(s)	Ref	SM	Normalized Ref	Normalized SM	Conv
0	11732	18753	1	18753	0
2	11789	18348	0.995165	18259.29	0.026327
4	11619	18291	1.009725	18468.89	0.01515
6	11875	18649	0.987958	18424.43	0.017521

Time(s)	Ref	SM	Normalized Ref	Normalized SM	Conv
0	11340	18839	1	18839	0
2	11122	18178	1.019601	18534.3	0.016174
4	10907	17845	1.039699	18553.43	0.015158
6	10307	17091	1.100223	18803.91	0.001862

Time(s)	Ref	SM	Normalized Ref	Normalized SM	Conv
0	9938	16861	1	16861	0
2	9821	16532	1.011913	16728.95	0.007832
4	10314	17459	0.963545	16822.53	0.002282
6	10019	16806	0.991915	16670.13	0.01132

LC–MS TIC peak area integration of Cou 4d in triplicate

Time(s)	Ref	SM	Normalized Ref	Normalized SM	Conv

0	7072	12726	1	12726	0
2	6930	12438	1.020491	12692.86	0.002604
4	7054	12472	1.002552	12503.83	0.017458
6	6823	12093	1.036494	12534.32	0.015062

Time(s)	Ref	SM	Normalized Ref	Normalized SM	Conv
0	6855	12478	1	12478	0
2	6827	12375	1.004101	12425.75	0.004187
4	6839	12225	1.00234	12253.6	0.017984
6	6962	12508	0.984631	12315.76	0.013002

Time(s)	Ref	SM	Normalized Ref	Normalized SM	Conv
0	6768	12064	1	12064	0
2	6721	11890	1.006993	11973.15	0.007531
4	6759	11903	1.001332	11918.85	0.012032
6	6307	11182	1.073093	11999.33	0.005361

cBiotin uncaging rate in media

Workflow:

A stock of cBiotin 50 uM in DMSO was diluted to 500nM of PBS 10mM (2000uL) [0.002L* 500nM=2nmol] and 20 equiv. streptavidin High-capacity beads(410nmol/mL) [40nmol] about 100uL were added into the solution, and incubated in the dark at 25 °C for 1 hour. Then the solution was centrifuged and PBS was removed and washed with PBS twice. DMEM media with 10% FBS was added (2mL followed by mixing the beads to make the suspension). The suspension was separated into 6 Eppendorf (150uL of each) which were irradiated with 488 nm laser (5 mW/cm^2) are for 0s 2s 4s 6s 8s 10s, and 30 min. Then the samples were centrifuged after irradiation. The fluorescent intensity of the supernatant was measured by the Tecan plate reader (wavelength between 500nm-510nm). The PBS before washing can be read as the negative control. The value recorded for media alone was used as blank. The extent of uncaging was determined by normalizing the fluorescence increase between t_0 and the fully uncaged state, and the resulting values were used to quantify the conversion of the starting material.

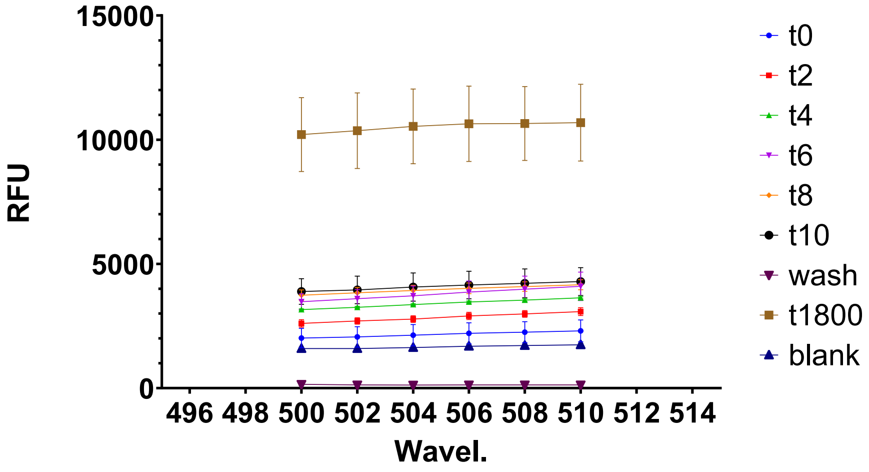

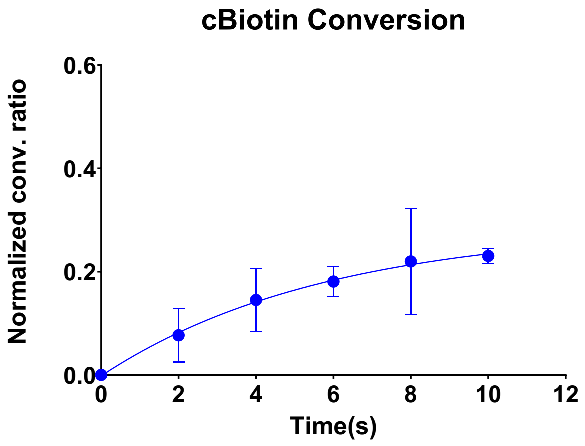

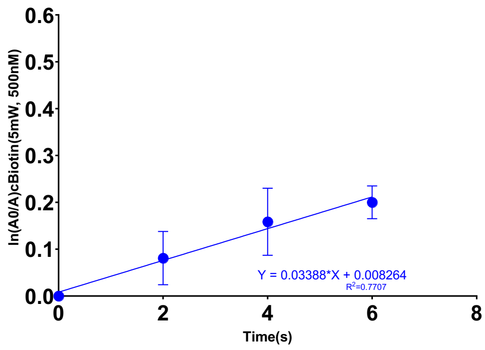

For cBiotin 488nm laser 5mW, $k_{\mathrm{cBiotin}} = 0.0339\ \mathrm{s}^{-1}$

$$\Phi_{4cBiotin} = \frac{0.0339}{0.947} = 0.0358$$

8. Biological activty

Cell culture

HeLa cells were obtained from the American Type Culture Collection (ATCC) and grown at 37 °C in a humidified incubator under constant 5% CO_2 concentration using Glutamax DMEM (Gibco - 10566016) supplemented with 10% FCS, 100 U/mL penicillin and 100 mg/mL streptomycin. Stably expressing EB3-dTomto HeLa cells were a kind gift from Prof. Patrick Meraldi (University of Geneva, Switzerland). Cells were grown in Glutamax DMEM supplemented with 10% FCS, 100 U/mL penicillin, 100 mg/mL streptomycin, 0.3 µg/mL puromycin, and 500 µg/mL G418 at 37 °C under 5% CO_2 concentration in a humidified incubator. Cells were regularly tested for mycoplasma contamination by qPCR using the MycoplasmaCheck test service (Eurofins). Colonies were kept in culture for no more than 15 passages.

Cytotoxicity

HeLa cells were seeded at a concentration of 5000 cells/mL in a 96-wells clear bottom plate and incubated. The following day, two 96-well plates were treated with a dilution series (1 mM to 1 pM) of cMMAF or caged control cCTL (caged butanoic acid in lieu of MMAF) for 2 hours. One plate was irradiated for 10 minutes using a 455 nm LED lamp (Irradiance 0.82 mW/cm^2 – Radiant exposure 0.49 J/cm^2), while the other plate was kept in the dark. Once treated, the cells were washed 3 times with 150 µL of warm media making sure to wait 10 minutes between washes and returned to the incubator for 48 hours. Before imaging, Hoechst 33342 (Chemodex) was added to the cells to a final concentration of 2 µg/mL for 30 minutes and washed 3 times with 150 mL of warm media to remove death and dying cells. After that, cells were imaged using a Molecular DevicesTM ImageXpress Micro (IXM) XL automated microscope with DAPI settings. Excitation = 100 ms. Objective 10×. The temperature was maintained constantly at 37 °C and CO_2 was kept at 5%. The number of healthy cells was quantified by using nuclei count protocol where the particle mask size was width = 10 µm; height = 25 µm; fluorescence cutoff threshold = 1000. Each well was analyzed by the acquisition of a matrix of images covering the well (25 tiles). Images were analyzed using the MetaXpress® software, and the data were plotted using GraphPad.

Single cell uncaging

EB3-dTomato HeLa cells were seeded at a concentration of 15’000 cells/mL in a 18-well slide (Ibidi – 81816). The following day cells were imaged using a Leica Stellaris 8 FALCON confocal microscope to find a suitable group of cells showing a satisfactory network of EB3-dTomato. Following the identification of a suitable group of cells, a ROI was drawn restricting the uncaging to one cell or part of it. Right before laser irradiation, the media was exchanged with Fluorobrite DMEM supplemented with 50 nM of cMMAF or caged control cCTL and the cells were quickly imaged using FRAP settings to facilitate the acquisition. The uncaging was performed by using a white light laser and scanning several times the ROI using the following parameters: wavelength 488 nm, laser intensity 4%, laser power 3.5 µW, resolution 1024 x 1024 pixels, pixel dwell time 4.125 µs, pixel size 180 nm^2, line averaging 2, scanning time 100 Hz, objective 63x

oil 1.4 NA, zoom 1. To detect EBR-dTomato comets cells were irradiated before, during and after uncaging with a white light laser (l_{em} 570 $l_{detected}$ = 585 to 670 nm). Knowing all the above, the irradiance per pixel is easily calculated as:

$$E = \frac{3.5 \cdot 10^{-6}\ W}{1.8 \cdot 10^{-10}\ cm^2} = 1.9\ \cdot 10^4\ W/cm^2$$

Knowing that each pixel is irradiated for exactly 4.125 ms and that each scan does an averaging between lines, the fluence per scan can then be derived as

$$F = 2 \cdot E \cdot t = 2 \cdot 1.9\ \cdot 10^4 \frac{W}{cm^2} \cdot 4.125 \cdot 10^{-6} = 0.16 \frac{J}{cm^2}$$

Multiphoton laser uncaging

EB3-dTomato HeLa cells were seeded at a concentration of 75’000 cells/mL in a 3.5 cm glass-bottom dish with a 10 mm microwell (Mattek). After one day cells were incubated with 50 µM cMMAF. To trigger the isomerization, the dishes were irradiated using a multiphoton laser at 976 nm and real-time images were acquired using FRAP settings. For the uncaging a ROI corresponding to a group of cells was used to trigger uncaging using: 4% laser power (4 mW - measured with a PM100USB - Thorlabs), 1.4x zoom, 100 Hz scanning speed, 1.4 NA, pixel size 93 nm^2, pixel dwell time 4.125 ms, 2x line averaging, 2x frame accumulation, 1024x1024 pixels.

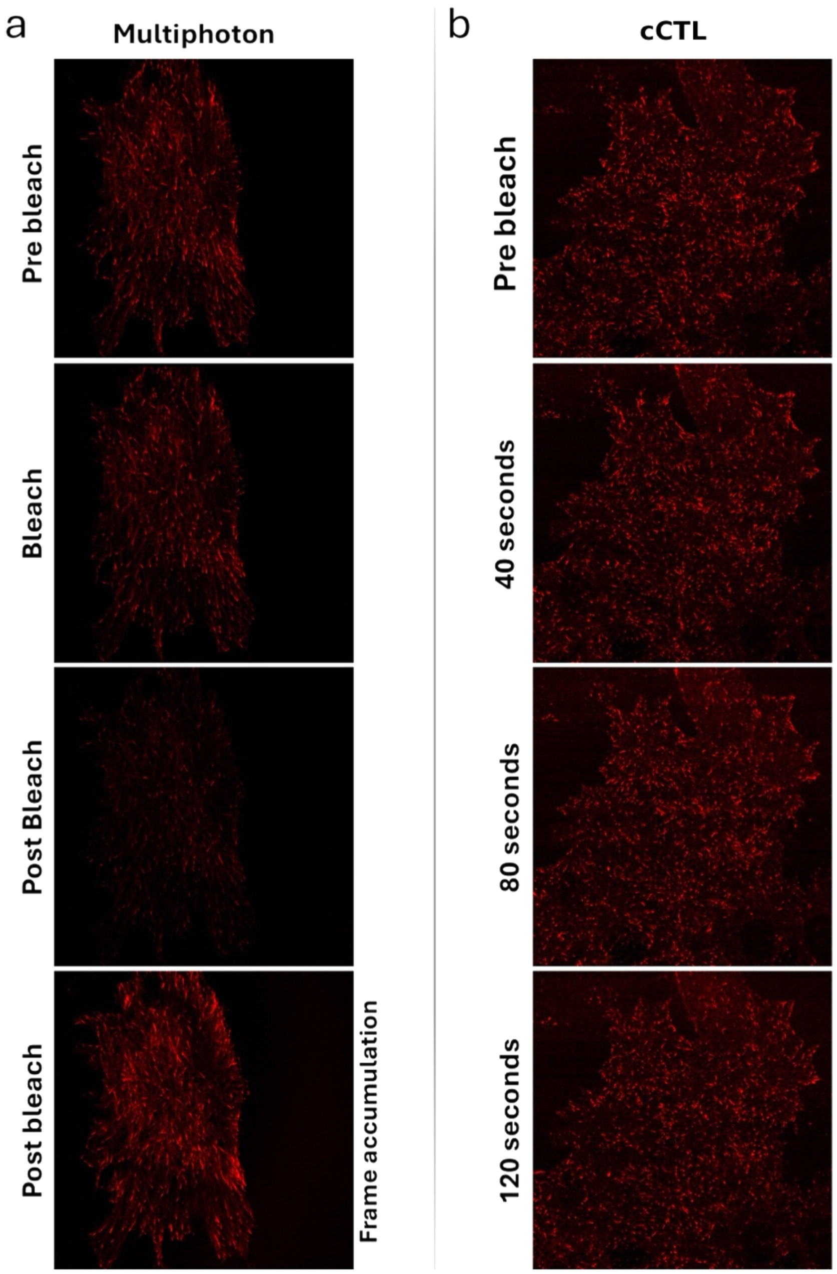

Figure S4 shows the effects of either multiphoton irradiation or caged control (**cCTL**: caged butanoic acid instead of MMAF) on dTomato-EB3 network. **a.** A group of dTomato-EB3 expressing cells is irradiated using a multiphoton laser as described above. While an overall loss of fluorescence intensity (photobleaching) can be observed in the post bleach image, the microtubular is not disturbed, as shown in the second post bleaching image obtained by accumulating the same frame twice to increase the signal. **b.** Cells treated with control **cCTL** are irradiated at 488 nm for up to 120 seconds. The microtubule dynamics is not altered by the irradiation alone or the photolysis byproducts of **cCTL**.

9. Stability of cMMAF in media

A 2 mM stock solution of cMMAF was diluted in FluoroBrite medium to a final concentration of 2 µM. Samples were prepared in triplicate in black Eppendorf tubes in medium supplemented with 10% fetal calf serum (FCS) and incubated for 12 h.

Following incubation, a control peptide of known concentration, H-VSGWRLFKKIS-NH_2, was added to each sample as an internal reference for normalization in the quantification. Proteins were precipitated by addition of an equal volume of ice-cold acetonitrile/ethanol (1:1, v/v), and the samples were kept cold for 12 h. The mixtures were then centrifuged for 10 min at 4 °C at 7000 rpm. The resulting supernatants were filtered prior to LC–HRMS analysis.

LC–HRMS analyses were performed on a Xevo G2 TOF instrument operated in positive ion mode using a PINNACLE DB C18 column (1.9 µm, 50 × 2.1 mm). Elution was carried out using a linear gradient from 5% to 95% acetonitrile in water containing 0.1% formic acid over 10 min. Under these conditions, the control peptide was detected at a retention time of 3.24 min, with an expected m/z of 1319.7946 ($[M+H]^+$) and an observed m/z of 1319.7948. cMMAF was detected at a retention time of 7.21 min, with an expected m/z of 1128.67xx ($[M+H]^+$) and an observed m/z of 1128.6698. Quantification was performed by normalization to the internal peptide standard.

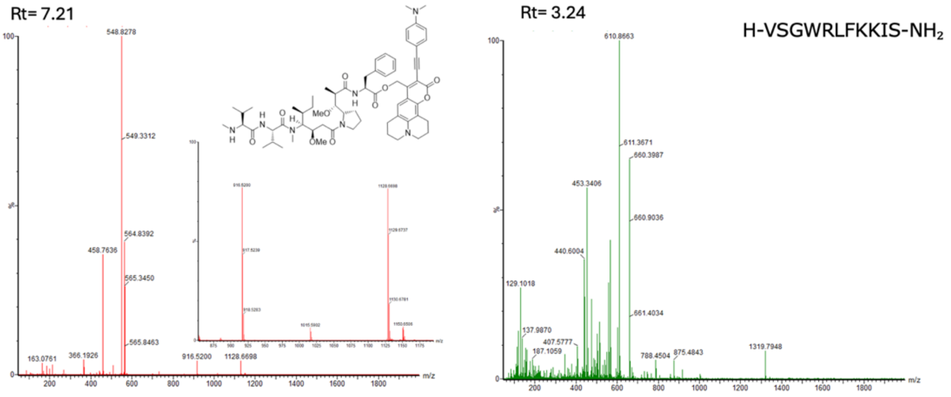

Relative cMMAF concentration was determined from the average integrated signal of the triplicate samples.

Under these conditions, cMMAF exhibited good stability with 86% integrity over 12h.

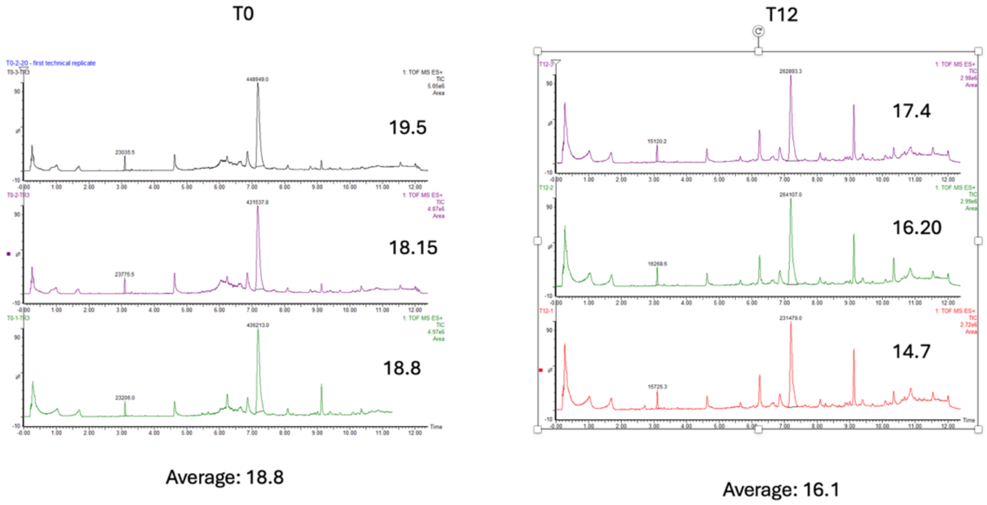

14% over 12 hours

10.Reference

(1) Lin, W.; Long, L.; Feng, J.; Wang, B.; Guo, C. Synthesis of *Meso* -Coumarin-Conjugated Porphyrins and Investigation of Their Luminescence Properties. *Eur. J. Org. Chem.* **2007**, *2007* (26), 4301–4304. https://doi.org/10.1002/ejoc.200700475.

(2) Bojtár, M.; Németh, K.; Domahidy, F.; Knorr, G.; Verkman, A.; Kállay, M.; Kele, P. Conditionally Activatable Visible-Light Photocages. *J. Am. Chem. Soc.* **2020**, *142* (35), 15164–15171. https://doi.org/10.1021/jacs.0c07508.

(3) Lin, Q.; Yang, L.; Wang, Z.; Hua, Y.; Zhang, D.; Bao, B.; Bao, C.; Gong, X.; Zhu, L. Coumarin Photocaging Groups Modified with an Electron-Rich Styryl Moiety at the 3-Position: Long-Wavelength Excitation, Rapid Photolysis, and Photobleaching. *Angew. Chem. Int. Ed.* **2018**, *57* (14), 3722–3726. https://doi.org/10.1002/anie.201800713.

(4) Banala, S.; Jin, X.-T.; Dilan, T. L.; Sheu, S.-H.; Clapham, D. E.; Drenan, R. M.; Lavis, L. D. Elucidating and Optimizing the Photochemical Mechanism of Coumarin-Caged Tertiary Amines. *J. Am. Chem. Soc.* **2024**, *146* (30), 20627–20635. https://doi.org/10.1021/jacs.4c03092.

11. Annex: NMR spectra

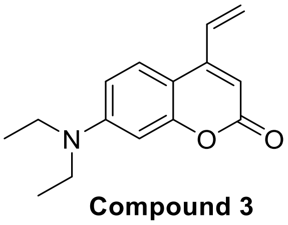
N
O
O

Compound 3

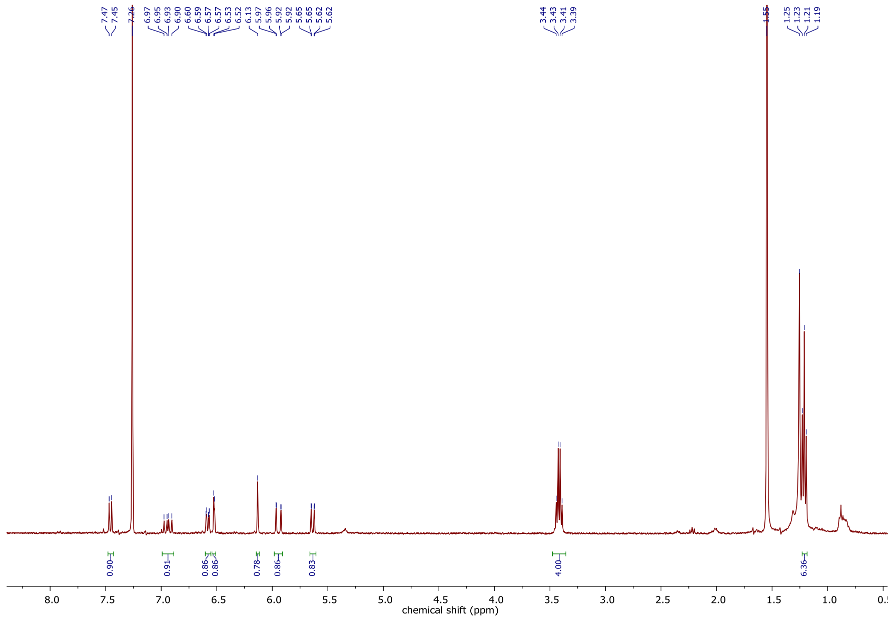
7.47
7.45
7.26
6.97
6.95
6.93
6.90
6.60
6.59
6.57
6.57
6.53
6.52
6.13
5.97
5.96
5.92
5.92
5.65
5.65
5.62
5.62
3.44
3.43
3.41
3.39
1.56
1.25
1.23
1.21
1.19
0.90
0.91
0.86
0.86
0.78
0.86
0.83
4.00
6.36
8.0
7.5
7.0
6.5
6.0
5.5
5.0
4.5
4.0
3.5
3.0
2.5
2.0
1.5
1.0
chemical shift (ppm)

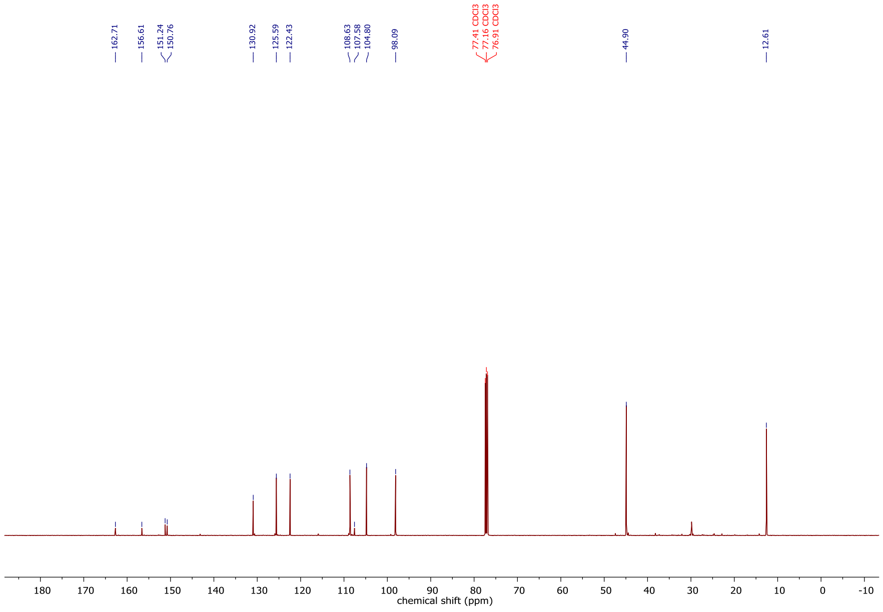
162.71
156.61
151.24
150.76
130.92
125.59
122.43
108.63
107.58
104.80
98.09
77.41 CDCl3
77.16 CDCl3
76.91 CDCl3
44.90
12.61
180
170
160
150
140
130
120
110
100
90
80
70
60
50
40
30
20
10
0
-10
chemical shift (ppm)

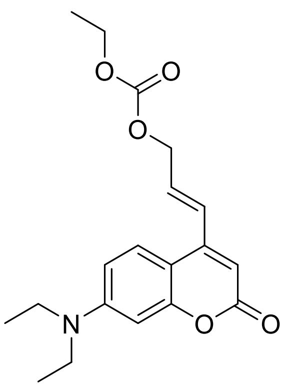

Cou 2a

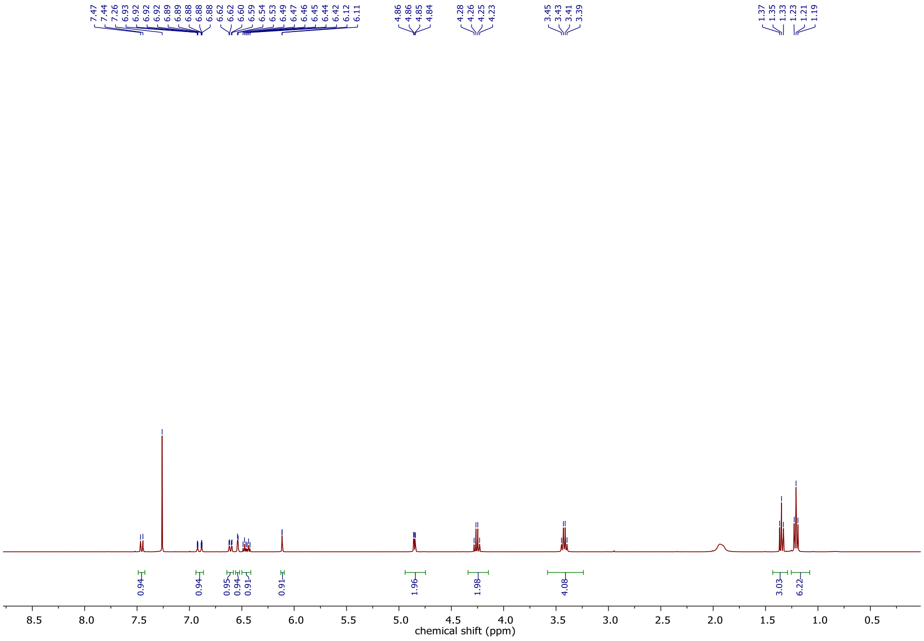
7.47 7.44 7.26 6.93 6.92 6.92 6.92 6.89 6.89 6.89 6.88 6.88 6.88 6.62 6.62 6.60 6.59 6.54 6.53 6.49 6.47 6.46 6.45 6.44 6.42 6.12 6.11
4.86 4.86 4.85 4.84
4.28 4.26 4.25 4.23
3.45 3.43 3.41 3.39
1.37 1.35 1.33 1.23 1.21 1.19
0.94 0.94 0.95 0.94 0.91 0.91 1.96 1.98 4.08 3.03 6.22
8.5 8.0 7.5 7.0 6.5 6.0 5.5 5.0 4.5 4.0 3.5 3.0 2.5 2.0 1.5 1.0 0.5
chemical shift (ppm)

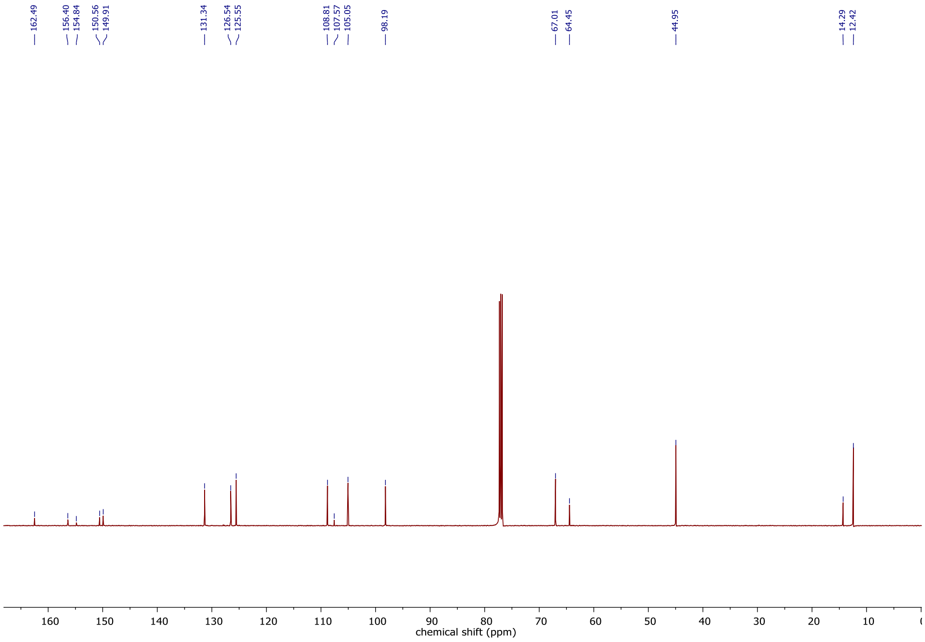
162.49
156.40 154.84
150.56 149.91
131.34
126.54 125.55
108.81 107.57 105.05
98.19
67.01 64.45
44.95
14.29 12.42
160 150 140 130 120 110 100 90 80 70 60 50 40 30 20 10
chemical shift (ppm)

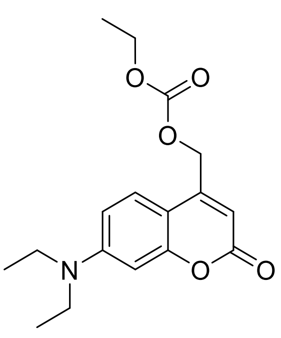

Cou 1a

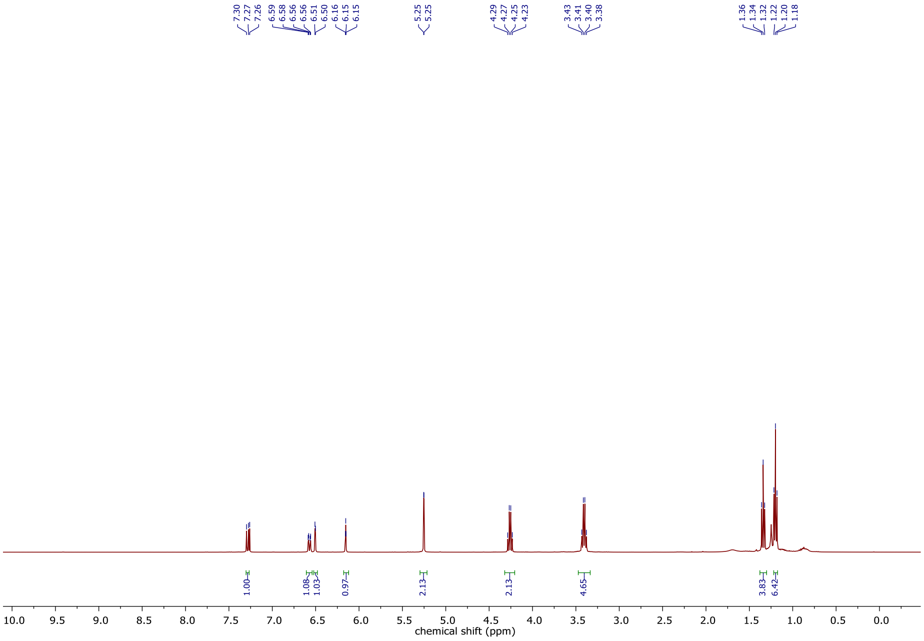
7.30
7.27
7.26
6.59
6.58
6.56
6.56
6.51
6.50
6.16
6.15
6.15
5.25
5.25
4.29
4.27
4.25
4.23
3.43
3.41
3.40
3.38
1.36
1.34
1.32
1.22
1.20
1.18
1.00
1.08
1.03
0.97
2.13
2.13
4.65
3.83
6.42
10.0 9.5 9.0 8.5 8.0 7.5 7.0 6.5 6.0 5.5 5.0 4.5 4.0 3.5 3.0 2.5 2.0 1.5 1.0 0.5 0.0
chemical shift (ppm)

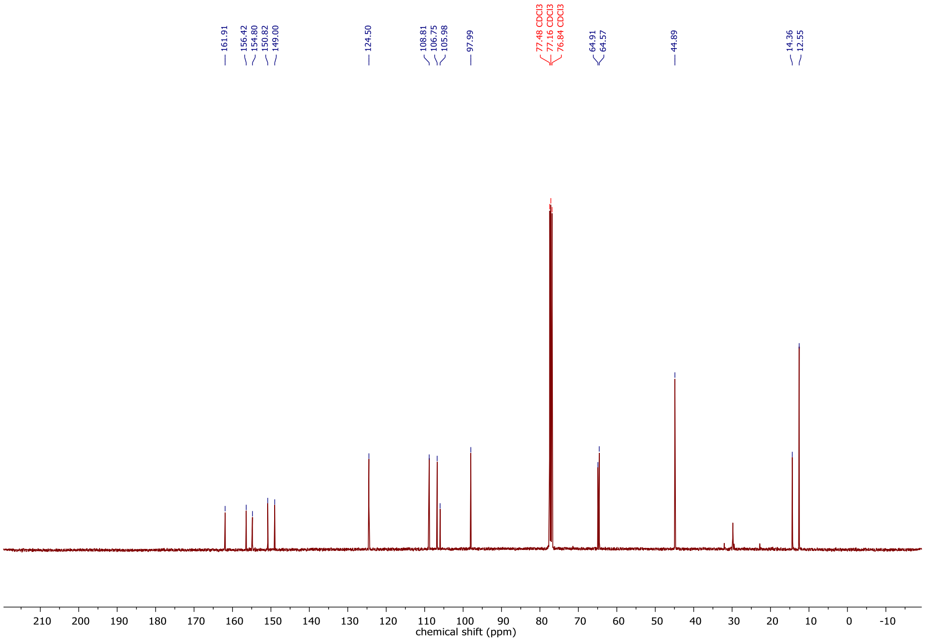
161.91
156.42
154.80
150.82
149.00
124.50
108.81
106.75
105.98
97.99
77.48 CDCl3
77.16 CDCl3
76.84 CDCl3
64.91
64.57
44.89
14.36
12.55
210 200 190 180 170 160 150 140 130 120 110 100 90 80 70 60 50 40 30 20 10 0 -10
chemical shift (ppm)

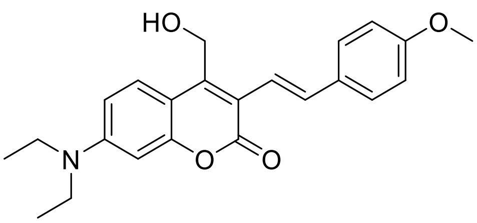
HO
N
O
O
O

Compound 6

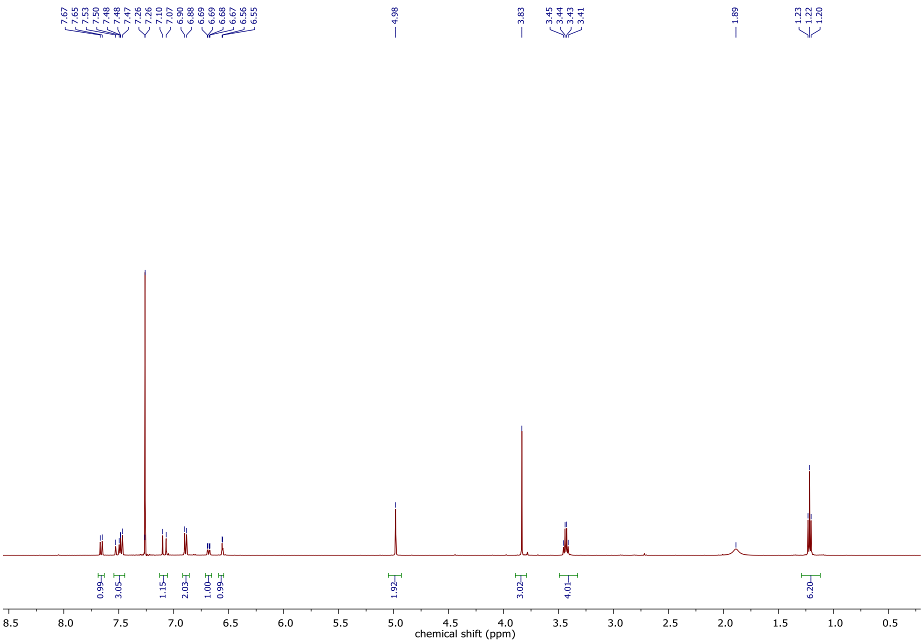
7.67 7.65 7.53 7.50 7.48 7.48 7.47 7.26 7.26 7.10 7.07 6.90 6.88 6.69 6.69 6.68 6.67 6.56 6.55
4.98
3.83
3.45 3.44 3.43 3.41
1.89
1.23 1.22 1.20
0.99 3.05 1.15 2.03 1.00 0.99 1.92 3.02 4.01 6.20
8.5 8.0 7.5 7.0 6.5 6.0 5.5 5.0 4.5 4.0 3.5 3.0 2.5 2.0 1.5 1.0 0.5
chemical shift (ppm)

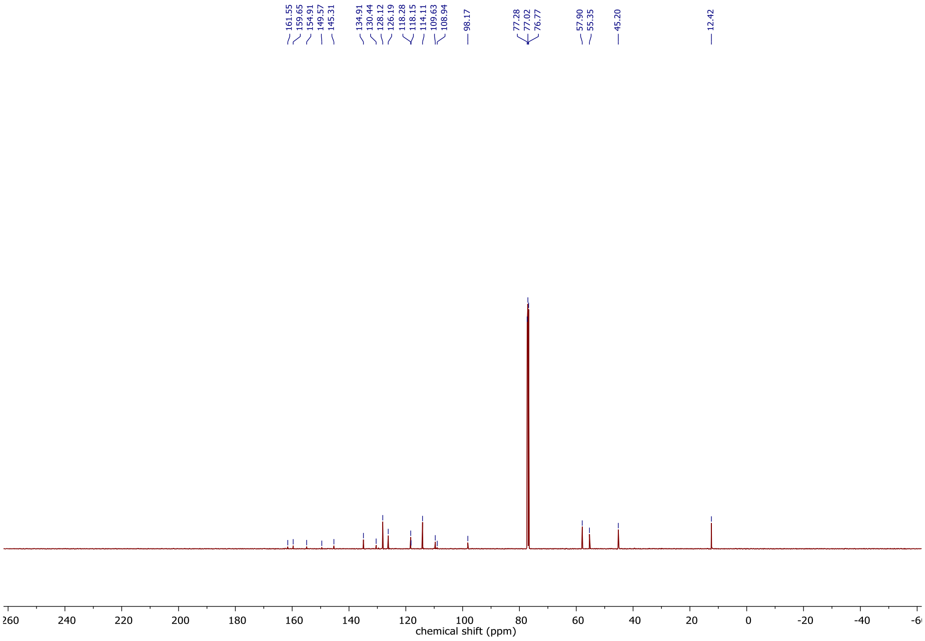
161.55 159.65 154.91 149.57 145.31
134.91 130.44 128.12 126.19 118.28 118.15 114.11 109.63 108.94
98.17
77.28 77.02 76.77
57.90 55.35
45.20
12.42
260 240 220 200 180 160 140 120 100 80 60 40 20 0 -20 -40
chemical shift (ppm)

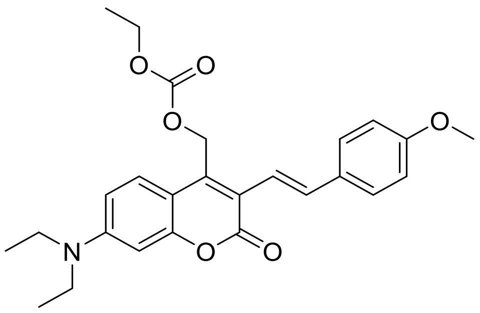

Cou 1c

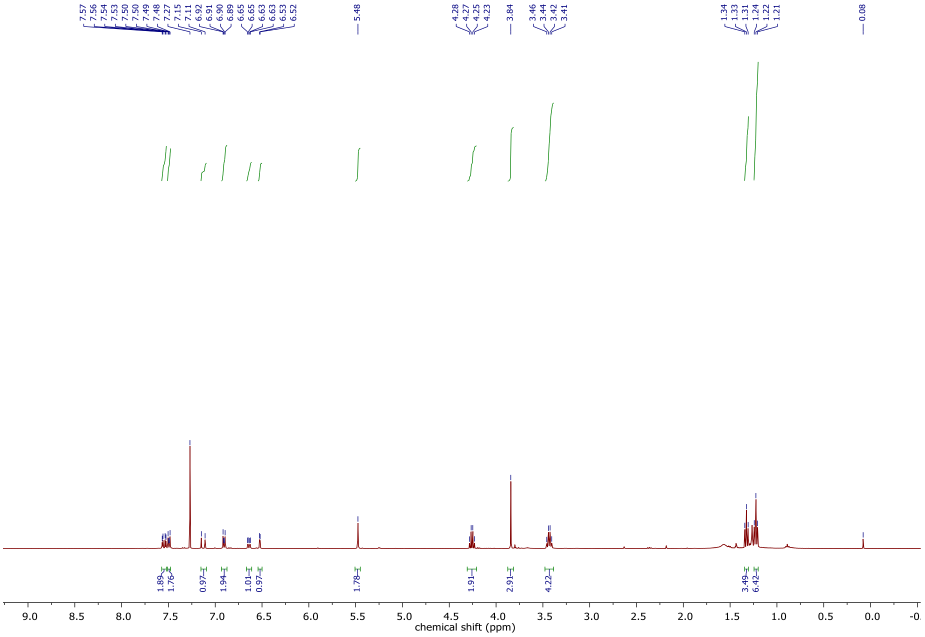

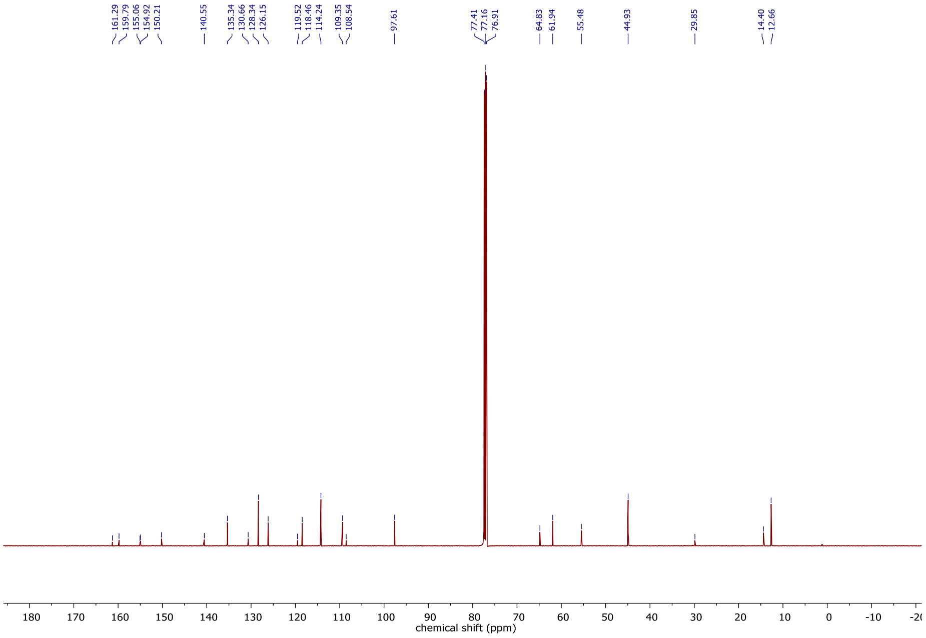

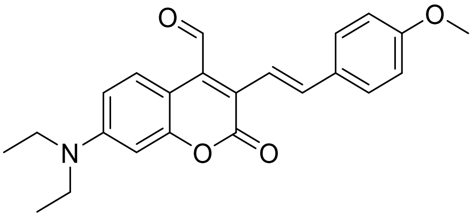

Compound 7

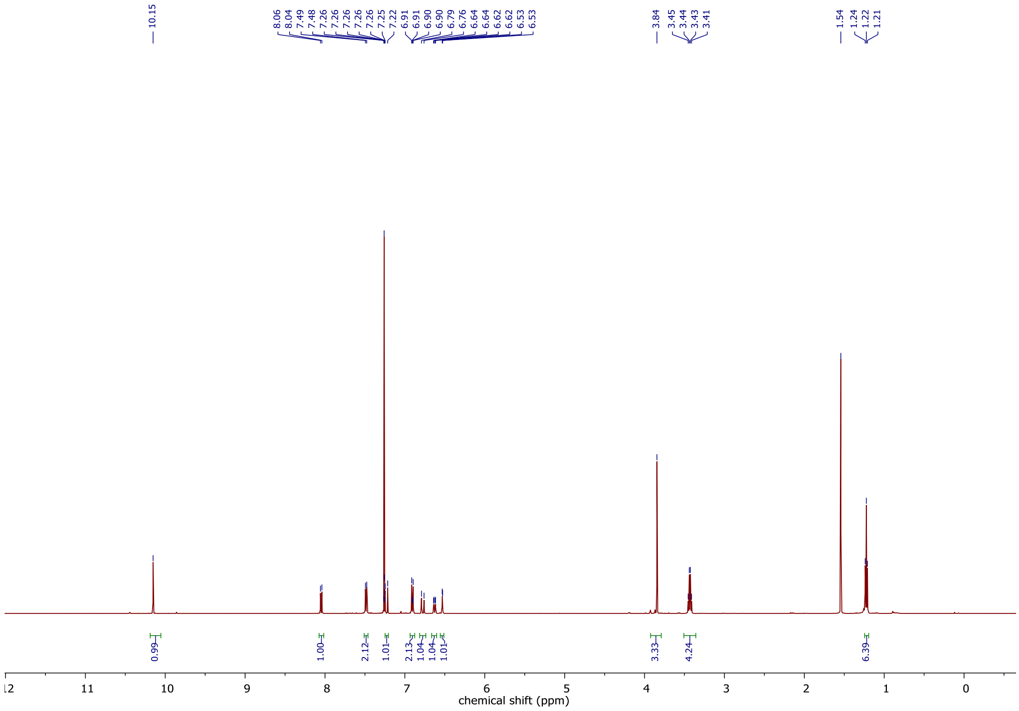

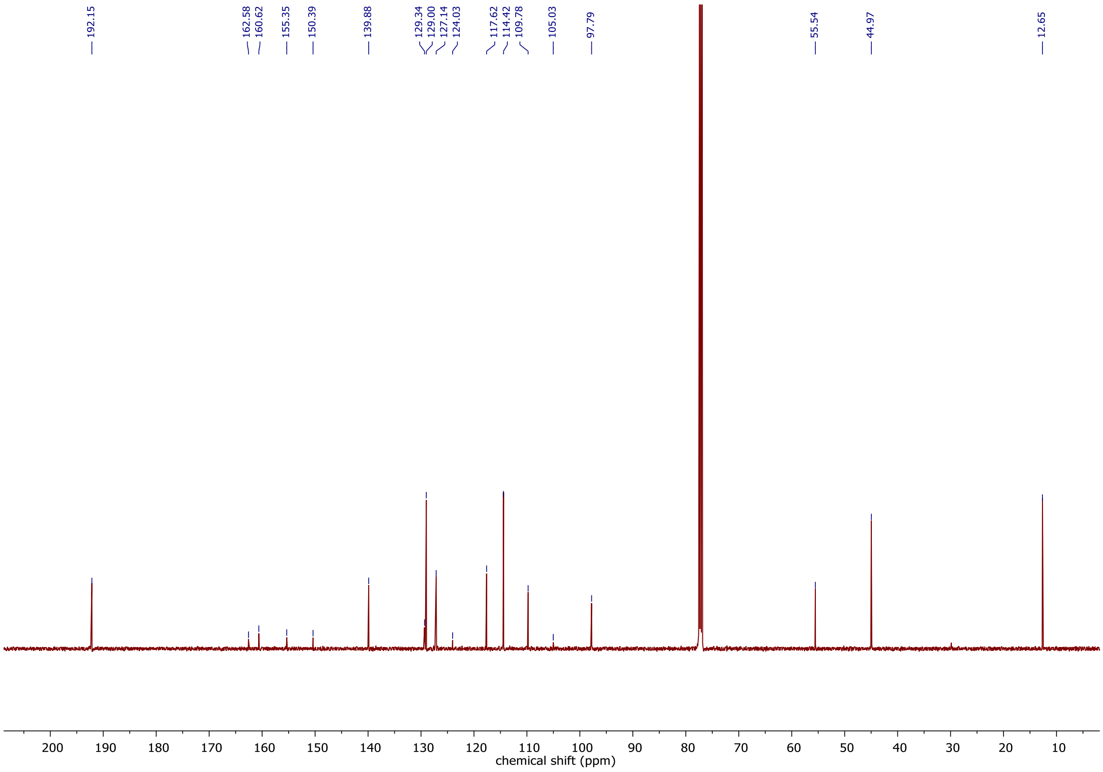

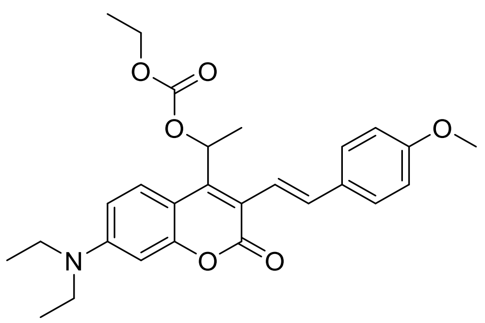

Cou 3a

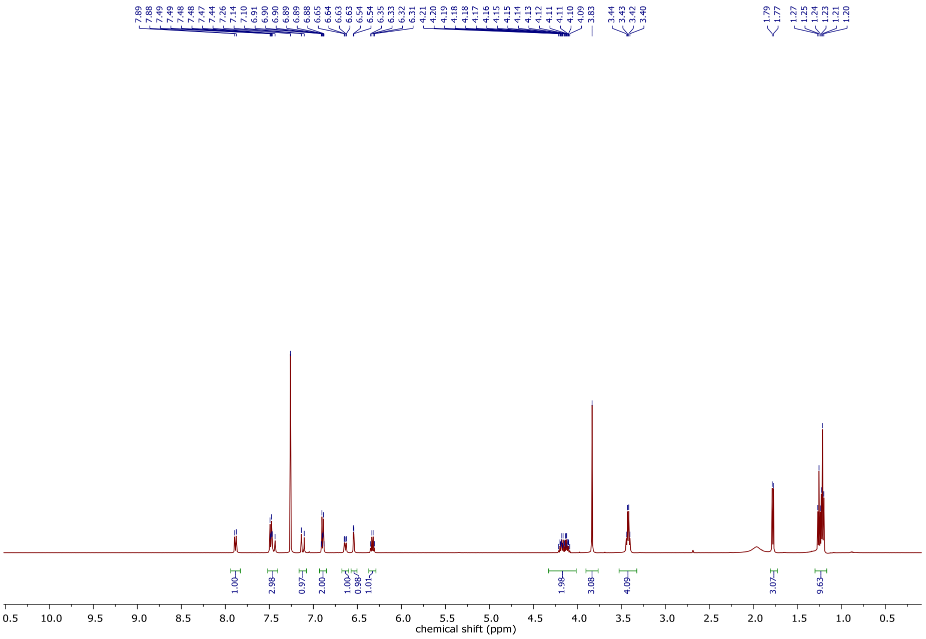
7.89 7.88 7.49 7.49 7.48 7.48 7.47 7.44 7.26 7.14 7.10 6.91 6.90 6.90 6.89 6.89 6.88 6.65 6.64 6.63 6.63 6.54 6.54 6.35 6.33 6.32 6.31 4.21 4.20 4.19 4.18 4.18 4.17 4.16 4.15 4.15 4.14 4.13 4.12 4.11 4.11 4.10 4.09 3.83 3.44 3.43 3.42 3.40 1.79 1.77 1.27 1.25 1.24 1.23 1.21 1.20
1.00 2.98 0.97 2.00 1.00 0.98 1.01 1.98 3.08 4.09 3.07 9.63
0.5 10.0 9.5 9.0 8.5 8.0 7.5 7.0 6.5 6.0 5.5 5.0 4.5 4.0 3.5 3.0 2.5 2.0 1.5 1.0 0.5
chemical shift (ppm)

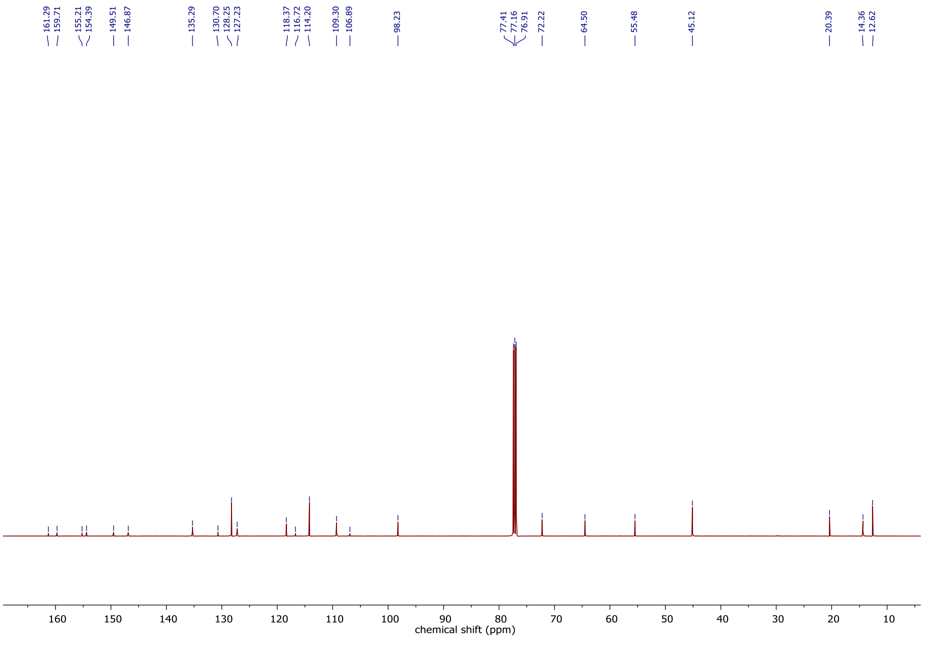
161.29 159.71 155.21 154.39 149.51 146.87 135.29 130.70 128.25 127.23 118.37 116.72 114.20 109.30 106.89 98.23 77.41 77.16 76.91 72.22 64.50 55.48 45.12 20.39 14.36 12.62
160 150 140 130 120 110 100 90 80 70 60 50 40 30 20 10
chemical shift (ppm)

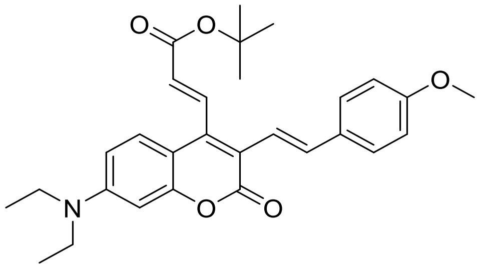

Compound 8

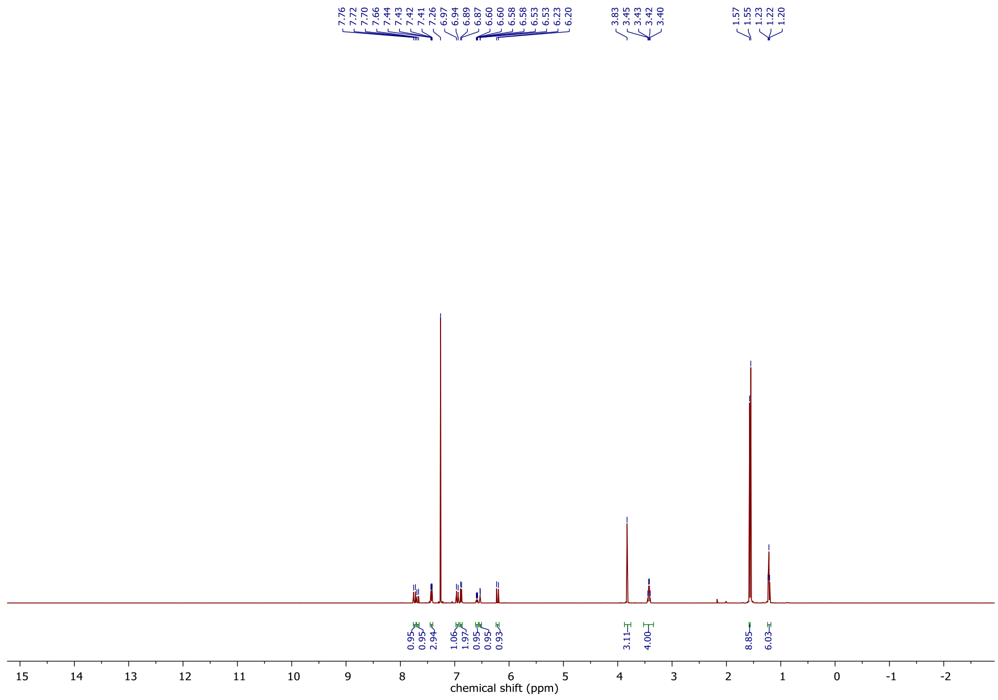

7.76 7.72 7.70 7.66 7.44 7.43 7.42 7.41 7.26 6.97 6.94 6.89 6.87 6.60 6.60 6.58 6.58 6.53 6.53 6.23 6.20
3.83 3.45 3.43 3.42 3.40
1.57 1.55 1.23 1.22 1.20
0.95 0.95 2.94 1.06 1.97 0.95 0.95 0.93 3.11 4.00 8.85 6.03
15 14 13 12 11 10 9 8 7 6 5 4 3 2 1 0 -1 -2
chemical shift (ppm)

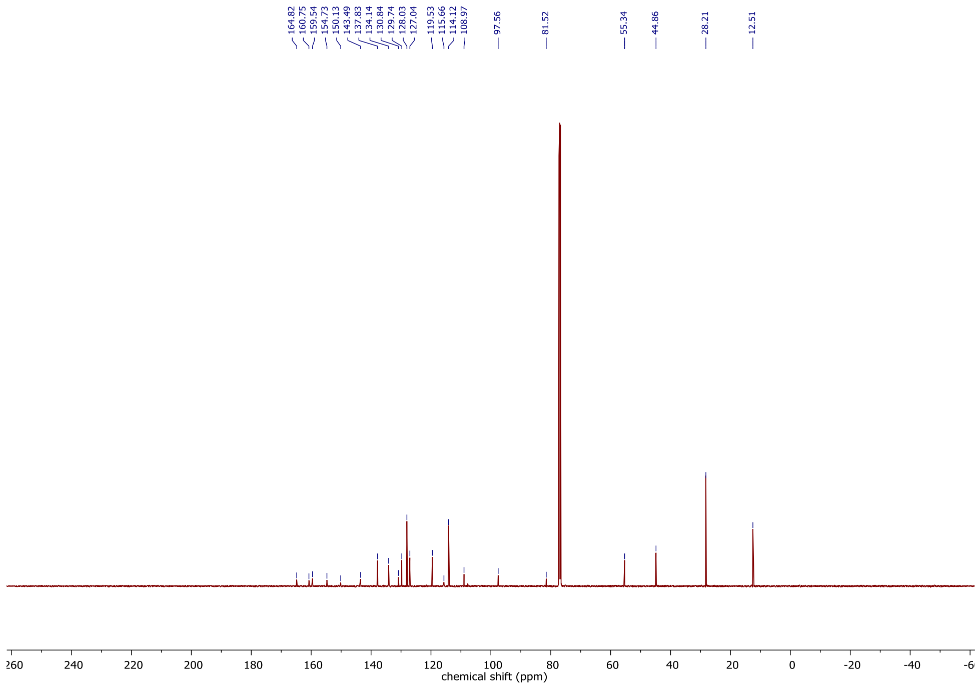

164.82 160.75 159.54 154.73 150.13 143.49 137.83 134.14 130.84 129.74 128.03 127.04 119.53 115.66 114.12 108.97 97.56 81.52 55.34 44.86 28.21 12.51
260 240 220 200 180 160 140 120 100 80 60 40 20 0 -20 -40
chemical shift (ppm)

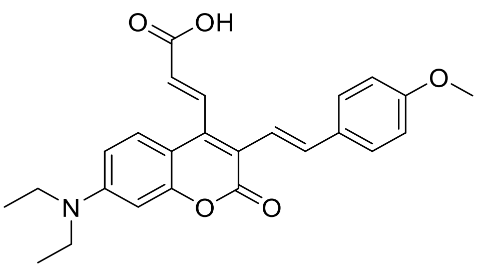

Compound 9

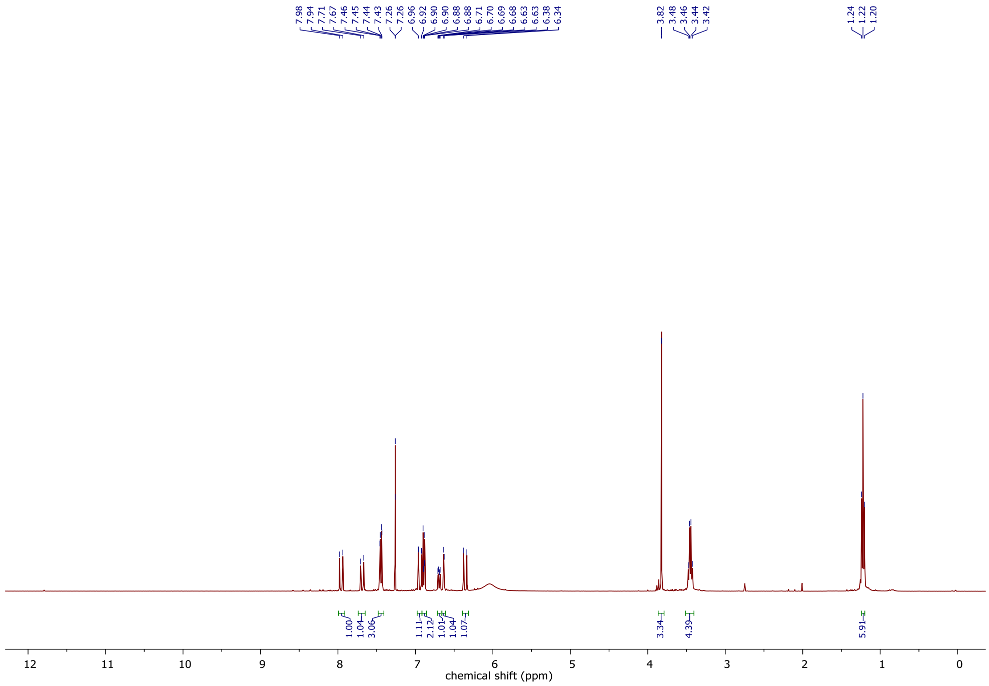
7.98 7.94 7.71 7.67 7.46 7.45 7.44 7.43 7.26 7.26 6.96 6.92 6.90 6.90 6.88 6.88 6.71 6.70 6.69 6.68 6.63 6.63 6.38 6.34
3.82 3.48 3.46 3.44 3.42
1.24 1.22 1.20
1.00 1.04 3.06 1.11 2.12 1.01 1.04 1.07 3.34 4.39 5.91
chemical shift (ppm)

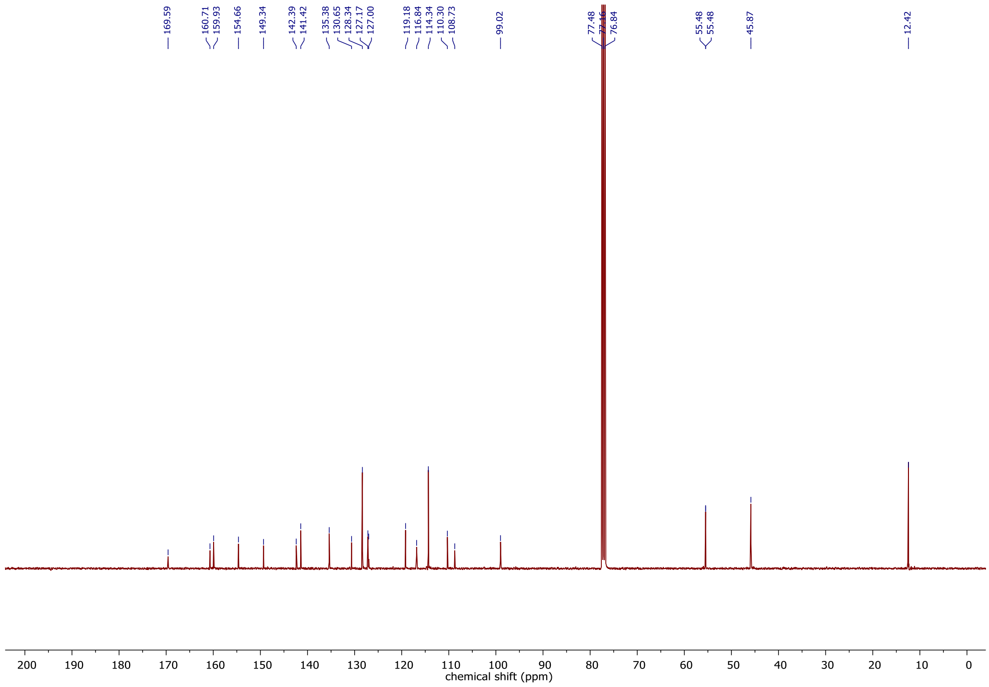
169.59 160.71 159.93 154.66 149.34 142.39 141.42 135.38 130.65 128.34 127.17 127.00 119.18 116.84 114.34 110.30 108.73 99.02 77.48 76.84 55.48 55.48 45.87 12.42
chemical shift (ppm)

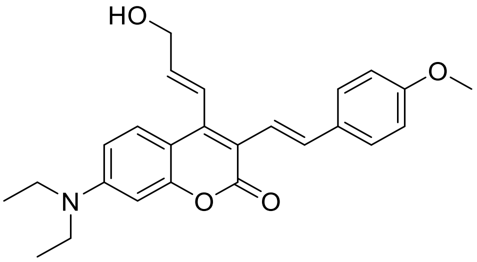
HO
O
N
O
O

Compound 10

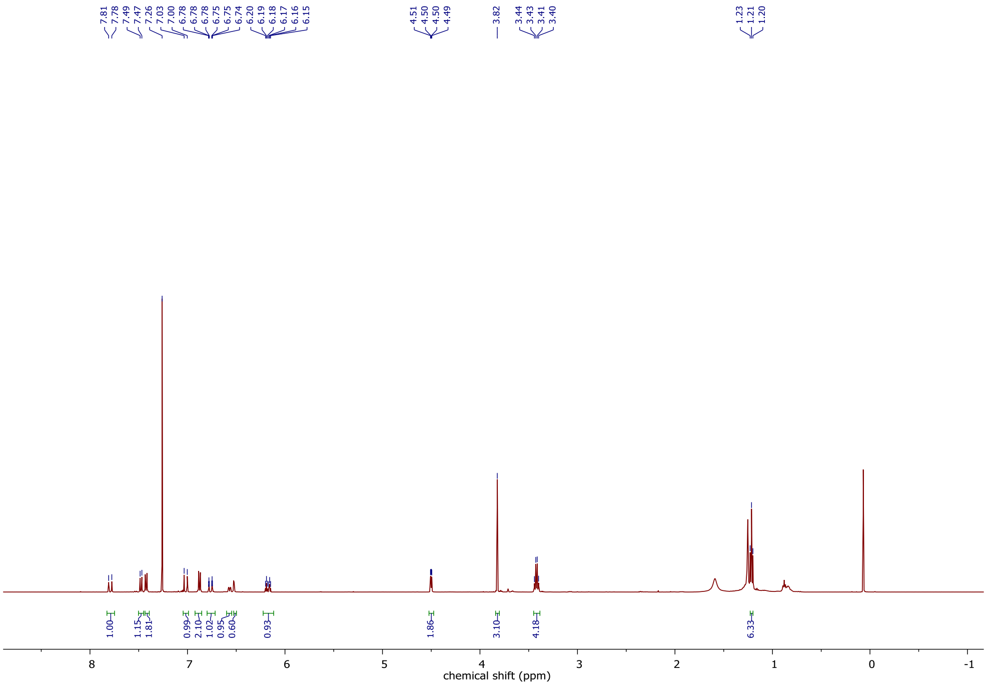
7.81
7.78
7.49
7.47
7.26
7.03
7.00
6.78
6.78
6.78
6.75
6.75
6.74
6.20
6.19
6.18
6.17
6.16
6.15
4.51
4.50
4.50
4.49
3.82
3.44
3.43
3.41
3.40
1.23
1.21
1.20
1.00
1.15
1.81
0.99
2.10
1.02
0.95
0.60
0.93
1.86
3.10
4.18
6.33
chemical shift (ppm)

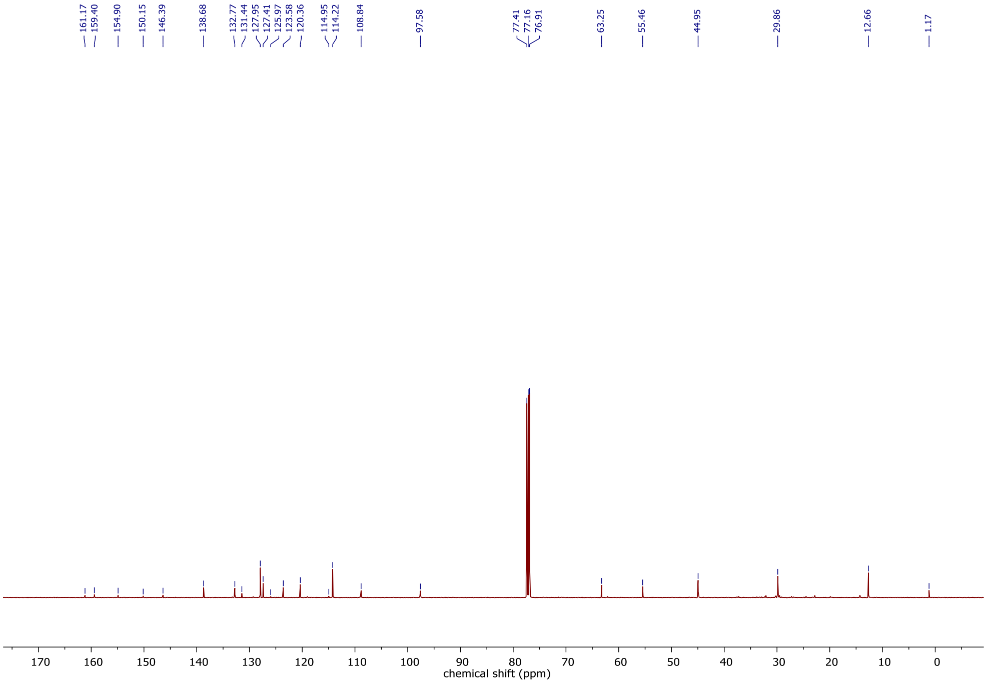
161.17
159.40
154.90
150.15
146.39
138.68
132.77
131.44
127.95
127.41
125.97
123.58
120.36
114.95
114.22
108.84
97.58
77.41
77.16
76.91
63.25
55.46
44.95
29.86
12.66
1.17
chemical shift (ppm)

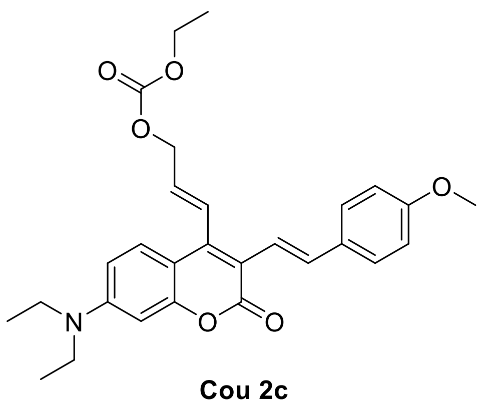
O
O
O
O
N
O
O

Cou 2c

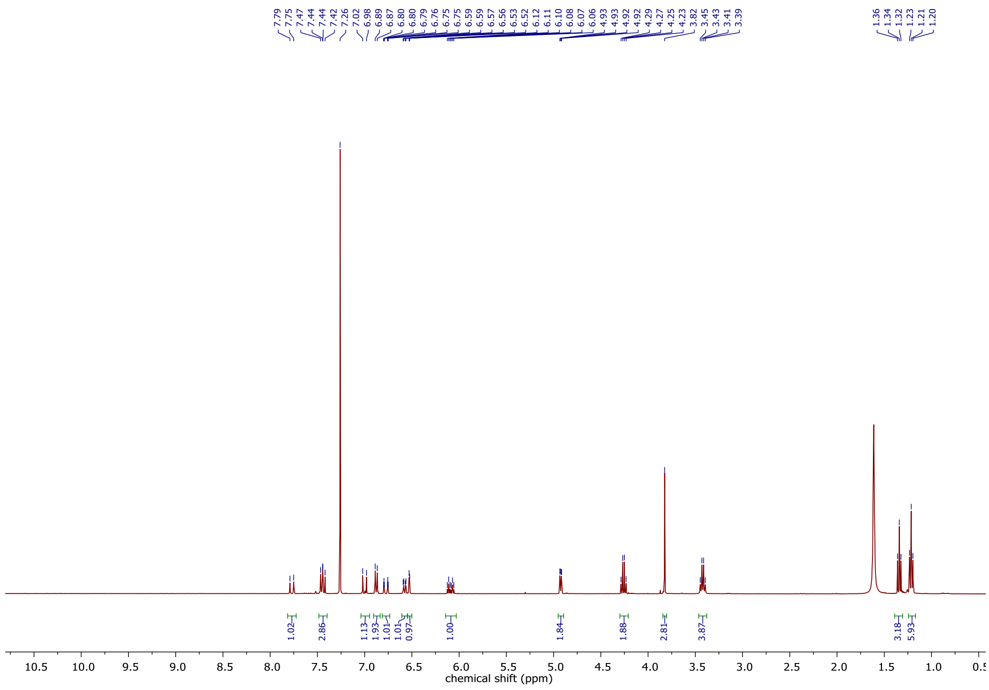
7.79
7.75
7.47
7.44
7.44
7.42
7.26
7.02
6.98
6.89
6.87
6.80
6.80
6.79
6.76
6.75
6.75
6.59
6.59
6.57
6.57
6.56
6.53
6.52
6.12
6.11
6.10
6.08
6.07
6.06
4.93
4.93
4.92
4.92
4.29
4.27
4.25
4.23
3.82
3.45
3.43
3.41
3.39
1.36
1.34
1.32
1.23
1.21
1.20
1.02
2.86
1.13
1.93
1.01
1.01
0.97
1.00
1.84
1.88
2.81
3.87
3.18
5.93
10.5 10.0 9.5 9.0 8.5 8.0 7.5 7.0 6.5 6.0 5.5 5.0 4.5 4.0 3.5 3.0 2.5 2.0 1.5 1.0
chemical shift (ppm)

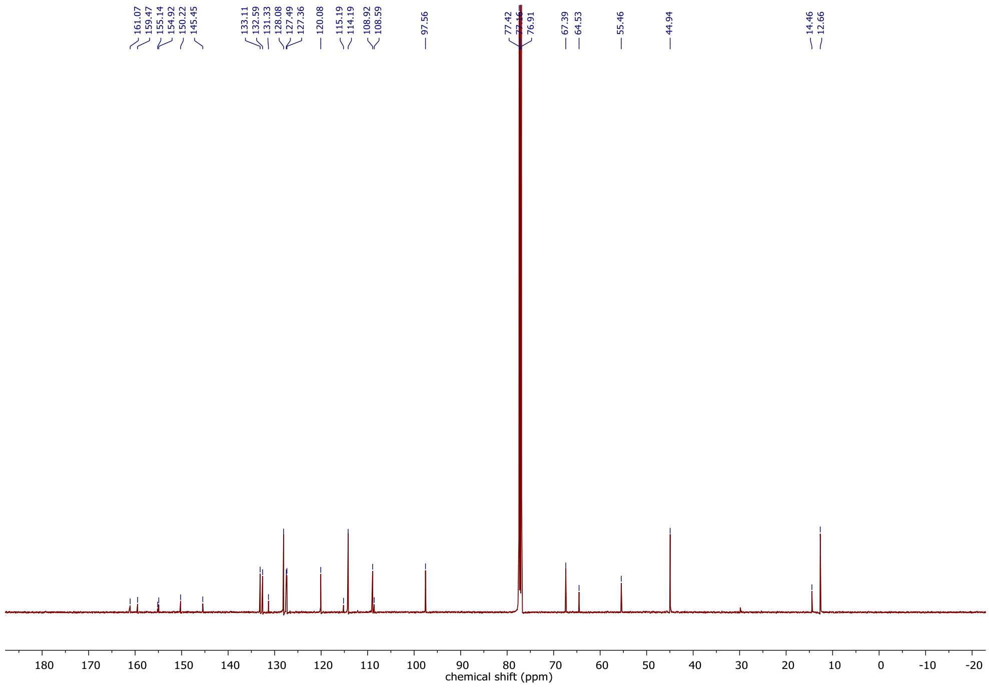
161.07
159.47
155.14
154.92
150.22
145.45
133.11
132.59
131.33
128.08
127.49
127.36
120.08
115.19
114.19
108.92
108.59
97.56
77.42
77.16
76.91
67.39
64.53
55.46
44.94
14.46
12.66
180 170 160 150 140 130 120 110 100 90 80 70 60 50 40 30 20 10 0 -10 -20
chemical shift (ppm)

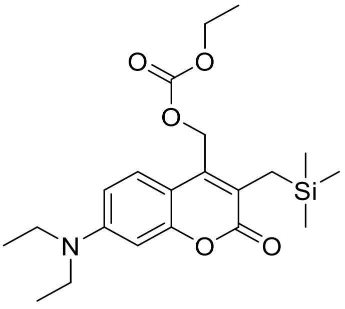
O
O
O
N
O
O
Si

Cou 1b

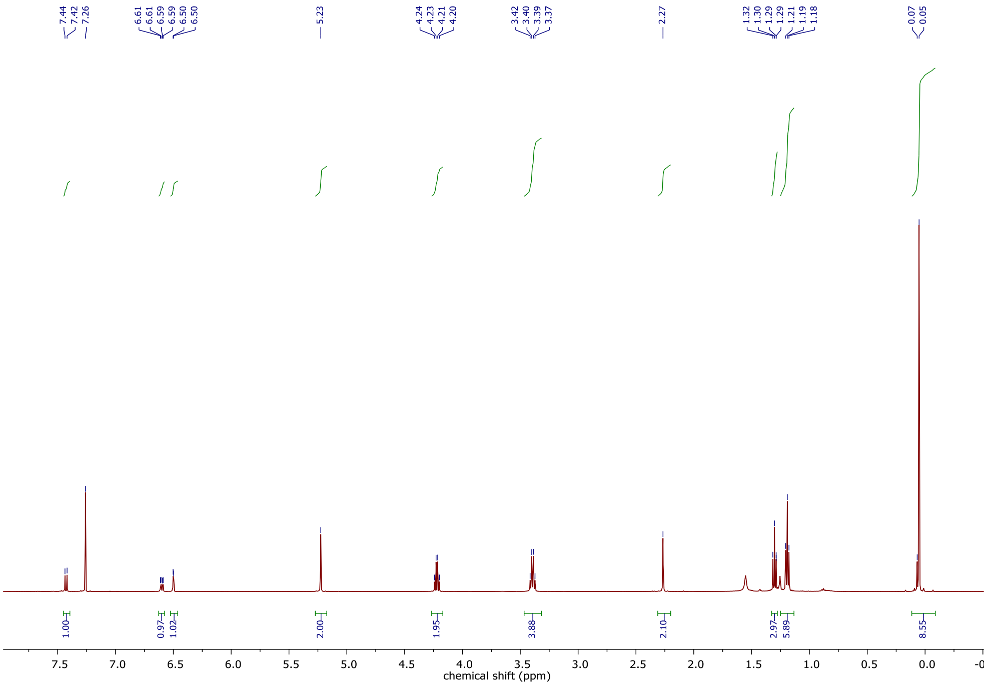
7.44
7.42
7.26
6.61
6.61
6.59
6.59
6.50
6.50
5.23
4.24
4.23
4.21
4.20
3.42
3.40
3.39
3.37
2.27
1.32
1.30
1.29
1.29
1.21
1.19
1.18
0.07
0.05
1.00
0.97
1.02
2.00
1.95
3.88
2.10
2.97
5.89
8.55
chemical shift (ppm)

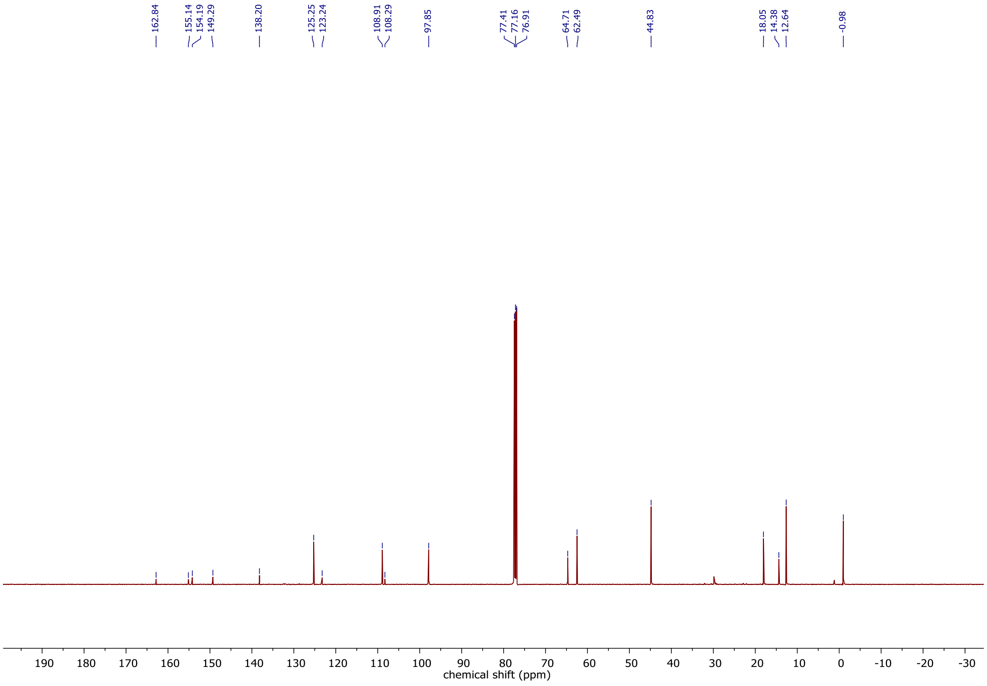
162.84
155.14
154.19
149.29
138.20
125.25
123.24
108.91
108.29
97.85
77.41
77.16
76.91
64.71
62.49
44.83
18.05
14.38
12.64
-0.98
chemical shift (ppm)

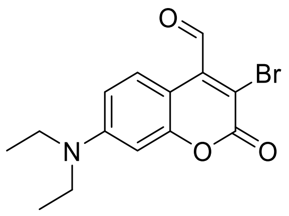

Compound 12

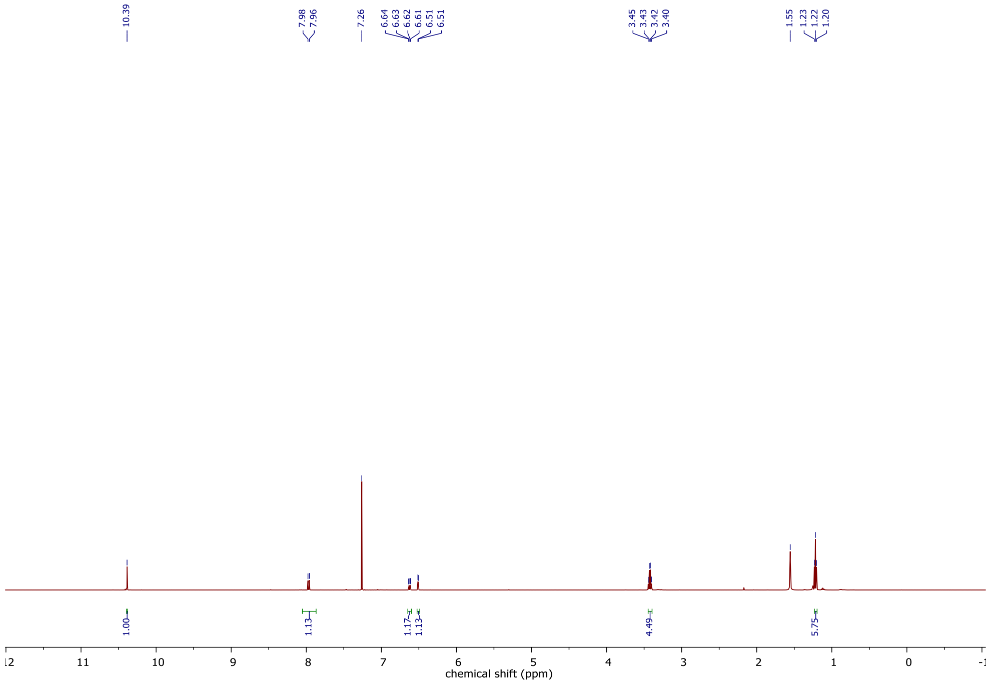
10.39
7.98
7.96
7.26
6.64
6.63
6.62
6.61
6.51
6.51
3.45
3.43
3.42
3.40
1.55
1.23
1.22
1.20
1.00
1.13
1.17
1.13
4.49
5.75
chemical shift (ppm)

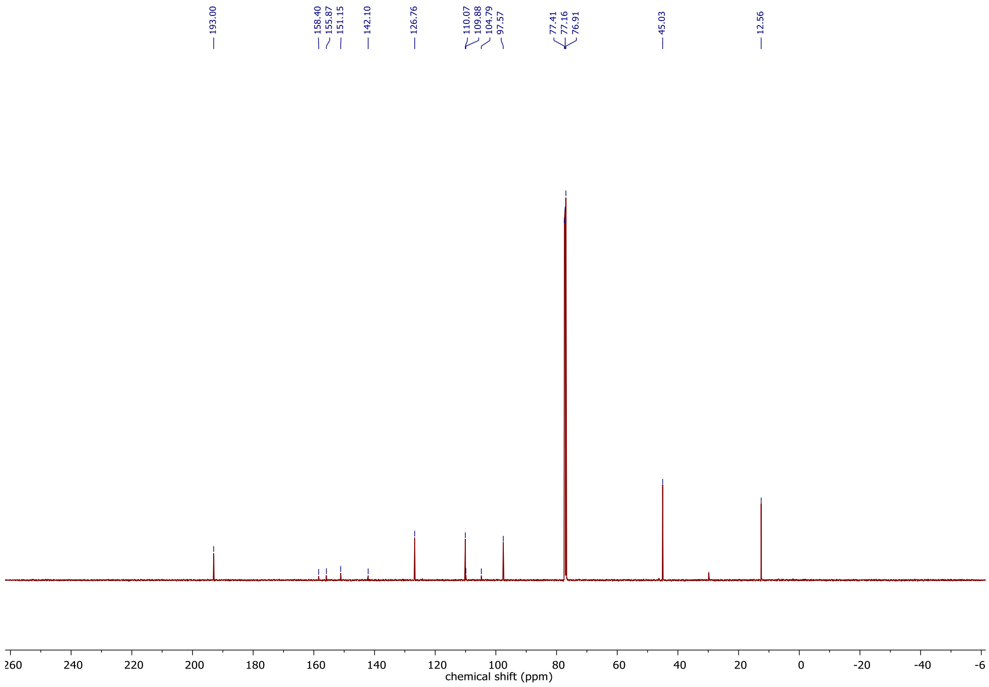
193.00
158.40
155.87
151.15
142.10
126.76
110.07
109.88
104.79
97.57
77.41
77.16
76.91
45.03
12.56
chemical shift (ppm)

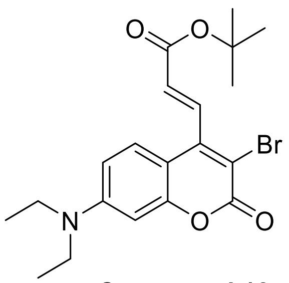
O
O
Br
N
O
O

Compound 13

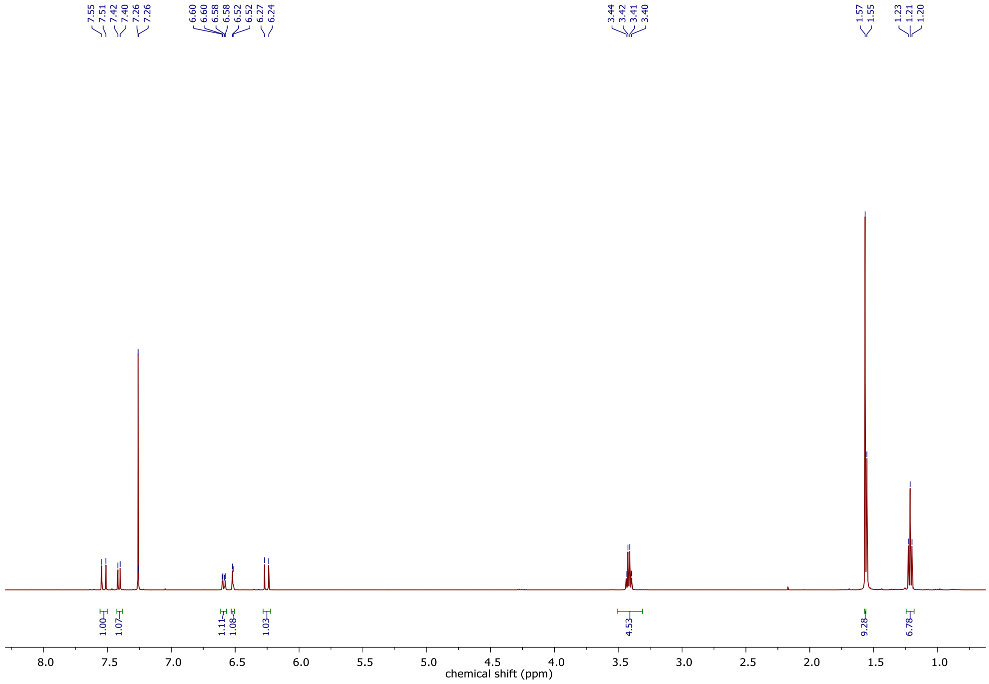
7.55
7.51
7.42
7.40
7.26
7.26
6.60
6.60
6.58
6.58
6.52
6.52
6.27
6.24
3.44
3.42
3.41
3.40
1.57
1.55
1.23
1.21
1.20
1.00
1.07
1.11
1.08
1.03
4.53
9.28
6.78
8.0 7.5 7.0 6.5 6.0 5.5 5.0 4.5 4.0 3.5 3.0 2.5 2.0 1.5 1.0
chemical shift (ppm)

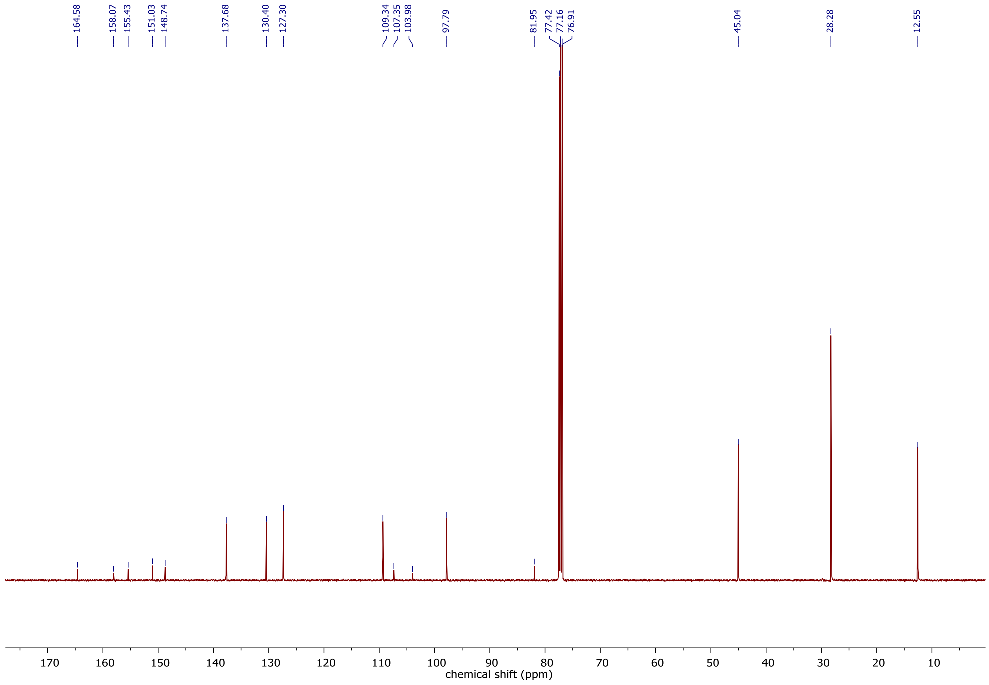
164.58
158.07
155.43
151.03
148.74
137.68
130.40
127.30
109.34
107.35
103.98
97.79
81.95
77.42
77.16
76.91
45.04
28.28
12.55
170 160 150 140 130 120 110 100 90 80 70 60 50 40 30 20 10
chemical shift (ppm)

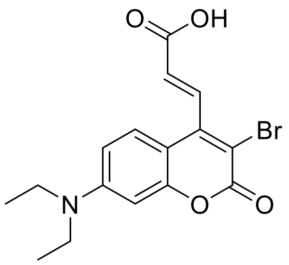

Compound 14

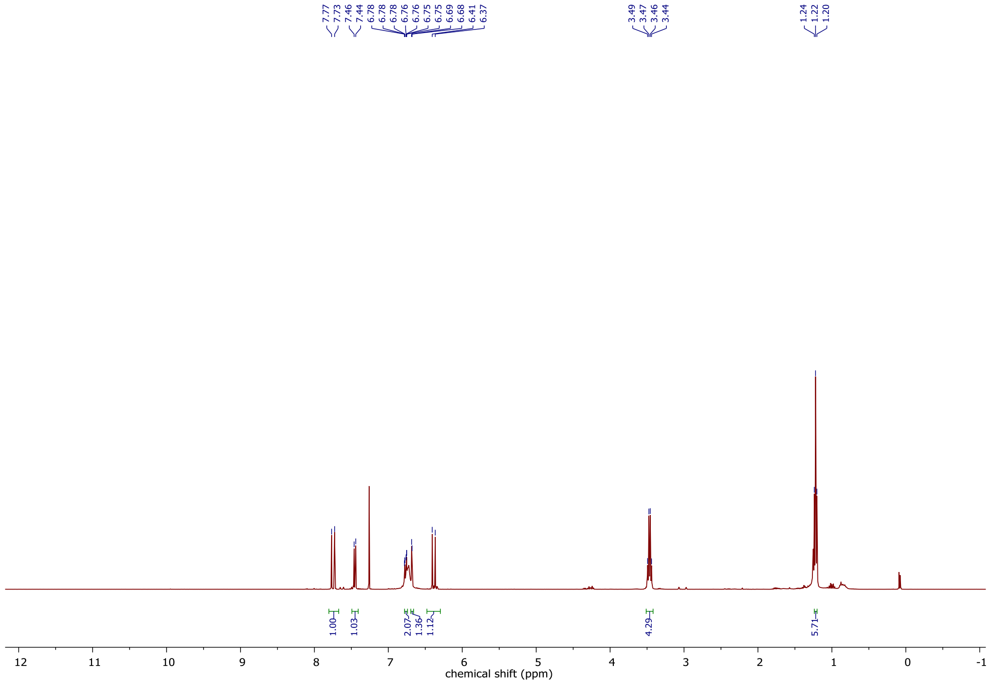
7.77
7.73
7.46
7.44
6.78
6.78
6.76
6.76
6.75
6.75
6.69
6.68
6.41
6.37
3.49
3.47
3.46
3.44
1.24
1.22
1.20
1.00
1.03
2.07
1.36
1.12
4.29
5.71
12 11 10 9 8 7 6 5 4 3 2 1 0 -1
chemical shift (ppm)

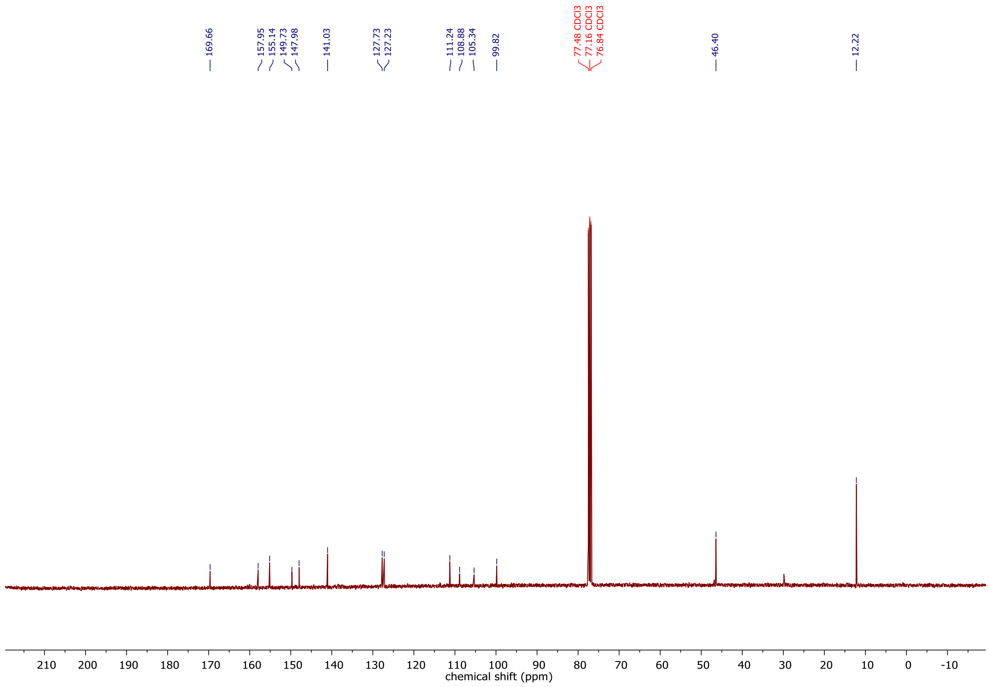
169.66
157.95
155.14
149.73
147.98
141.03
127.73
127.23
111.24
108.88
105.34
99.82
77.48 CDCl3
77.16 CDCl3
76.84 CDCl3
46.40
12.22
210 200 190 180 170 160 150 140 130 120 110 100 90 80 70 60 50 40 30 20 10 0 -10
chemical shift (ppm)

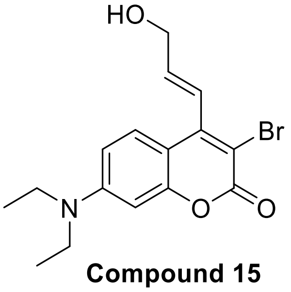
HO
Br
N
O
O

Compound 15

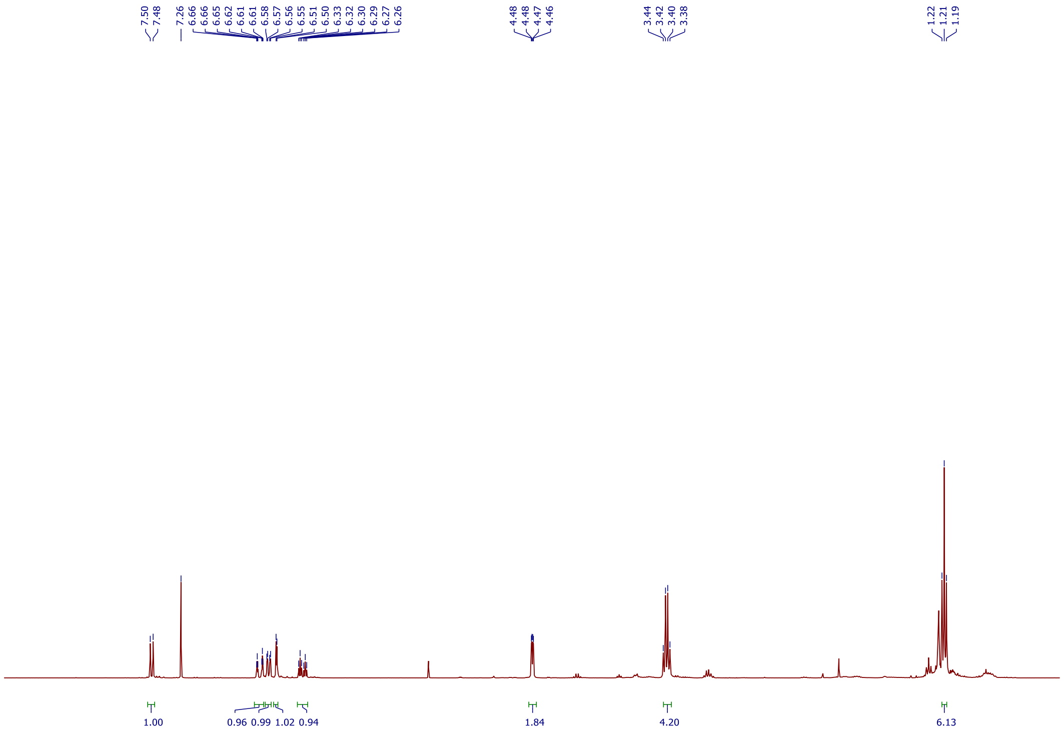
7.50
7.48
7.26
6.66
6.66
6.65
6.62
6.61
6.61
6.58
6.57
6.56
6.55
6.51
6.50
6.33
6.32
6.30
6.29
6.27
6.26
4.48
4.48
4.47
4.46
3.44
3.42
3.40
3.38
1.22
1.21
1.19
1.00
0.96
0.99
1.02
0.94
1.84
4.20
6.13

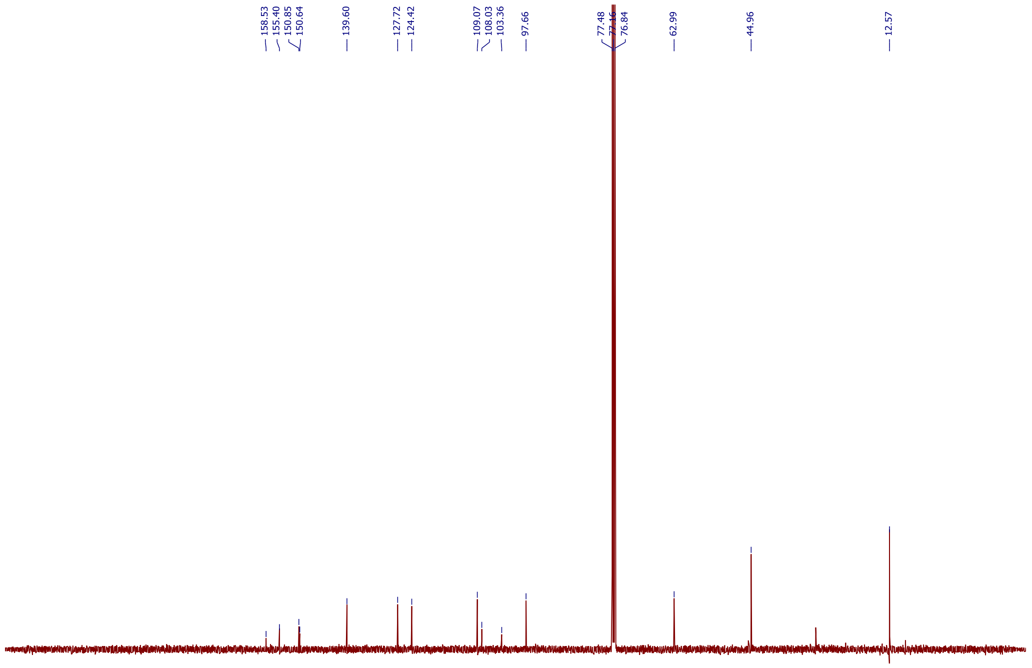
158.53
155.40
150.85
150.64
139.60
127.72
124.42
109.07
108.03
103.36
97.66
77.48
76.84
62.99
44.96
12.57

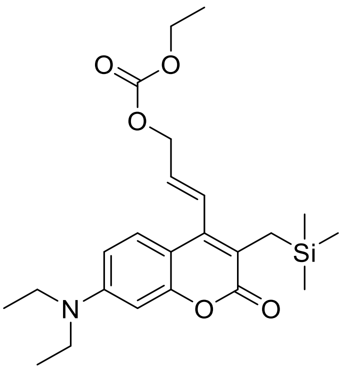
O
O
O
Si
N
O
O

Cou 2b

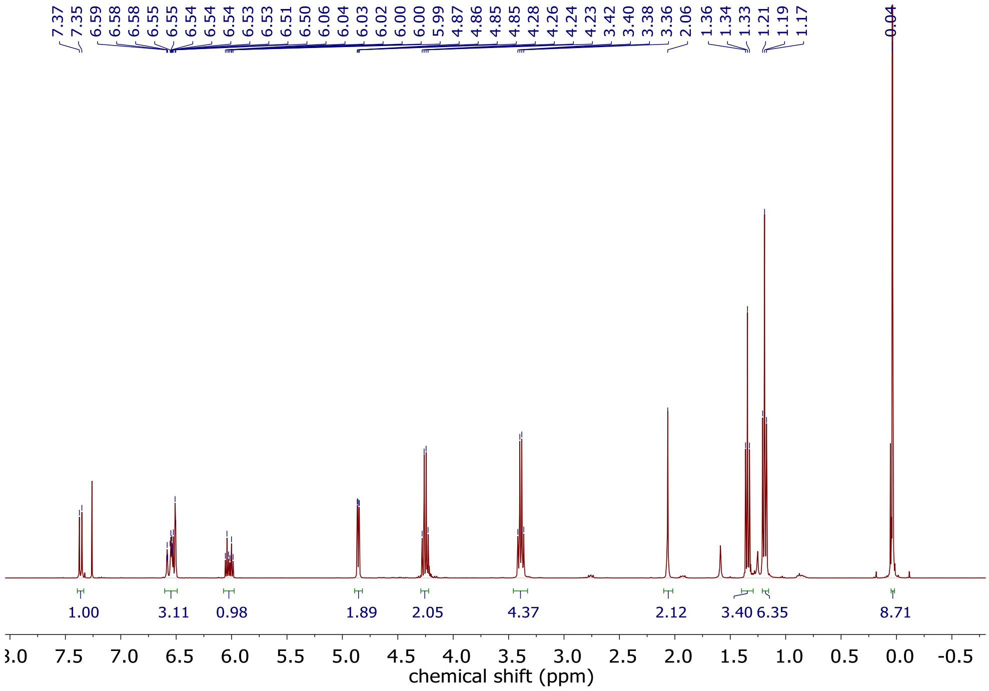
7.37
7.35
6.59
6.58
6.58
6.55
6.55
6.54
6.54
6.54
6.53
6.53
6.51
6.50
6.06
6.04
6.03
6.02
6.00
6.00
5.99
4.87
4.86
4.85
4.85
4.28
4.26
4.24
4.24
4.23
3.42
3.40
3.38
3.36
2.06
1.36
1.34
1.33
1.21
1.19
1.17
0.04
1.00
3.11
0.98
1.89
2.05
4.37
2.12
3.40
6.35
8.71
chemical shift (ppm)

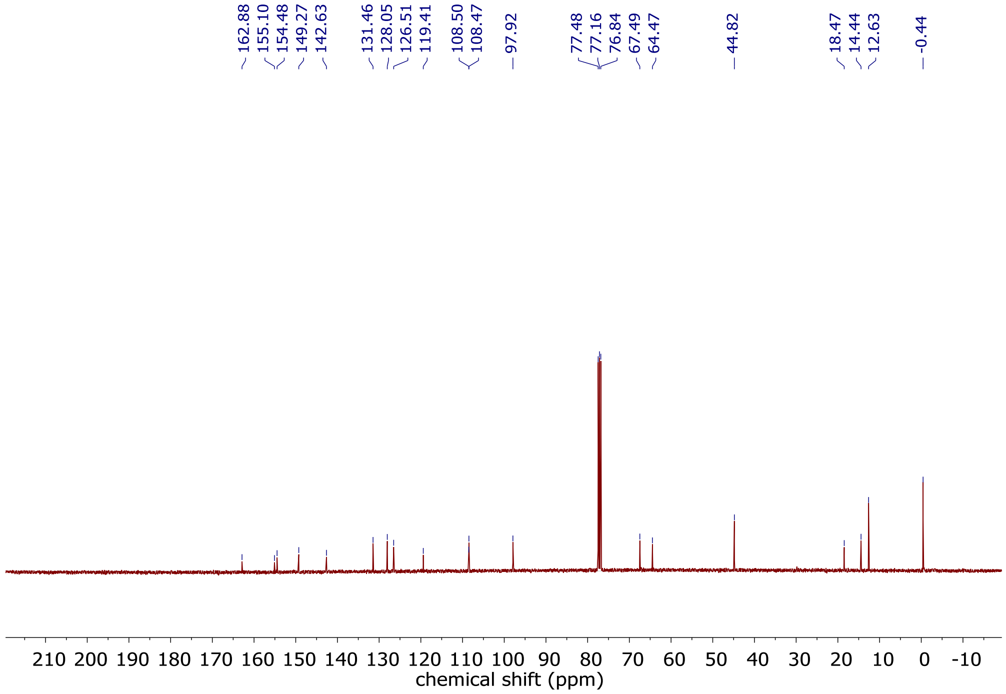
162.88
155.10
154.48
149.27
142.63
131.46
128.05
126.51
119.41
108.50
108.47
97.92
77.48
77.16
76.84
67.49
64.47
44.82
18.47
14.44
12.63
-0.44
chemical shift (ppm)

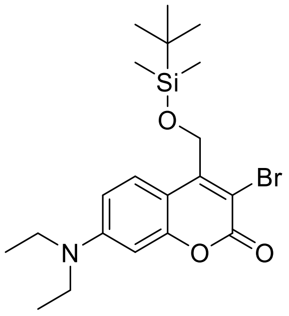
Si
O
Br
N
O
O

Compound 16

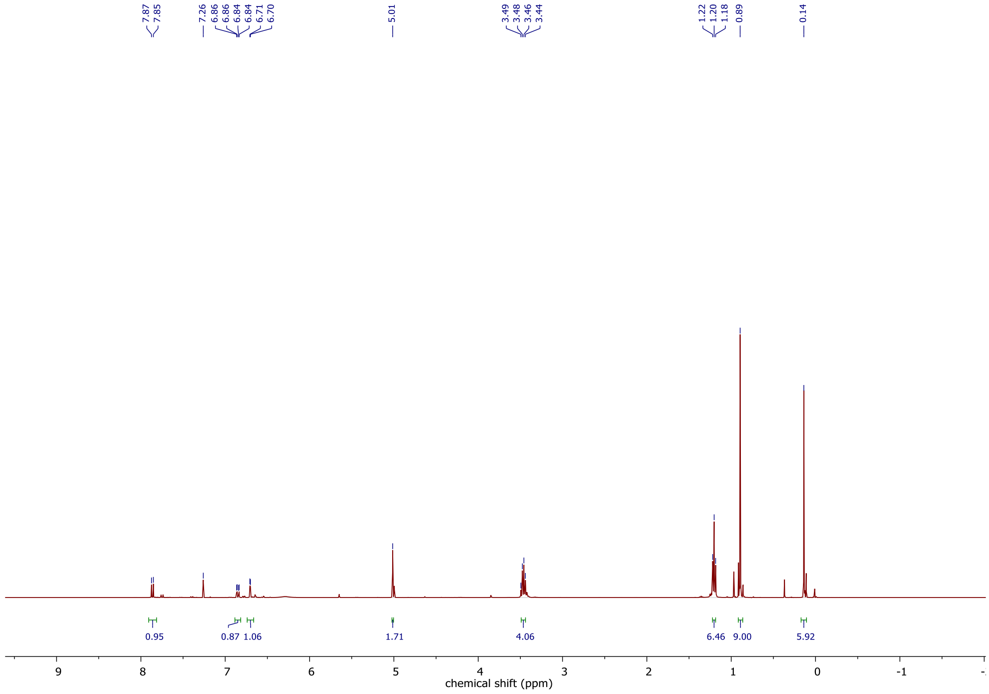
7.87
7.85
7.26
6.86
6.86
6.84
6.84
6.71
6.70
5.01
3.49
3.48
3.46
3.44
1.22
1.20
1.18
0.89
0.14
0.95
0.87
1.06
1.71
4.06
6.46
9.00
5.92
9
8
7
6
5
4
3
2
1
0
-1
chemical shift (ppm)

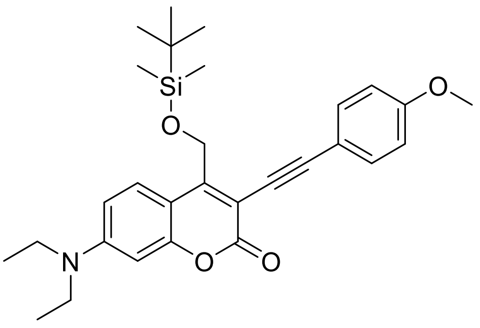
Si
O
O
N
O
O

Compound 17

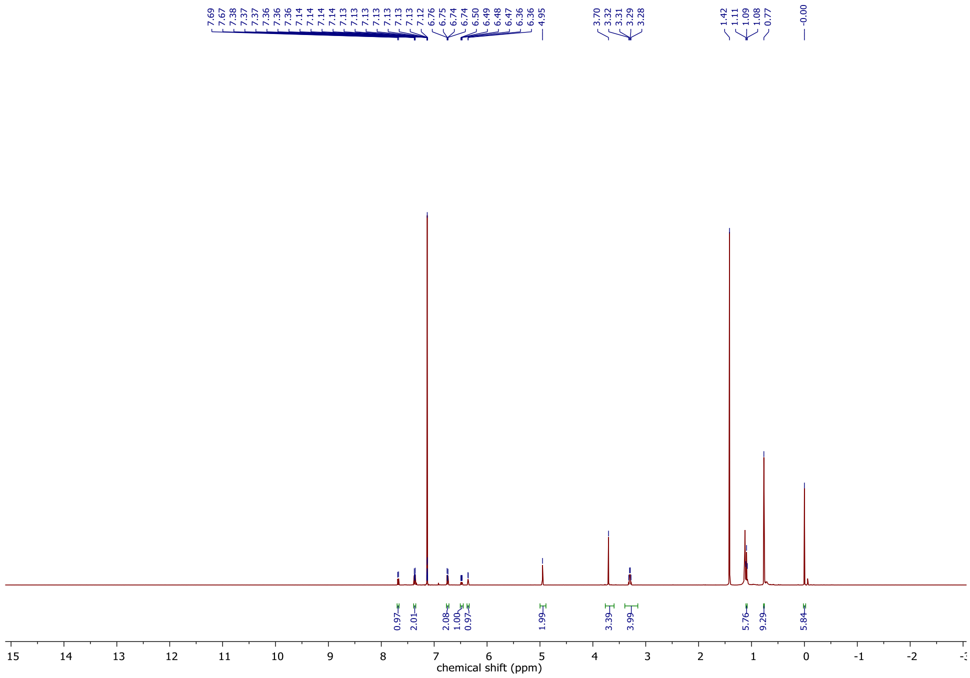
7.69
7.67
7.38
7.37
7.37
7.36
7.36
7.36
7.14
7.14
7.14
7.14
7.13
7.13
7.13
7.13
7.13
7.13
7.13
7.12
6.76
6.75
6.74
6.74
6.50
6.49
6.48
6.47
6.36
6.36
4.95
3.70
3.32
3.31
3.29
3.28
1.42
1.11
1.09
1.08
0.77
-0.00
0.97
2.01
2.08
1.00
0.97
1.99
3.39
3.99
5.76
9.29
5.84
chemical shift (ppm)

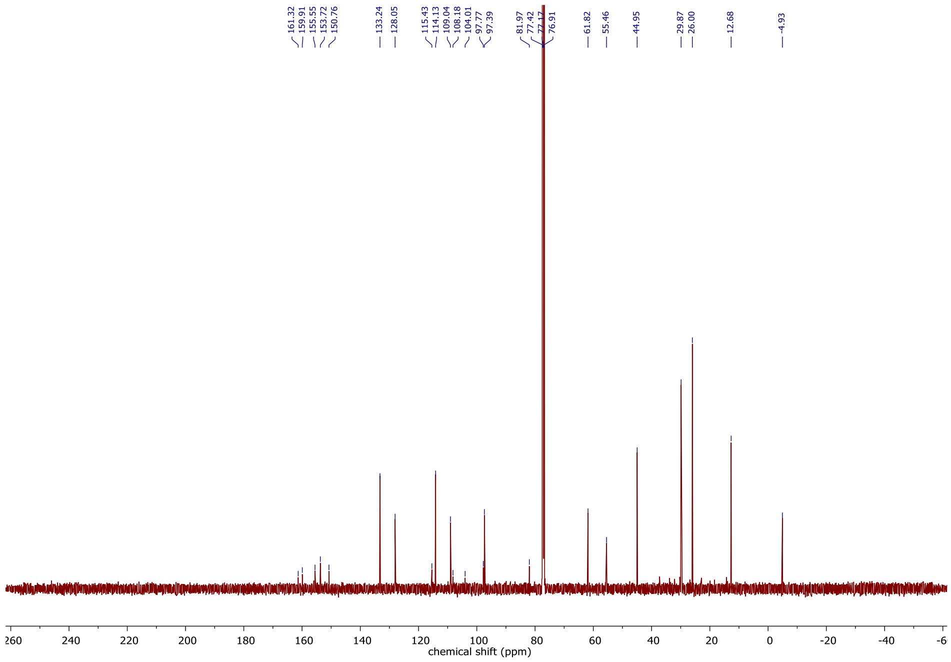
161.32
159.91
155.55
153.72
150.76
133.24
128.05
115.43
114.13
109.04
108.18
104.01
97.77
97.39
81.97
77.42
77.17
76.91
61.82
55.46
44.95
29.87
26.00
12.68
-4.93
chemical shift (ppm)

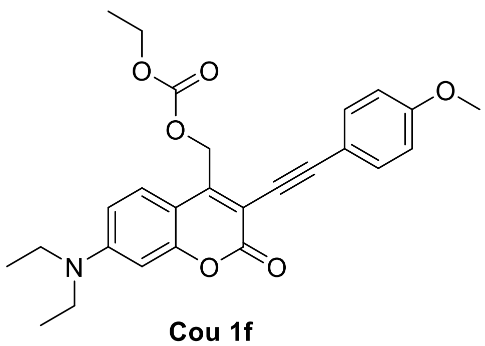

Cou 1f

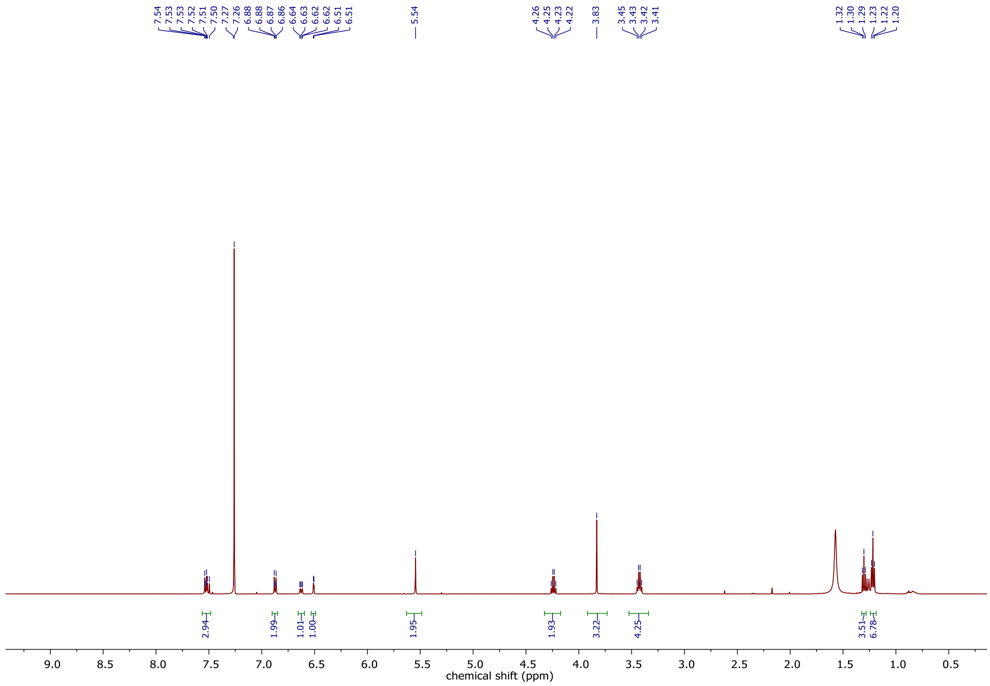
7.54 7.53 7.53 7.52 7.51 7.50 7.27 7.26 6.88 6.88 6.87 6.86 6.64 6.63 6.62 6.62 6.51 6.51
5.54
4.26 4.25 4.23 4.22
3.83
3.45 3.43 3.42 3.41
1.32 1.30 1.29 1.23 1.22 1.20
2.94 1.99 1.01 1.00 1.95 1.93 3.22 4.25 3.51 6.78
9.0 8.5 8.0 7.5 7.0 6.5 6.0 5.5 5.0 4.5 4.0 3.5 3.0 2.5 2.0 1.5 1.0 0.5
chemical shift (ppm)

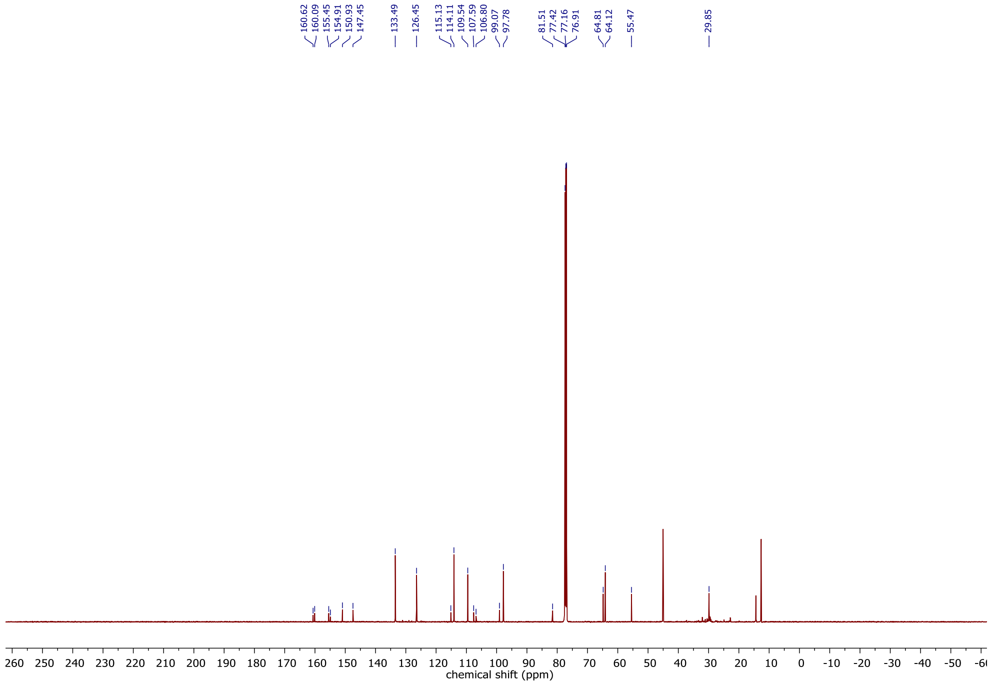
160.62 160.09 155.45 154.91 150.93 147.45
133.49
126.45
115.13 114.11 109.54 107.59 106.80 99.07 97.78
81.51 77.42 77.16 76.91
64.81 64.12
55.47
29.85
260 250 240 230 220 210 200 190 180 170 160 150 140 130 120 110 100 90 80 70 60 50 40 30 20 10 0 -10 -20 -30 -40 -50 -60
chemical shift (ppm)

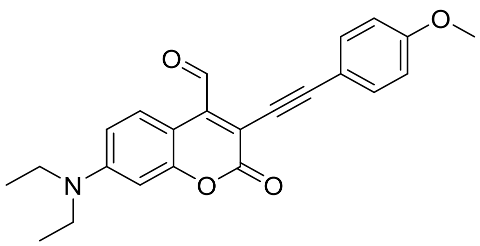
O
O
O
N
O

Compound 18

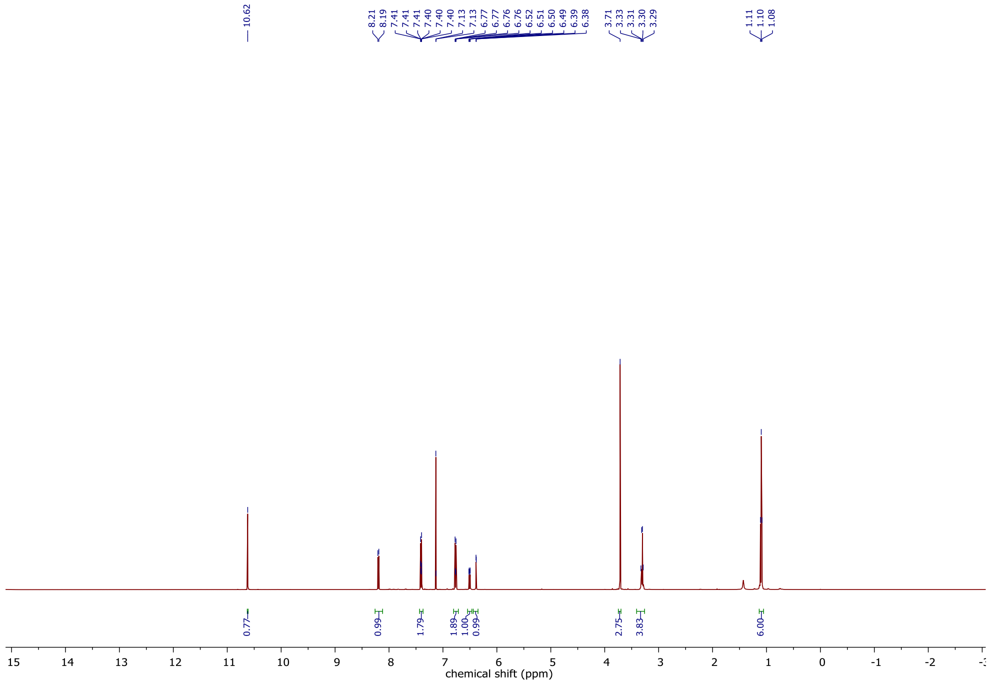
10.62
8.21
8.19
7.41
7.41
7.40
7.40
7.40
7.13
7.13
6.77
6.77
6.76
6.76
6.52
6.51
6.50
6.49
6.39
6.38
3.71
3.33
3.31
3.30
3.29
1.11
1.10
1.08
0.77
0.99
1.79
1.89
1.00
0.99
2.75
3.83
6.00
chemical shift (ppm)

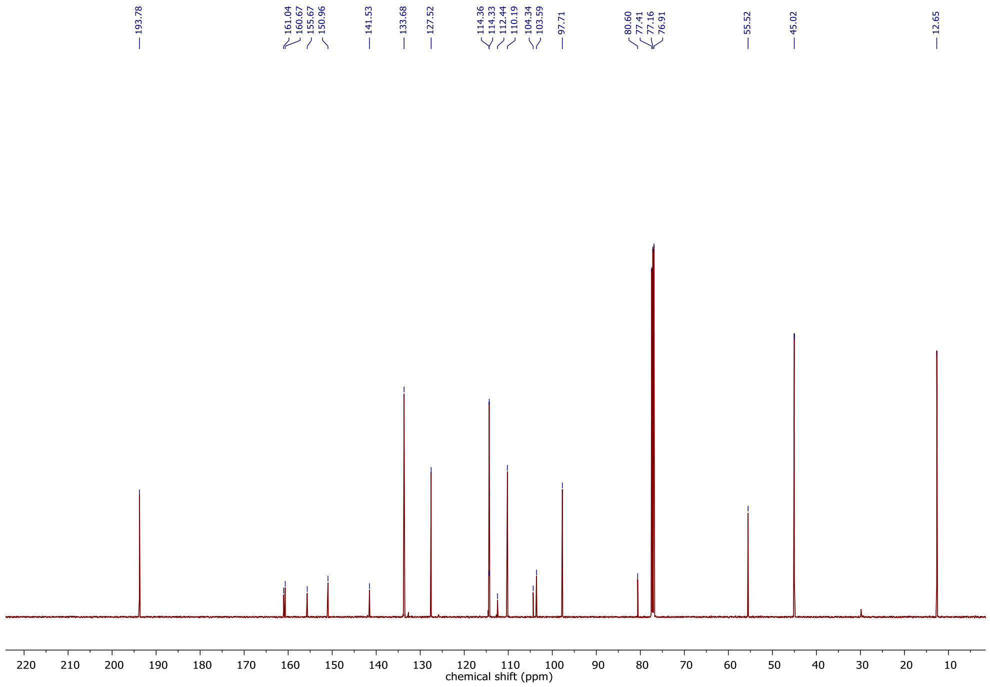
193.78
161.04
160.67
155.67
150.96
141.53
133.68
127.52
114.36
114.33
112.44
110.19
104.34
103.59
97.71
80.60
77.41
77.16
76.91
55.52
45.02
12.65
chemical shift (ppm)

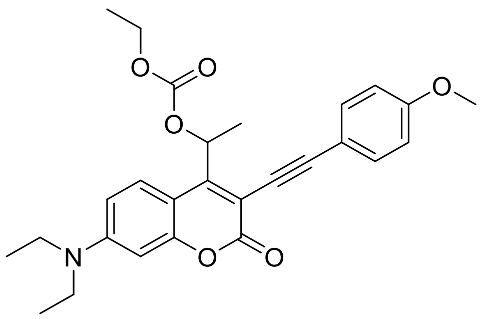

Cou 3b

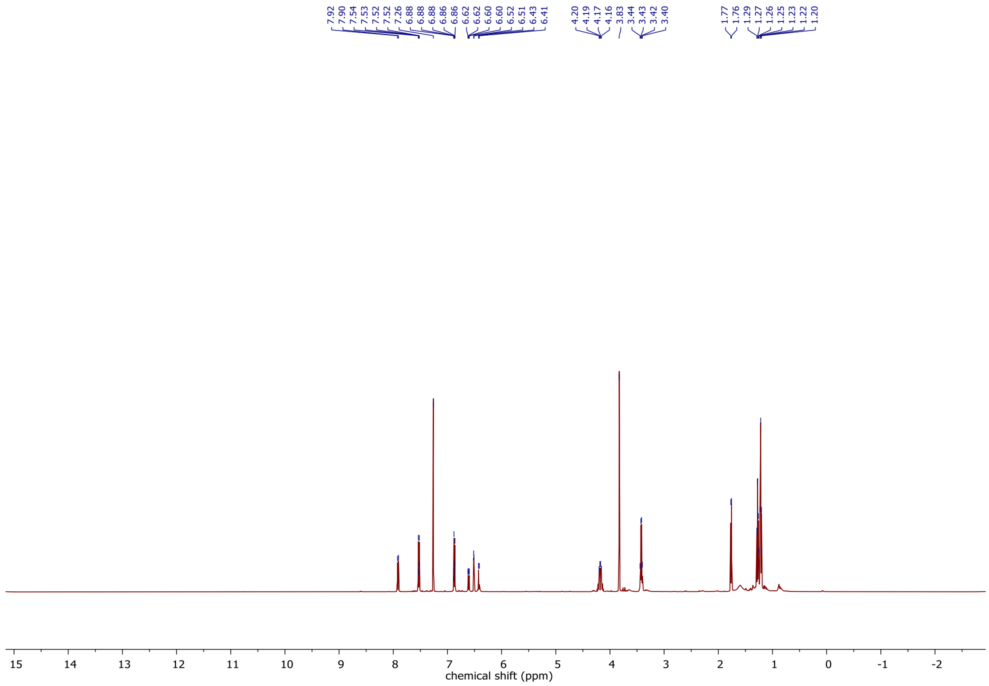
7.92
7.90
7.54
7.53
7.52
7.52
7.26
6.88
6.88
6.88
6.86
6.86
6.62
6.62
6.60
6.60
6.52
6.51
6.43
6.41
4.20
4.19
4.17
4.16
3.83
3.44
3.43
3.42
3.40
1.77
1.76
1.29
1.27
1.26
1.25
1.23
1.22
1.20
chemical shift (ppm)

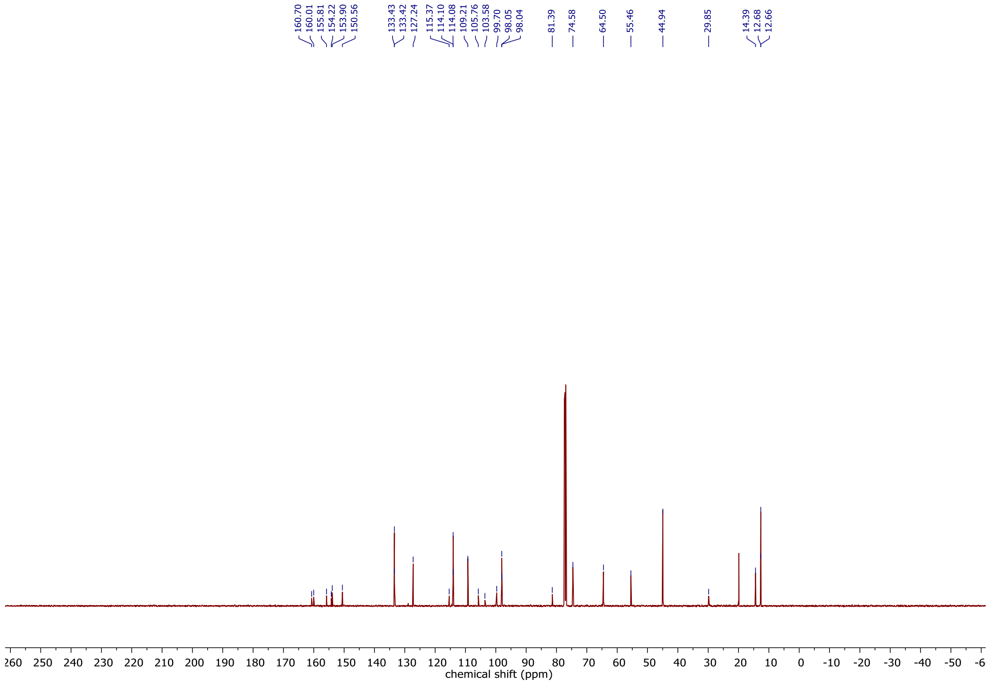
160.70
160.01
155.81
154.22
153.90
150.56
133.43
133.42
127.24
115.37
114.10
114.08
109.21
105.76
103.58
99.70
98.05
98.04
81.39
74.58
64.50
55.46
44.94
29.85
14.39
12.68
12.66
chemical shift (ppm)

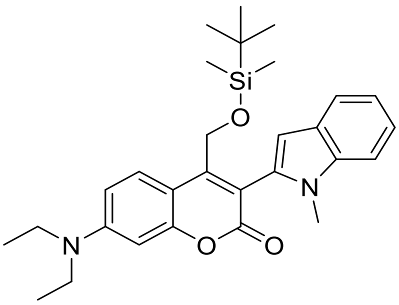

Compound 19-1

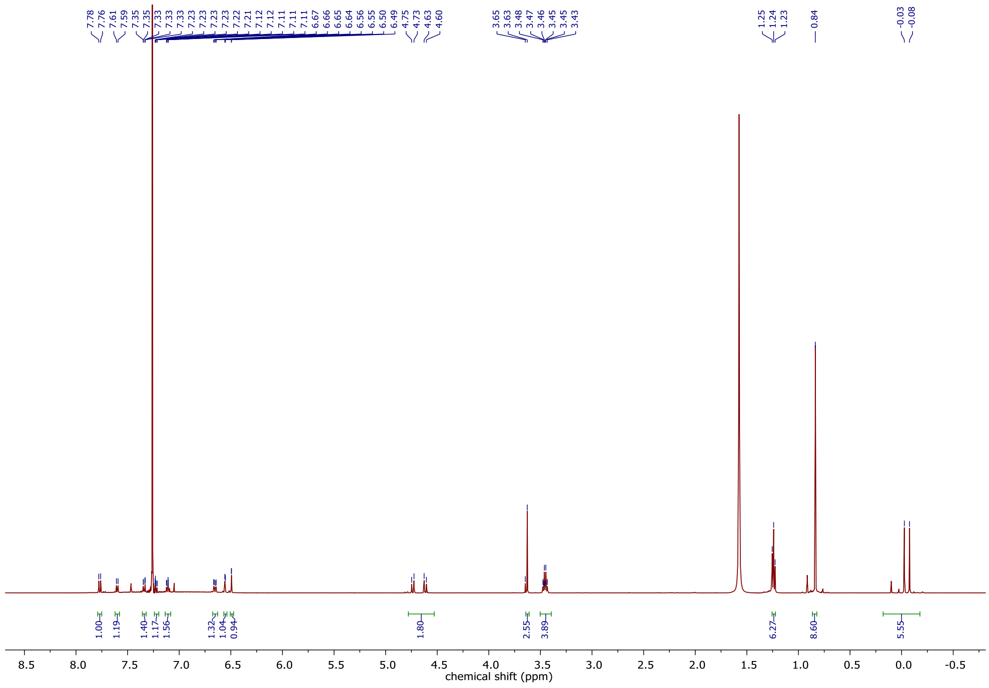

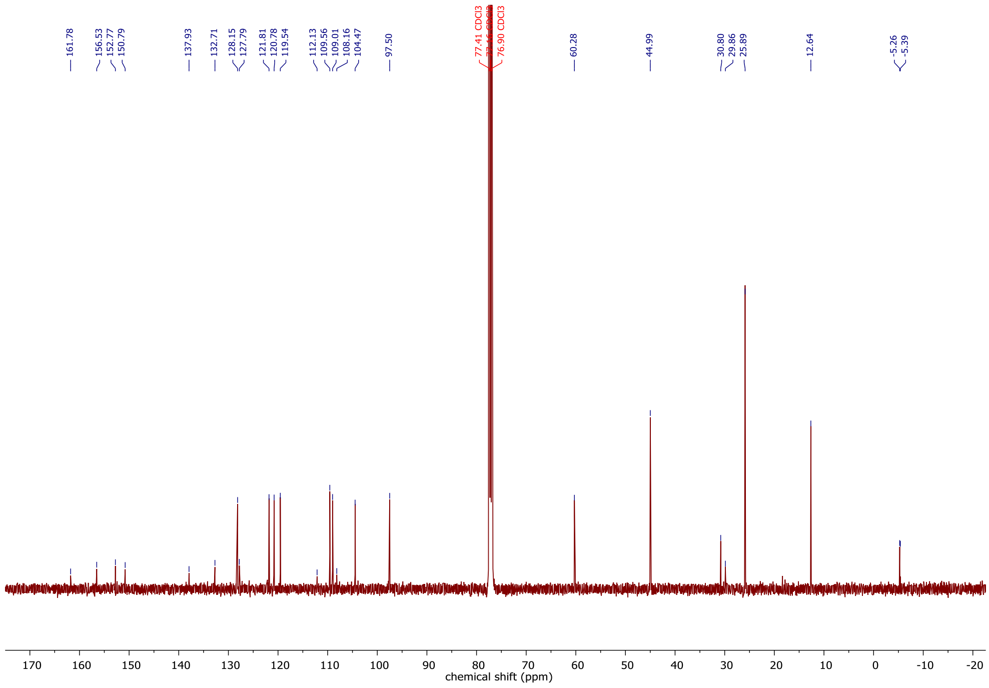

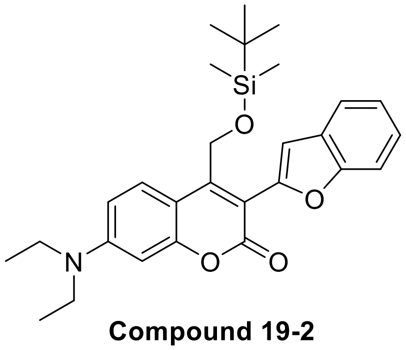

Compound 19-2

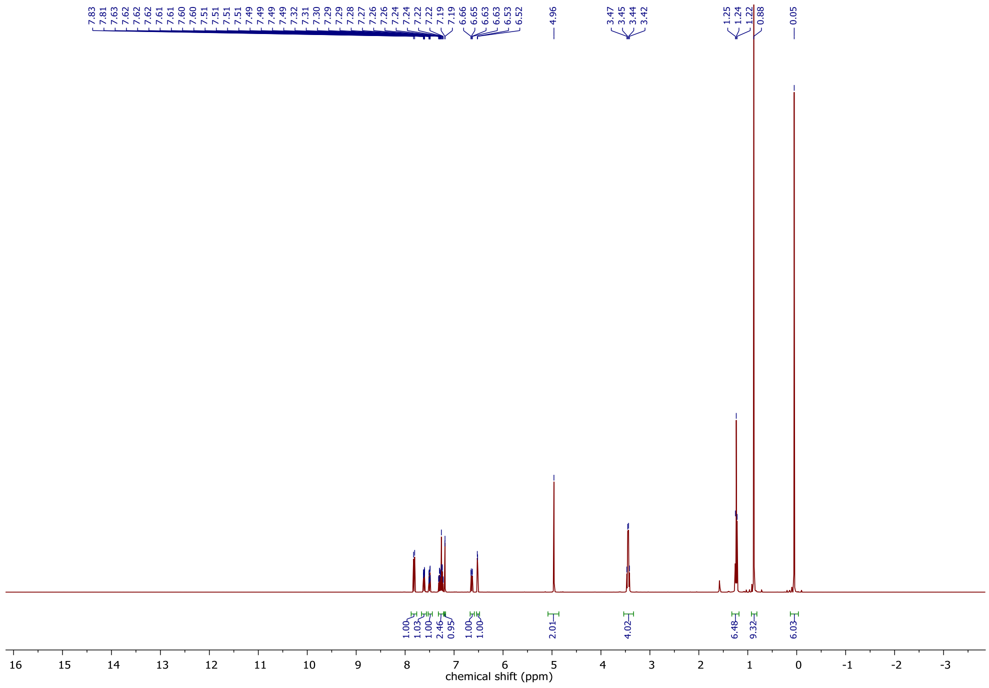

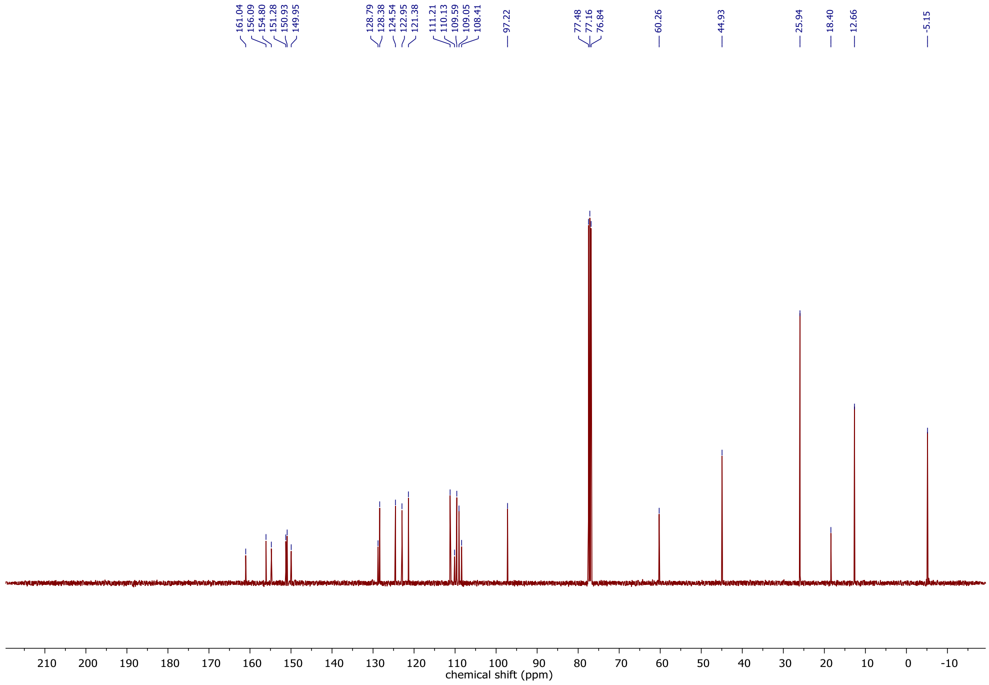

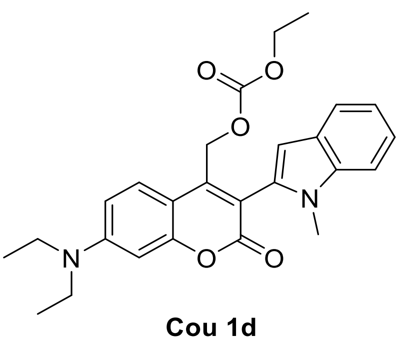

Cou 1d

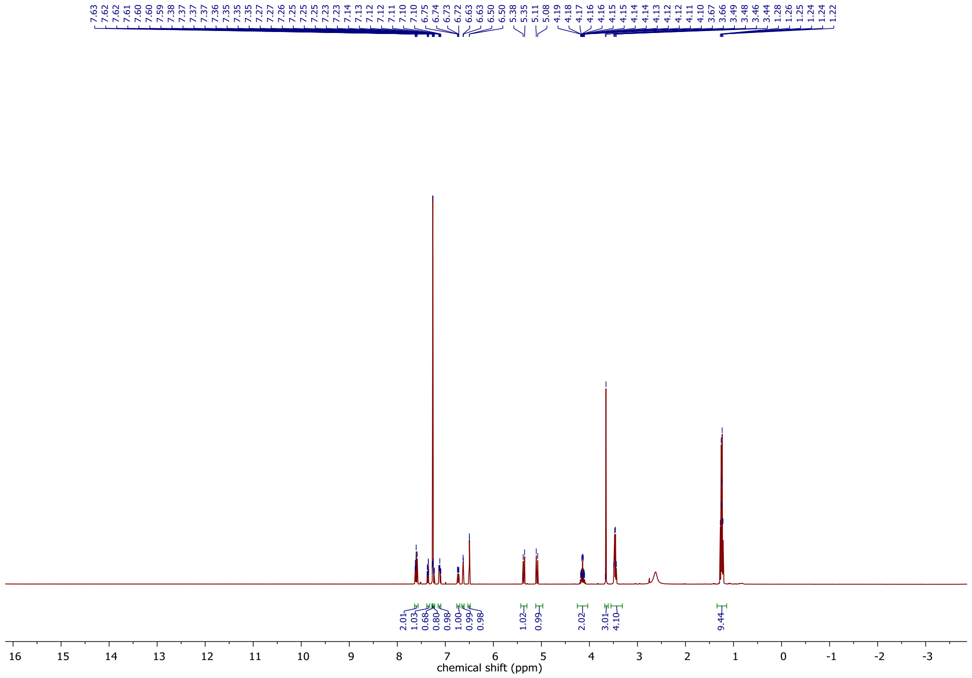

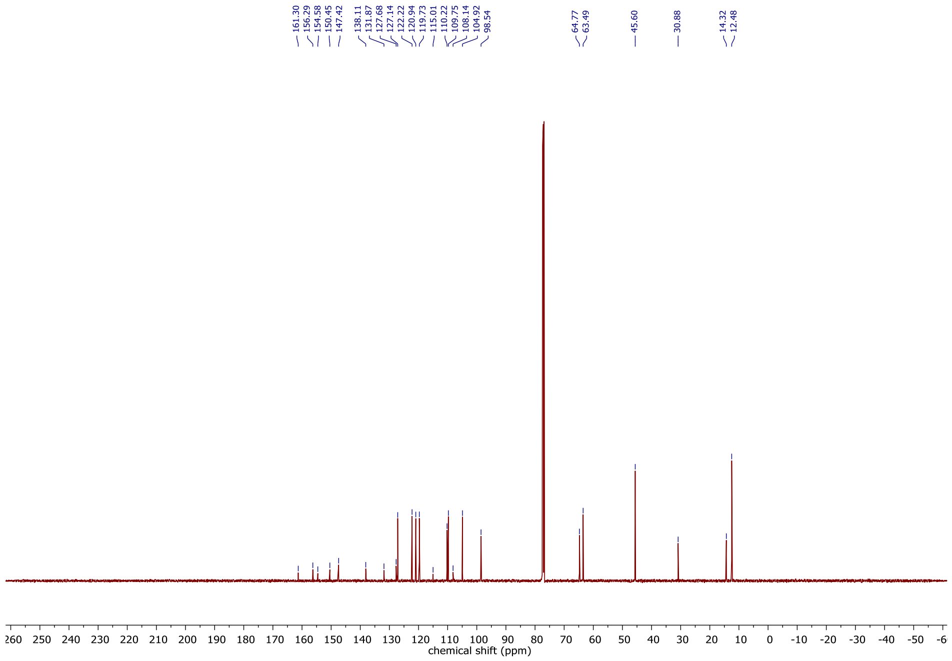

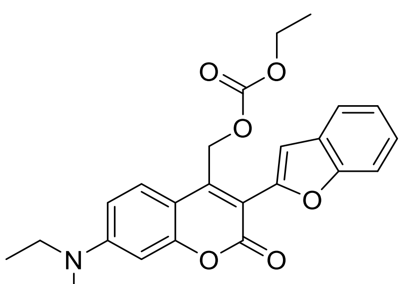

Cou 1e

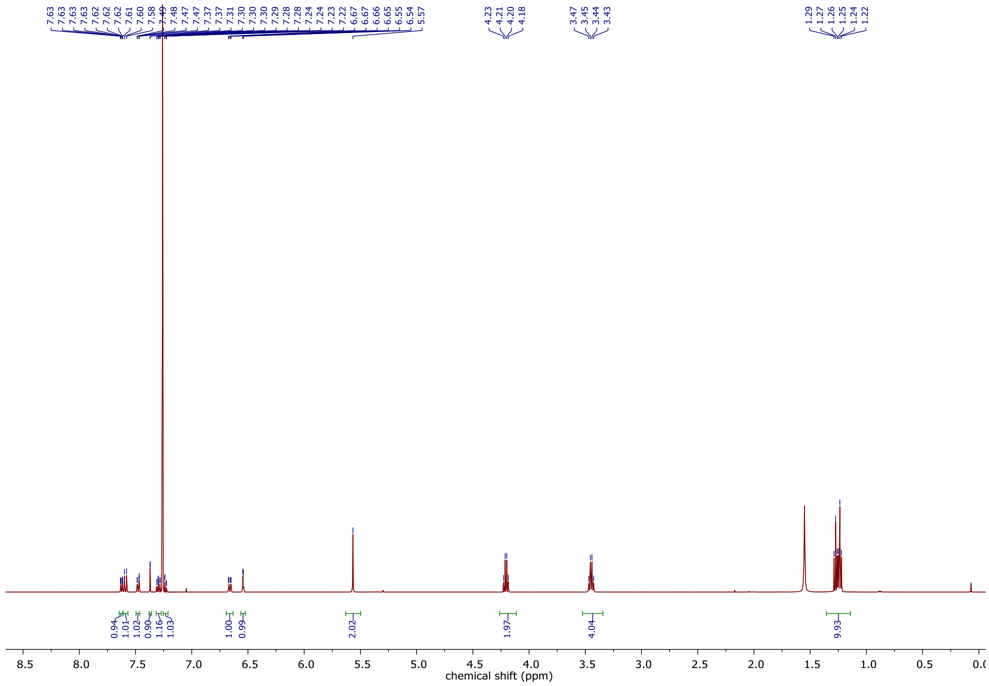
7.63 7.63 7.63 7.62 7.62 7.61 7.60 7.58 7.48 7.47 7.47 7.37 7.37 7.31 7.30 7.30 7.30 7.29 7.28 7.28 7.24 7.24 7.23 7.22 6.67 6.67 6.66 6.65 6.55 6.54 5.57
4.23 4.21 4.20 4.18
3.47 3.45 3.44 3.43
1.29 1.27 1.26 1.25 1.24 1.22
0.94 1.01 1.02 0.90 1.16 1.03 1.00 0.99 2.02 1.97 4.04 9.93
chemical shift (ppm)

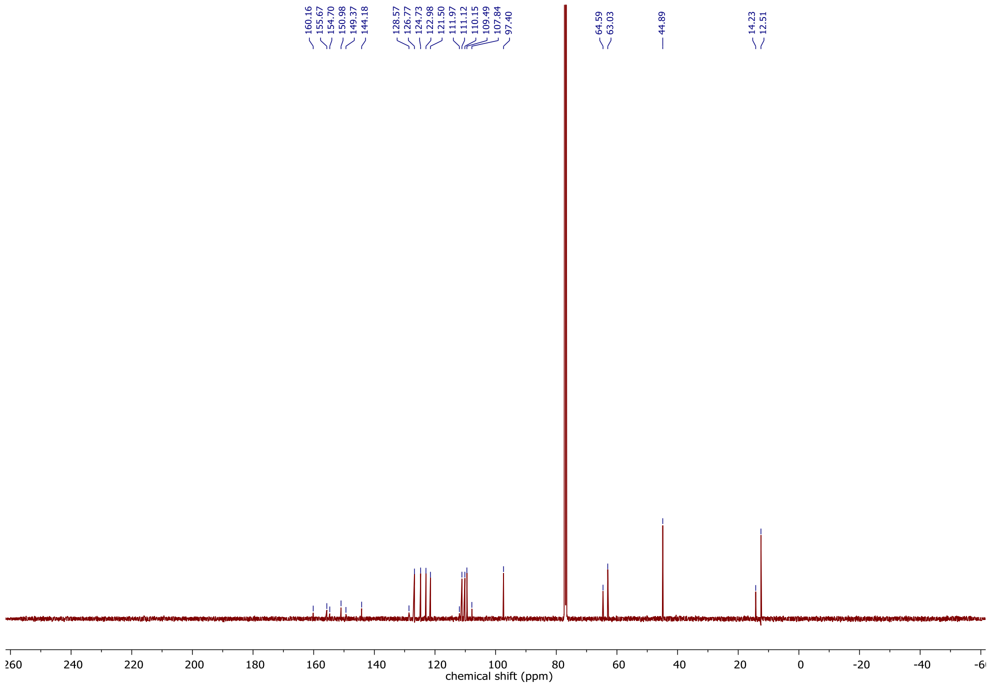
160.16 155.67 154.70 150.98 149.37 144.18
128.57 126.77 124.73 122.98 121.50 111.97 111.12 110.15 109.49 107.84 97.40
64.59 63.03
44.89
14.23 12.51
chemical shift (ppm)

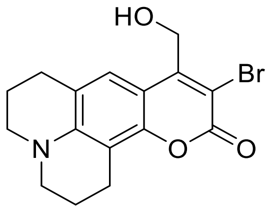
HO
Br
N
O
O

Compound 21

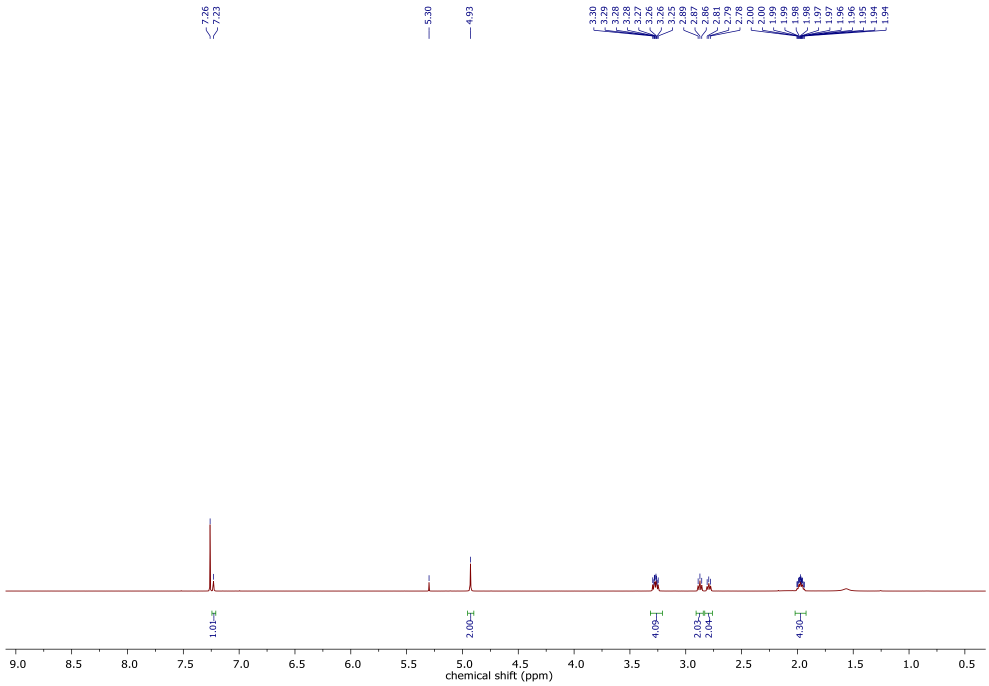
7.26
7.23
5.30
4.93
3.30 3.29 3.28 3.28 3.27 3.26 3.26 3.25 2.89 2.87 2.86 2.81 2.79 2.78 2.00 2.00 1.99 1.99 1.98 1.98 1.97 1.97 1.96 1.96 1.95 1.94 1.94
1.01
2.00
4.09
2.03
2.04
4.30
chemical shift (ppm)

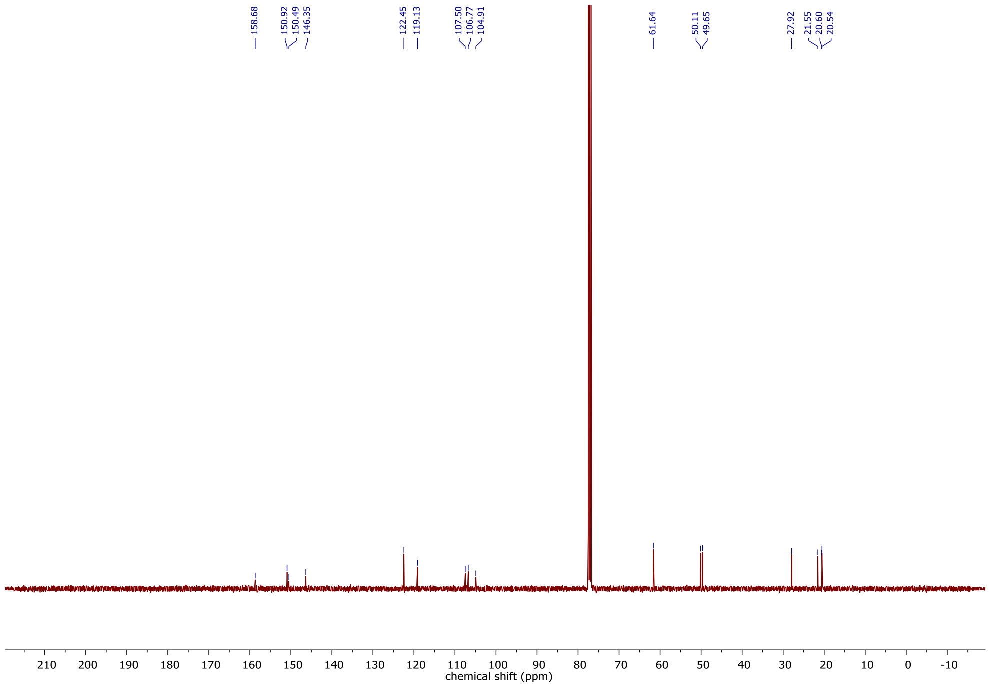
158.68
150.92
150.49
146.35
122.45
119.13
107.50
106.77
104.91
61.64
50.11
49.65
27.92
21.55
20.60
20.54
chemical shift (ppm)

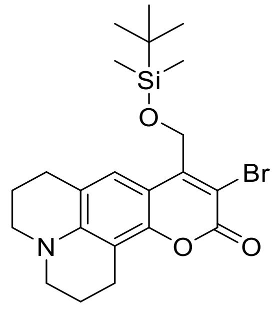
Si
O
Br
N
O
O

Compound 22

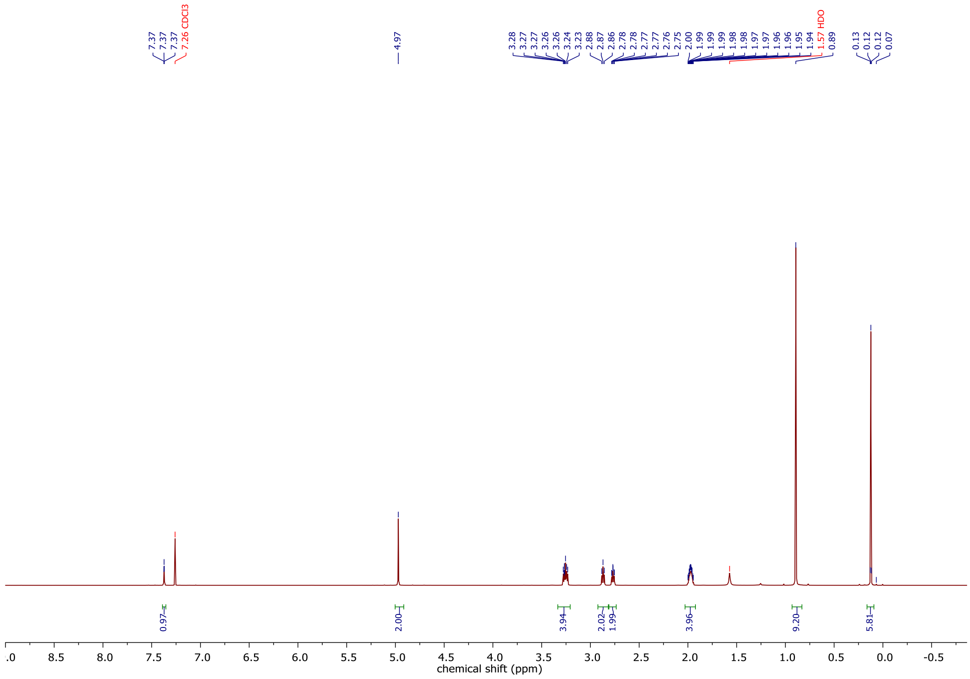
7.37
7.37
7.37
7.26 CDCl3
4.97
3.28
3.27
3.27
3.26
3.26
3.24
3.23
2.88
2.87
2.86
2.78
2.78
2.77
2.77
2.76
2.75
2.00
1.99
1.99
1.99
1.99
1.98
1.98
1.97
1.97
1.96
1.96
1.95
1.94
1.57 HDO
0.89
0.13
0.12
0.12
0.07
0.97
2.00
3.94
2.02
1.99
3.96
9.20
5.81
chemical shift (ppm)

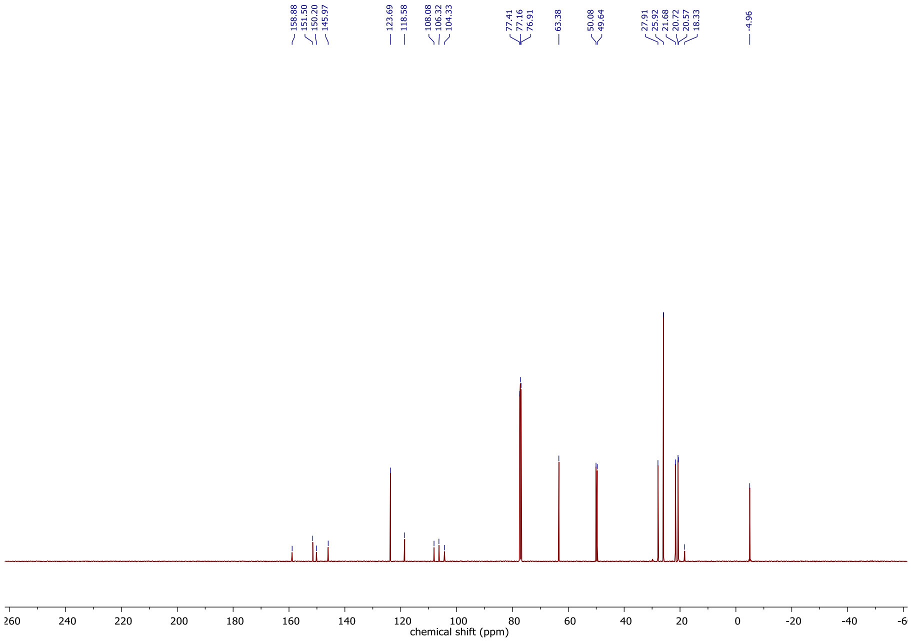
158.88
151.50
150.20
145.97
123.69
118.58
108.08
106.32
104.33
77.41
77.16
76.91
63.38
50.08
49.64
27.91
25.92
21.68
20.72
20.57
18.33
-4.96
chemical shift (ppm)

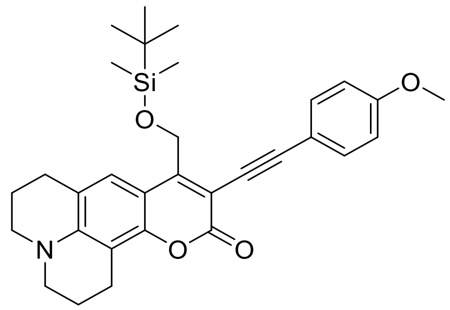

Compound 23-1

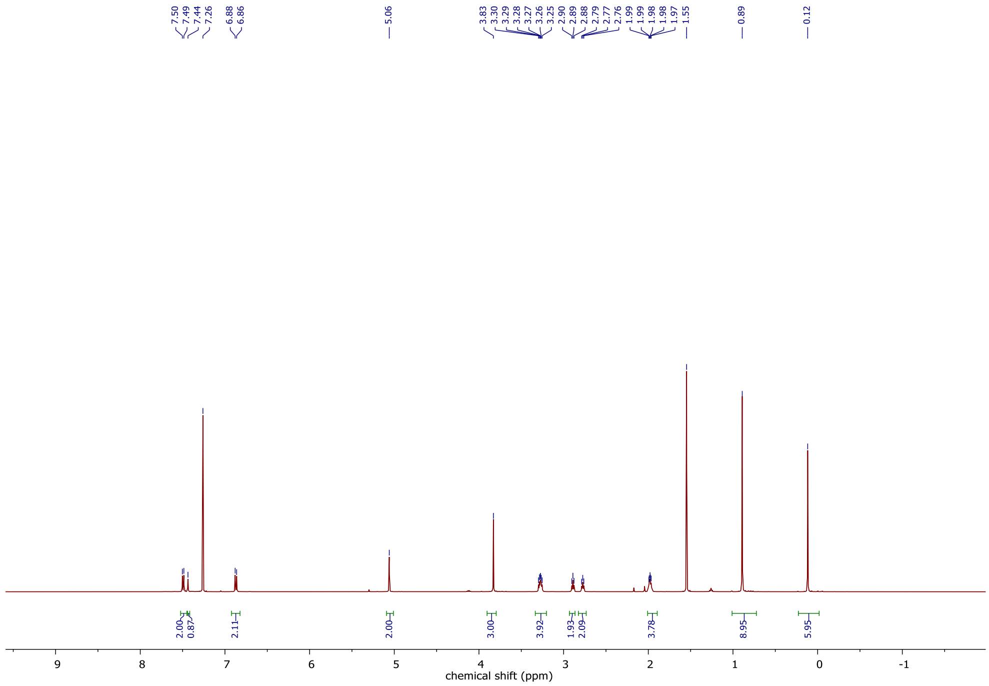

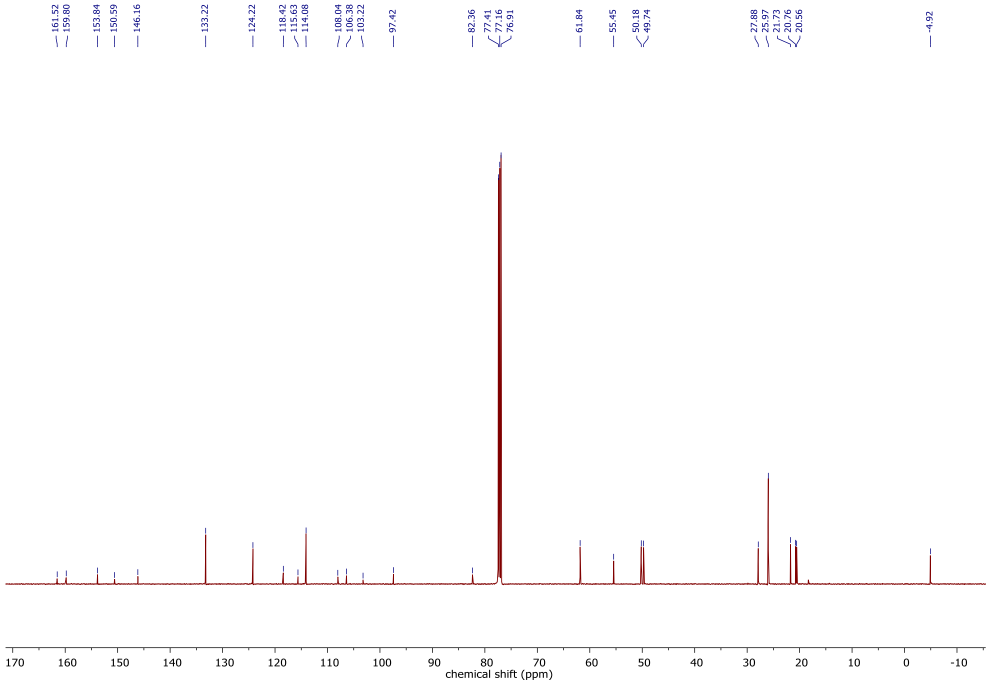

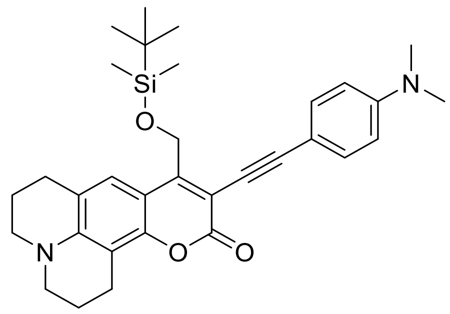

Compound 23-2

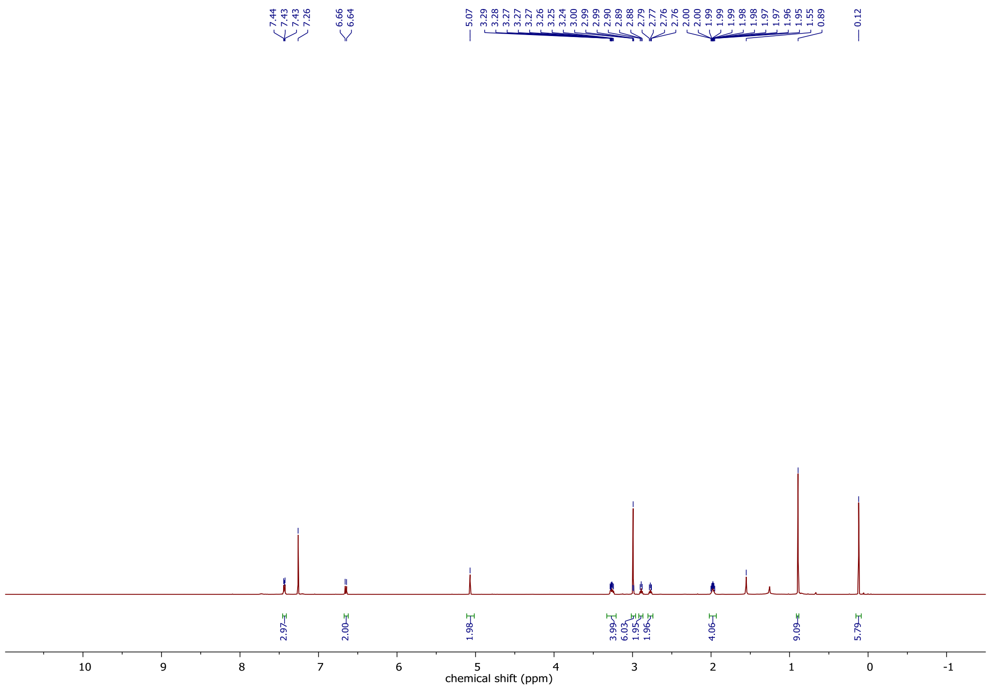

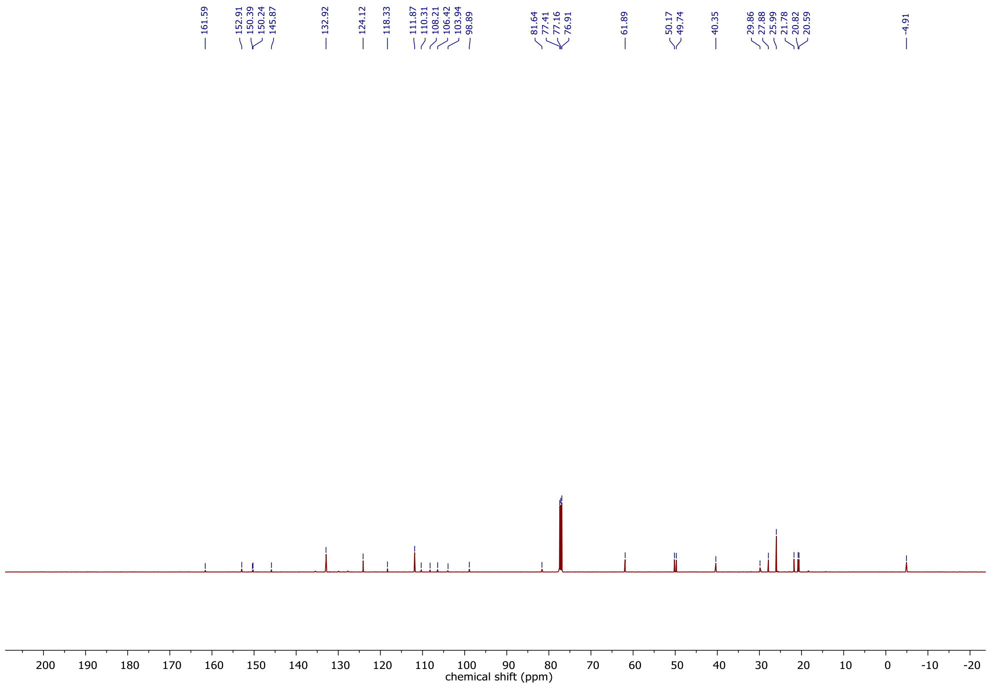

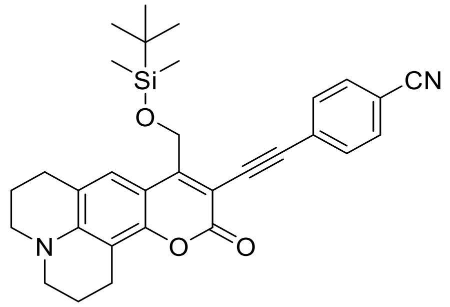

Compound 23-3

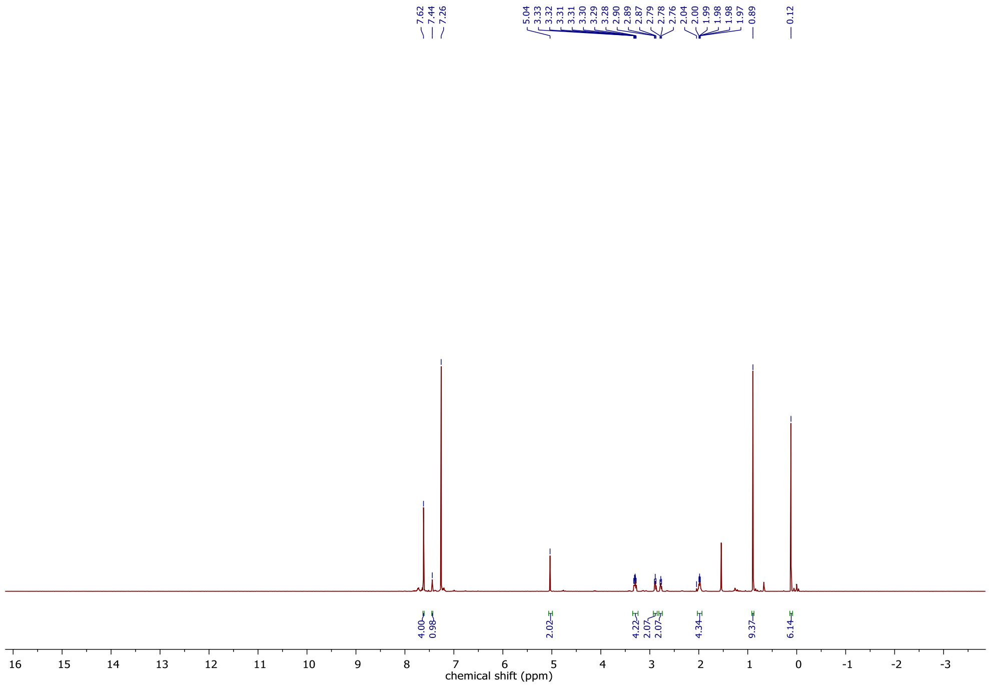

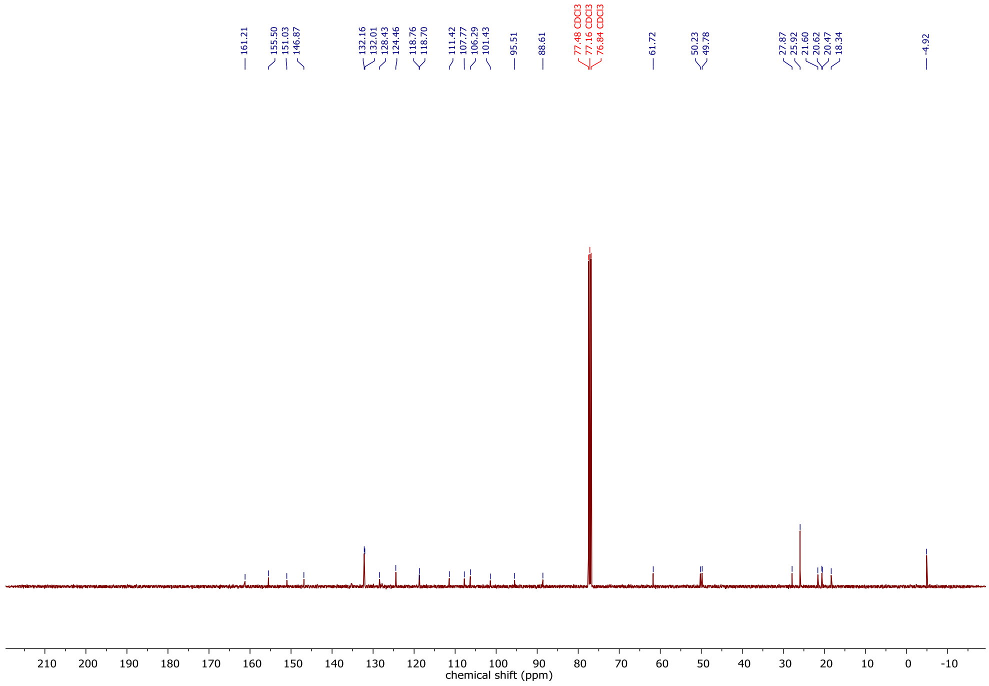

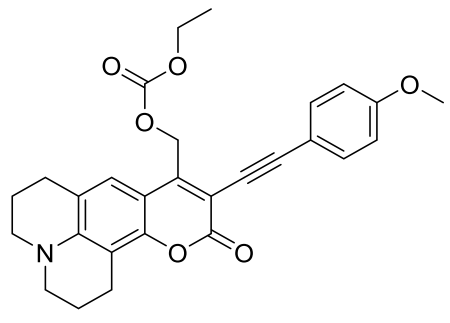
O
O
O
O
N
O
O
O

Cou 4a

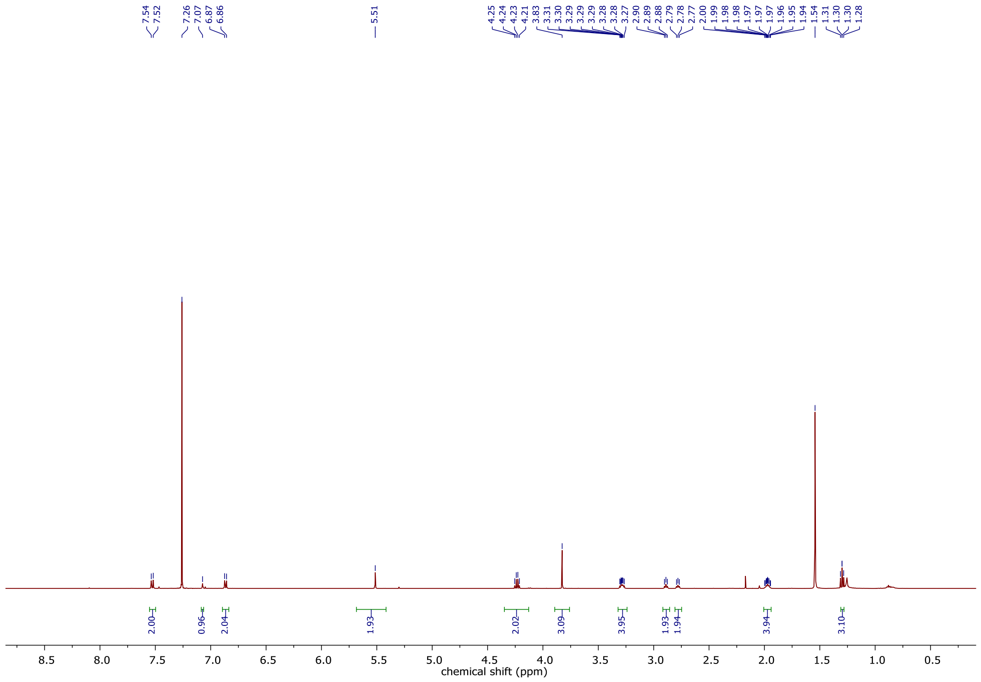
7.54
7.52
7.26
7.07
6.87
6.86
5.51
4.25
4.24
4.23
4.21
3.83
3.31
3.30
3.29
3.29
3.29
3.28
3.28
3.27
2.90
2.89
2.88
2.79
2.78
2.77
2.00
1.99
1.98
1.98
1.97
1.97
1.97
1.96
1.95
1.94
1.54
1.31
1.30
1.30
1.28
2.00
0.96
2.04
1.93
2.02
3.09
3.95
1.93
1.94
3.94
3.10
8.5
8.0
7.5
7.0
6.5
6.0
5.5
5.0
4.5
4.0
3.5
3.0
2.5
2.0
1.5
1.0
0.5
chemical shift (ppm)

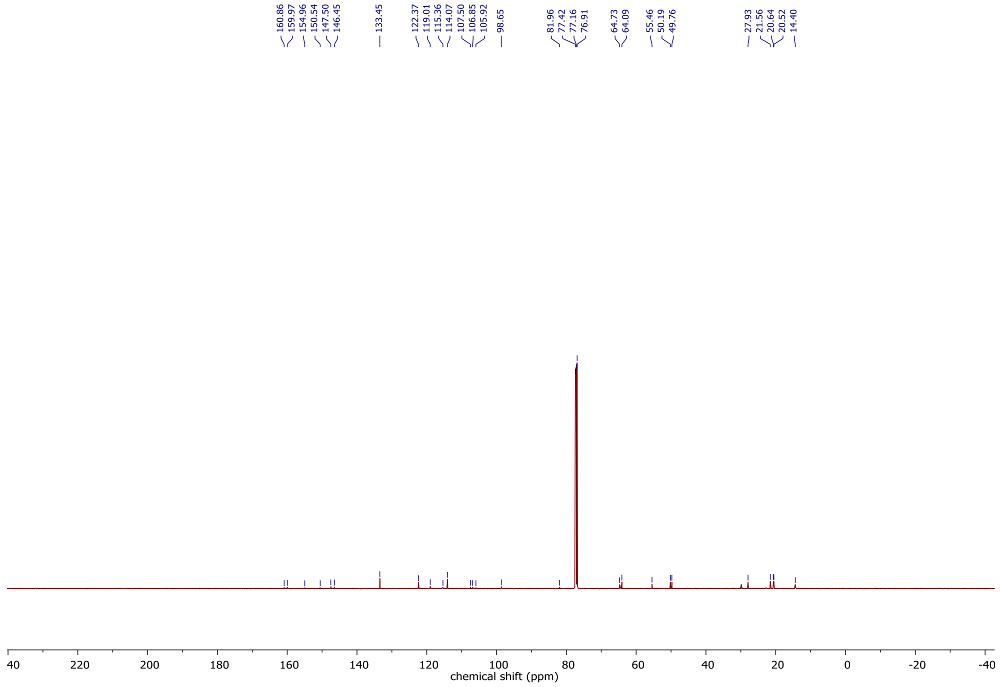
160.86
159.97
154.96
150.54
147.50
146.45
133.45
122.37
119.01
115.36
114.07
107.50
106.85
105.92
98.65
81.96
77.42
77.16
76.91
64.73
64.09
55.46
50.19
49.76
27.93
21.56
20.64
20.52
14.40
40
220
200
180
160
140
120
100
80
60
40
20
0
-20
-40
chemical shift (ppm)

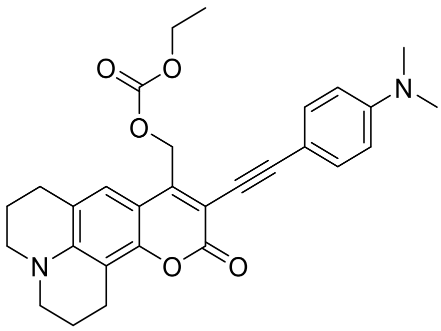

Cou 4b

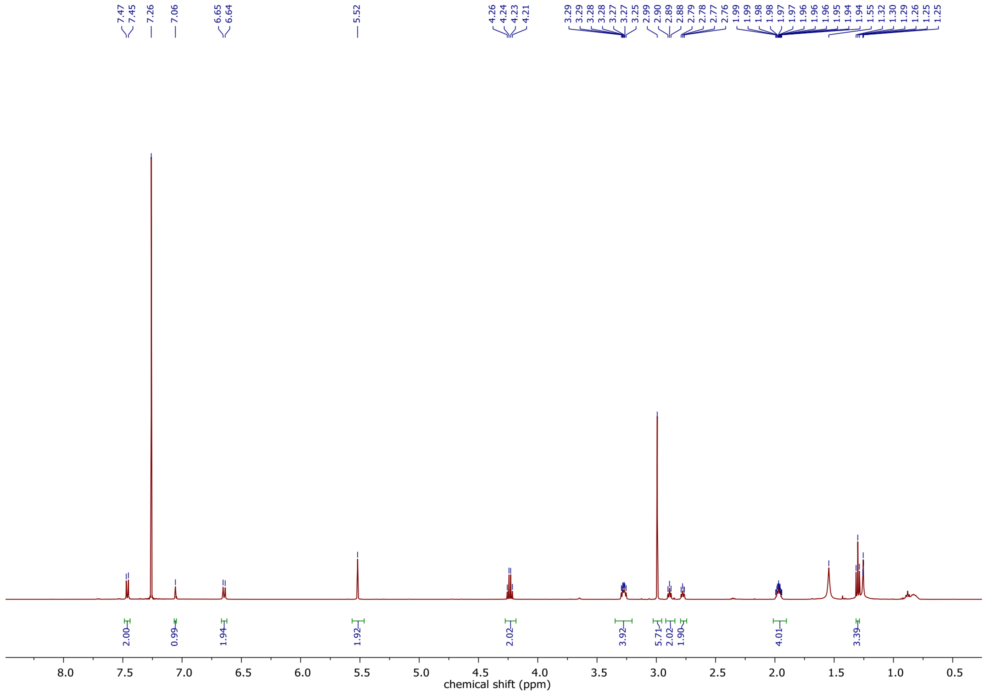

7.47
7.45
7.26
7.06
6.65
6.64
5.52
4.26
4.24
4.23
4.21
3.29
3.29
3.28
3.28
3.27
3.27
3.25
2.99
2.90
2.89
2.88
2.79
2.78
2.77
2.76
1.99
1.99
1.98
1.98
1.97
1.97
1.96
1.96
1.96
1.95
1.94
1.94
1.55
1.32
1.30
1.29
1.26
1.25
1.25
2.00
0.99
1.94
1.92
2.02
3.92
5.71
2.02
1.90
4.01
3.39
8.0 7.5 7.0 6.5 6.0 5.5 5.0 4.5 4.0 3.5 3.0 2.5 2.0 1.5 1.0 0.5
chemical shift (ppm)

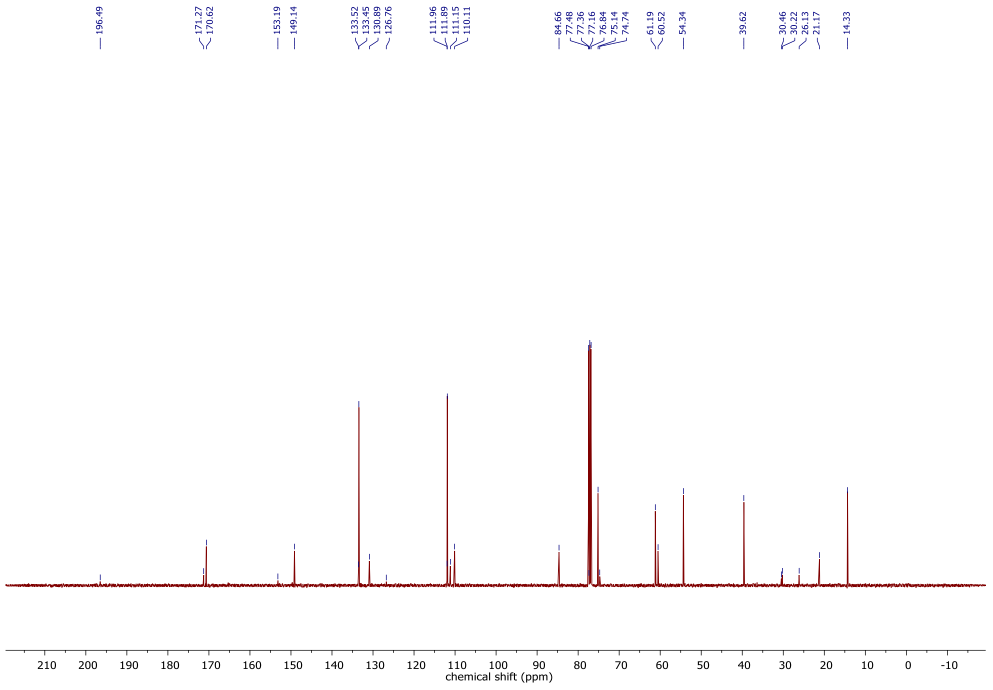

196.49
171.27
170.62
153.19
149.14
133.52
133.45
130.89
126.76
111.96
111.89
111.15
110.11
84.66
77.48
77.36
77.16
76.84
75.14
74.74
61.19
60.52
54.34
39.62
30.46
30.22
26.13
21.17
14.33
210 200 190 180 170 160 150 140 130 120 110 100 90 80 70 60 50 40 30 20 10 0 -10
chemical shift (ppm)

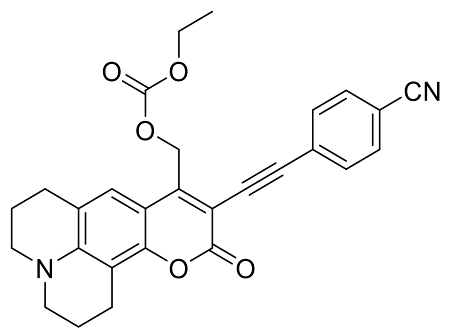

CN
O
O
O
N
O
O

Cou 4c

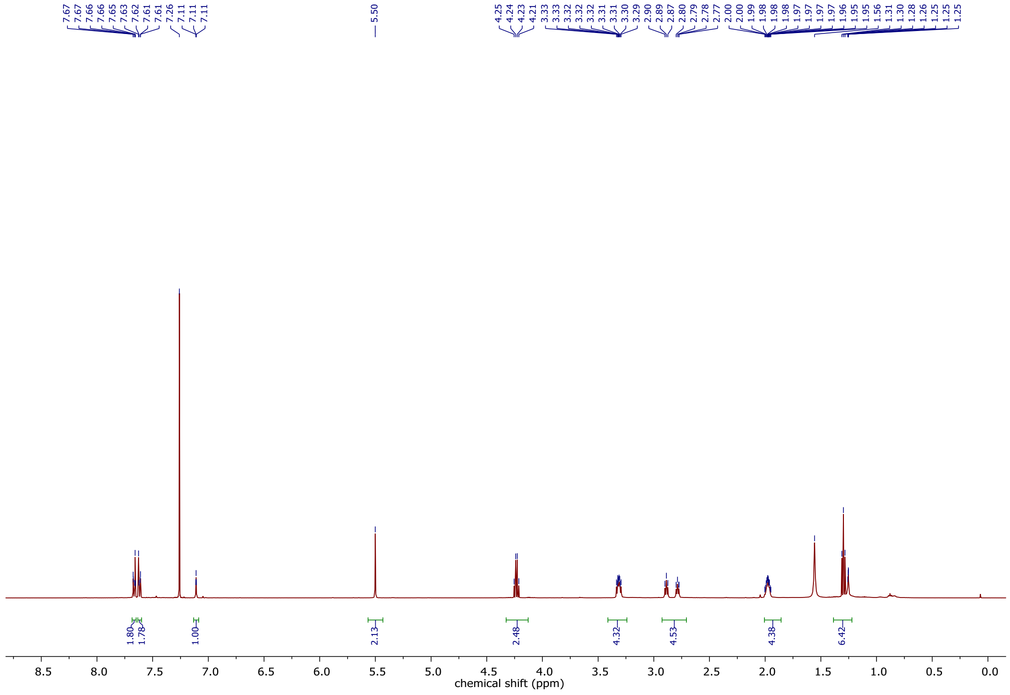

7.67
7.67
7.66
7.66
7.65
7.63
7.62
7.61
7.61
7.26
7.11
7.11
7.11
5.50
4.25
4.24
4.23
4.23
4.21
3.33
3.33
3.32
3.32
3.32
3.31
3.31
3.30
3.29
2.90
2.89
2.87
2.80
2.79
2.78
2.77
2.00
2.00
1.99
1.98
1.98
1.98
1.97
1.97
1.97
1.96
1.95
1.95
1.56
1.31
1.30
1.28
1.26
1.25
1.25
1.80
1.78
1.00
2.13
2.48
4.32
4.53
4.38
6.42
8.5 8.0 7.5 7.0 6.5 6.0 5.5 5.0 4.5 4.0 3.5 3.0 2.5 2.0 1.5 1.0 0.5 0.0
chemical shift (ppm)

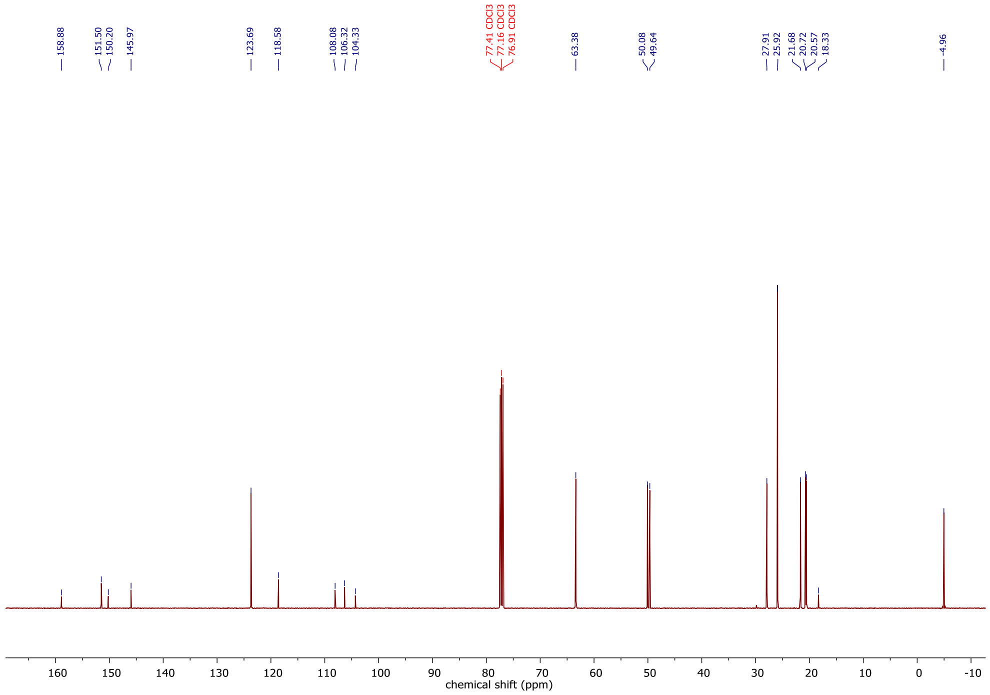

158.88
151.50
150.20
145.97
123.69
118.58
108.08
106.32
104.33
77.41 CDCl3
77.16 CDCl3
76.91 CDCl3
63.38
50.08
49.64
27.91
25.92
21.68
20.72
20.57
18.33
-4.96
160 150 140 130 120 110 100 90 80 70 60 50 40 30 20 10 0 -10
chemical shift (ppm)

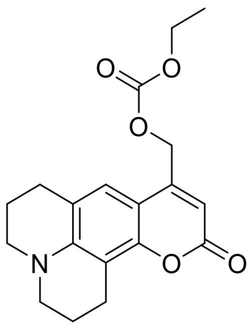
O
O
O
N
O
O

Cou 4d

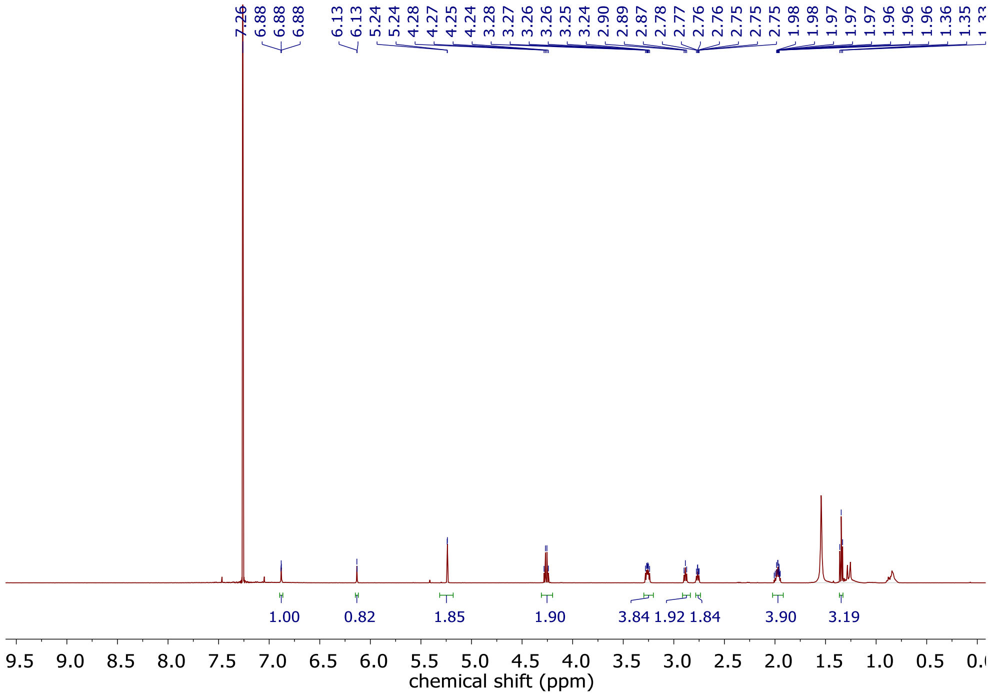
7.26
6.88
6.88
6.88
6.13
6.13
5.24
5.24
4.28
4.27
4.25
4.24
3.28
3.27
3.26
3.26
3.25
3.24
2.90
2.89
2.87
2.78
2.77
2.76
2.76
2.75
2.75
2.75
1.98
1.98
1.97
1.97
1.97
1.96
1.96
1.96
1.36
1.35
1.00
0.82
1.85
1.90
3.84
1.92
1.84
3.90
3.19
9.5 9.0 8.5 8.0 7.5 7.0 6.5 6.0 5.5 5.0 4.5 4.0 3.5 3.0 2.5 2.0 1.5 1.0 0.5
chemical shift (ppm)

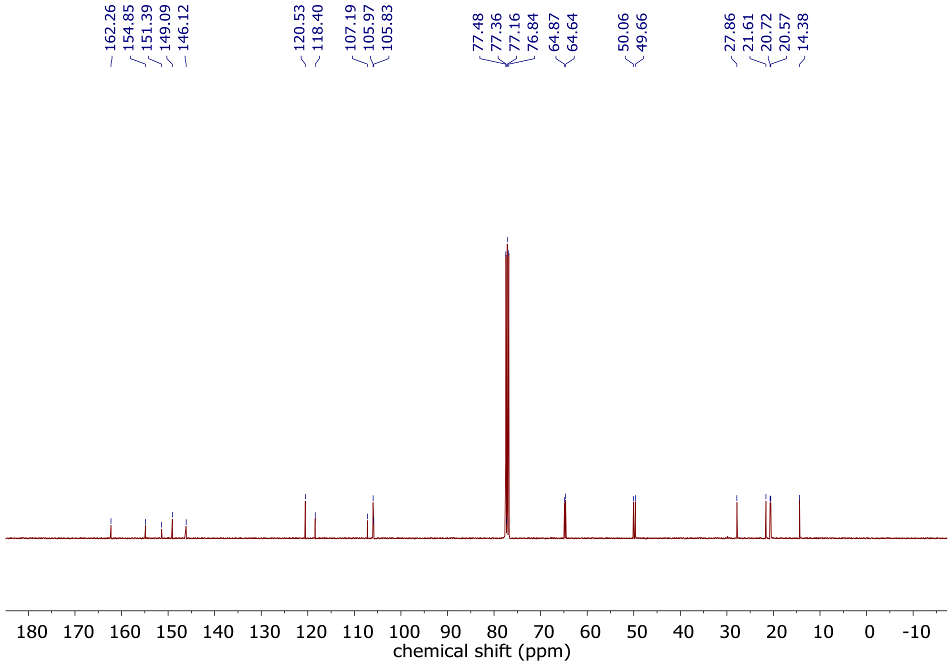
162.26
154.85
151.39
149.09
146.12
120.53
118.40
107.19
105.97
105.83
77.48
77.36
77.16
76.84
64.87
64.64
50.06
49.66
27.86
21.61
20.72
20.57
14.38
180 170 160 150 140 130 120 110 100 90 80 70 60 50 40 30 20 10 0 -10
chemical shift (ppm)

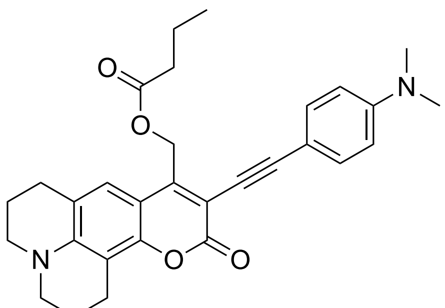

cCTL

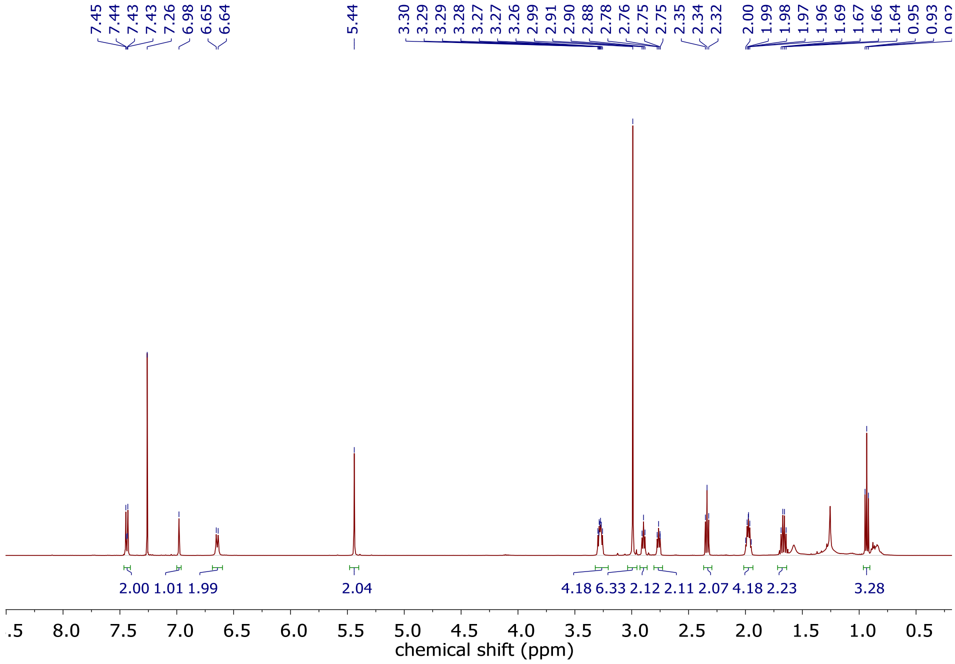
7.45 7.44 7.43 7.43 7.26 6.98 6.65 6.64
5.44
3.30 3.29 3.29 3.28 3.27 3.27 3.26 2.99 2.91 2.90 2.88 2.78 2.76 2.75 2.75 2.35 2.34 2.32
2.00 1.99 1.98 1.97 1.96 1.69 1.67 1.66 1.64 0.95 0.93
2.00 1.01 1.99 2.04 4.18 6.33 2.12 2.11 2.07 4.18 2.23 3.28
chemical shift (ppm)

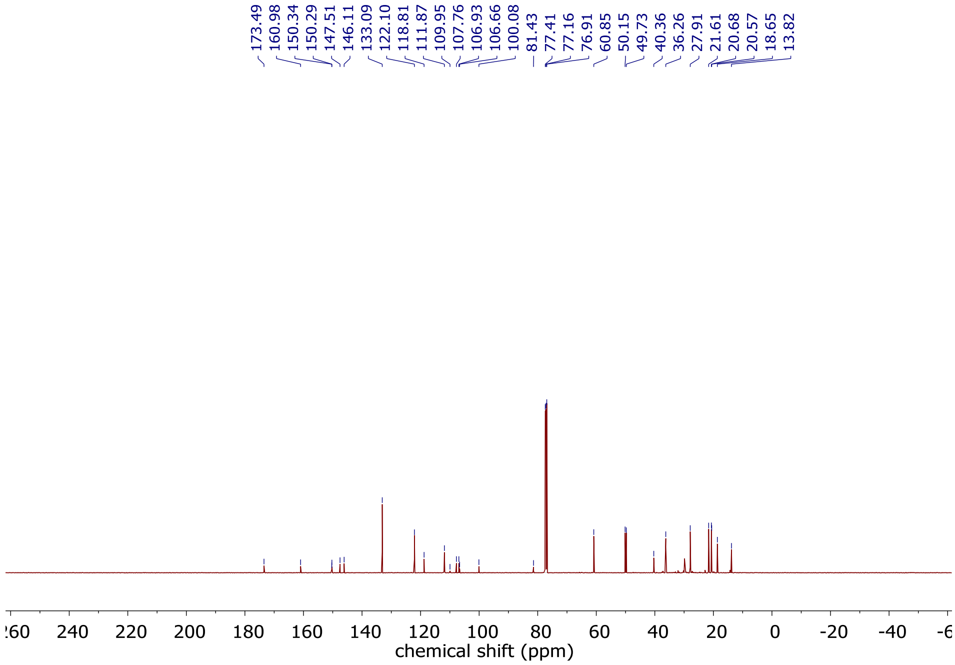
173.49 160.98 150.34 150.29 147.51 146.11 133.09 122.10 118.81 111.87 109.95 107.76 106.93 106.66 100.08
81.43 77.41 77.16 76.91 60.85 50.15 49.73 40.36 36.26 27.91 21.61 20.68 20.57 18.65 13.82
chemical shift (ppm)

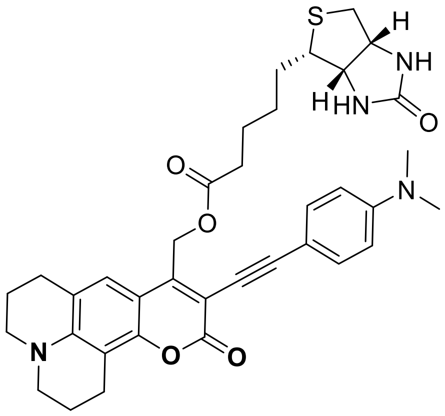

cBiotin

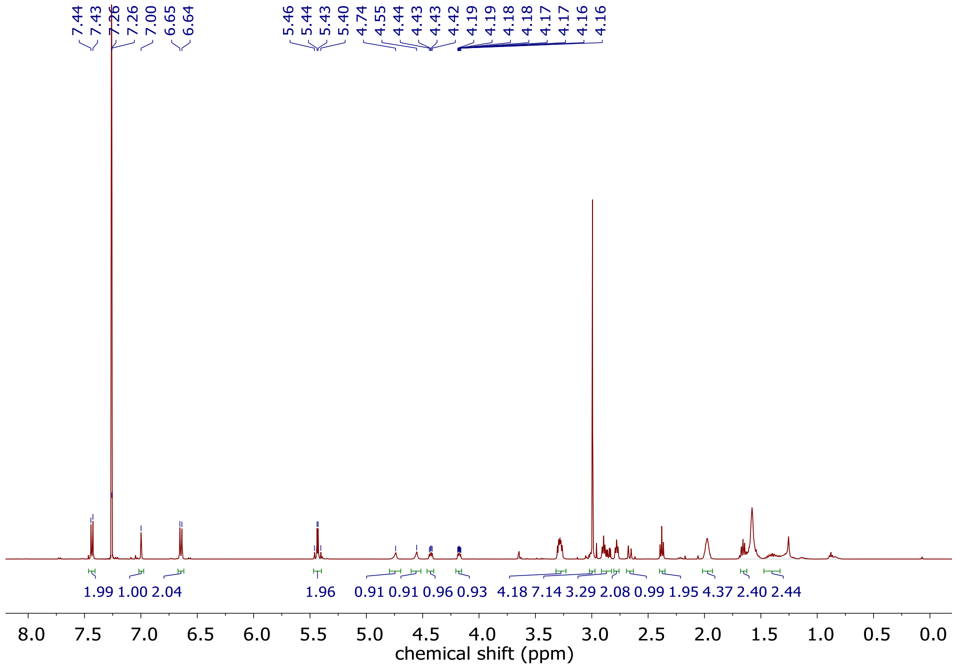

7.44 7.43 7.26 7.26 7.00 6.65 6.64
5.46 5.44 5.43 5.40 4.74 4.55 4.44 4.43 4.43 4.42 4.19 4.19 4.18 4.18 4.17 4.17 4.16 4.16
1.99 1.00 2.04 1.96 0.91 0.91 0.96 0.93 4.18 7.14 3.29 2.08 0.99 1.95 4.37 2.40 2.44
8.0 7.5 7.0 6.5 6.0 5.5 5.0 4.5 4.0 3.5 3.0 2.5 2.0 1.5 1.0 0.5 0.0
chemical shift (ppm)

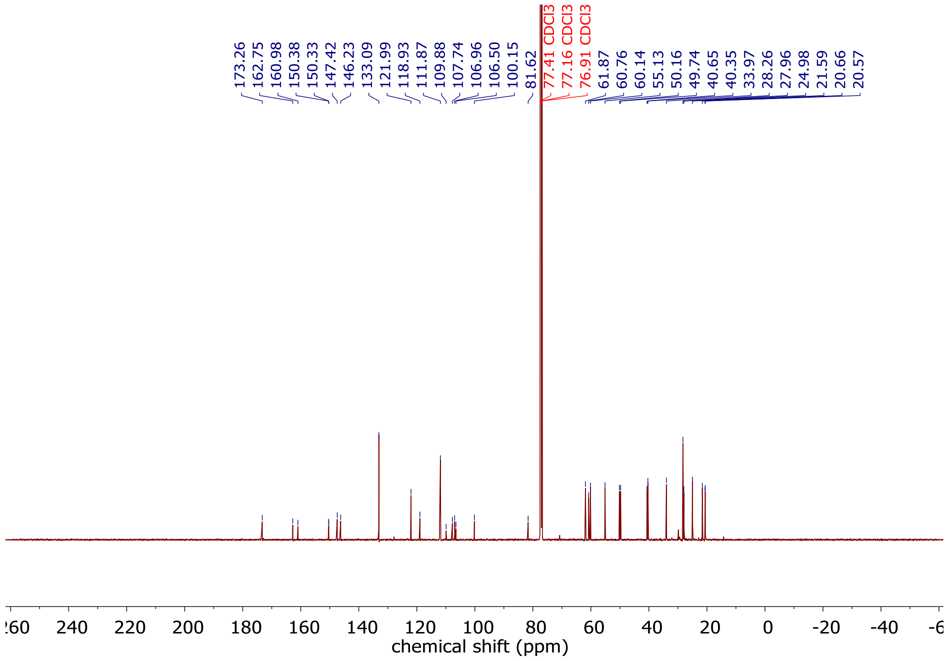

173.26 162.75 160.98 150.38 150.33 147.42 146.23 133.09 121.99 118.93 111.87 109.88 107.74 106.96 106.50 100.15 81.62
77.41 CDCl3 77.16 CDCl3 76.91 CDCl3
61.87 60.76 60.14 55.13 50.16 49.74 40.65 40.35 33.97 28.26 27.96 24.98 21.59 20.66 20.57
240 220 200 180 160 140 120 100 80 60 40 20 0 -20 -40
chemical shift (ppm)

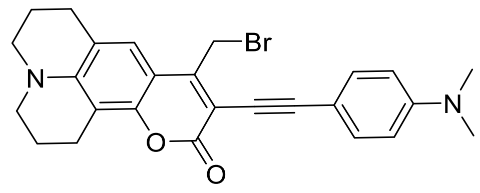
Br
N
O
O
N

Cou 5

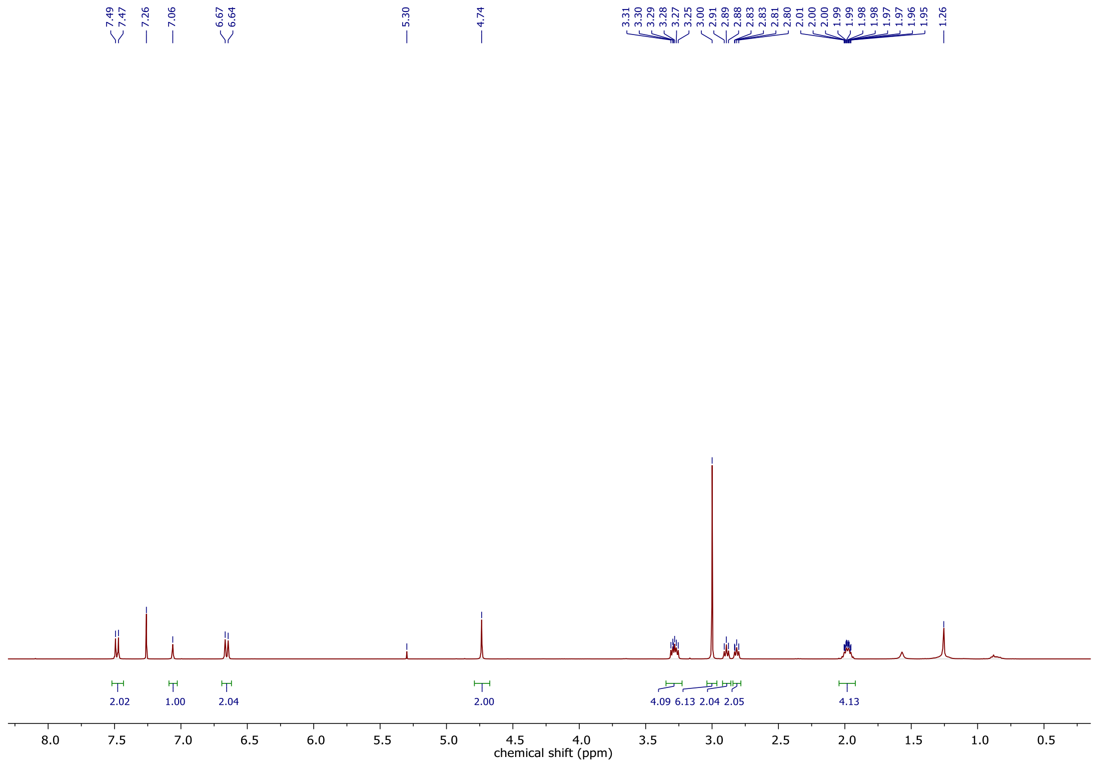
7.49
7.47
7.26
7.06
6.67
6.64
5.30
4.74
3.31
3.30
3.29
3.28
3.27
3.25
3.00
2.91
2.89
2.88
2.83
2.83
2.81
2.80
2.01
2.00
2.00
1.99
1.99
1.98
1.98
1.97
1.97
1.96
1.95
1.26
2.02
1.00
2.04
2.00
4.09
6.13
2.04
2.05
4.13
chemical shift (ppm)

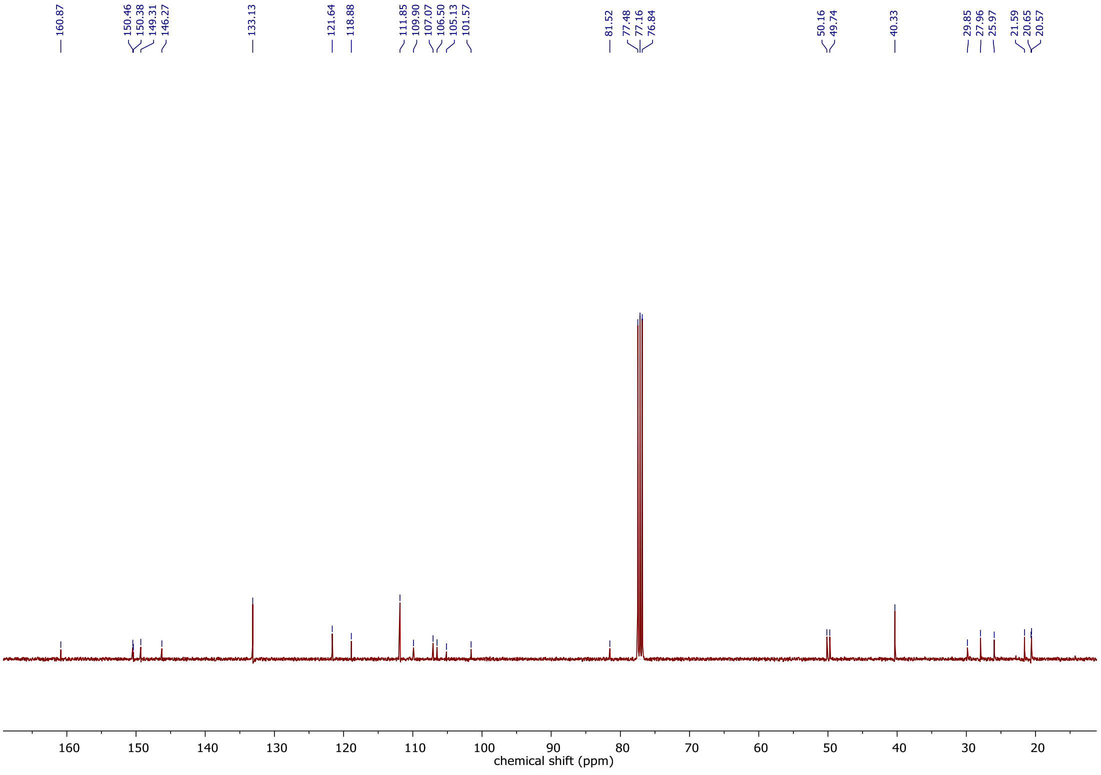
160.87
150.46
150.38
149.31
146.27
133.13
121.64
118.88
111.85
109.90
107.07
106.50
105.13
101.57
81.52
77.48
77.16
76.84
50.16
49.74
40.33
29.85
27.96
25.97
21.59
20.65
20.57
chemical shift (ppm)

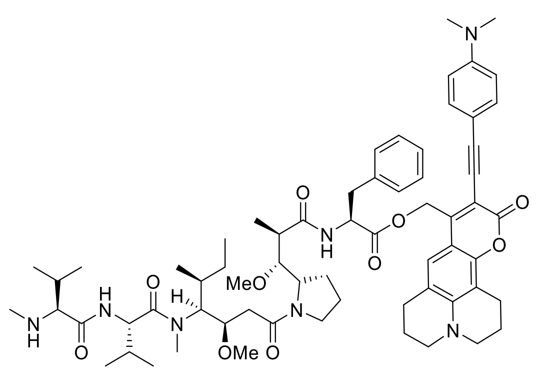

cMMAF

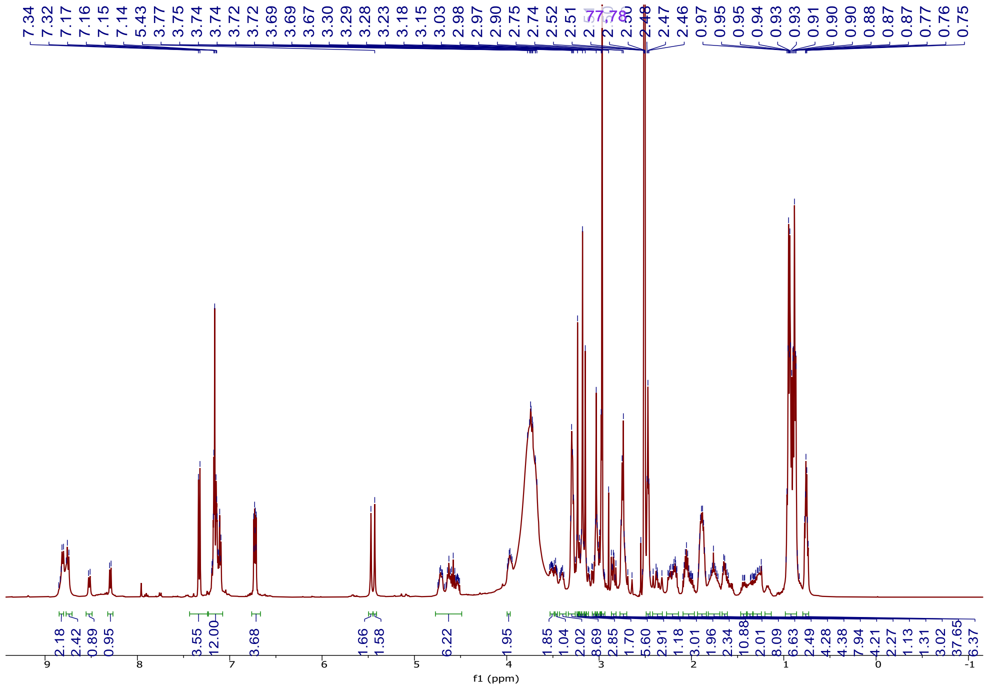
7.34
7.32
7.17
7.16
7.15
7.14
5.43
3.77
3.75
3.74
3.74
3.72
3.72
3.69
3.69
3.67
3.30
3.29
3.28
3.23
3.18
3.15
3.03
2.98
2.97
2.90
2.75
2.74
2.52
2.51
2.47
2.46
0.97
0.95
0.94
0.93
0.93
0.91
0.90
0.90
0.88
0.87
0.77
0.76
0.75
2.18
2.42
0.89
0.95
3.55
12.00
3.68
1.66
1.58
6.22
1.95
1.85
1.04
2.02
3.69
2.85
1.70
5.60
2.91
1.18
3.01
1.96
2.34
10.88
2.01
8.09
6.63
2.49
4.28
4.38
7.94
4.21
2.27
1.13
1.31
3.02
37.65
6.37
9
8
7
6
5
4
3
2
1
0
-1
f1 (ppm)

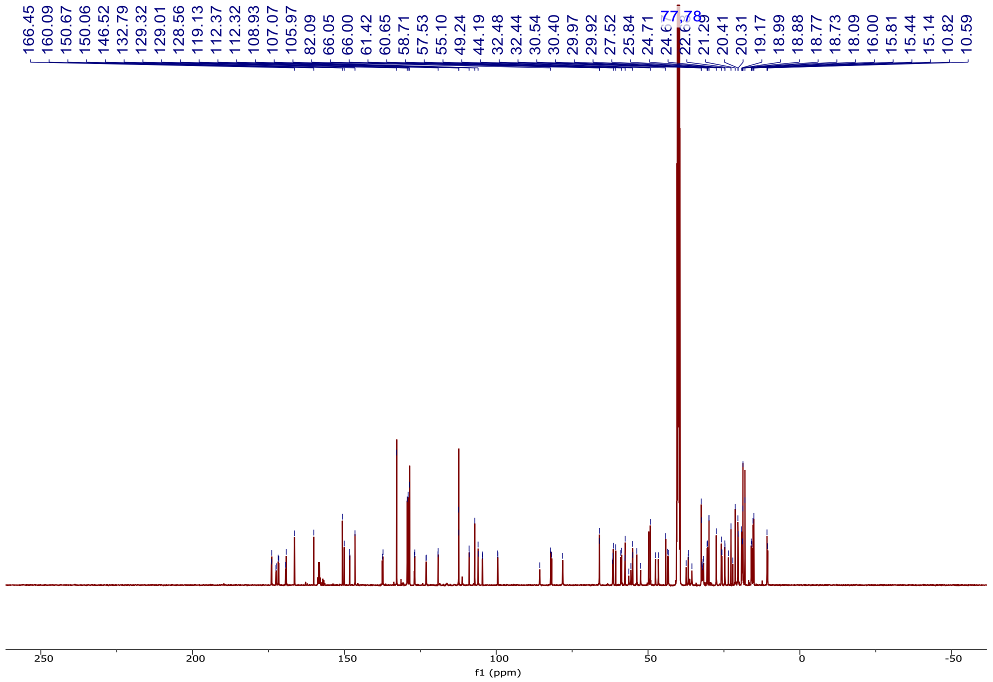
166.45
160.09
150.67
150.06
146.52
132.79
129.32
129.01
128.56
119.13
112.37
112.32
108.93
107.07
105.97
82.09
66.05
66.00
61.42
60.65
58.71
57.53
55.10
49.24
44.19
32.48
32.44
30.54
30.40
29.97
29.92
27.52
25.84
24.71
21.29
20.41
20.31
19.17
18.99
18.88
18.77
18.73
18.09
16.00
15.81
15.44
15.14
10.82
10.59
250
200
150
100
50
0
-50
f1 (ppm)
